# Stereoselective, Ruthenium-Photocatalyzed Synthesis of 1,2-Diaminotruxinic Bis-amino Acids from 4-Arylidene-5(4*H*)-oxazolones

**DOI:** 10.1021/acs.joc.1c03092

**Published:** 2022-02-10

**Authors:** Sonia Sierra, M. Victoria Gomez, Ana I. Jiménez, Alexandra Pop, Cristian Silvestru, Maria Luisa Marín, Francisco Boscá, Germán Sastre, Enrique Gómez-Bengoa, Esteban P. Urriolabeitia

**Affiliations:** † Instituto de Síntesis Química y Catálisis Homogénea, ISQCH (CSIC-Universidad de Zaragoza), Pedro Cerbuna 12, 50009 Zaragoza, Spain; ‡ Instituto Regional de Investigación Científica Aplicada (IRICA), Universidad de Castilla-La Mancha, Avenida Camilo José Cela s/n, 13071 Ciudad Real, Spain; § Department of Chemistry, Supramolecular Organic and Organometallic Chemistry Centre (SOOMCC), Faculty of Chemistry and Chemical Engineering, Babeş-Bolyai University, 11 Arany Janos, 400028 Cluj-Napoca, Romania; ∥ Instituto Universitario Mixto de Tecnología Química (ITQ-UPV), Universitat Politècnica de València-Consejo Superior de Investigaciones Científicas, Av. de los Naranjos s/n, 46022 Valencia, Spain; ⊥ Departamento de Química Orgánica I, Universidad del País Vasco, UPV-EHU, Apdo. 1072, CP-20080 Donostia-San Sebastián, Spain

## Abstract

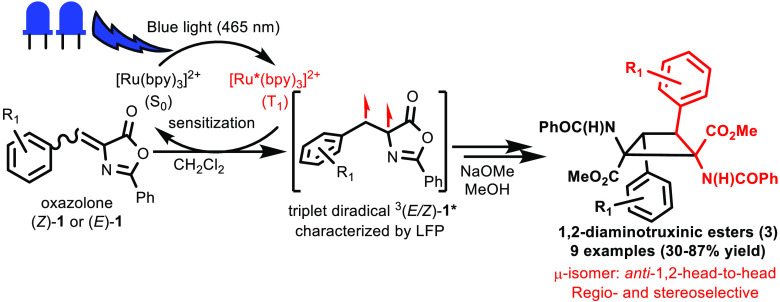

The irradiation of (*Z*)-2-phenyl-4-aryliden-5(4*H*)-oxazolones **1** in deoxygenated CH_2_Cl_2_ at 25 °C with blue light (465 nm) in the presence of [Ru(bpy)_3_](BF_4_)_2_ (5% mole ratio) as a triplet photocatalyst promotes the [2+2] photocycloaddition of the C=C bonds of the 4-arylidene moiety, thus allowing the completely regio- and stereoselective formation of cyclobutane-bis(oxazolone)s **2** as single stereoisomers. Cyclobutanes **2** have been unambiguously characterized as the μ-isomers and contain two *E*-oxazolones coupled in an *anti*-head-to-head form. The use of continuous-flow techniques in microreactors allows the synthesis of cyclobutanes **2** in only 60 min, compared with the 24–48 h required in batch mode. Ring opening of the oxazolone heterocycle in **2** with a base affords the corresponding 1,2-diaminotruxinic bis-amino esters **3**, which are also obtained selectively as μ-isomers. The ruthenium complex behaves as a triplet photocatalyst, generating the reactive excited state of the oxazolone via an energy-transfer process. This reactive excited state has been characterized as a triplet diradical ^3^(*E*/*Z*)-1* by laser flash photolysis (transient absorption spectroscopy). This technique also shows that this excited state is the same when starting from either (*Z*)- or (*E*)-oxazolones. Density functional theory calculations show that the first step of the [2+2] cycloaddition between ^3^(*E/Z*)-1* and (*Z*)-**1** is formation of the C(H)–C(H) bond and that (*Z*) to (*E*) isomerization takes place at the 1,4-diradical thus formed.

## Introduction

The synthesis of organic compounds by photocatalysis is becoming increasingly popular due to the intrinsic advantages of this methodology.^1−8^ Indeed, the use of visible light as a renewable source of energy to reach high-energy excited states that cannot be achieved using conventional thermal methods, the different reactivity of the excited states with respect to the ground state, the virtual absence of waste material, and the high atom economy and efficiency of these reactions make photochemical processes attractive from the point of view of a sustainable synthetic methodology.^1−8^ The synthesis of cyclobutanes by [2+2] photocycloaddition of alkenes is a paradigm of photochemical reactions due to the versatility, high efficiency, and atom economy of the process, and the mild reaction conditions required.^9^ Cyclobutanes are compounds of interest because they are intermediates in organic synthesis, especially in ring-expansion or ring-opening reactions,^10^ and because they are present in many natural products.^11^ Despite this interest, alternative synthetic methods for the synthesis of cyclobutanes are scarce and, in general, do not provide simple access to customized complex scaffolds.^12^

**Figure 1:**
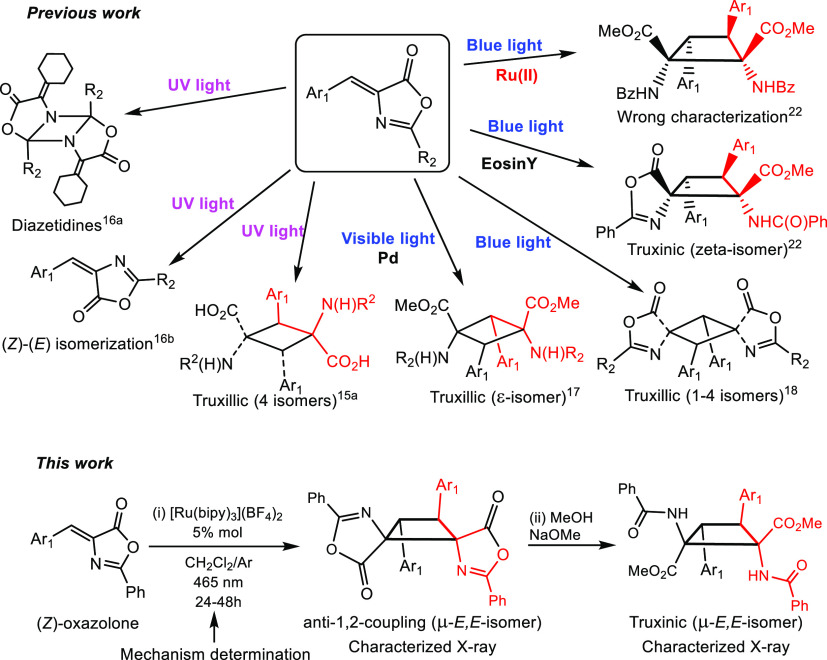
Previous studies of the synthesis of 1,3-diaminotruxillic derivatives from oxazolones and work reported here on the synthesis of 1,2-diaminotruxinic esters.

1,3-Diaminotruxillic and 1,2-diaminotruxinic species make up a very interesting group of bis-amino acids with a cyclobutane core (Figure 1). Both derivatives, among other structurally related compounds, are found in coca leaves (*Erythroxylum coca* and *Erythroxylum truxillense*) in very low concentrations^13^ and have been known since the late 19th century due to their pharmacological properties. More specifically, they show strong antinociceptive and anti-inflammatory activities.^14^ Similarly, recent reports have described them as the only nonpeptidic agonists of GLP-1R (glucagon-like peptide 1 receptor), which is widely used in the treatment of diabetes.^15^ Despite their importance and properties, the availability of synthetic methods for accessing 1,3-diaminotruxillic and 1,2-diaminotruxinic derivatives is somewhat limited.

The most straightforward synthetic pathway for the construction of 1,3-diaminotruxillic and 1,2-diaminotruxinic scaffolds seems to be the [2+2] photocycloaddition of 4-aryliden-5(4*H*)-oxazolones, as this type of cyclobutane should be obtained in just one step. However, previous studies of the photochemical reactivity of 4-aryliden-5(4*H*)-oxazolones gave a variety of results, as shown in Figure 1. The formation of diazetidines^16a^ or (*Z*) to (*E*) isomerization^16b^ has been reported under UV irradiation, instead of the expected [2+2] photocycloaddition. Using a 500 W ultraviolet high-pressure mercury lamp, Wang et al. reported a few examples of the metal-free [2+2] photocycloaddition of oxazolones to give 1,3-diaminotruxillic derivatives in low yields (10%) as a mixture of four isomers.^15a^ Recently, our group reported a synthetic methodology for obtaining 1,3-diaminotruxillic derivatives regioselectively in good yields based on the use of orthopalladated complexes as templates.^17^ This synthesis involves orthopalladation of the oxazolone, followed by [2+2] photocycloaddition promoted by blue light (465 nm), and release of the 1,3-diaminotruxillic as the ε-isomer by hydrogenation,^17a,17b^ oxidation,^17c^ or carbonylation^17d^ and methanolysis. The use of blue light provided by low-power LEDs has also allowed us to markedly improve the metal-free photocycloaddition of oxazolones. In this respect, we have recently reported the synthesis of 1,3-diaminotruxillics, expanding the scope, reducing the reaction times, and achieving full conversions with remarkable selectivity considering that the reactions occur in solution.^18^ Solid-state [2+2] cycloadditions take place with good regioselectivity when the topochemical Schmidt’s conditions are achieved.^19^ However, the corresponding reactions in solution usually afford several stereoisomers,^20^ unless chiral photocatalysts or enantiomerically pure templates or reagents are used.^7,21^ Therefore, the development of highly regio- and stereoselective photochemical processes in solution is still challenging.

While the selective synthesis of 1,3-diaminotruxillic cyclobutanes has attracted some attention, preparation of the corresponding 1,2-diaminotruxinic derivatives remains virtually unexplored. To the best of our knowledge, there is only one contribution reporting the synthesis of such cyclobutanes from irradiation of (*Z*)-4-aryliden-2-aryl-5(4*H*)-oxazolones (Figure 1) with two different photocatalysts, eosin Y and [Ru(bpy)_3_](PF_6_)_2_.^22^ When eosin Y was used, the ζ-isomer was obtained via the head-to-head *anti*-1,2-coupling of one (*Z*)-oxazolone and one (*E*)-oxazolone.^22^ However, when [Ru(bpy)_3_](PF_6_)_2_ was the photocatalyst, the obtained 1,2-diaminotruxinic derivatives were not accurately characterized.^22^ In this case, the authors propose the formation of the same ζ-isomer (which lacks symmetry elements), but the NMR data provided show unambiguously that a symmetric species is obtained.^22^ Furthermore, a mechanism for the eosin Y-photocatalyzed reaction through a photoredox pathway was proposed, which involved the formation of the radical anion of the oxazolone (although without experimental evidence), but no mention at all is made to the mechanism operating in the case of [Ru(bpy)_3_](PF_6_)_2_ as a photocatalyst. Therefore, it is clear that the Ru-catalyzed [2+2] photocycloaddition of oxazolones is poorly characterized, regarding both the geometry of the obtained isomer and the mechanism taking place.

To shed light on the Ru-photocatalyzed reaction, due to the intrinsic interest of 1,2-diaminotruxinic derivatives, and our own interest in bis-amino acids with cyclobutane skeleton,^17,18^ we have investigated in depth the photocycloaddition of (*Z*)-4-aryliden-5(4*H*)-oxazolones in the presence of [Ru(bpy)_3_](BF_4_)_2_ as a triplet photocatalyst.^23^ Herein, we present the fully regio- and stereoselective Ru-photocatalyzed synthesis of methyl esters of 1,2-diaminotruxinic acid derivatives **3** by [2+2] photocycloaddition of (*Z*)-4-aryliden-5(4*H*)-oxazolones. The 1,2-diaminotruxinic ester derivatives are obtained as unique diastereoisomers, fully characterized as the μ-isomer, which contains two (*E*)-oxazolones coupled in a 1,2-head-to-head *anti* form. The wide scope of the reaction, the huge increase in the reaction rate achieved in flow reactors, the characterization of the reactive excited state of the oxazolone as a triplet state, and a plausible reaction mechanism based on laser flash photolysis evidence and density functional theory (DFT) methods, including elucidation of the role of the Ru(II) species, are new findings presented and discussed herein. All of these facts show that the synthesis of cyclobutanes in solution, with complete selectivity, is possible in the absence of additives or templates.

## Results and Discussion

### Synthesis and Characterization of Cyclobutane-bis(oxazolone)s **2**

**Figure 2:**
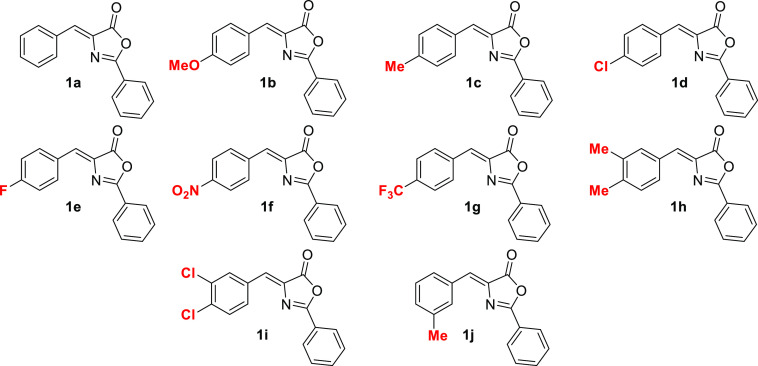
(*Z*)-4-Aryliden-2-phenyl-5(4*H*)-oxazolones **1a–1j** used in this work.

(*Z*)-Oxazolones **1a–1j** (Figure 2), which contain electron-releasing and electron-withdrawing substituents at different positions of the aromatic ring, were selected to ensure the widest reaction scope. These compounds were prepared as reported in the literature.^24^

**Figure 3:**
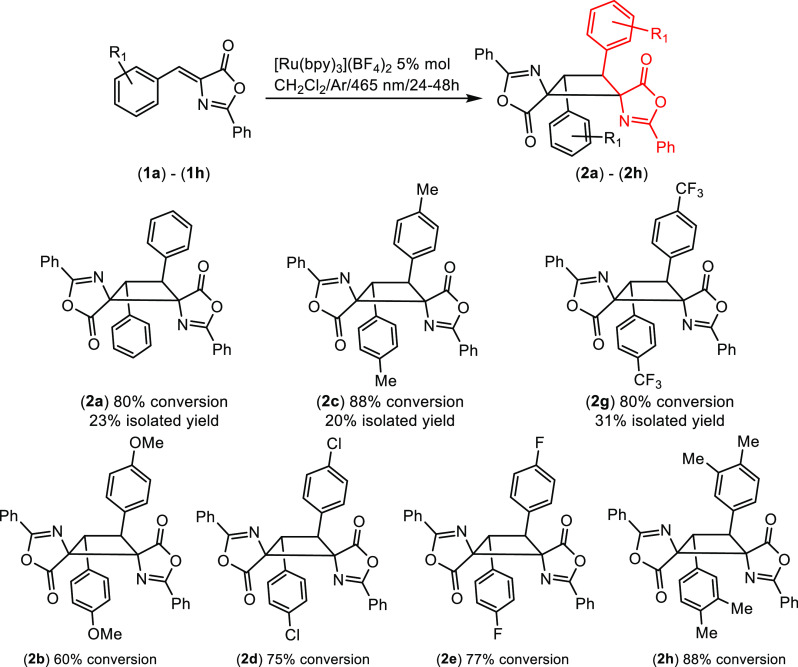
Synthesis of cyclobutane-bis(oxazolone)s **2**.

Irradiation of (*Z*)-oxazolone **1c** with blue light (465 nm) in a deoxygenated CH_2_Cl_2_ solution and in the presence of catalytic amounts of [Ru(bpy)_3_](BF_4_)_2_ (5% mole ratio; bpy = 2,2′-bipyridine) led to selective formation of cyclobutane **2c**, as shown in Figure 3. The reaction takes place with remarkable selectivity as the photocycloaddition could theoretically afford ≤11 different stereoisomers (SIs), whereas cyclobutane **2c** was obtained as a single isomer. To optimize the reaction conditions, deoxygenated solutions of oxazolone (*Z*)-**1c** and [Ru(bpy)_3_](BF_4_)_2_ (5% mole ratio) in CD_2_Cl_2_ under an argon atmosphere were irradiated with blue light (465 nm), and the progress of the reaction was monitored by *in situ* illumination ^1^H NMR spectroscopy (Figure S1). The time for maximal conversion was set as the reaction time (usually 24 h). A short screening of deoxygenated solvents (CD_2_Cl_2_, CD_3_CN, acetone-*d*_6_, CDCl_3_, and DMSO-*d*_6_) showed that the best conversions were achieved in CD_2_Cl_2_. With respect to the amount of the Ru complex, similar conversions were obtained using 5 and 10 mol % (75% vs 76%), whereas the conversion decreased when 1 mol % was used (70%). Under the optimized reaction conditions, **2c** was isolated as a white solid in 20% yield. The Ir(III) photocatalyst [Ir(dF(CF_3_)ppy)_2_(dtbbpy)](PF_6_) was also examined, and it gave best conversions of **1c** in CD_2_Cl_2_, almost reaching 90%. However, three different isomers of cyclobutane **2c** were detected after irradiation; therefore, the selectivity of this process is worse using [Ir(dF(CF_3_)ppy)_2_(dtbbpy)](PF_6_) than using [Ru(bpy)_3_](BF_4_)_2_. Due to the excellent stereoselectivity shown by the Ru species, we focused our research on this photocatalyst.

Compound **2c** is stable in the solid state at room temperature, whereas in solution, it has to be stored at a low temperature (−15 °C) to prevent it from undergoing a thermal retro-[2+2] reaction in solution at room temperature to form free (*Z*)-**1c**. This low stability is probably the reason for the low isolated yield of pure **2c** even though high conversions were observed. Despite this, we attempted to determine the scope of this photocycloaddition and obtained the results shown in Figure 3. Although high conversions were observed by NMR spectroscopy in most cases, pure cyclobutanes **2** could be obtained for only **2a** (23% isolated yield) and **2c** (20% isolated yield), with mixtures of **2** with oxazolones **1** being obtained for **2b**, **2d**, **2e**, **2g**, and **2h**. In the case of **1f**, a maximum conversion of 30% of **2f** was achieved, although the subsequent fast thermal retro-[2+2] reaction precluded its characterization. Given these difficulties, isolation of the corresponding **2i** and **2j** was not attempted in the case of **1i** and **1j**, respectively. Nevertheless, **1f**, **1i**, and **1j** were successfully converted into the corresponding truxinic esters (*vide infra*).

**Figure 4:**
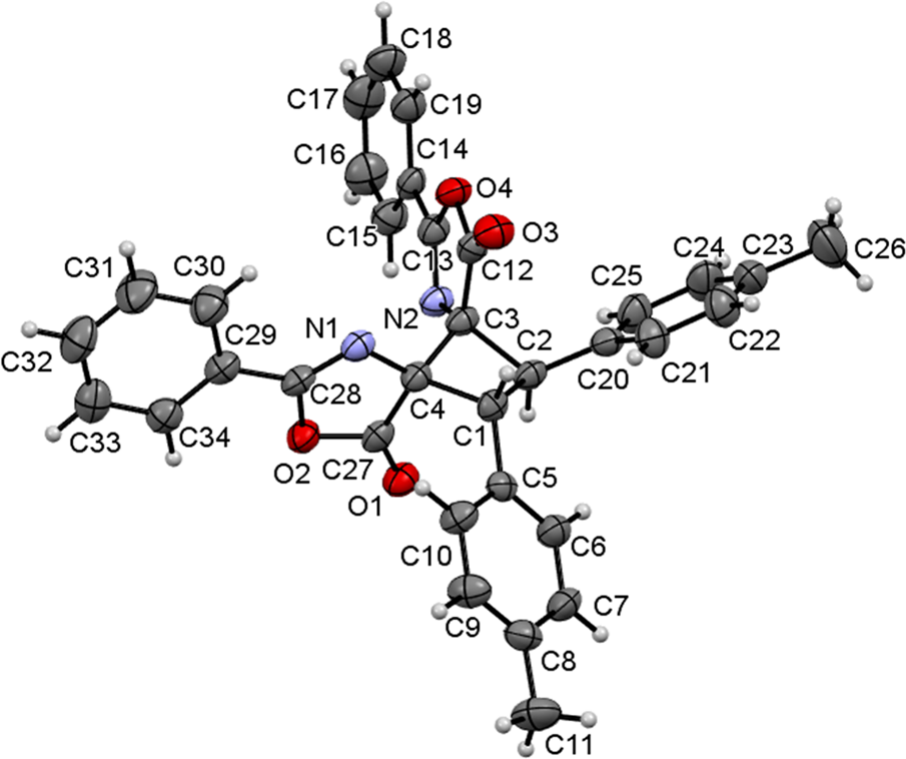
Molecular drawing of **2c**. Ellipsoids are drawn at the 50% probability level.

The full characterization of cyclobutanes **2a–2h** was carried out by mass spectrometry, NMR spectroscopy, and X-ray diffraction methods. Although the [2+2] photocycloaddition of oxazolones **1** could give up to 11 isomers (SIs), the ^1^H and ^13^C NMR spectra (and ^19^F NMR for **2e** and **2g**) showed signals due to the presence of a cyclobutane of high symmetry as a single isomer in all cases. We discarded formation of the four isomers resulting from coupling of one (*Z*)-oxazolone with one (*E*)-oxazolone for symmetry reasons,^22^ as well as the ε- and α-isomers, which were characterized by us very recently.^18^ As such, five possible isomers [peri, β, δ, μ, and ω (see the Supporting Information for detailed structures)] were still consistent with the NMR data and thus required additional methods to elucidate the structure. Information provided by the DP4 method (see the Supporting Information for details about DP4) suggests that the isomer obtained is the μ-isomer, which contains two (*E*)-oxazolones coupled in a 1,2-head-to-head *anti* form, because this has the highest probability (86.2%).^25^ However, this value is still far from the values deemed to be acceptable to consider a given structure to be unambiguously determined (typically >98%).^25^ Fortunately, determination of the crystal structure of **2c** provided definitive evidence of the formation of the μ-isomer, as shown in Figure 4.

The structure shows the cyclobutane C(1)–C(2)–C(3)–C(4) core formed by the head-to-head *anti* photocycloaddition of two (*E*)-oxazolones, thus confirming the prediction of the DP4 analysis. The cyclobutane has two *trans* C_6_H_4_Me rings on adjacent carbons [C(1) and C(2)], which point toward opposite sides of the molecular plane, and two oxazolones on the other two adjacent carbons [C(3) and C(4)], also in a *trans* arrangement. However, the arrangement of the C_6_H_4_Me rings with respect to the C=O groups of the oxazolones on adjacent carbons [for instance, C(1) and C(4)] is *cis*, meaning that the configuration of the molecule is 1,2-*trans*-2,3-*trans*-3,4-*trans*. The cyclobutane is not planar, as deduced from the dihedral angles [C(1)–C(2)–C(3)–C(4) angle of −23.2(2)° and C(2)–C(3)–C(4)–C(1) angle of 22.8(2)°]. These values are larger than those found in other cyclobutanes containing heterocycles as substituents at positions 1 and 2 [range of 16.93(2)–18.56(2)°].^26^ The C–C bond distances in the cyclobutane ring [1.545(4), 1.572(4), 1.564(4), and 1.557(4) Å] are identical within experimental error and are in the usual range reported for C–C bonds.^27^ The values found for other internal parameters are also identical, within experimental error, to those found in the literature.^27^

### Synthesis and Characterization of Methyl Esters of 1,2-Diaminotruxinic Acids **3**

**Figure 5:**
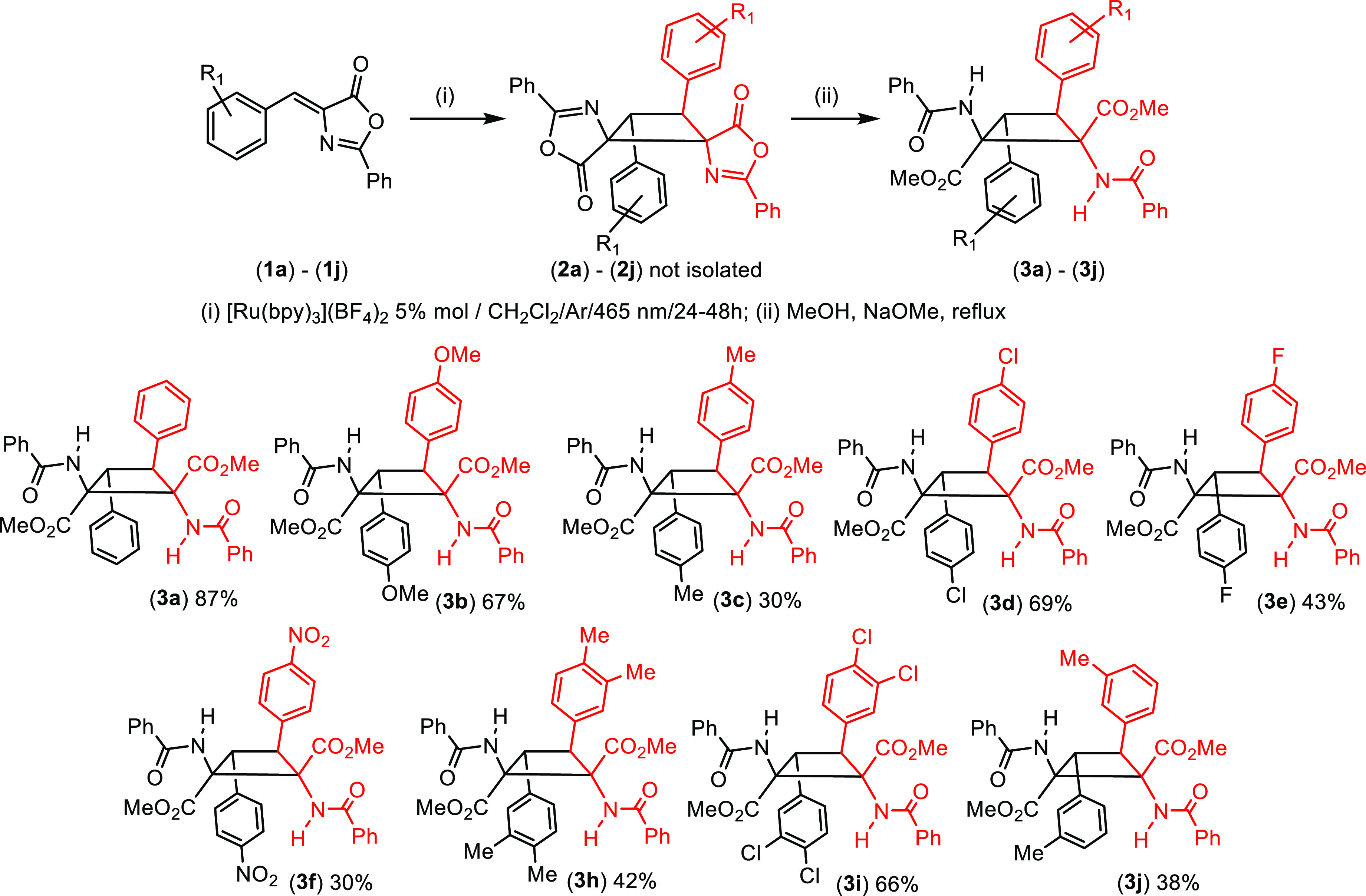
Synthesis and scope of the 1,2-diaminotruxinic methyl ester derivatives **3a–3j** prepared.

After formation of the cyclobutane ring in **2**, the base-catalyzed ring-opening reaction of oxazolones in alcoholic medium is the next step in the synthesis of 1,2-diaminotruxinic derivatives.^28^ Considering the low stability of **2** in solution, and to minimize the retro-[2+2] reaction, we carried out the synthesis of 1,2-diaminotruxinic esters **3** from oxazolones **1** in a straightforward one-pot, two-step method, without isolating cyclobutanes **2** (Figure 5).

According to this method, oxazolones **1** were irradiated with blue light (465 nm) under optimized reaction conditions. Once the maximum conversion of **1** into **2** was reached, the solvent was removed to dryness under vacuum while the irradiation was maintained. The solid residue was then treated, under air, with MeOH and a catalytic amount of NaOMe, and the resulting suspension heated to reflux for 30–45 min in the dark in an oil bath. After evaporation of the methanol, analysis of the crude residue showed formation of 1,2-diaminotruxinic methyl esters **3** as single isomers (Figure 5). In some cases, the presence of small amounts of the corresponding dehydrophenylalanine was also observed. Methyl esters **3** were purified by column chromatography or crystallization and were isolated as air- and moisture-stable white solids that are even stable against the retro-[2+2] reaction in the solid state and in solution.

This one-pot, two-step method represents a better synthetic pathway for the production of pure 1,2-diaminotruxinic esters **3** from **1** than from **2**. (i) The yields of **3**, without isolating **2**, are better than those obtained from isolated **2**. (ii) The synthesis of **3** is simpler, and the purification of **3** is much easier than that of **2** (only **3i** showed a small impurity reluctant to be removed). (iii) Esters **3** can be obtained with a wider variety of electron-donating [OMe (**3b**), Me (**3c** and **3j**), or 3,4-Me_2_ (**3h**)] or electron-attracting substituents [Cl (**3d**), F (**3e**), NO_2_ (**3f**), or 3,4-Cl_2_ (**3i**)]. Only 4-CF_3_ derivative **3g** could not be obtained in pure form due to decomposition. The reaction shows a dependence on the position of the substituents at the aromatic ring, probably as a result of steric interactions. In general, *para* substituents allow the corresponding cyclobutanes **3** to be obtained in moderate to good yields, *meta* substituents afford lower yields, and *ortho* substituents (examples not shown) hinder the reaction, with no cyclobutanes being formed. Moreover, the method reported here shows clear advantages with respect to other reported procedures.^22^ Thus, the stereochemistry of the resulting 1,2-diaminotruxinic esters **3** in solution and in the solid state is clearly defined and has been unambiguously assigned as the μ-isomer by NMR spectroscopic and X-ray diffraction methods (see below), thus meaning that this method using Ru(II) as a photocatalyst provides a different isomer and extends the list of isomers that can be formed via photocatalyzed cycloaddition. This fact opens the door to the tailored synthesis of specific isomers (μ) of biologically relevant 1,2-diaminotruxinic bis-amino acids simply by controlling the reaction conditions (solvent and photocatalyst). Second, this synthesis is simpler than in previous cases as no Lewis acids or other additives are required. Third, the yields are, in general, higher than in previous reports.^22^

**Figure 6:**
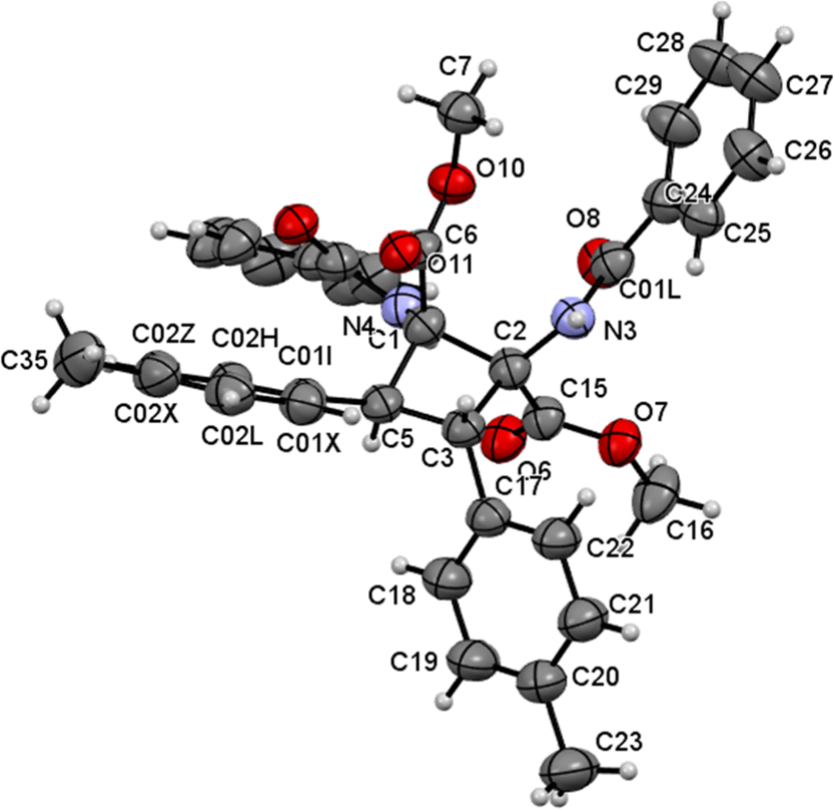
Molecular structure of **3c**. Ellipsoids are drawn at the 50% probability level.

**Figure 7:**
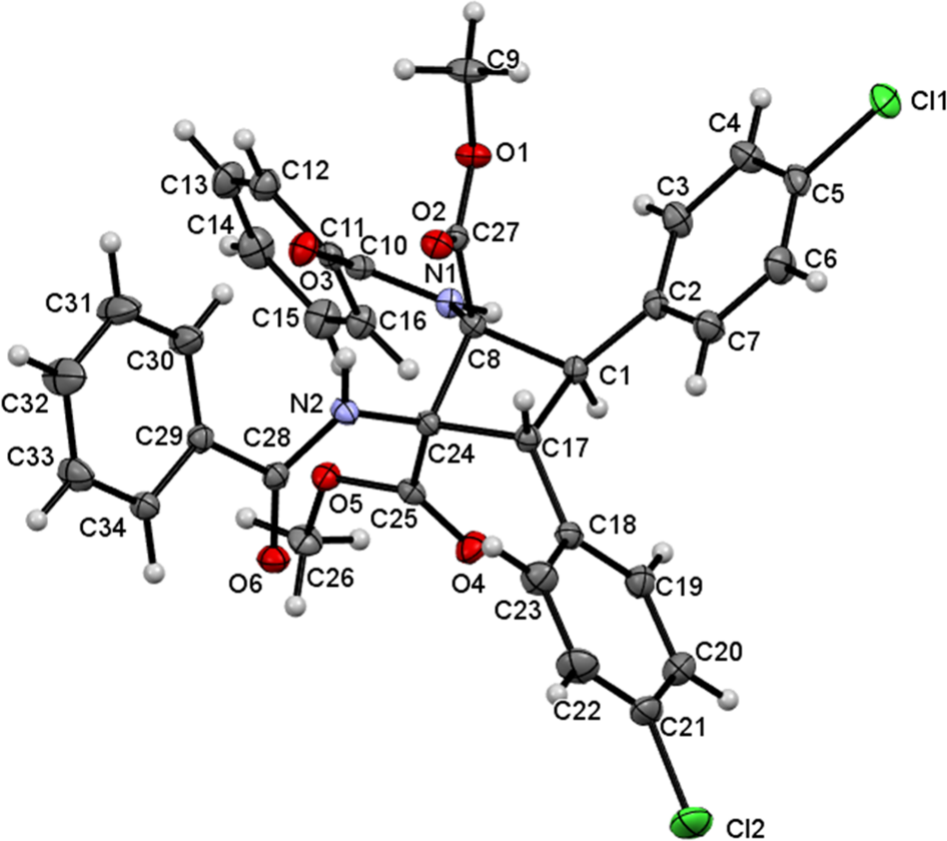
Molecular structure of **3d**. Ellipsoids are drawn at the 50% probability level.

Full characterization of 1,2-diaminotruxinics **3a–3j** showed that the isomer obtained is the expected μ-isomer; in other words, the ring-opening reaction of the oxazolone takes place with complete retention of the configuration at the cyclobutane moiety. This was confirmed by determining the crystal structure of compounds **3c** and **3d**. Figure 6 shows the molecular structure of **3c**, while Figure 7 shows that of compound **3d**.

The X-ray crystal structures show that cyclobutanes **3c** and **3d** have two *p*-tolyl and two *p*-ClC_6_H_4_ rings, respectively, in a *trans* arrangement on adjacent carbons [C(3) and C(5) for **3c** and C(1) and C(17) for **3d**] and two methyl esters and two N(H)C(O)Ph moieties at the other two adjacent carbons [C(1) and C(2) for **3c** and C(8) and C(24) for **3d**], as a result of the oxazolone ring-opening reaction. The relative arrangements of the esters and amido groups with respect to the *p*-tolyl or *p*-ClC_6_H_4_ rings in each case are the same as in the cyclobutane-bis(oxazolone) precursors **2**. Other than the spatial arrangement, analysis of the internal parameters (bond distances and angles) did not show any unusual features, with all values being in the expected ranges.^27^

### *Ex Situ* On-Flow Irradiation of (*Z*)-4-Aryliden-5(4*H*)-oxazolones **1** in the Presence of [Ru(bpy)_3_](BF_4_)_2_ as a Triplet Photocatalyst

The use of microreactors has demonstrated its relevance when chemical reactions have been performed in comparison with the batch methodology.^17b,29^ Thus, microreactors increase the surface:volume ratio, which leads to more efficient heat and mass transfer, improved diffusion, and good control of the reaction temperature. Microreactors also maximize the selectivity of the reaction, avoid undesired reactions, and offer the possibility of continuous-flow operation. This means that the reaction conditions can be rapidly optimized, and efficient mixing of the reactants decreases the reaction time and increases the reaction yield. With regard to photochemical applications, the small dimensions of the microreactors ensure efficient light irradiation of the reaction, thus increasing the selectivity, shortening the reaction time, accelerating reaction optimization, and allowing the catalyst loading to be reduced.^29^

With the aim of further improving the performance of the [2+2] photocycloaddition of (*Z*)-oxazolones **1** to give cyclobutanes **2**, we followed the procedure described in the Experimental Section using a LED-based illumination device. The use of very small volumes and optimum mixing of the reagents notably reduced the level of bleaching of the photocatalyst due to the presence of oxygen in the samples; therefore, these experiments were carried out with no special precautions to ensure the exclusion of oxygen. We also optimized the residence time, varying it from 20 to 40 min. Oxazolone (*Z*)-**1b** was chosen as a test substrate because it was one of the cases in which it was not possible to isolate pure cyclobutane **2b** due to moderate conversion and fast retro-[2+2] reaction. The results are summarized in Table 1.

**Table 1:** Optimization of the Residence Time for (*Z*)-**1b**^a^

entry	τ (min)	first reaction (%)	τ (min)	second reaction (%)	τ (min)	third reaction (%)
1	20	47	20	69	–	–
2	30	45	30	75	30	77

a With 120 mM **1b**, 5 mol % [Ru(bpy)_3_](BF_4_)_2_, CD_2_Cl_2_, blue light (465 nm), 25 °C.

For 20 min of residence time τ (entry 1), a conversion of 47% was obtained in the first reaction. After a second consecutive reaction for 20 min, the conversion increased to 69%. Longer residence times were tested, but the conversions obtained were similar (45%, entry 2) after 30 min. However, after two consecutive reactions of 30 min each, a conversion of 75% was achieved. No further improvements were possible in a third reaction (77%). We also performed the reaction with a residence time of 40 min but obtained conversions similar to those obtained after 30 min. As such, the best option for carrying out this reaction was two consecutive reactions with 5 mol % photocatalyst and a residence time of 30 min for each reaction. Despite the fact that the use of small volumes notably reduces the amount of oxygen that would cause photobleaching of the catalyst, we observed some photobleaching after the first cycle of 30 min. This is probably due to excessive irradiation of the catalyst as microchannels enable more efficient light penetration. As such, the photobleached catalyst was eliminated from the reaction medium (using CHCl_3_ and filtration), and fresh catalyst was added to carry out the second cycle. For all cases studied, the reaction conversion increases notably in the second cycle after the catalyst is refreshed (see page S54 of the Supporting Information). After optimizing the reaction conditions, we analyzed the scope of this reaction with oxazolones **1a–1g**, **1i**, and **1j**; the results are listed in Table 2.

**Table 2:** Comparison of Conversions (percent) of Oxazolones (*Z*)-**1** to Give Cyclobutane-bis(oxazolone)s **2** Obtained Using Batch and Continuous-Flow Methodologies^a^

oxazolone	[**1**] (mM)^a^	flow (O_2_, 1 h)	batch (O_2_, 24 h)	batch (24 h)^b^
**1a**	120	75	60	80
**1b**	120	75	65	70
**1c**	150	65	75	88
**1d**	120	85	51	75
**1e**	120	80	64	77
**1f**	25	70	53 (48 h)	60 (48 h)
**1g**	120	78	63	80
**1i**	120	59	62	80
**1j**	150	79	75	81

a The concentration is 120 mM by default, except for those of **1c** and **1j** (150 mM) and poorly soluble **1f** (25 mM).

b In the absence of O_2_.

The selectivity of the reaction remains unchanged, and μ-truxinic derivatives **2a–2j** were obtained stereo- and regioselectively. A comparison of the reaction conversions in continuous flow with those obtained in batch mode in the presence of oxygen shows a considerable increase in conversion in flow reactors (except for that of **1c**) of ≤30% in the best case [4-Cl (**2d**)], and a remarkable decrease in the reaction times from 24 h (even 48 h for **1f**) to 60 min, thus demonstrating the efficient light penetration when photochemical reactions are performed in microreactors. Moreover, the conversions obtained on flow are roughly comparable to those obtained in batch mode in the absence of oxygen, although the acceleration observed when working in continuous-flow mode (1 h vs 24 h in batch mode) tips the scale in favor of the flow system. As expected, the comparison of the space time yields (STYs) of the reactions performed under flow conditions, in the presence of O_2_, or in batch reactors (even in the absence of O_2_) shows the better efficiency of the former (see Table S1).

### Mechanistic Considerations

**Figure 8:**
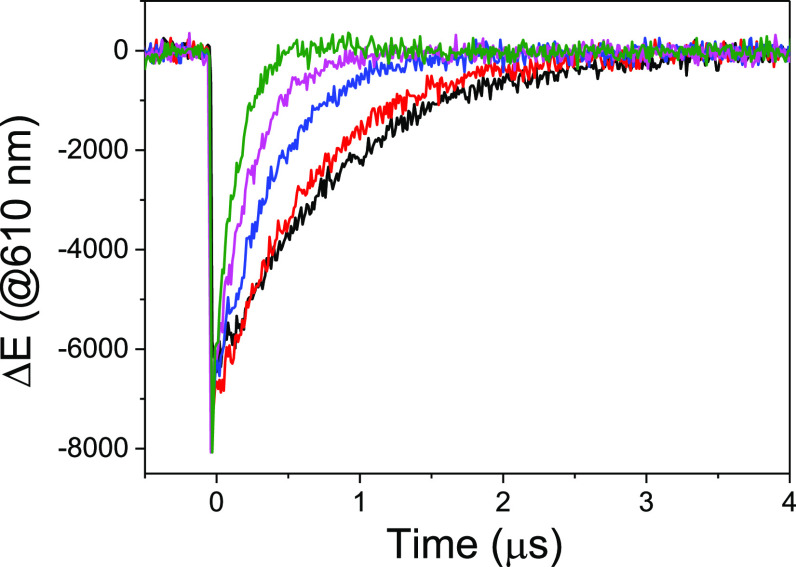
Decay traces recorded at 610 nm for Ru(bpy)_3_^2+^ (in deaerated CH_2_Cl_2_) upon addition of different amounts of (*E*)-**1c**: 0 (black), 1.4 × 10^–4^ M (red), 4.2 × 10^–4^ M (blue), 8.4 × 10^–4^ M (pink), and 1.7 × 10^–3^ M (green), obtained after LFP excitation (532 nm).

Initially, the absorption spectra of oxazolones and the complex [Ru(bpy)_3_](BF_4_)_2_ (SIs) were recorded to postulate a plausible Ru-photocatalyzed mechanism for this selective photochemical transformation. All oxazolones studied showed a broad absorption centered at around 365–375 nm, with a well-defined shoulder at around 385–395 nm and another less-defined one in the region of 350–360 nm, in good agreement with the absorption spectra reported previously for (*Z*)- and (*E*)-oxazolones.^16b,30^ The [Ru(bpy)_3_](BF_4_)_2_ complex, in turn, exhibits a strong absorption in the visible region, with a peak centered at 460 nm corresponding to the ^1^MLCT state.^31^ As such, the incident light (centered at 465 nm) is selectively absorbed by the ruthenium complex, and therefore, oxazolone transformations must be initiated by the absorption of visible light by Ru(bpy)_3_^2+^, which involves initial formation of the ^1^MLCT state followed by a very fast ISC (intersystem crossing) to the ^3^MLCT state. Hence, as described in the literature for other reactions photocatalyzed by Ru(bpy)_3_^2+^,^31−33^ several processes could occur between the triplet excited state of the Ru(bpy)_3_^2+^ and oxazolones: an electron transfer to generate the corresponding radical anions and radical cations (see eqs 1 and 2) or an energy transfer to populate the triplet of the non-absorbing oxazolones (eq 3). The Gibbs free energies associated with eqs 1 and 2 and *ΔH*_ET_ to eq 3, which are given by eqs 4–6, respectively, were determined to evaluate the thermodynamic feasibility of these three options. Thus, photoredox processes, as described by eqs 1 and 2, were found to be thermodynamically disfavored (Δ*G*_et_^°^ = 0.3 and 0.8 eV, respectively) using the reported values for Ru(bpy)_3_^3+^/Ru(bpy)_3_^2+^ and Ru(bpy)_3_^2+^/Ru(bpy)_3_^+^ (1.29 and −1.33 V vs SCE, respectively)^1d,31b−31d^ and the redox potentials measured for oxazolone (*Z*)-**1c** in a CH_2_Cl_2_ solution [−1.37 and 1.81 V vs SCE for oxazolone/oxazolone^•–^ and oxazolone^•+^/oxazolone, respectively (details in the Supporting Information)]. However, the triplet–triplet energy transfer (eq 3) was found to be slightly exothermic (Δ*H*_ET_^°^ = −0.5 eV) using the energy for ^3^[Ru(bpy)_3_^2+^]* (*E*_T_ = 2.36 eV) and the reported value for ^3^oxazolone* (*E*_T_ = 1.86 eV).^34^
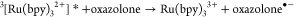
1
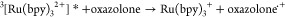
2
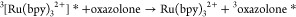
3
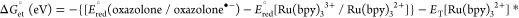
4
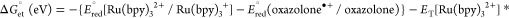
5
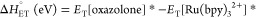
6The dynamics of ^3^[Ru(bpy)_3_^2+^]* in the presence of oxazolone (*Z*)-**1c**, as a typical example, were then investigated using steady-state spectroscopic techniques. Thus, an efficient quenching of the phosphorescence of ^3^[Ru(bpy)_3_^2+^]* was observed with increasing concentrations of (*Z*)-**1c** (data not shown). In an attempt to provide more information about the intermediates involved in the Ru-photocatalyzed stereoselective cyclization of oxazolones, laser flash photolysis (LFP) experiments were carried out using Ru(bpy)_3_^2+^ in the presence and absence of typical oxazolones such as oxazolone (*E*)-**1c** or oxazolone (*Z*)-**1c**. Initially, the transient absorption spectrum recorded upon laser flash excitation of Ru(bpy)_3_^2+^ at 532 nm shows a transient absorption band centered at 360 nm, a ground-state bleaching at around 450 nm, and a stimulated emission centered at 620 nm. All three bands exhibit the same kinetic behavior, thus indicating that they correspond to ^3^[Ru(bpy)_3_^2+^]*. Moreover, as expected for a triplet excited state, the lifetime decreases from 620 ns under N_2_ to 340 ns under air due to the quenching effect of molecular oxygen. Indeed, a quenching rate constant of 7 × 10^9^ M^–1^ s^–1^ was determined for this reaction process. The study was then continued by adding increasing amounts of (*E*)-**1c** and (*Z*)-**1c** to anaerobic dichloromethane solutions of Ru(bpy)_3_^2+^. The results revealed an efficient quenching of ^3^[Ru(bpy)_3_^2+^]* in the presence of both isomers [see Figure 8 for (*E*)-**1c** as an example]. The linear fittings based on the Stern–Volmer relationships gave a quenching constant of 2.9 × 10^10^ M^–1^ s^–1^, which was found to be identical for the two isomers and diffusion-controlled, as expected from the Δ*E* for the initial and final triplet excited states (−0.5 eV, eq 6).^35^

**Figure 9:**
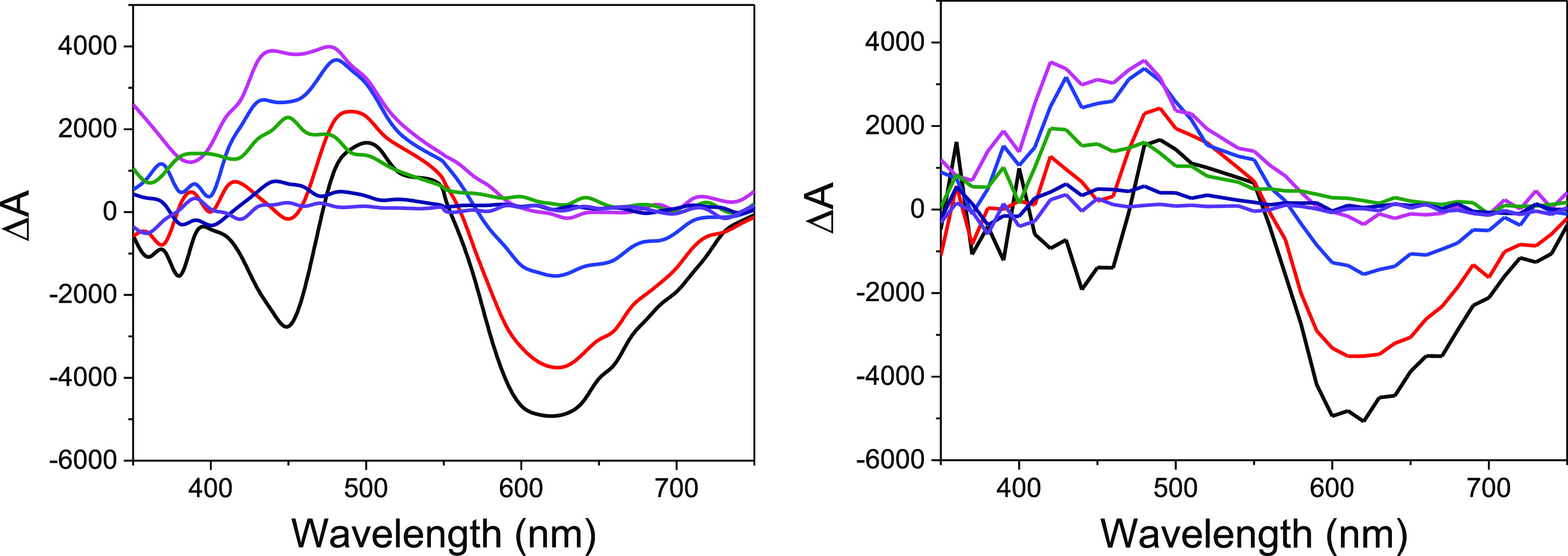
Transient absorption spectra for deaerated CH_2_Cl_2_ solutions of Ru(bpy)_3_^2+^ in the presence of (*E*)-**1c** (left) or (*Z*)-**1c** (right) (2.5 × 10^–3^ M) recorded at different times after the laser pulse (λ_exc_ = 532 nm): 10 ns (black), 42 ns (red), 124 ns (blue), 224 ns (pink), 550 ns (green), 924 ns (dark blue), and 1376 ns (violet).

**Figure 10:**
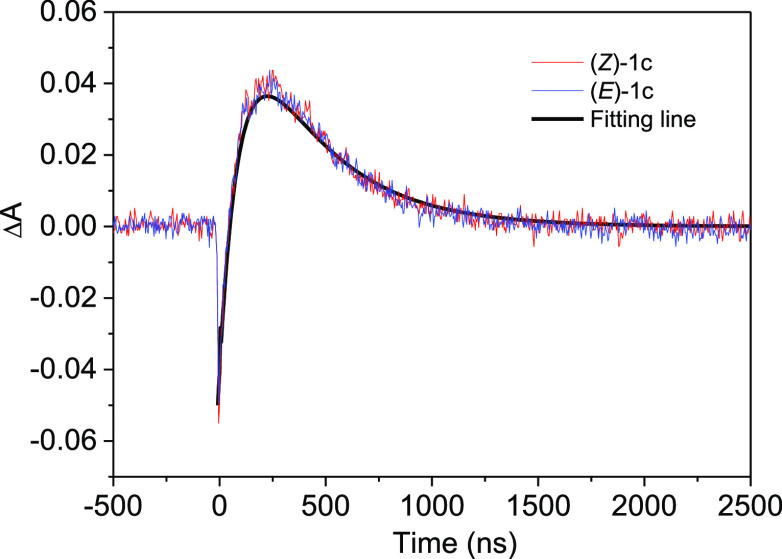
Transient absorption traces recorded at 470 nm upon LFP excitation (532 nm) of Ru(bpy)_3_^2+^ (in deaerated CH_2_Cl_2_) in the presence of (*Z*)-**1c** (red) or (*E*)-**1c** (blue) (2.5 × 10^–3^ M) and a fitting line {black; Δ*A* = −[*A*_1_ × exp(−*k*_1_*t*)] – [*A*_2_ × exp (−*k*_1_*t*)] + [*A*_2_ × exp(−*k*_2_*t*)]}. *A*_1_ = 0.05 is the value of the initial bleaching. *k*_1_ = 1 × 10^7^ s^–1^ is the deactivation rate constant of the Ru photocatalyst triplet excited state obtained from traces at 650 nm. *A*_2_ = 0.95 and *k*_2_ = 2.8 × 10^6^ s^–1^ are the maximum absorption of the diradical triplet excited state and its deactivation rate constant determined from fitting of the decay segment of the traces obtained at 470 nm, respectively.

Furthermore, addition of the oxazolones to deaerated solutions of Ru(bpy)_3_^2+^ in CH_2_Cl_2_ also revealed the generation of a new transient species. Thus, as shown in Figure 9, while the transient absorption spectrum of ^3^[Ru(bpy)_3_^2+^]* disappears with time, a new intermediate showing an absorption band with two maxima at around 430 and 480 nm is generated concomitantly and then disappears after several hundreds of nanoseconds. Interestingly, the new transient species detected for the two isomers were found to exhibit a similar absorption spectrum and the same behavior. Thus, identical decays were observed for deaerated solutions of Ru(bpy)_3_^2+^ in the presence of (*E*)-**1c** or (*Z*)-**1c** at 470 nm, as shown in Figure 10. Moreover, given the unambiguous overlapping of the two decays of Figure 10, one can conclude that generation of the new intermediate has an identical quantum yield for both isomers. The lifetime of this identical intermediate, which was determined from the exponential fitting of both decays, was around 368 ns under N_2_. The fact that both transient species were also efficiently quenched by O_2_ (a quenching rate constant of 1.2 × 10^10^ M^–1^ s^–1^ was determined for both) is consistent with a triplet excited state for the common intermediate arising from the two oxazolones. Moreover, deconvolution of transient absorption traces detected at 470 nm revealed that the recovery of Ru photocatalyst bleaching follows a first-order reaction with a rate constant value similar to that determined at 650 nm for its triplet excited state (∼1 × 10^7^ s^–1^). This fact, as it has been well documented,^36a^ proves that energy transfer is occurring from the Ru photocatalyst to oxazolones. Thus, as shown in Figure 10, the transient absorption traces obtained at 470 nm upon LFP excitation of Ru(bpy)_3_^2+^ in the presence of oxazolone (*E*)-**1c** (blue) or (*Z*)-**1c** (red) (2.5 × 10^–3^ M) were coincident with a fitting line determined from the summatory of three first-order reactions using the same rate constant for the recovery of the Ru photocatalyst bleaching as for the generation of the 1,4-diradical triplet excited state (∼1 × 10^7^ s^–1^) and a value of 2.8 × 10^6^ s^–1^ for the diradical triplet decay.

In conclusion, the LFP experiments provided unambiguous evidence for the triplet excited-state nature of the transient absorption species formed as a result of efficient energy transfer from ^3^[Ru(bpy)_3_^2+^]* to (*E*)-**1c** or (*Z*)-**1c**. The resulting oxazolone triplet excited state can be denoted as ^3^(*E/Z*)-1c* and is exactly the same species regardless of the starting isomer. Not surprisingly, ^3^(*E/Z*)-1c* was not generated when control solutions containing (*E*)-**1c** or (*Z*)-**1c** were excited at 532 nm in the absence of Ru(bpy)_3_^2+^. Even more interestingly, ^3^(*E/Z*)-1c* was also not detected when laser excitation at 355 nm, where oxazolones show an intense absorption, was used. As such, ^3^(*E/Z*)-1c* is not involved in the direct photolysis of oxazolones.

If the reaction of the generated triplet excited-state ^3^(*E/Z*)-1* with an oxazolone (Z)-**1** affords the cyclobutane derivative (μ-*E*,*E* isomer)-**2** in all cases studied, then the reaction starting from oxazolone (*E*)-**1** should give a different isomer even though the same triplet excited state is involved in the reaction. To clarify this scenario, the composition of a solution initially containing either oxazolone (*Z*)-**1c** or (*E*)-**1c** and Ru(bpy)_3_^2+^ was examined by ^1^H NMR spectroscopy during the very early stages of the photocatalyzed reaction (see details in the Supporting Information). Thus, analysis of the ^1^H NMR spectrum of the mixture (*Z*)-**1c** and Ru(bpy)_3_^2+^, after irradiation for 10 min, showed signals due to the starting materials and a very small amount of cyclobutane **2c**. Interestingly, when the solution initially containing (*E*)-**1c** and Ru(bpy)_3_^2+^ was irradiated, only signals due to (*Z*)-**1c**, the Ru species, and a small amount of cyclobutane **2c** were detected. Hence, the results of the two experiments are completely coincident and provide further support for a fast and quantitative isomerization of the (*E*)-isomer, via the photosensitized ^3^(*E/Z*)-1c*, followed by back intersystem crossing to the more stable (*Z*)-isomer, during the very early stages of the reaction.^18^ Bearing in mind that formation of dimers takes 24 h to reach completion, we can safely assume that (*E*)-**1c** is isomerized to (*Z*)-**1c** before reacting with ^3^(*E/Z*)-1c*. The photosensitized isomerization of oxazolones as molecular switches could be occurring as described in the literature for other compounds, such as azobenzene or stilbene.^33,36b,37^

**Figure 11:**
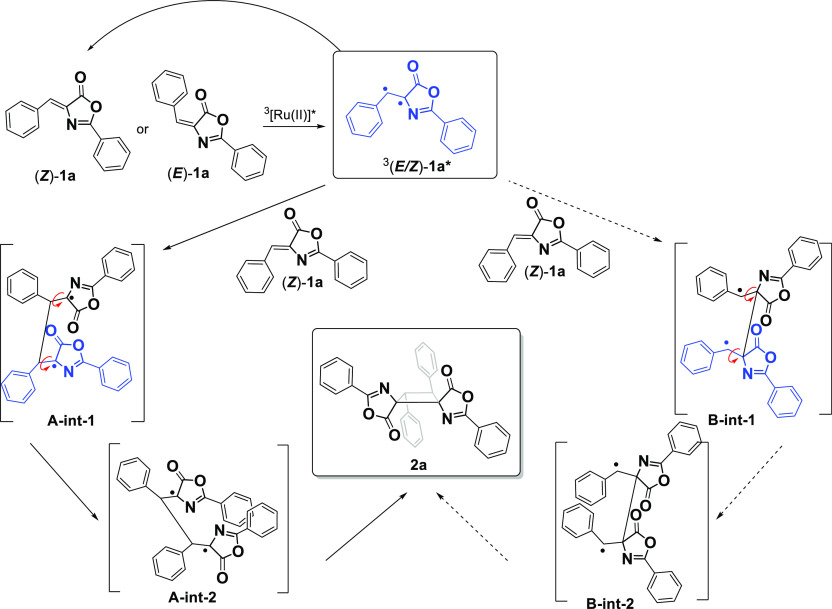
Potential mechanisms for explaining the photocatalyzed dimerization of (*Z*)-**1a** or (*E*)-**1a** with (*Z*)-**1a** giving rise to **2a**.

On the basis of these results, DFT calculations were performed on different reaction pathways to postulate a plausible mechanism to explain formation of the μ-*E*,*E*-isomer from the reaction between ^3^(*E/Z*)-oxazolones* and a ground state of (*Z*)-oxazolones (Figure 11 and Table 3).

**Table 3:** Absolute and Relative DFT Energies for the Minimum Energy Conformations of the Intermediates Shown in Figure 11, Using B3LYP-D3 and the cc-pVTZ Basis Set^a^

method/intermediate/solvent	absolute energy (Ha)	relative energy (kJ/mol)
B3LYP-D3/**A-int-1**/dichloromethane	–1643.687489	0.0
B3LYP-D3/**A-int-2**/dichloromethane	–1643.684299	8.4
B3LYP-D3/**B-int-1**/dichloromethane	–1643.633378	142.1
B3LYP-D3/**B-int-2**/dichloromethane	–1643.627527	157.4
B3LYP-D3/**A-int-1**/no solvent	–1643.673487	0.0
B3LYP-D3/**A-int-2**/no solvent	–1643.670443	8.0
B3LYP-D3/**B-int-1**/no solvent	–1643.620540	139.0
B3LYP-D3/**B-int-2**/no solvent	–1643.614788	154.1

a The calculations include the absence of solvent as well as the presence of dichloromethane as the solvent (using the SCRF/PCM option in Gaussian09).^39^ Results with other functionals are listed in Tables S2 and S3.

### Computational Results

All geometries were optimized using first-principles methods based on DFT, using four different functionals (B3LYP-D3,^38a^ PBE-D3,^38b^ M062X-D3,^38c^ and wB97XD^38d^) and two basis sets (Def2-TZVP^38e^ and cc-pVTZ^38f^). All of these functionals contain, or have been completed (using Grimme’s D3 approach^38g^), with corrections for dispersion terms. The first-principles DFT-optimized geometries showed similar features irrespective of the functional and basis set employed. Some optimized geometries (B3LYP-D3/cc-pVTZ) for selected intermediates are shown in Figures S3 and S4. With regard to the energies, all of the energy values for intermediates (**A** and **B**)**-int-**(**1** and **2**) were determined using four functionals (B3LYP-D3, M062X-D3, PBE1-D3, and WB97XD) and two basis sets (Def2-TZVP and cc-pVTZ). Negligible differences with respect to the use of different basis sets (cc-pVTZ and Def2-TZVP) were obtained. Overall, this demonstrates that all methods give roughly the results shown in Table 3. Finally, the results obtained do not depend on the effect of the solvent: a comparison between no solvent and CH_2_Cl_2_ gave similar results.

DFT calculations were performed using the structurally simplest oxazolones, (*Z*)- and (*E*)-**1a**, to shed light on the interaction between the photosensitized triplet excited state of one oxazolone and the ground state of another, which gives rise to a truxinic acid (μ-*E,E*-isomer), in CH_2_Cl_2_. Although different scenarios can be envisaged starting from (*E*)-**1a** or (*Z*)-**1a**, only routes that could give rise to the isomer observed (**2a**) were considered (Figure 11). Upon formation of the common triplet ^3^(*E/Z*)-1a*, two different options were investigated to explain the subsequent interaction with the (*Z*)-**1a** isomer. Initially, ^3^(*E/Z*)-1a* interacts with (*Z*)-**1a**, forming a new C–C bond between the two units via the benzylic CH positions (route A, left part of Figure 11) and giving rise to the 1,4-biradical intermediate (**A-int-1**). Next, as a result of two rotations, **A-int-1** is transformed into **A-int-2**, before back intersystem crossing to the singlet biradical and final formation of cyclobutane **2a**. Alternatively, ^3^(*E/Z*)-1a* interacts with (*Z*)-**1a** forming a new bond between the two quaternary C atoms, giving rise to a different 1,4-biradical (**B-int-1**, right part of Figure 11). Subsequent rotation of two bonds transforms **B-int-1** into **B-int-2**, which eventually gives rise to **2a**.

The first conclusion is that route B can be easily excluded because both **B-int-1** and **B-int-2** gave very high energies [139.0 and 154.1 kJ/mol (Table 3)] with respect to the most stable intermediate (**A-int-1**). With regard to route A, the energies of the two intermediates differ by only 8.0 kJ/mol using B3LYP-D3 [8.9 kJ/mol using M062X-D3, 9.5 kJ/mol using PBE1PBE, and 9.6 kJ/mol using WB97XD (Table S2)], which is easily reached at room temperature. In conclusion, DFT calculations show the feasibility of the coupling between oxazolone (*Z*)-**1a** and the triplet diradical excited state ^3^(*E/Z*)-1a* via the formation of a CH–CH bond between the benzylic positions, and that further rotation of the 2-phenyloxazolone fragment around the C–C bond is allowed and almost barrierless before final C–C coupling, thus resulting in selective formation of the μ-(*E,E*)-isomer.

**Figure 12:**
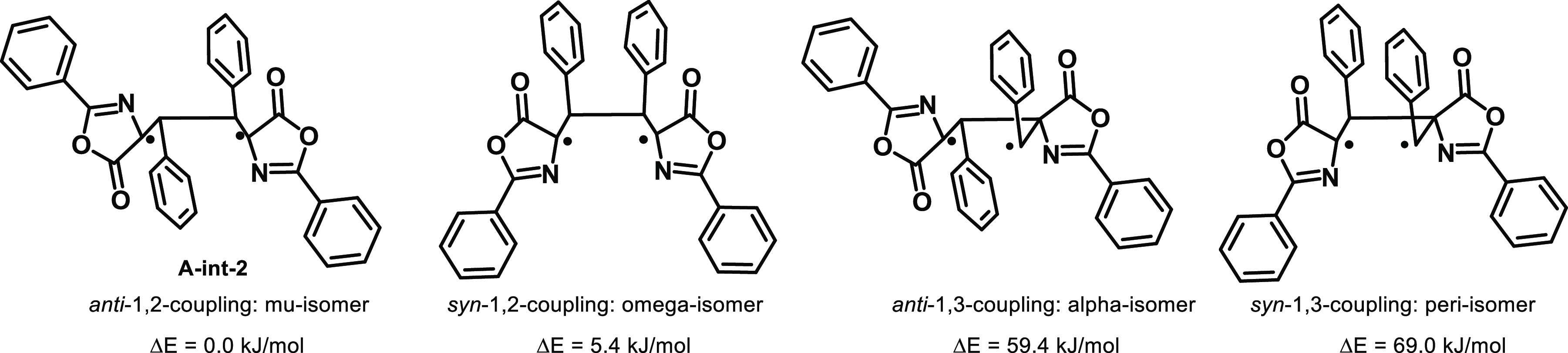
Relative energies of the triplet diradical intermediates leading to different isomers.

Calculation of the energy of triplet 1,4-diradical **A-int-2** in other geometries that could lead to other plausible isomers also shed some light on the selectivity of the reaction. Thus, we compared the energy of the intermediates involved in the head-to-head 1,2-couplings in *anti* (μ-isomer) and *syn* (ω-isomer) forms with those involved in the head-to-tail 1,3-couplings in *anti* (α-isomer) and *syn* (peri isomer) forms, taking into account the fact that the final conformation of the oxazolone is *E* (see the Supporting Information). The relative energies of these intermediates (Figure 12) show that the species formed via 1,2-coupling are much more stabilized than those formed via 1,3-coupling and the *anti* arrangement appears to be slightly more stabilized than the *syn* one.

## Conclusion

The fully stereoselective synthesis of cyclobutanes in solution is possible in the absence of templates. In this respect, we have demonstrated how methyl esters of 1,2-diaminotruxinic bis-amino acids can be obtained as single isomers with complete regio- and stereoselectivity by Ru-photocatalyzed [2+2] photocycloaddition of (*Z*)-4-aryliden-5(4*H*)-oxazolones in solution. The reaction is promoted by blue light (465 nm); the photocatalyst is [Ru(bpy)_3_](BF_4_)_2_ (5 mol %), and the μ-isomer of the cyclobutane is obtained. The reaction has been performed in batch and flow reactors, with a much better performance being achieved in flow devices (reaction time of 1 h vs 24 or 48 h). The photochemically reactive species has been characterized as a triplet diradical by laser flash photolysis, a technique that has also shown that this reactive species is the same irrespective of the geometry of the starting oxazolone (*Z* or *E*). In addition, DFT calculations have shown that the first step of the [2+2] cycloaddition is formation of the C(H)–C(H) bond and that the (*Z*)- to (*E*)-isomerization takes place in a 1,4-diradical after the formation of this bond.

## Experimental Section

### General Methods

The [2+2] photocycloaddition reactions were carried out under an inert (Ar) atmosphere, using deoxygenated CH_2_Cl_2_. The CH_2_Cl_2_ was deoxygenated using at least four freeze–thaw cycles and stored under Ar at 4 °C. The ring-opening reactions were carried out in reagent-grade methanol in open air. Flash column liquid chromatographies were performed on aluminum oxide 90 neutral (50–200 μm) or silica gel (70–230 μm). Infrared spectra (4000–380 cm^–1^) were recorded on a PerkinElmer Spectrum One IR spectrophotometer. ^1^H, ^13^C, and ^19^F NMR spectra of the isolated products **2** and **3** were recorded in CDCl_3_ or CD_2_Cl_2_ solutions at 25 °C (other temperatures were specified) on Bruker AV300, Bruker AV500, and Varian Unity INOVA 500 spectrometers (δ in parts per million, *J* in hertz) at ^1^H operating frequencies of 300.13, 500.13, and 499.77 MHz, respectively. ^1^H and ^13^C NMR spectra were referenced using the solvent signal as the internal standard, while ^19^F NMR spectra were referenced to CFCl_3_. The assignment of ^1^H NMR peaks has been performed through standard two-dimensional (2D) ^1^H–COSY (2K points in *t*_2_ using a spectral width of 10 ppm; 128 *t*_1_ experiments were recorded and zero-filled to 1K; for each *t*_1_ value, four scans were signal-averaged using a recycle delay of 1 s) and selective one-dimensional (1D) ^1^H SELNOE experiments. Typical mixing times in the case of selective 1D SELNOE experiments were in the range of 1.2–1.8 s, as a function of the irradiated signal. These values of optimized mixing times were set equal to the longitudinal relaxation time *T*_1_, determined using the inversion–recovery sequence. The ^13^C NMR peaks were identified using standard ^1^H–^13^C edited HSQC and ^1^H–^13^C HMBC 2D experiments. In both cases, 4K points in *t*_2_ using spectral widths of 10 ppm (^1^H) and 200 ppm (^13^C) were used, with averaged values of the coupling constants ^1^*J*_CH_ of 145 Hz and long-range *^n^J*_CH_ of 10 Hz. Typically, 256 *t*_1_ experiments were recorded and zero-filled to 2K. For each *t*_1_ value, 8 (HSQC) or 32 (HMBC) scans were signal-averaged using a recycle delay of 1 s. ESI (ESI^+^) mass spectra were recorded using an Esquire 3000 ion-trap mass spectrometer (Bruker Daltonic GmbH) equipped with a standard ESI/APCI source. Samples were introduced by direct infusion with a syringe pump. Nitrogen served as both the nebulizer gas and the dry gas. HRMS and ESI (ESI^+^) mass spectra were recorded using a MicroToF Q, API-Q-ToF ESI instrument with a mass range from *m*/*z* 20 to 3000 and a mass resolution of 15000 (full width at half-maximum). The absorption spectra of oxazolones **1a–1j** have been recorded in an Evolution 600 UV–vis spectrophotometer in CH_2_Cl_2_ solutions at a concentration of 10^–5^ M. The quenching of the phosphorescence of [Ru(bpy)_3_](BF_4_)_2_ by oxazolone **1c** was measured on a Horiba Jobin Yvon Fluorolog FL-3.11 spectrophotometer. All spectra have been recorded at 25 °C using a 10 mm quartz cuvette. The electrochemical measurements (cyclic voltammetry of oxazolone **1c**) were carried out using a Voltalab50 potentiostat/galvanostat. A glass electrochemical cell was used, with the typical configuration of three electrodes: a Pt working electrode, another Pt counter electrode, and the SCE electrode. The solution of the pure electrolyte (NBu_4_PF_6_, 0.1 M) was measured over the whole window of the solvent (CH_2_Cl_2_) to check the absence of electroactive impurities. The concentration of oxazolone **1c** was 5 × 10^–4^ M. The melting points (degrees Celsius) were determined on a Gallenkamp apparatus and are uncorrected. The oxazolones **1** used as starting materials were synthesized according to published methods.^24^ Compound [Ru(bpy)_3_](BF_4_)_2_ was also prepared following published procedures^40^ and stored under a protecting atmosphere (Ar) at 4 °C.

### General Procedure for *In Situ* NMR Monitoring

#### *In Situ* Irradiation Setup

The irradiation source consists of a model DL5146-101S blue laser diode with an optical output power of 1.6 W and peaks at 450 nm. To address uniform irradiation of the sample volume, current (LDC240C) and temperature (TED200C) controllers are used to regulate the amount of output light (see Figure S1). An optical fiber with an internal diameter of 600 μm guides the light beam from the laser diode to the detection region of the detection coil. Two lenses are employed to collimate the light beam that enters through the optical fiber. The tip of the fiber is sandblasted as reported to obtain uniform illumination from both the tip and the core.^41^ To fix the fiber inside the NMR tube, a coaxial insert is employed, not only to fix the fiber but also to protect the fiber from the reaction mixture and prevent the evaporation of the solvent as a consequence of the heat from the tip of the fiber. A photometer (PM100D) provides a value for the optical power of 525 mW, obtained out of the fiber when working at 750 mA.

#### Sample Preparation for *In Situ* Illumination NMR Experiments

Ten milligrams of oxazolone **1c** (75 mM) and 2 mg of [Ru(bpy)_3_](BF_4_)_2_ photocatalyst are added to an amberized 5 mm NMR tube, and CD_2_Cl_2_ is used as the solvent. Argon flux is employed to remove the O_2_ present in the reaction mixture to avoid quenching of the excited state. Consecutive ^1^H NMR experiments are launched immediately after irradiation begins. The NMR acquisition parameters define a time per spectrum of 30 s (relaxation delay of 1 s, acquisition time of 2 s, 10 scans).

#### Irradiation Setup for Batch or Continuous-Flow Synthesis

The irradiation setup consists of a Teflon capillary (flow reactions) or a balloon (batch reactions) irradiated by a printed circuit board (PCB) formed by 18, 20, or 25 LED bulbs of 10 mm diameter LEDs. The LEDs are serially connected in blocks of six, with a minimum voltage possible (3 V) and a resistance of 60 Ω (according to Ohm’s law). The output power per LED unit (blue, 465 nm) is 250 kmcd; the optical output power of the PCB of LEDs measured with a photometer (PM100D, Thorlabs) is 1 W. The PCB (dimensions of 7 cm × 6 cm) and the flow reactor or the balloon are placed inside a custom-built setup for fixing the light source and the sample container and dissipate the excess heat. For the flow reactions, a total length of 0.4 m (inner diameter of 500 μm) of the Teflon capillary is exposed to the light of the LEDs, via placement of the coil of the capillary on top of the light source, maximally reducing the distance between the LEDs and the coil. The length of the irradiated capillary (0.2 m) results in a reaction volume of 40 μL. A concave mirror is placed in front of the PCB to maximize the light that irradiates the coil. The system works on continuous flow, pumping the sample by a programmable Labtrix Start syringe pump (SGE glass gas chromatography syringe with a volume of 1 mL and an inner diameter of 4.61 mm). The illumination setup (laser diode, current and temperature controllers, lens, optical fiber, and photometer) were purchased from Thorlabs. Light-emitting diodes (LEDs) were purchased from Topbright.

#### Flow Sample Preparation

Oxazolone **1** at a concentration that depends on its solubility is added to a glass vial along with [Ru(bpy)_3_](BF_4_)_2_ as a photocatalyst (5 mol %, absorption maximum of 452 nm). Dichloromethane (500 μL) is selected as the solvent due to its higher solubility for both the oxazolone and the photocatalyst. The reaction sample is introduced into a sonication bath to ensure the complete dissolution of the reagents and then transferred to the glass syringe for the beginning of the reaction (see Figure S2). The syringe pump is programmed for a residence time of 30 min (1.33 μL/min). Two consecutive reactions are performed, refreshing the amount of photocatalyst and eliminating the “used” one; thus, higher yields are obtained. Continuous illumination of the microreactor leads to the higher efficiency of the reaction. The reaction mixture is collected in a glass vial, and off-line NMR analysis is carried out (20 μL of the reaction mixture is transferred into a 5 mm NMR tube for the NMR characterization, using CD_2_Cl_2_ as the solvent). The use of very small volumes and the optimum mixing of the reagents minimize the bleaching of the photocatalyst due to the presence of oxygen in the samples; therefore, these experiments are carried out without special precautions against the exclusion of oxygen.

### X-ray Crystallography

X-ray quality crystals of **2c**, **3c**, and **3d** were grown by slow diffusion of *n*-pentane into CH_2_Cl_2_ solutions of the crude product at −18 °C for several weeks. One selected single crystal of each compound was mounted at the end of a quartz fiber in a random orientation, covered with perfluorinated oil (magic oil) (**2c**) or paratone oil (**3c** or **3d**) on a MiTeGen microMounts cryoloop, and placed under a cold stream of N_2_ gas. Data were collected at 100 K (**3c** and **3d**) or 150 K (**2c**) on an Oxford Diffraction Xcalibur Sapphire 3 (**2c**) or Bruker D8 Venture (**3c** and **3d**) diffractometer, using graphite-monochromated Mo Kα radiation (λ = 0.71073 Å) (**2c** and **3d**) or Cu Kα radiation (λ = 1.54178 Å) (**3c**). A hemisphere of data was collected on the basis of ω-scan and ϕ-scan runs. The diffraction frames were integrated using CrysAlis RED^42^ or SAINT,^43^ and the integrated intensities were corrected for absorption with SADABS.^44^ The structures were determined and developed by Fourier methods.^45^ All non-hydrogen atoms were refined with anisotropic displacement parameters. The H atoms were placed at idealized positions and treated as riding atoms. Each H atom was assigned an isotropic displacement parameter equal to 1.2 times the equivalent isotropic displacement parameter of its parent atom. To determine and refine the structure, SHELX-97^46^ and Bruker APEX3 Software Package^47^ were used. The structures were refined to *F*_o_^2^, and all reflections were used in the least-squares calculations.^46^ CCDC 1887636 (**2c**), CCDC 1971420 (**3c**), and CCDC 1971421 (**3d**) contain the supplementary crystallographic data for this paper. These data can be obtained free of charge from The Cambridge Crystallographic Data Centre via www.ccdc.cam.ac.uk/data_request/cif.

### Photophysical Experiments

A pulsed Nd:YAG SL404G-10 Spectron Laser Systems laser at an excitation wavelength of 532 nm was employed to carry out the laser flash photolysis (LFP) experiments. The energy of the single pulses (∼10 ns duration) was <15 mJ pulse^–1^. The laser flash photolysis system is formed by the pulsed laser, a pulsed Lo255 Oriel xenon lamp, a model 77,200 Oriel monochromator, an Oriel photomultiplier tube (PMT) housing, a model 70,705 PMT power supply, and a TDS-640A Tektronix oscilloscope. Quenching rate constants (*k*_q_) were determined according to the Stern–Volmer equation [1/τ = 1/τ_ο_ + *k*_q_[Q], where τ_ο_ is the triplet lifetime of ^3^[Ru(bpy)_3_^2+^]* in the absence of oxazolone (Q), τ is the lifetime of ^3^[Ru(bpy)_3_^2+^]* in the presence of a given concentration of oxazolone, and [Q] is the oxazolone concentration]. The quenching rate constants (*k*_q_, M^–1^ s^–1^) were the corresponding slopes of the linear fittings of the Stern–Volmer plots.

### Computational Details

The geometries of the triplet intermediates shown in Figure 11 have been optimized using first-principles methods based in DFT where four different functionals (B3LYP-D3,^38a^ PBE-D3,^38b^ M062X-D3,^38c^ and wB97XD^38d^) were used for the sake of completeness and to allow a more critical and safe interpretation of the results. Also, for the same reason, two basis sets were employed: Def2-TZVP^38e^ and cc-pVTZ.^38f^ All of these functionals contain or have been completed (using Grimme’s D3 approach^38g^) with corrections for dispersion terms that are essential for the correct account of energetics in conformationally large molecules. All of the calculations have been performed using an updated version of Gaussian09^39^ software. For each molecule, the minimum energy conformation of the triplet state has been calculated, and the corresponding triplet energies have been obtained. The geometries of the triplet intermediates shown in Figure 12 were optimized at the DFT level by using the M062X^38c^ functional as implemented in Gaussian 09.^39^ Initial structure optimizations were carried out by using 6-31G(d,p), and further refinements were performed at the M062X/def2TZVPP level. Solvent effects were taken into account at the same levels of theory by applying the conductor-like polarizable continuum model and Truhlar’s radii and non-electrostatic terms (SMD),^48^ using dichloromethane as the solvent, as in the experimental conditions. The solvent-corrected Δ*G* energy was calculated as the difference between the gas phase and the SMD solvated model. The critical stationary points were characterized by frequency calculations to verify that they have the right number of imaginary frequencies.

### Synthesis of Cyclobutane Intermediates **2a–2h**

#### Synthesis of Bis(oxazolone)cyclobutane **2a**

A deoxygenated solution of (*Z*)-4-benzyliden-2-phenyl-5(4*H*)oxazolone **1a** (300.0 mg, 1.20 mmol) and photocatalyst [Ru(bpy)_3_](BF_4_)_2_ (44.7 mg, 0.060 mmol) in CH_2_Cl_2_ (10 mL) under an argon atmosphere was irradiated with blue light (465 nm, 20 W) for 24 h at room temperature. After the reaction time, the solvent was removed to dryness while irradiation was maintained to prevent the retro-[2+2] reaction. The solid residue thus obtained was treated with CHCl_3_ (8 mL) and stirred for an additional 5 min at room temperature. The resulting suspension was filtered through a Celite bed to remove the insoluble Ru photocatalyst, washing the Celite with additional CHCl_3_ (4 mL). This colorless solution was evaporated to dryness, and the solid residue treated with *n*-pentane (25 mL). Further stirring promoted the precipitation of a white solid, which was filtered, washed with additional pentane (10 mL), dried by suction, and identified as **2a**. Yield: 67.7 mg (23%). Mp: 145–146 °C. HRMS (ESI) *m*/*z*: [M + Na]^+^ calcd for C_32_H_22_N_2_NaO_4_, 521.1472; found, 521.1482. IR (ν, cm^–1^): 1818 (ν_CO_), 1636 (ν_CN_). ^1^H NMR (CDCl_3_, 300.13 MHz): δ 8.07 (m, 2H), 7.61 (tt, 1H, ^3^*J*_HH_ = 7.4 Hz, ^4^*J*_HH_ = 1.1 Hz), 7.49 (t, 2H, ^3^*J*_HH_ = 7.3 Hz), 7.36–7.29 (m, 5H), 5.28 (s, 1H). ^13^C{^1^H} NMR (CDCl_3_, 75.5 MHz): δ 172.4, 163.9, 135.4, 133.5, 128.9, 128.8, 128.6, 127.9, 126.9, 124.9, 77.0, 46.3.

#### Synthesis of Bis(oxazolone)cyclobutane **2b**

The synthesis of bis(oxazolone)cyclobutane **2b** has been carried out following the same experimental procedure as that described for **2a**. Therefore, oxazolone **1b** (300.0 mg, 1.08 mmol) and the photocatalyst (39.9 mg, 0.053 mmol) were irradiated in CH_2_Cl_2_ (10 mL) with blue light (465 nm) for 24 h to afford a yellow solid (123.6 mg), whose characterization showed it to be a 1.0:4.0 **2b**/**1b** mixture. IR (ν, cm^–1^): 1820 (ν_CO_), 1652 (ν_CN_). HRMS (ESI) *m*/*z*: [M + Na]^+^ calcd for C_34_H_26_N_2_NaO_6_, 581.1683; found, 581.1659. ^1^H NMR (CD_2_Cl_2_, 300.13 MHz): δ 8.05 (m, 2H), 7.55–7.49 (m, 3H), 7.26 (m, 2H), 6.89 (m, 2H), 5.17 (s, 1H), 3.79 (s, 3H). ^13^C{^1^H} NMR (CD_2_Cl_2_, 75.5 MHz): δ 172.5, 163.7, 159.5, 133.5, 128.8, 128.4, 128.3, 127.2, 125.0, 114.2, 77.0, 55.2, 46.2.

#### Synthesis of Bis(oxazolone)cyclobutane **2c**

The synthesis of bis(oxazolone)cyclobutane **2c** has been carried out following the same experimental procedure as that described for **2a**. Therefore, oxazolone **1c** (300.0 mg, 1.14 mmol) and the photocatalyst (42.3 mg, 0.057 mmol) were irradiated in CH_2_Cl_2_ (10 mL) with blue light (465 nm) for 24 h to afford pure **2c** as a white solid. Yield: 58.3 mg (20%). Mp: 134–135 °C. HRMS (ESI) *m*/*z*: [M + Na]^+^ calcd for C_34_H_26_N_2_NaO_4_, 549.1785; found, 549.1788. ^1^H NMR (CDCl_3_, 300.13 MHz): δ 8.05 (m, 2H), 7.60 (tt, 1H, ^3^*J*_HH_ = 7.6 Hz, ^4^*J*_HH_ = 1.2 Hz), 7.49 (t, 2H, ^3^*J*_HH_ = 7.6 Hz), 7.18 (AA′ part of an AA′BB′ spin system, 2H), 7.13 (BB′ part of an AA′BB′ spin system, 2H), 5.22 (s, 1H), 2.33 (s, 3H). ^13^C{^1^H} NMR (CDCl_3_, 75.5 MHz): δ 172.6, 163.7, 137.7, 133.5, 132.3, 129.6, 128.8, 128.6, 126.9, 125.0, 77.0, 46.2, 21.1.

#### Synthesis of Bis(oxazolone)cyclobutane **2d**

The synthesis of bis(oxazolone)cyclobutane **2d** has been carried out following the same experimental procedure as that described for **2a**. Therefore, oxazolone **1d** (300.0 mg, 1.06 mmol) and the photocatalyst (39.3 mg, 0.053 mmol) were irradiated in CH_2_Cl_2_ (10 mL) with blue light (465 nm) for 24 h to afford a pale-yellow solid (177.6 mg), whose characterization showed it to be a 1.0:5.2 **2d**/**1d** mixture. IR (ν, cm^–1^): 1820 (ν_CO_), 1632 (ν_CN_). HRMS (ESI) *m*/*z*: [M + Na]^+^ calcd for C_32_H_20_Cl_2_N_2_NaO_4_, 589.0692; found, 589.0690. ^1^H NMR (CD_2_Cl_2_, 300.13 MHz): δ 8.04 (m, 2H), 7.69 (m, 1H), 7.62 (m, 2H), 7.35 (AA′ part of an AA′BB′ spin system, 2H), 7.25 (BB′ part of an AA′BB′ spin system, 2H), 5.18 (s, 1H). ^13^C{^1^H} NMR (CD_2_Cl_2_, 75.5 MHz): δ 172.2, 164.2, 137.0, 133.7, 133.7, 133.6, 129.1, 128.5, 128.4, 125.5, 76.9, 45.8.

#### Synthesis of Bis(oxazolone)cyclobutane **2e**

The synthesis of bis(oxazolone)cyclobutane **2e** has been carried out following the same experimental procedure as that described for **2a**. Therefore, oxazolone **1e** (300.0 mg, 1.12 mmol) and the photocatalyst (41.7 mg, 0.056 mmol) were irradiated in CH_2_Cl_2_ (10 mL) with blue light (465 nm) for 24 h to afford a pale-yellow solid (88.2 mg), whose characterization showed it to be a 1.7:1.0 **2e**/**1e** mixture. HRMS (ESI) *m*/*z*: [M + Na]^+^ calcd for C_32_H_20_F_2_N_2_NaO_4_, 557.1283; found, 557.1272. IR (ν, cm^–1^): 1818 (ν_CO_), 1639 (ν_CN_). ^1^H NMR (CDCl_3_, 300.13 MHz): δ 8.04 (m, 2H), 7.65–7.51 (m, 1H; overlapped with signals due to **1e**), 7.48 (t, 2H, ^3^*J*_HH_ = 7.3 Hz), 7.25 (m, 2H), 7.03 (t, 2H, ^3^*J*_HH_ = ^3^*J*_HF_ = 8.7 Hz), 5.18 (s, 1H). ^19^F NMR (CDCl_3_, 282.40 MHz): δ −113.68 (tt, ^3^*J*_FH_ = 8.7 Hz, ^4^*J*_FH_ = 5.1 Hz). ^13^C{^1^H} NMR (CDCl_3_, 75.5 MHz): δ 172.3, 164.2, 162.5 (d, ^1^*J*_CF_ = 238 Hz), 130.6 (d, ^4^*J*_CF_ = 3 Hz), 129.0, 128.8, 128.7 (d, ^3^*J*_CF_ = 7 Hz), 128.6, 124.7, 116.0 (d, ^2^*J*_CF_ = 21 Hz), 77.0, 45.9.

#### Synthesis of Bis(oxazolone)cyclobutane **2g**

The synthesis of bis(oxazolone)cyclobutane **2g** has been carried out following the same experimental procedure as that described for **2a**. Therefore, oxazolone **1g** (300.0 mg, 0.95 mmol) and the photocatalyst (35.1 mg, 0.047 mmol) were irradiated in CH_2_Cl_2_ (10 mL) with blue light (465 nm) for 24 h to afford pure **2g** as a white solid. Yield: 94.5 mg (31%). Mp: 177–178 °C. HRMS (ESI) *m*/*z*: [M]^+^ calcd for C_34_H_20_F_6_N_2_O_4_, 634.1327; found, 634.1318. ^1^H NMR (CDCl_3_, 300.13 MHz): δ 8.05 (m, 2H), 7.65–7.60 (m, 3H), 7.50 (t, 2H, ^3^*J*_HH_ = 7.3 Hz), 7.39 (AA′ part of an AA′BB′ spin system, 2H), 5.31 (s, 1H). ^13^C{^1^H} NMR (CDCl_3_, 75.5 MHz): δ 172.1, 164.5, 138.8, 133.9, 130.5 (q, ^2^*J*_CF_ = 34.7 Hz), 128.9, 128.7, 127.3, 126.0, 124.5, 123.8 (q, ^1^*J*_CF_ = 273 Hz), 77.0, 45.8. ^19^F NMR (CDCl_3_, 282.40 MHz): δ −62.74 (s).

#### Synthesis of Bis(oxazolone)cyclobutane **2h**

The synthesis of bis(oxazolone)cyclobutane **2h** has been carried out following the same experimental procedure as that described for **2a**. Therefore, oxazolone **1h** (300.0 mg, 1.08 mmol) and the photocatalyst (40.2 mg, 0.054 mmol) were irradiated in CH_2_Cl_2_ (10 mL) with blue light (465 nm) for 24 h to afford a pale-yellow solid (34.9 mg), whose characterization showed it to be a 2.7:1.0 **2h**/**1h** mixture. IR (ν, cm^–1^): 1816 (ν_CO_), 1634 (ν_CN_). HRMS (ESI) *m*/*z*: [M + Na]^+^ calcd for C_36_H_30_N_2_NaO_4_, 577.2098; found, 577.2094. ^1^H NMR (CDCl_3_, 300.13 MHz): δ 8.05 (m, 2H), 7.60 (m, 1H), 7.48 (t, 2H, ^3^*J*_HH_ = 7.5 Hz), 7.08 (d, 1H, ^3^*J*_HH_ = 8 Hz), 7.07 (s, 1H), 7.03 (dd, 1H, ^3^*J*_HH_ = 8.0 Hz, ^4^*J*_HH_ = 3.0 Hz), 5.19 (s, 1H), 2.23 (s, 3H), 2.21 (s, 3H). ^13^C{^1^H} NMR (CDCl_3_, 75.5 MHz): δ 172.6, 163.7, 137.0, 136.3, 133.4, 132.7, 130.1, 128.8, 128.6, 128.2, 125.0, 124.4, 77.0, 46.1, 19.9, 19.5.

### Synthesis of 1,2-Diaminotruxinic Acid Derivatives **3a–3l**

#### Synthesis of Dimethyl-1,2-bis(benzamido)-3,4-diphenylcyclobutane-1,2-dicarboxylate **3a**

Oxazolone **1a** (284.9 mg, 1.143 mmol) and photocatalyst [Ru(bpy)_3_](BF_4_)_2_ (40.0 mg, 0.054 mmol) were dissolved under an Ar atmosphere in dexoygenated CH_2_Cl_2_ (5 mL). This red solution was irradiated with the blue light (465 nm) provided by a LED lamp (20 W) for 24 h. After the reaction, the solvent was evaporated to dryness while irradiation was continued, to avoid retro-[2+2] reaction. The solid residue was redissolved in methanol (6 mL); a catalytic amount of NaOMe (9 mg, 0.167 mmol) was added, and the resulting suspension was heated to the reflux temperature in an oil bath for 30 min. During this time, the initial suspension dissolved, giving a red solution, which was allowed to cool at room temperature and then evaporated to dryness. The dry residue was treated with CHCl_3_ (4 mL) and subjected to flash chromatography on silica gel using chloroform as the eluent. The colorless solution, containing cyclobutane **3a**, was evaporated to dryness, and the solid residue treated with *n*-pentane to give a white solid, which was filtered, washed with *n*-pentane (2 × 2 mL), dried by suction, and identified as **3a**. Yield: 249.6 mg (87%). Pure crystalline **3a** can be obtained by slow diffusion of *n*-pentane into a CH_2_Cl_2_ solution of **3a** at −18 °C. HRMS (ESI) *m*/*z*: [M + Na]^+^ calcd for C_34_H_30_N_2_NaO_6_, 585.1996; found, 585.2012. ^1^H NMR (CDCl_3_, 300.13 MHz): δ 7.91 (m, 2H), 7.82 (s, 1H), 7.56 (tt, 1H, ^3^*J*_HH_ = 6.9 Hz, ^4^*J*_HH_ = 2.4 Hz), 7.48 (t, 2H, ^3^*J*_HH_ = 6.9 Hz), 7.42 (m, 2H), 7.34–7.23 (m, 3H), 5.07 (s, 1H), 3.31 (s, 3H). ^13^C{^1^H} NMR (CDCl_3_, 75.5 MHz): δ 168.8, 167.7, 136.8, 133.9, 128.7, 128.2, 127.7, 131.9, 127.4, 127.2, 69.0, 52.4, 47.0.

#### Synthesis of Dimethyl-1,2-bis(benzamido)-3,4-bis(4-methoxyphenyl)cyclobutane-1,2-dicarboxylate **3b**

Compound **3b** was obtained following an experimental procedure identical to that described for **3a**, but using an optimized reaction time. Therefore, oxazolone **1b** (276.0 mg, 0.989 mmol) and [Ru(bpy)_3_](BF_4_)_2_ (34.7 mg, 0.047 mmol) (5% mole ratio) were irradiated for 24 h in CH_2_Cl_2_ (5 mL) and then reacted with NaOMe in refluxing MeOH (5 mL) for 45 min to give **3b** as a white solid after chromatographic purification and crystallization in a CH_2_Cl_2_/*n*-pentane solvent. Yield: 186.4 mg (67%). Mp: 157–158 °C. HRMS (ESI) *m*/*z*: [M + Na]^+^ calcd for C_36_H_34_N_2_NaO_8_, 645.2207; found, 645.2234. ^1^H NMR (CDCl_3_, 300.13 MHz): δ 7.90 (m, 2H), 7.73 (s, 1H), 7.56 (tt, 1H, ^3^*J*_HH_ = 7 Hz, ^4^*J*_HH_ = 1.5 Hz), 7.48 (t, 1H, ^3^*J*_HH_ = 7 Hz), 7.33 (m, 2H), 6.84 (m, 2H), 4.91 (s, 1H), 3.79 (s, 3H), 3.35 (s, 3H). ^13^C{^1^H} NMR (CDCl_3_, 75.5 MHz): δ 168.9, 167.6, 158.9, 133.9, 131.9, 129.0, 128.7, 128.5, 127.2, 113.6, 68.9, 55.2, 52.4, 46.9.

#### Synthesis of Dimethyl-1,2-bis(benzamido)-3,4-bis(*p*-tolyl)cyclobutane-1,2-dicarboxylate **3c**

Compound **3c** was obtained following an experimental procedure identical to that described for **3a**, but using an optimized reaction time. Therefore, oxazolone **1c** (293.3 mg, 1.115 mmol) and [Ru(bpy)_3_](BF_4_)_2_ (40.4 mg, 0.054 mmol) (5% mole ratio) were irradiated for 20 h in CH_2_Cl_2_ (5 mL) and then reacted with NaOMe in refluxing MeOH (5 mL) for 30 min to give **3c** as a white solid after chromatographic purification and crystallization in a CH_2_Cl_2_/*n*-pentane solvent. Yield: 80.8 mg (30%). Mp: 197–198 °C. HRMS (ESI) *m*/*z*: [M + Na]^+^ calcd for C_36_H_34_N_2_NaO_6_, 613.2309; found, 613.2332. ^1^H NMR (CDCl_3_, 300.13 MHz): δ 7.90 (m, 2H), 7.75 (s, 1H), 7.56 (tt, 1H, ^3^*J*_HH_ = 7.0 Hz, ^4^*J*_HH_ = 1.5 Hz), 7.49 (t, 2H, ^3^*J*_HH_ = 7.4 Hz), 7.29 (m, 2H), 7.11 (m, 2H), 4.95 (s, 1H), 3.33 (s, 3H), 2.32 (s, 3H). ^13^C{^1^H} NMR (CDCl_3_, 75.5 MHz): δ 168.9, 167.6, 137.0, 134.0, 133.5, 131.8, 128.9, 128.7, 127.7, 127.2, 68.9, 52.4, 47.0, 21.1.

#### Synthesis of Dimethyl-1,2-bis(benzamido)-3,4-bis(4-chlorophenyl)cyclobutane-1,2-dicarboxylate **3d**

Compound **3d** was obtained following an experimental procedure identical to that described for **3a**, but using an optimized reaction time. Therefore, oxazolone **1d** (289.1 mg, 1.021 mmol) and [Ru(bpy)_3_](BF_4_)_2_ (37.1 mg, 0.050 mmol) (5% mole ratio) were irradiated for 19 h in CH_2_Cl_2_ (5 mL) and then reacted with NaOMe in refluxing MeOH (5 mL) for 45 min to give **3d** as a white solid after chromatographic purification and crystallization in a CH_2_Cl_2_/*n*-pentane solvent. Yield: 199.5 mg (69%). Mp: 222–223 °C. HRMS (ESI) *m*/*z*: [M + Na]^+^ calcd for C_34_H_28_Cl_2_N_2_NaO_6_, 653.1217; found, 653.1228. ^1^H NMR (CDCl_3_, 300.13 MHz): δ 7.88 (m, 2H), 7.79 (s, 1H), 7.57 (tt, 1H, ^3^*J*_HH_ = 7.2 Hz, ^4^*J*_HH_ = 2.4 Hz), 7.49 (m, 2H), 7.35–7.27 (AA′BB′ spin system, 4H), 4.97 (s, 1H), 3.36 (s, 3H). ^13^C{^1^H} NMR (CDCl_3_, 75.5 MHz): δ 168.6, 167.8, 134.9, 133.6, 133.5, 132.1, 129.2, 128.8, 128.5, 127.2, 68.8, 52.6, 46.6.

#### Synthesis of Dimethyl-1,2-bis(benzamido)-3,4-bis(4-fluorophenyl)cyclobutane-1,2-dicarboxylate **3e**

Compound **3e** was obtained following an experimental procedure identical to that described for **3a**, but using an optimized reaction time. Therefore, oxazolone **1e** (297.7 mg, 1.06 mmol) and [Ru(bpy)_3_](BF_4_)_2_ (42.4 mg, 0.057 mmol) (5% mole ratio) were irradiated for 46 h in CH_2_Cl_2_ (5 mL) and then reacted with NaOMe in refluxing MeOH (5 mL) for 45 min to give **3e** as a white solid after chromatographic purification and crystallization in a CH_2_Cl_2_/*n*-pentane solvent. Yield: 126.8 mg (43%). Mp: 155–156 °C. HRMS (ESI) *m*/*z*: [M + Na]^+^ calcd for C_34_H_28_F_2_N_2_NaO_6_, 621.1808; found, 621.1803. ^1^H NMR (CDCl_3_, 300.13 MHz): δ 7.89 (m, 2H), 7.74 (s, 1H), 7.57 (tt, 1H, ^3^*J*_HH_ = 9 Hz, ^4^*J*_HH_ = 3 Hz), 7.49 (t, 2H, ^3^*J*_HH_ = 7.5 Hz), 7.38 (dd, 2H, ^3^*J*_HH_ = 9 Hz, ^4^*J*_FH_ = 6 Hz), 7.01 (t, 2H, ^3^*J*_HH_ = ^3^*J*_FH_ = 9 Hz), 4.97 (s, 1H), 3.35 (s, 3H). ^13^C{^1^H}NMR (CDCl_3_, 75.5 MHz): δ 168.7, 167.8, 162.2 (d, ^1^*J*_CF_ = 246 Hz), 133.7, 132.2 (d, ^4^*J*_CF_ = 4 Hz), 132. 0, 129.5 (d, ^3^*J*_CF_ = 8 Hz), 128.7, 127.2, 115.2 (d, ^2^*J*_CF_ = 21 Hz), 68.9, 52.5, 46.7. ^19^F NMR (CDCl_3_, 282.40 MHz): δ −114.75 (tt, ^3^*J*_FH_ = 9 Hz, ^4^*J*_FH_ = 6 Hz).

#### Synthesis of Dimethyl-1,2-bis(benzamido)-3,4-bis(4-nitrophenyl)cyclobutane-1,2-dicarboxylate **3f**

Compound **3f** was obtained following an experimental procedure identical to that described for **3a**, but using an optimized reaction time. Therefore, oxazolone **1f** (295.4 mg, 1.00 mmol) and [Ru(bpy)_3_](BF_4_)_2_ (38.0 mg, 0.051 mmol) (5% mole ratio) were irradiated for 48 h in CH_2_Cl_2_ (5 mL) and then reacted with NaOMe in refluxing MeOH (5 mL) for 45 min to give **3f** as a white solid after chromatographic purification and crystallization in a CH_2_Cl_2_/*n*-pentane solvent. Yield: 87.0 mg (30%). HRMS (ESI) *m*/*z*: [M + Na]^+^ calcd for C_34_H_28_N_4_NaO_10_, 675.1698; found, 675.1665. ^1^H NMR (CDCl_3_, 300.13 MHz): δ 8.21 (m, 2H), 7.89 (m, 2H), 7.72 (s, 1H), 7.63–7.50 (m, 5H), 5.16 (s, 1H), 3.39 (s, 3H). ^13^C{^1^H}NMR (CDCl_3_, 75.5 MHz): δ 168.3, 167.9, 147.4, 143.6, 133.2, 132. 4, 128.9, 128.8, 127.2, 123.6, 69.0, 52.9, 47.0.

#### Synthesis of Dimethyl-1,2-bis(benzamido)-3,4-bis(3,4-dimethylphenyl)cyclobutane-1,2-dicarboxylate **3h**

Compound **3h** was obtained following an experimental procedure identical to that described for **3a**, but using an optimized reaction time. Therefore, oxazolone **1h** (273.1 mg, 0.985 mmol) and [Ru(bpy)_3_](BF_4_)_2_ (36.6 mg, 0.049 mmol) (5% mole ratio) were irradiated for 21 h in CH_2_Cl_2_ (5 mL) and then reacted with NaOMe in refluxing MeOH (5 mL) for 30 min to give **3h** as a white solid after chromatographic purification and crystallization in a CH_2_Cl_2_/*n*-pentane solvent. Yield: 113.6 mg (42%). Mp: 224–225 °C. HRMS (ESI) *m*/*z*: [M + Na]^+^ calcd for C_38_H_38_N_2_NaO_6_, 641.2630; found, 641.2622. ^1^H NMR (CDCl_3_, 300.13 MHz): δ 7.91 (m, 2H), 7.77 (s, 1H), 7.56 (tt, 1H, ^3^*J*_HH_ = 7.2 Hz, ^4^*J*_HH_ = 1.2 Hz), 7.49 (tt, 2H, ^3^*J*_HH_ = 7.8 Hz, ^4^*J*_HH_ = 1.2 Hz), 7.16 (s, 1H), 7.12 (dd, ^3^*J*_HH_ = 7.8 Hz, ^4^*J*_HH_ = 1.5 Hz), 7.06 (d, ^3^*J*_HH_ = 7.8 Hz), 4.91 (s, 1H), 3.33 (s, 3H), 2.24 (s, 3H), 2.22 (s, 3H). ^13^C{^1^H} NMR (CDCl_3_, 75.5 MHz): δ 169.0, 167.6, 136.2, 135.6, 134.1, 133.9, 131.8, 129.4, 129.0, 128.6, 127.2, 125.3, 69.0, 52.3, 47.0, 19.9, 19.4.

#### Synthesis of Dimethyl-1,2-bis(benzamido)-3,4-bis(3,4-dichlorophenyl)cyclobutane-1,2-dicarboxylate **3i**

Compound **3i** was obtained following an experimental procedure identical to that described for **3a**, but using an optimized reaction time. Therefore, oxazolone **1i** (300.9 mg, 0.949 mmol) and [Ru(bpy)_3_](BF_4_)_2_ (36.2 mg, 0.049 mmol) (5% mole ratio) were irradiated for 22 h in CH_2_Cl_2_ (5 mL) and then reacted with NaOMe in refluxing MeOH (5 mL) for 45 min to give **3i** as a white solid after chromatographic purification and crystallization in a CH_2_Cl_2_/*n*-pentane solvent. Yield: 197.5 mg (66%). HRMS (ESI) *m*/*z*: [M + Na]^+^ calcd for C_34_H_26_Cl_4_N_2_NaO_6_, 721.0437; found, 721.0427. ^1^H NMR (CDCl_3_, 300.13 MHz): δ 7.88 (m, 2H), 7.83 (s, 1H), 7.57 (tt, 1H, ^3^*J*_HH_ = 7.2 Hz, ^4^*J*_HH_ = 1.2 Hz), 7.53–7.44 (m, 2H), 7.38 (m, 1H), 7.25 (dd, 1H, ^3^*J*_HH_ = 8.4 Hz, ^4^*J*_HH_ = 2.1 Hz), 4.93 (s, 1H), 3.40 (s, 3H). ^13^C{^1^H} NMR (CDCl_3_, 75.5 MHz): δ 168.4, 167.9, 136.5, 133.4, 132.5, 132.2, 131.8, 130.3, 129.8, 128.8, 127.5, 127.2, 68.8, 52.8, 46.3.

#### Synthesis of Dimethyl-1,2-bis(benzamido)-3,4-bis(*m*-tolyl)cyclobutane-1,2-dicarboxylate **3j**

Compound **3j** was obtained following an experimental procedure identical to that described for **3a**, but using an optimized reaction time. Therefore, oxazolone **1j** (289.1 mg, 1.1 mmol) and [Ru(bpy)_3_](BF_4_)_2_ (41.0 mg, 0.055 mmol) (5% mole ratio) were irradiated for 24 h in CH_2_Cl_2_ (5 mL) and then reacted with NaOMe in refluxing MeOH (5 mL) for 30 min to give **3j** as a white solid after chromatographic purification and crystallization in a CH_2_Cl_2_/*n*-pentane solvent. Yield: 110.0 mg (38%). Mp: 150–151 °C. HRMS (ESI) *m*/*z*: [M + Na]^+^ calcd for C_36_H_34_N_2_NaO_6_, 613.2309; found, 613.2315. ^1^H NMR (CDCl_3_, 300.13 MHz): δ 7.91 (m, 2H), 7.76 (s, 1H), 7.58–7.46 (m, 4H), 7.23–7.17 (m, 2H), 7.07 (m, 1H), 4.99 (s, 1H), 3.31 (s, 3H), 2.34 (s, 3H). ^13^C{^1^H}NMR (CDCl_3_, 75.5 MHz): δ 168.9, 167.6, 137.7, 136.6, 134.0, 131.8, 128.7, 128.3, 128.2, 128.1, 127.2, 124.8, 69.0, 52.3, 46.9, 21.5.

## Supporting Information Available

Figures for the Experimental Section (illustration of the setup utilized for *in situ* NMR experiments and continuous-flow reactions), control ^1^H NMR spectra of oxazolones **1**, copies of ^1^H, ^13^C, and ^19^F NMR spectra for all new compounds **2** and **3**, DP4 characterization of the μ-isomer, conversions (percent) of oxazolones **1** to give cyclobutene-bis(oxazolones) **2** from two consecutive reactions in microreactors, copies of the absorption (UV–vis) spectra of (*Z*)-oxazolones **1** and selected (*E*)-oxazolones, quenching experiments, LFP experiments, cyclic voltammograms, and DFT calculations (optimized geometries and tables with absolute and relative energies) (PDF)
Geometries: coordinates of all species calculated in Figure 11, using different functionals, basis and solvents (ZIP)

## Accession Codes

CCDC 1887636 and 1971420–1971421 contain the supplementary crystallographic data for this paper. These data can be obtained free of charge via www.ccdc.cam.ac.uk/data_request/cif, or by emailing data_request@ccdc.cam.ac.uk, or by contacting The Cambridge Crystallographic Data Centre, 12 Union Road, Cambridge CB2 1EZ, UK; fax: +44 1223 336033.
