# Supplementary material for: Stereoselective, Ruthenium-Photocatalyzed Synthesis of 1,2-Diaminotruxinic Bis-amino Acids from 4-Arylidene-5(4H)-oxazolones
Source: J Org Chem. 2022 Feb 10;87(5):3529–45. doi: 10.1021/acs.joc.1c03092 (PMC8902759; doi:10.1021/acs.joc.1c03092)
Supplement: Supplementary file 1 — jo1c03092_si_001.pdf [file jo1c03092_si_001.pdf]

Stereoselective, ruthenium-photocatalyzed synthesis of 1,2-diaminotruxinic bis-amino acids from 4-arylidene-5(4*H*)-oxazolones

Sonia Sierra,[†] M. Victoria Gomez,[‡] Ana I. Jiménez,[†] Alexandra Pop,[#] Cristian Silvestru,[#] Maria Luisa Marín,[∇] Francisco Boscá,[∇] Germán Sastre,[∇] Enrique Gómez-Bengoa,[§] Esteban P. Urriolabeitia*[†]

† Instituto de Síntesis Química y Catálisis Homogénea, ISQCH (CSIC-Universidad de Zaragoza), Pedro Cerbuna 12, 50009 Zaragoza (Spain). Corresponding author: esteban@unizar.es

‡ Instituto Regional de Investigación Científica Aplicada (IRICA), Universidad de Castilla-La Mancha, Avenida Camilo José Cela s/n, 13071 Ciudad Real (Spain).

Departament of Chemistry, Supramolecular Organic and Organometallic Chemistry Centre (SOOMCC), Faculty of Chemistry and Chemical Engineering, Babeş-Bolyai University, 11 Arany Janos, 400028 Cluj−Napoca (Romania)

∇ Instituto Universitario Mixto de Tecnología Química (ITQ-UPV), Universitat Politècnica de València-Consejo Superior de Investigaciones Científicas, Av.de los Naranjos s/n, 46022 Valencia (Spain)

§ Departamento de Química Orgánica I, Universidad del País Vasco, UPV-EHU, Apdo. 1072, CP-20080 Donostia−San Sebastián (Spain)

SUPPORTING INFORMATION

Index of the Supporting Information

**Experimental Section**	
Figure S1: Experimental setup for the *in-situ* measurements	S2
Figure S2: Experimental setup for the measurement of flow reactions	S3
Table S1: Comparison of STY of oxazolones (*Z*)-**1** to give **2** obtained using batch and continuous-flow methodologies	S3
**NMR spectra of all prepared compounds**	
NMR spectra of all starting (*Z*)-4-aryliden-2-phenyl-5(4*H*)-oxazolones **1a-1j**	S4
NMR spectra of the cyclobutane-bis(oxazolone) intermediates **2a-2h** (except **2f**, see main text)	S9
NMR spectra of the 1,2-diaminotruxinic acid derivatives **3a-3j** (except **3g**, see main text)	S27
**Miscellaneous Information**	
All potential isomers for [2+2] cycloaddition and corresponding NMR symmetry	S50
DP4 characterization of mu isomer of **2a**	S51
Conversions (%) of oxazolones **1** to give cyclobutene-bis(oxazolones) **2** from two consecutive reactions in continuous flow conditions	S54
Absorption spectra in CH_2Cl_2 solution of *Z*-oxazolones **1** and some examples of *E*-oxazolones	S55
Cyclic Voltammetry of oxazolone **1c**	S59
Transient absorption spectra of $Ru(bpy)_3^{2+}$ (deaerated CH_2Cl_2) recorded at different times after the laser pulse	S59
NMR spectra of mixtures of oxazolones (*Z*)-**1c** or (*E*)-**1c** with the Ru-photocatalyst after 10 min irradiation with LED light (465 nm)	S60
Figure S3. Optimized geometries (B3LYP-D3/cc-pVTZ) of selected intermediates as shown in Figure 11.	S61
Figure S4. Optimized geometries of **A-int-1** using cc-pVTZ basis set and different functionals	S62
Table S2. Absolute (Ha) and relative (kJ/mol) DFT energies of minimum energy conformations of intermediates using four different functionals including dispersion: B3LYP-D3, M062X-D3, PBE-D3 and wB97XD, as well as cc-pVTZ as basis set.	S63
Table S3. Absolute (Ha) and relative (kJ/mol) DFT energies of minimum energy conformations of intermediates, using four different functionals including dispersion: B3LYP-D3, M062X-D3 and PBE-D3, and cc-pVTZ basis sets. The calculations include the presence of dichloromethane as solvent (using the SCRF/PCM option in Gaussian09).	S64
Table S4. Cartesian coordinates of calculated structures of intermediates shown in Figure 12	S65

Experimental Section

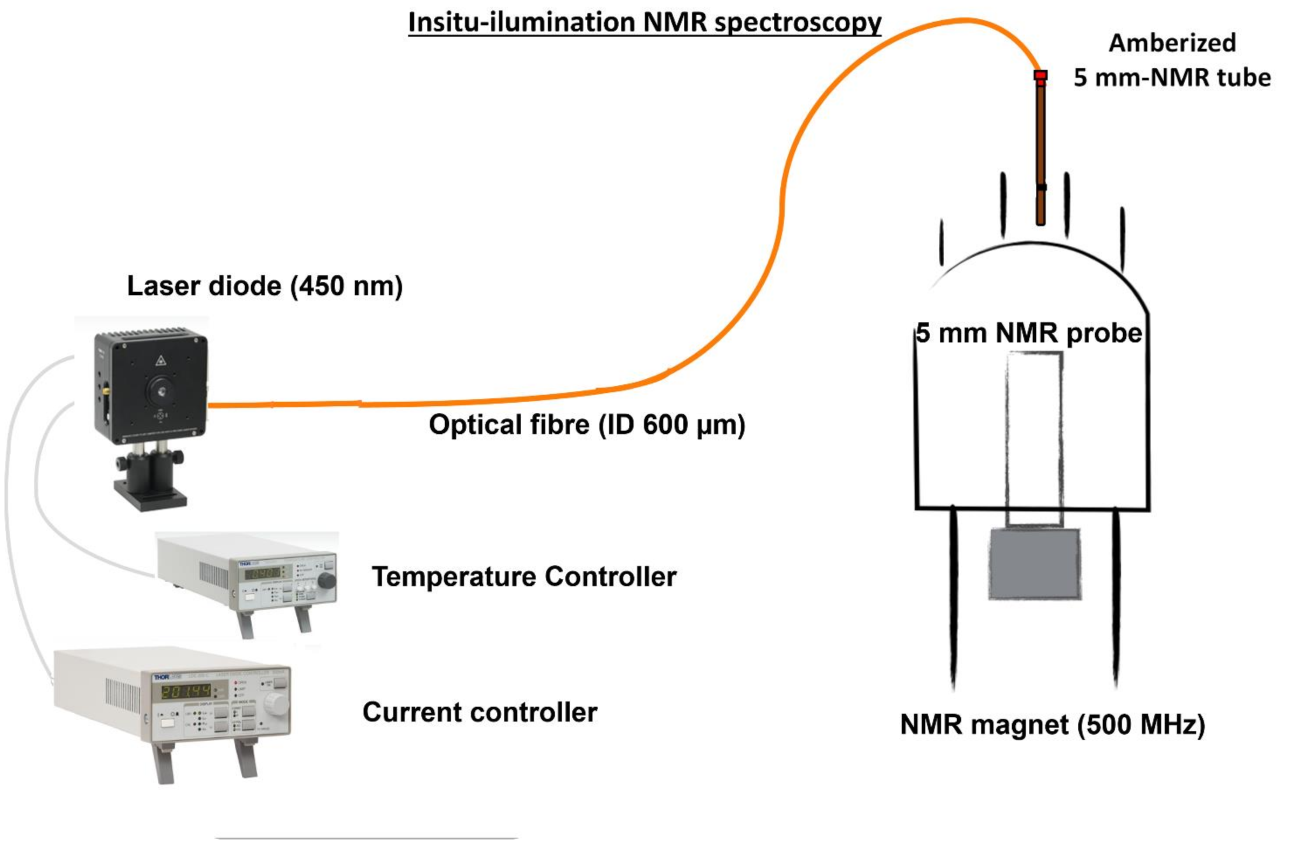

Figure S1: Experimental setup for the *in-situ* measurements. An amberized 5 mm-NMR tube that contains the reaction mixture is placed inside the NMR magnet. An optical fiber guides the light from an external laser diode (at 450 nm) towards the 5 mm NMR tube. The current controller is optimized at 750 mA which provides 525 mW optical power out of the fiber. The temperature controller assures a constant optical power during the execution of the whole experiment. The temperature control unit of the NMR spectrometer was used for temperature control of the different values (from -40 °C to RT).

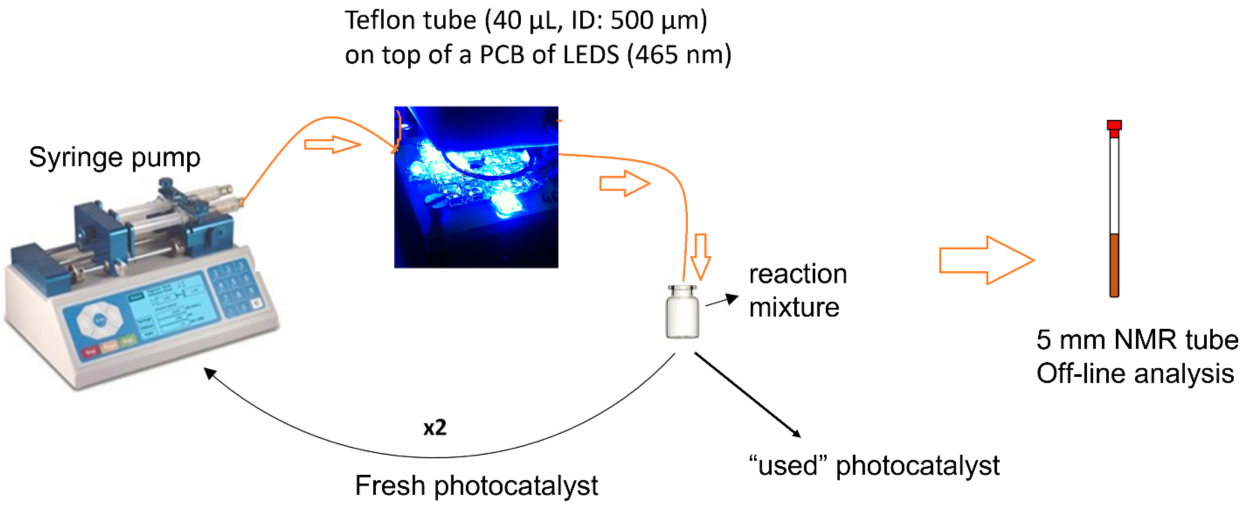

Figure S2: Experimental setup for the measurement of flow reactions. The free oxazolone is pumped by means of a syringe pump working at 1.33 µL / min, towards a custom-made microreactor (Teflon coil that contains 40 µl volume) irradiated by a PCB of LEDs at 465 nm. It results in a 30 min. residence time. After the first cycle of 30 min., the photocatalyst is removed and "fresh" catalyst is added for the second cycle (30 min.). The reaction mixture is collected and transferred to an NMR tube for its off-line analysis.

Table S1. Comparison of Space Time Yields[a] of oxazolones (*Z*)-**1** to give cyclobutane-bis(oxazolone)s **2** obtained using batch and continuous-flow methodologies.

	STY (flow)	STY (batch)
**2a**	20.18	0.89
**2b**	20.18	0.79
**2c**	17.50	0.99
**2d**	22.87	0.84
**2e**	21.52	0.86
**2f**	18.83	0.33
**2g**	20.99	0.90
**2h**	-	0.82
**2i**	15.88	0.87
**2j**	21.26	0.86

[a] STY = mol product / Kg catalyst * h

NMR spectra of all prepared compounds

1.- NMR spectra of all starting (*Z*)-4-arylidene(or -allylidene)-2-phenyl-5(4*H*)-oxazolones 1a-1j

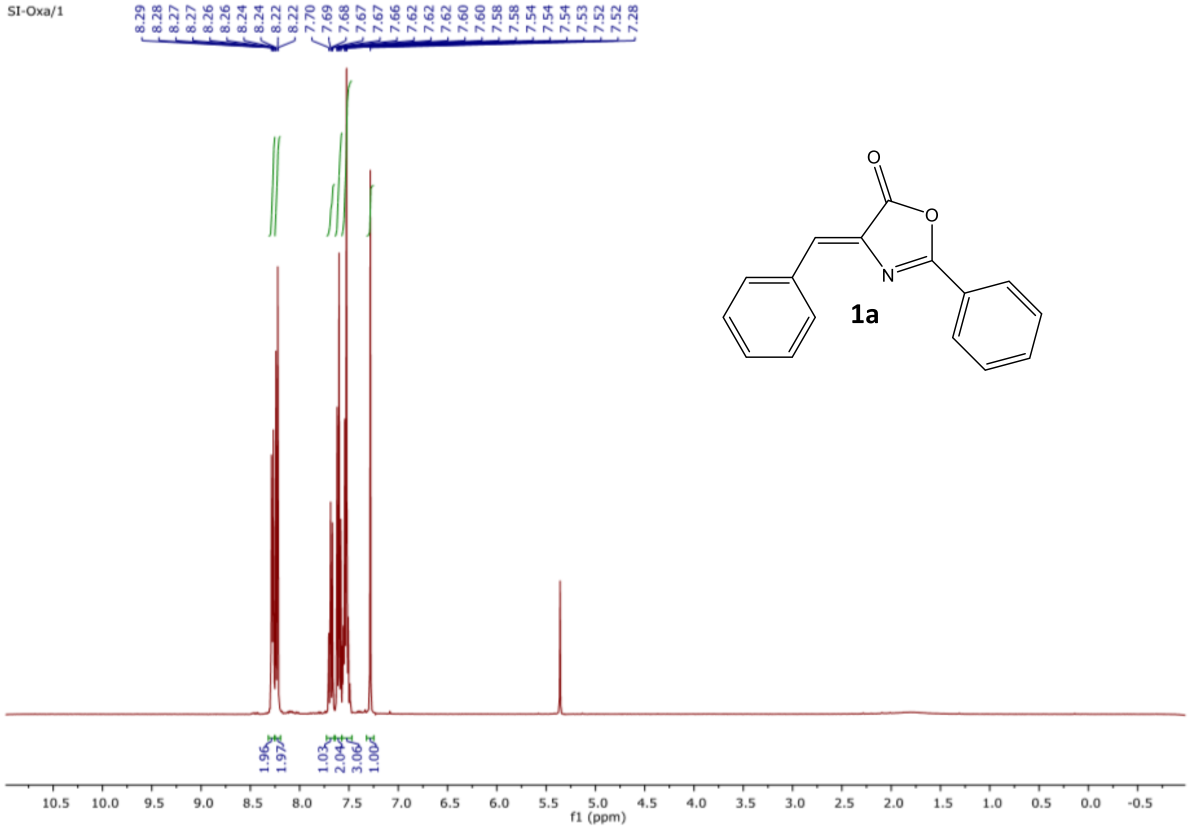

^{1}H NMR (CD_2Cl_2, 300.13 MHz) spectrum of oxazolone **1a**

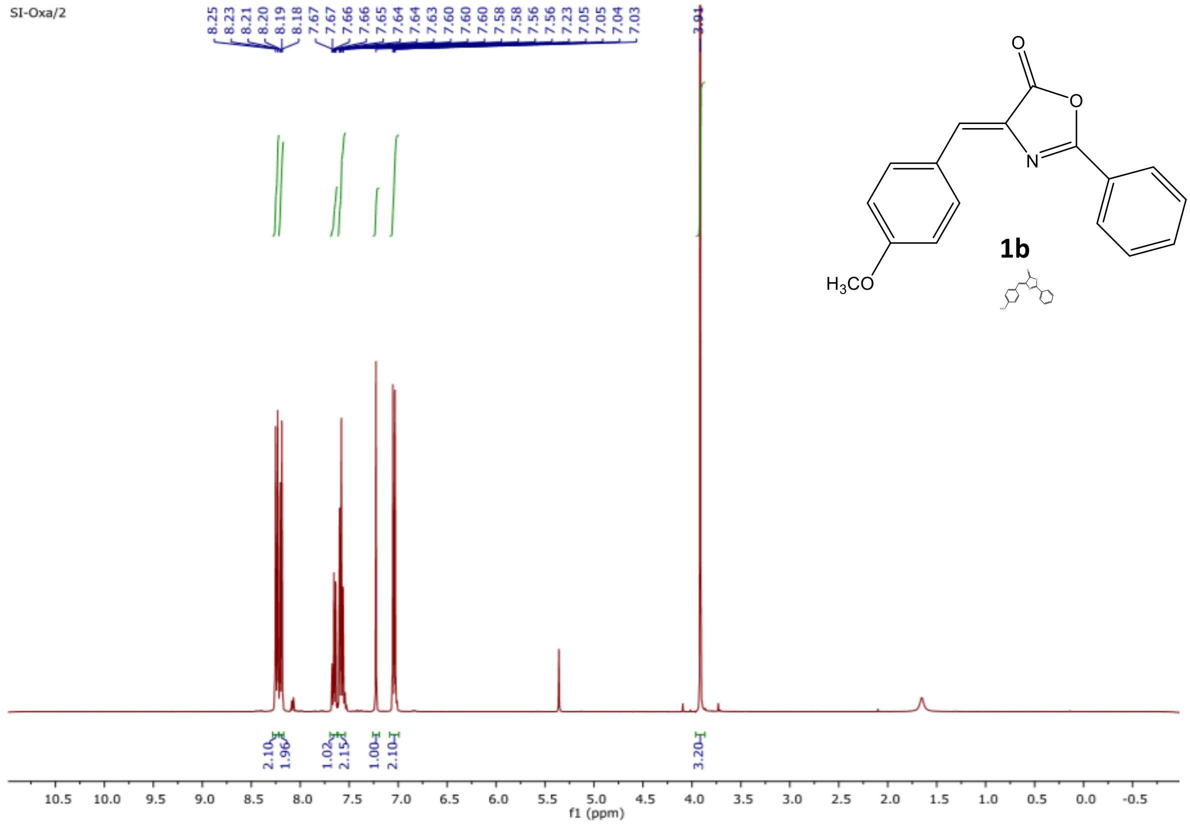

^{1}H NMR (CD_2Cl_2, 300.13 MHz) spectrum of oxazolone **1b**

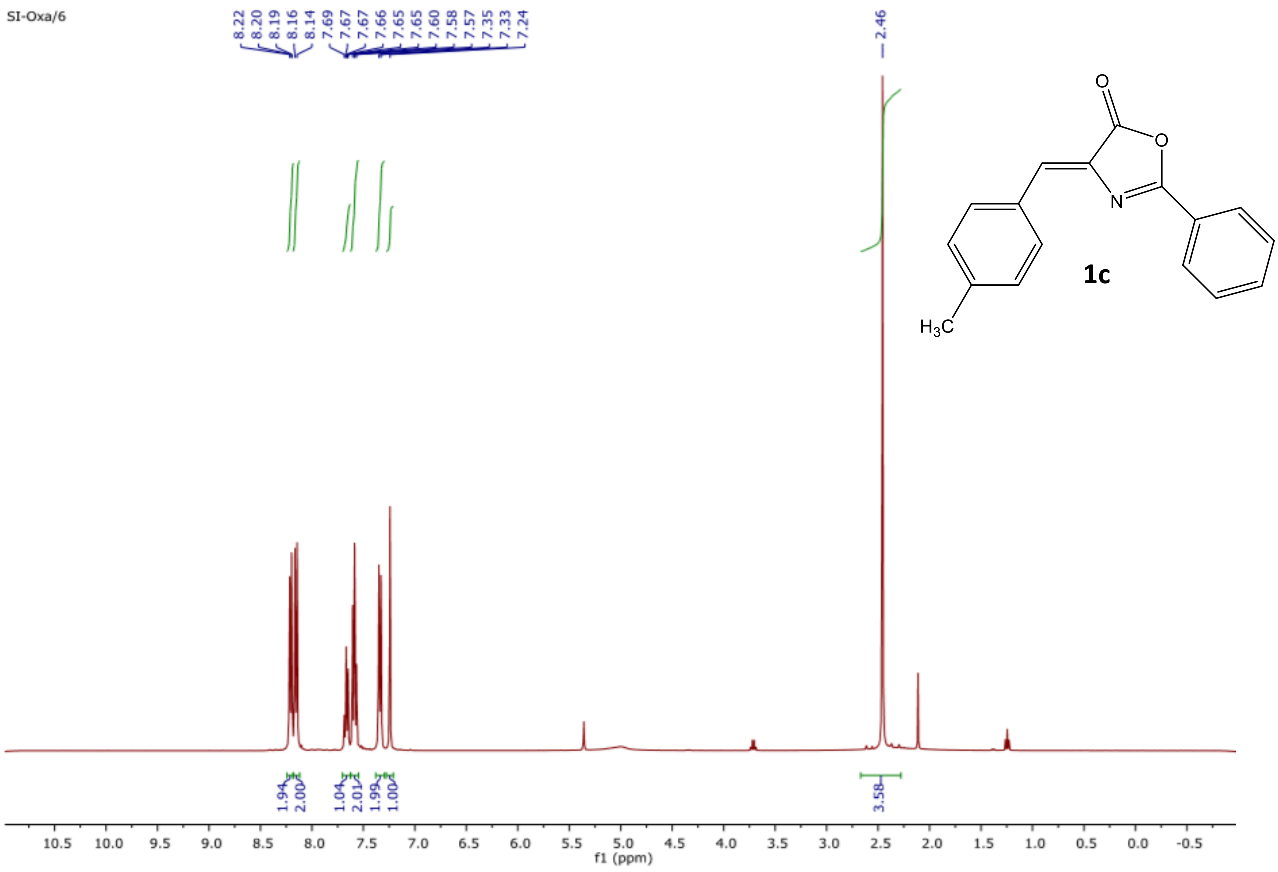

^{1}H NMR (CD_2Cl_2, 300.13 MHz) spectrum of oxazolone **1c**

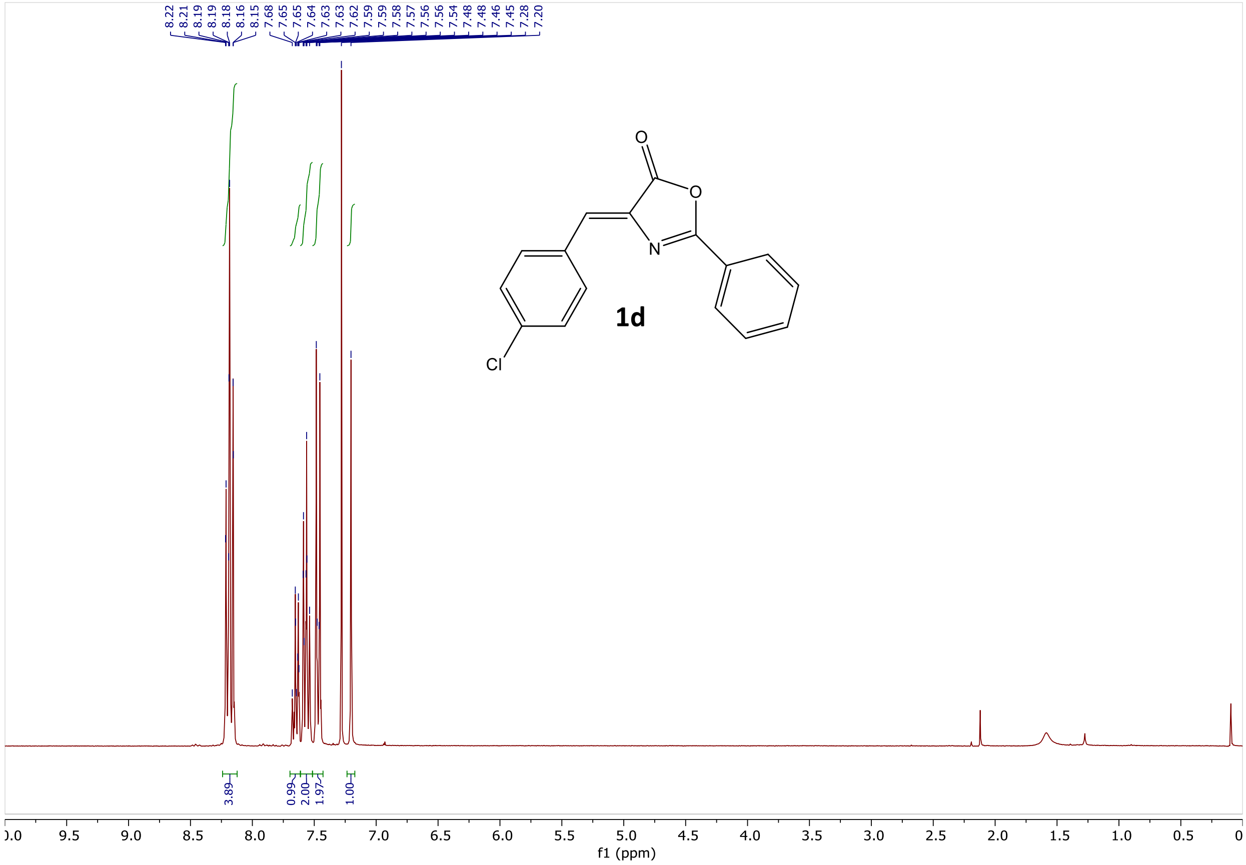

^{1}H NMR ($CDCl_3$, 300.13 MHz) spectrum of oxazolone **1d**

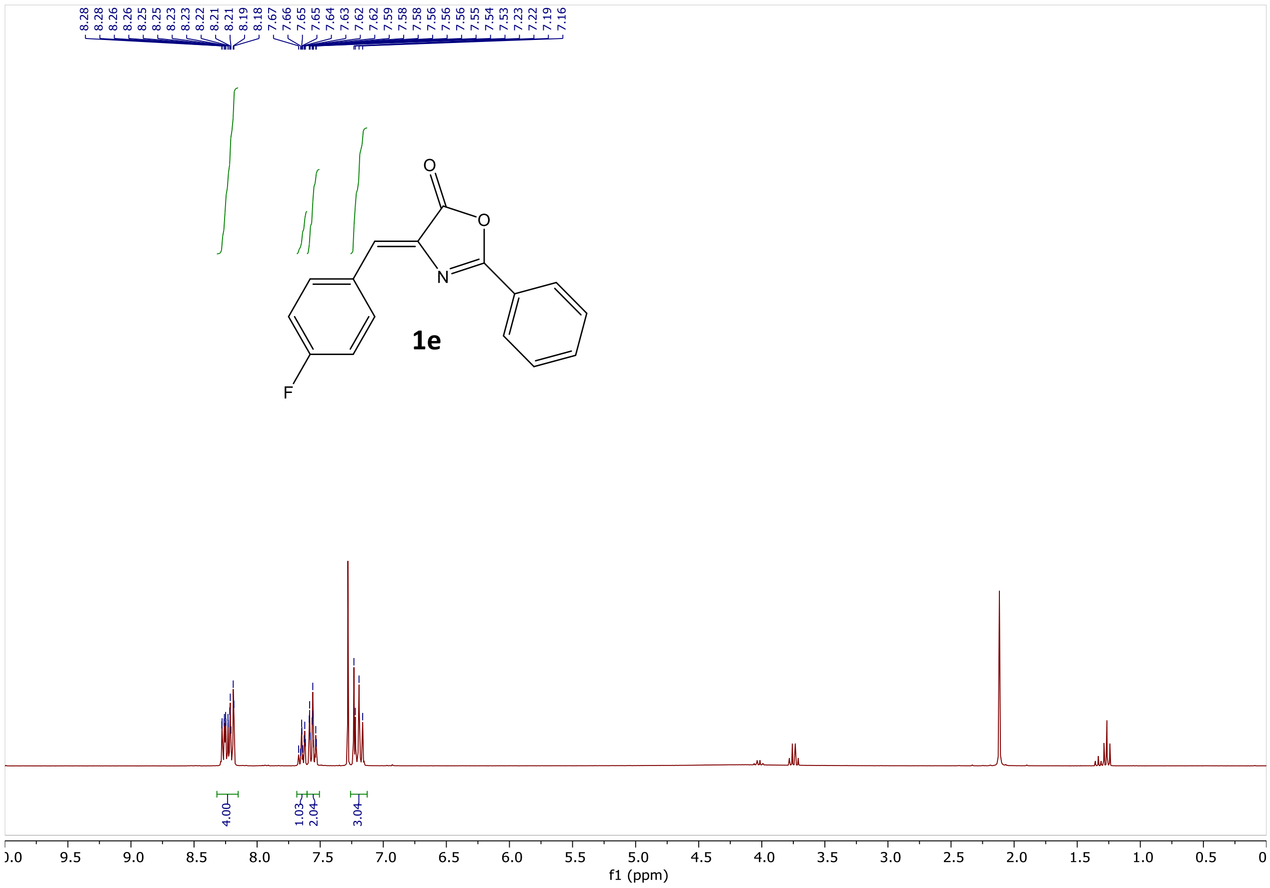

^{1}H NMR ($CDCl_3$, 300.13 MHz) spectrum of oxazolone **1e**

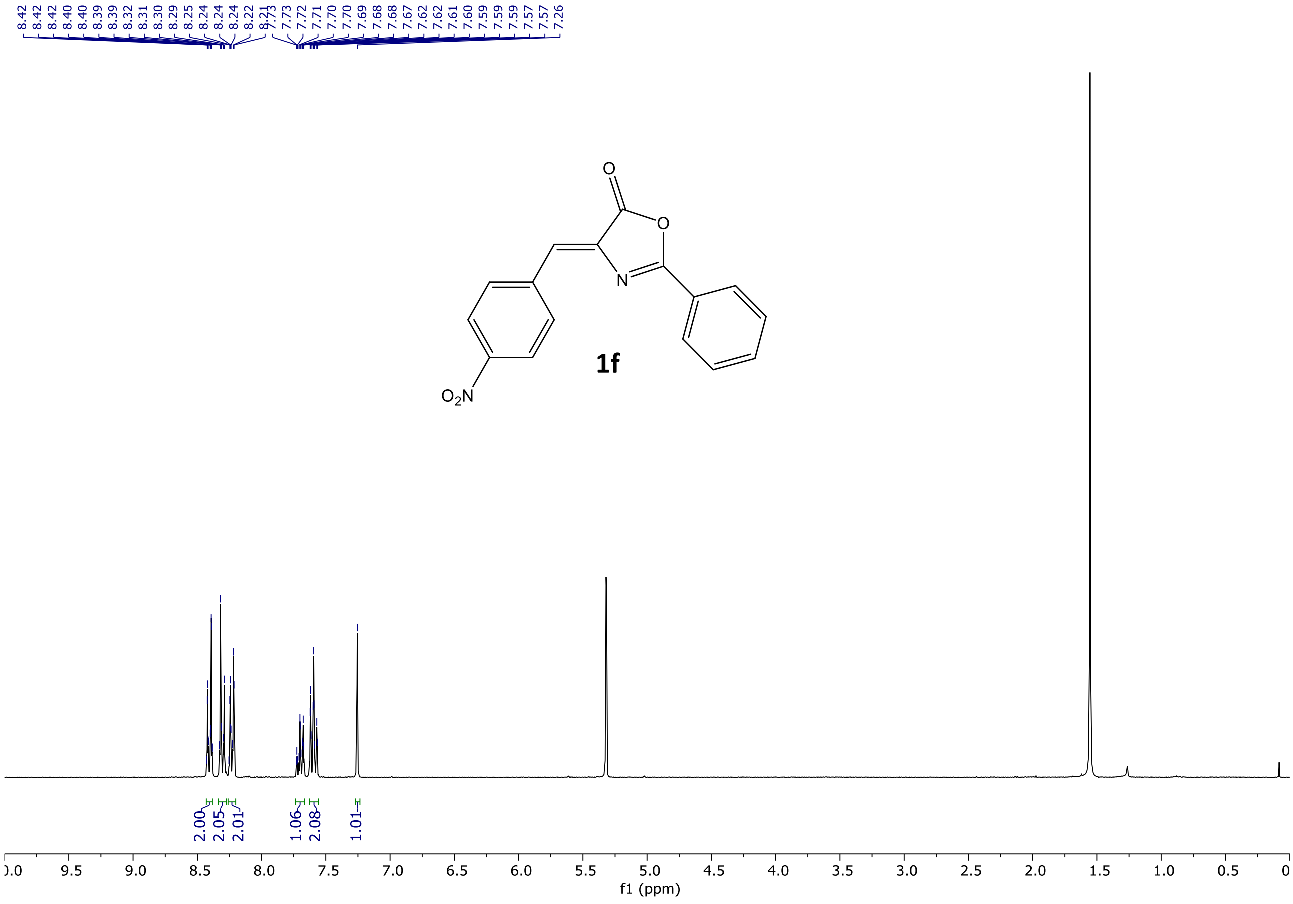

^{1}H NMR (CD_2Cl_2, 300.13 MHz) spectrum of oxazolone **1f**

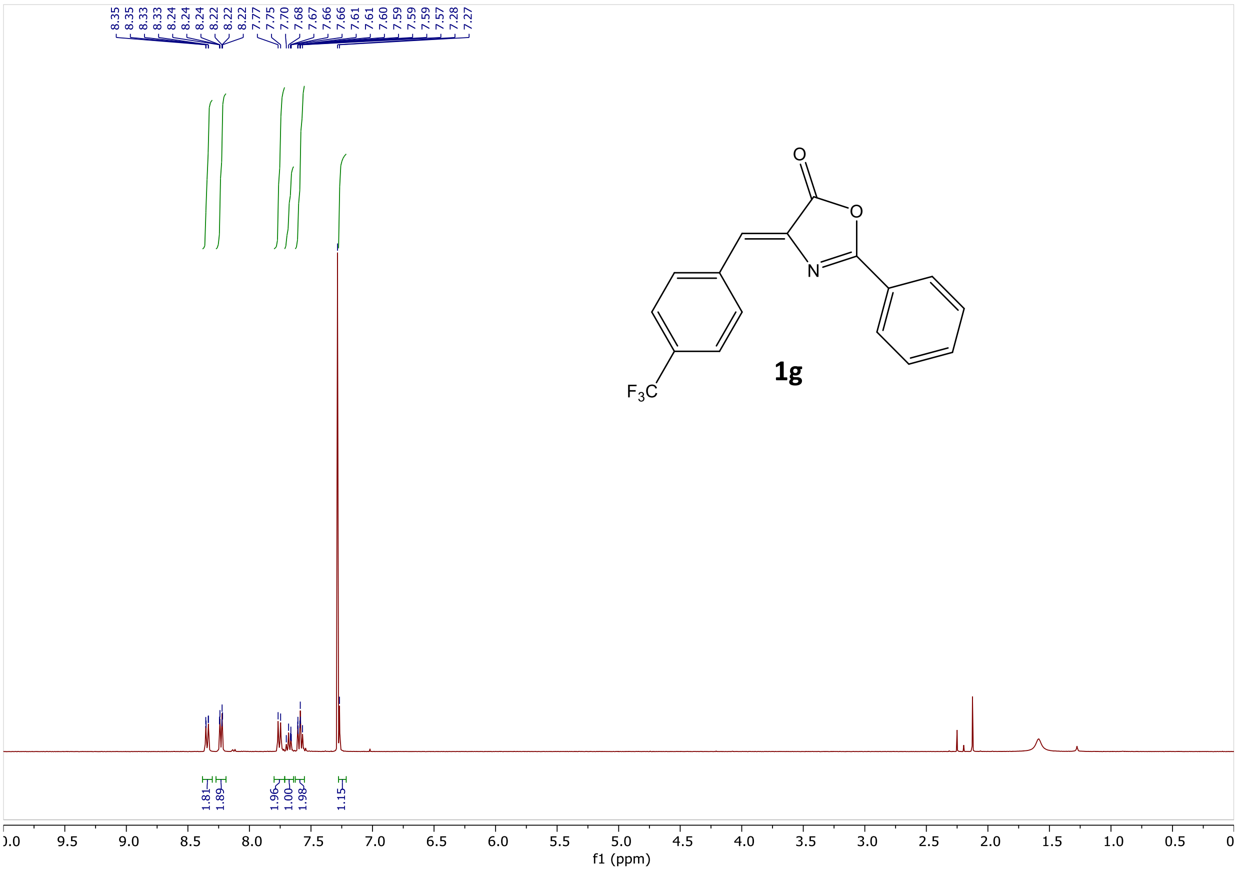

^{1}H NMR ($CDCl_3$, 300.13 MHz) spectrum of oxazolone **1g**

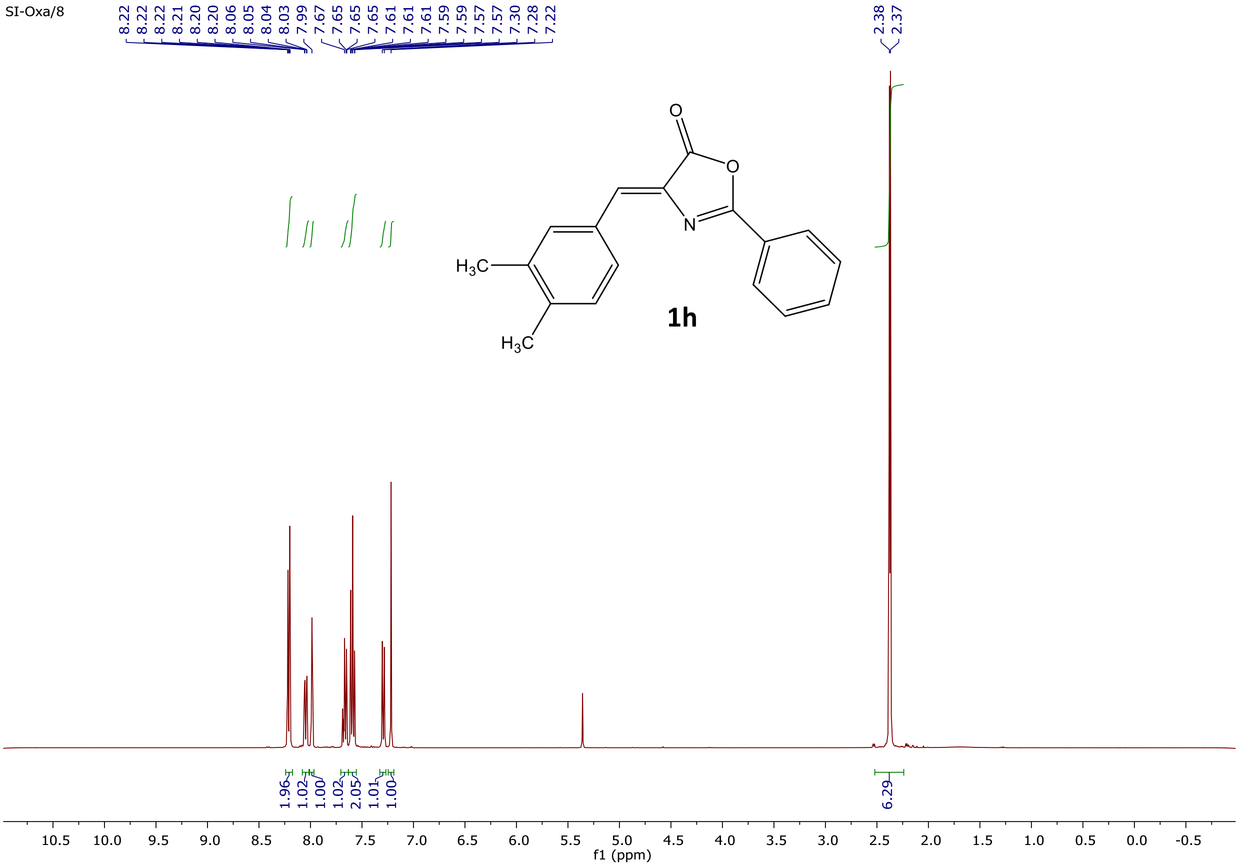

^{1}H NMR (CD_2Cl_2, 300.13 MHz) spectrum of oxazolone **1h**

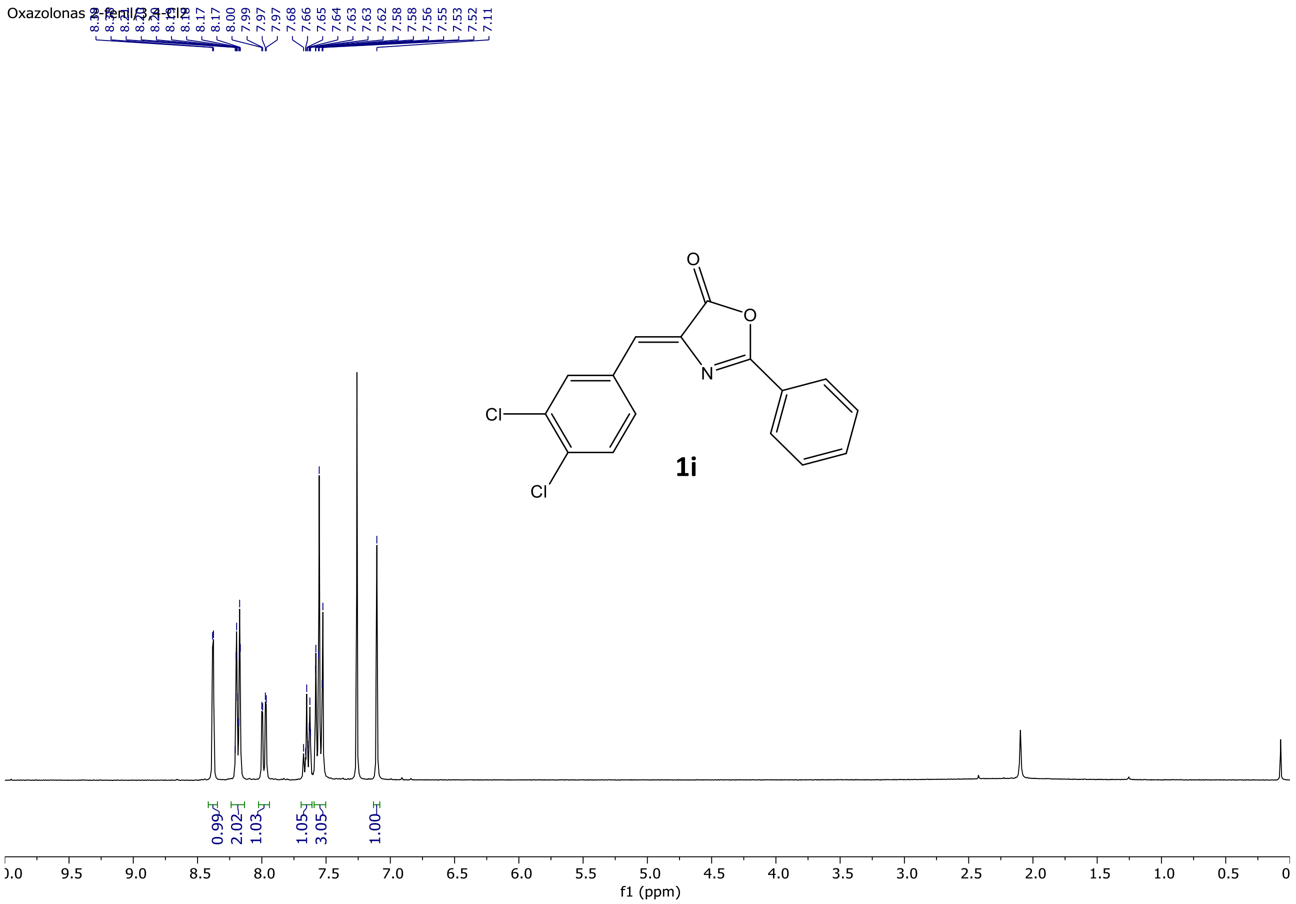

^1H NMR ($CDCl_3$, 300.13 MHz) spectrum of oxazolone **1i**

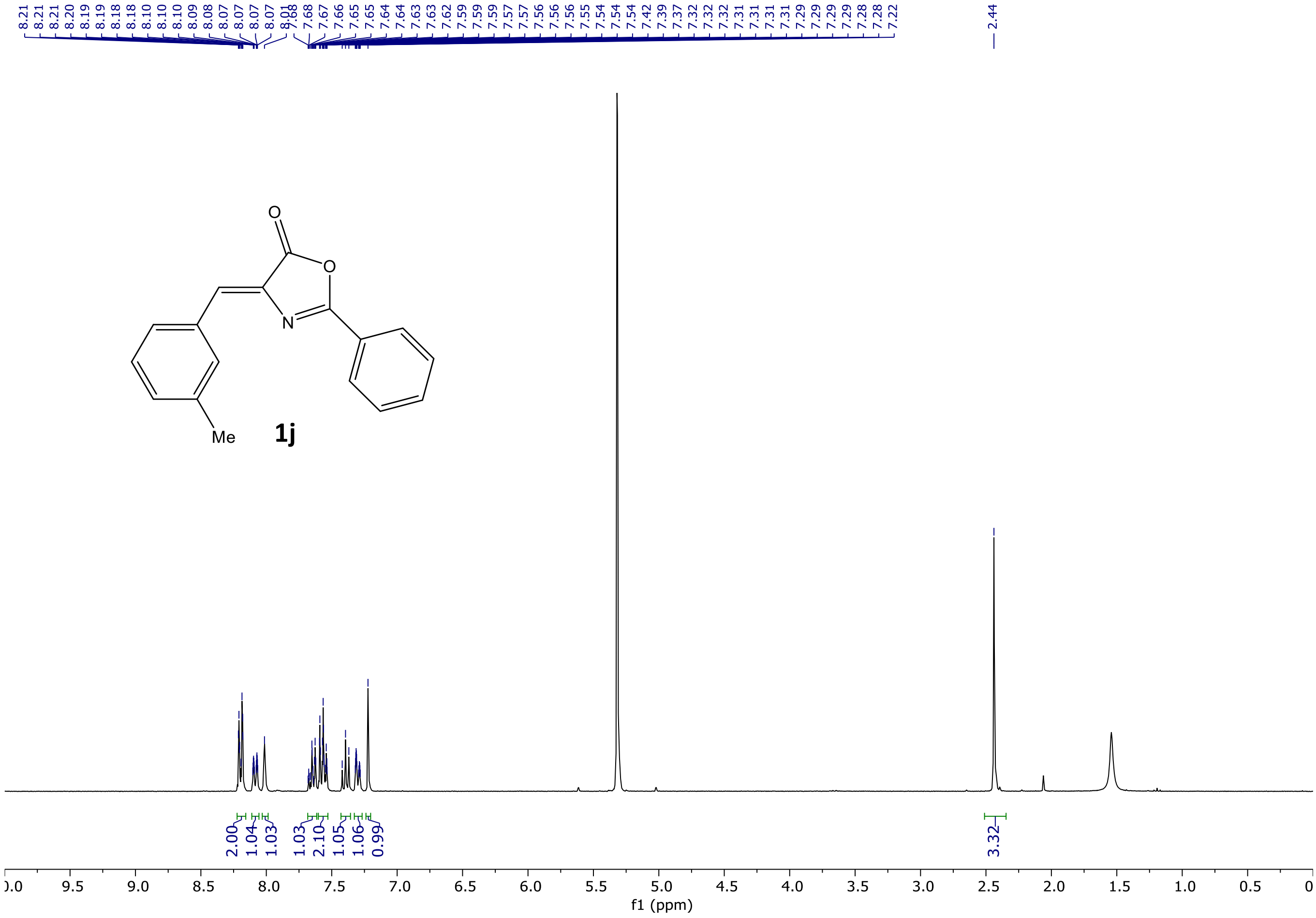

^1H NMR (CD_2Cl_2, 300.13 MHz) spectrum of oxazolone **1j**

2.- NMR spectra of the cyclobutane-bis(oxazolone) intermediates 2a-2h (except 2f, see main text)

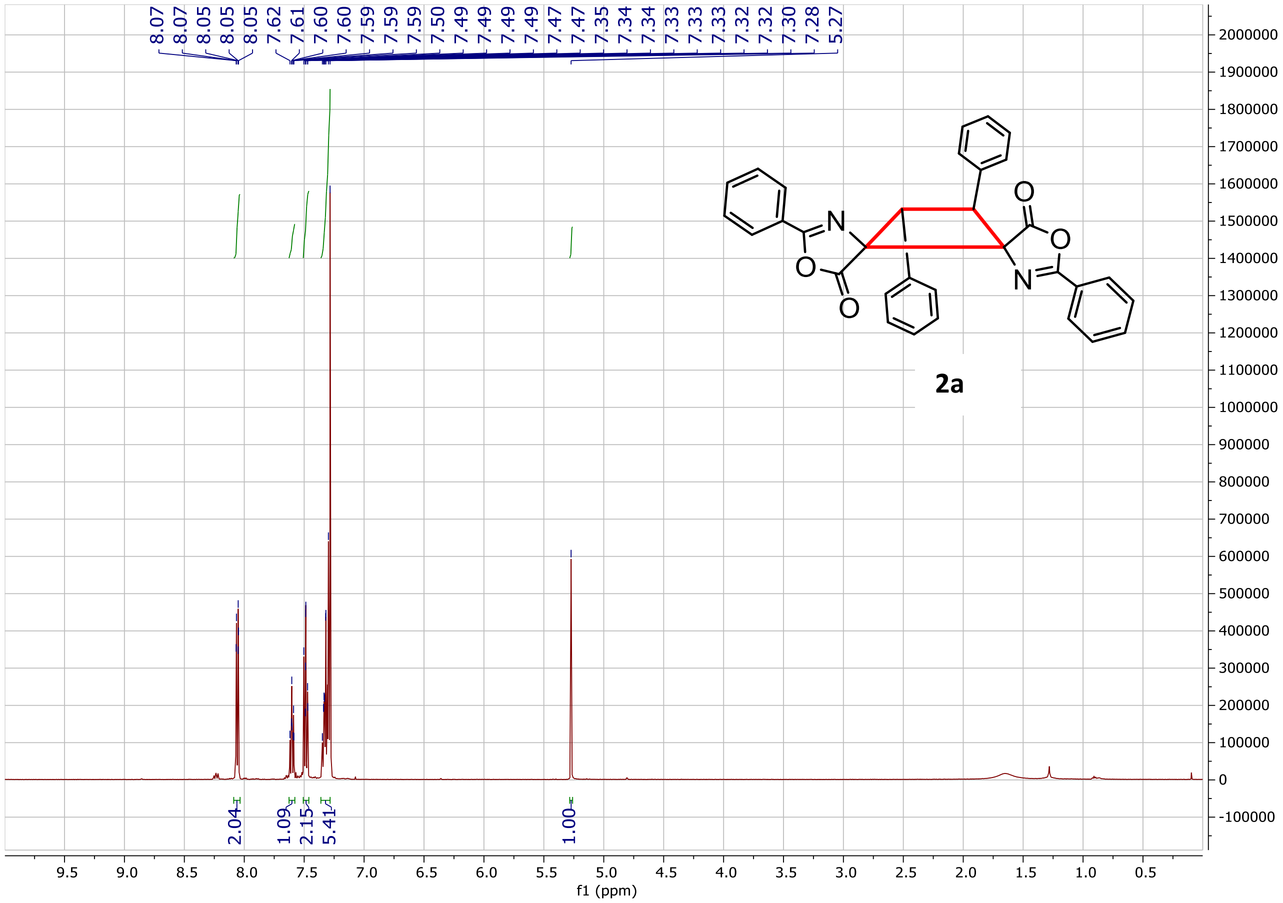

^{1}H NMR ($CDCl_3$, 500.13 MHz) of **2a**

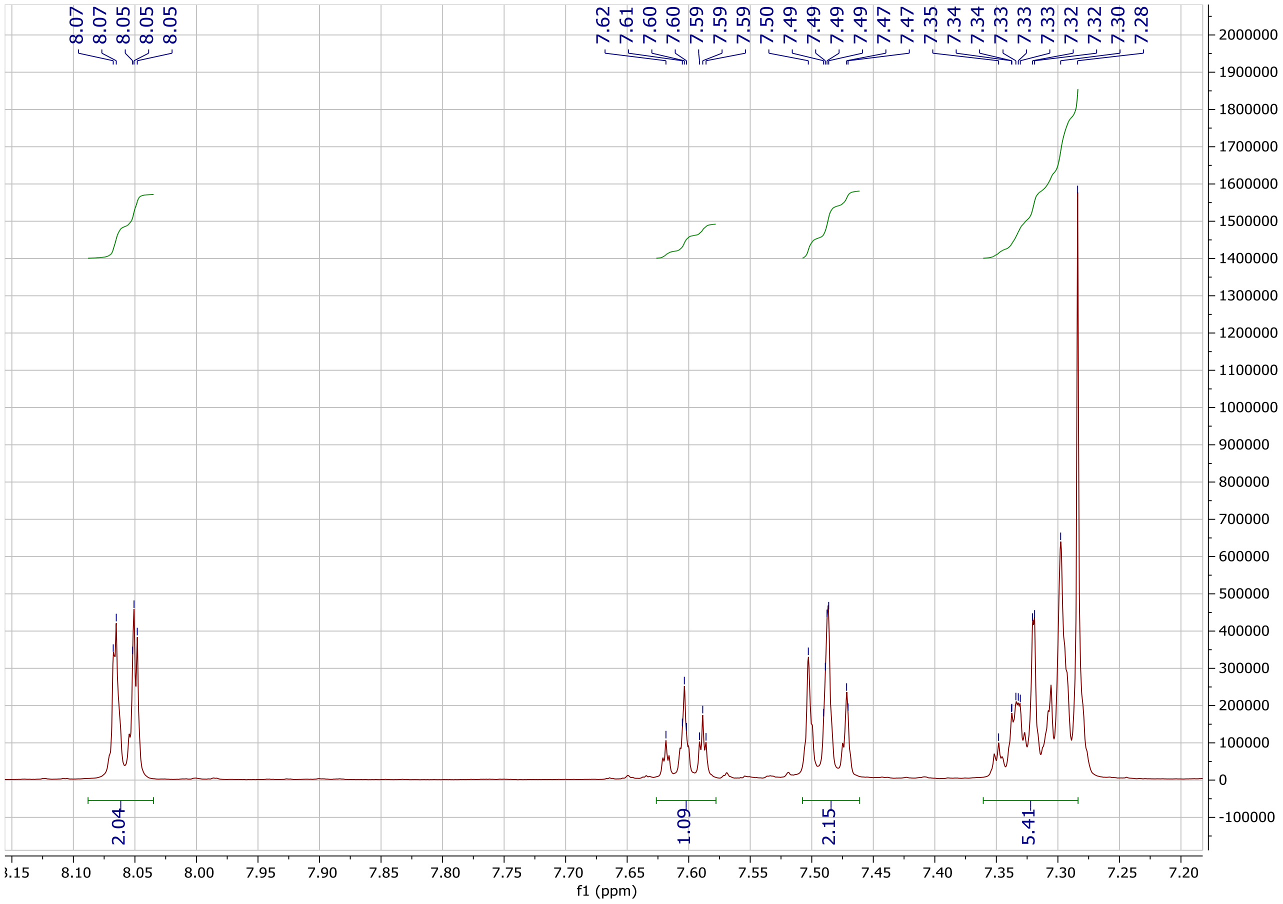

^{1}H NMR of **2a** (zoom aromatic region)

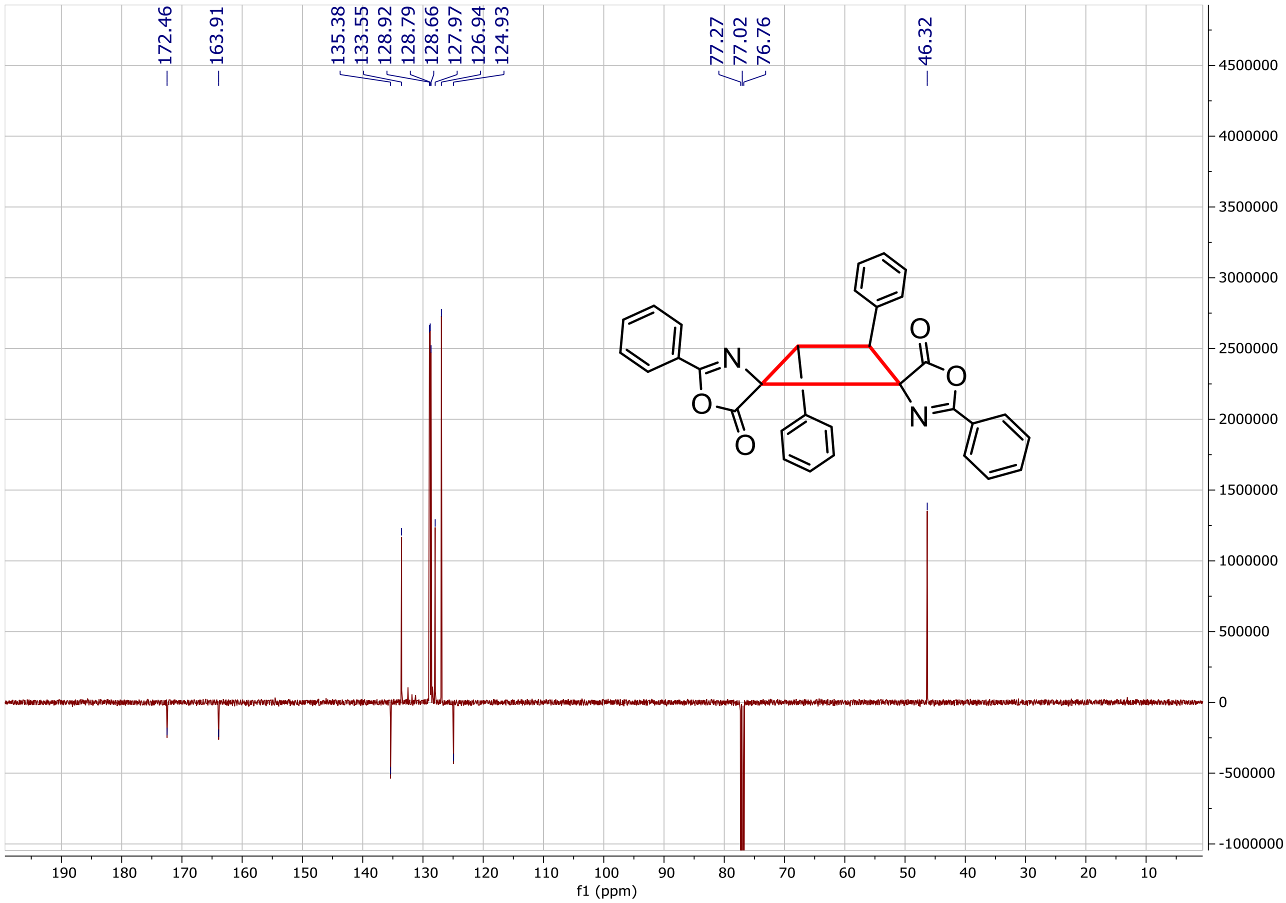

$^{13}C\{^{1}H\}$ (APT) NMR ($CDCl_3$, 125.76 MHz) of **2a**

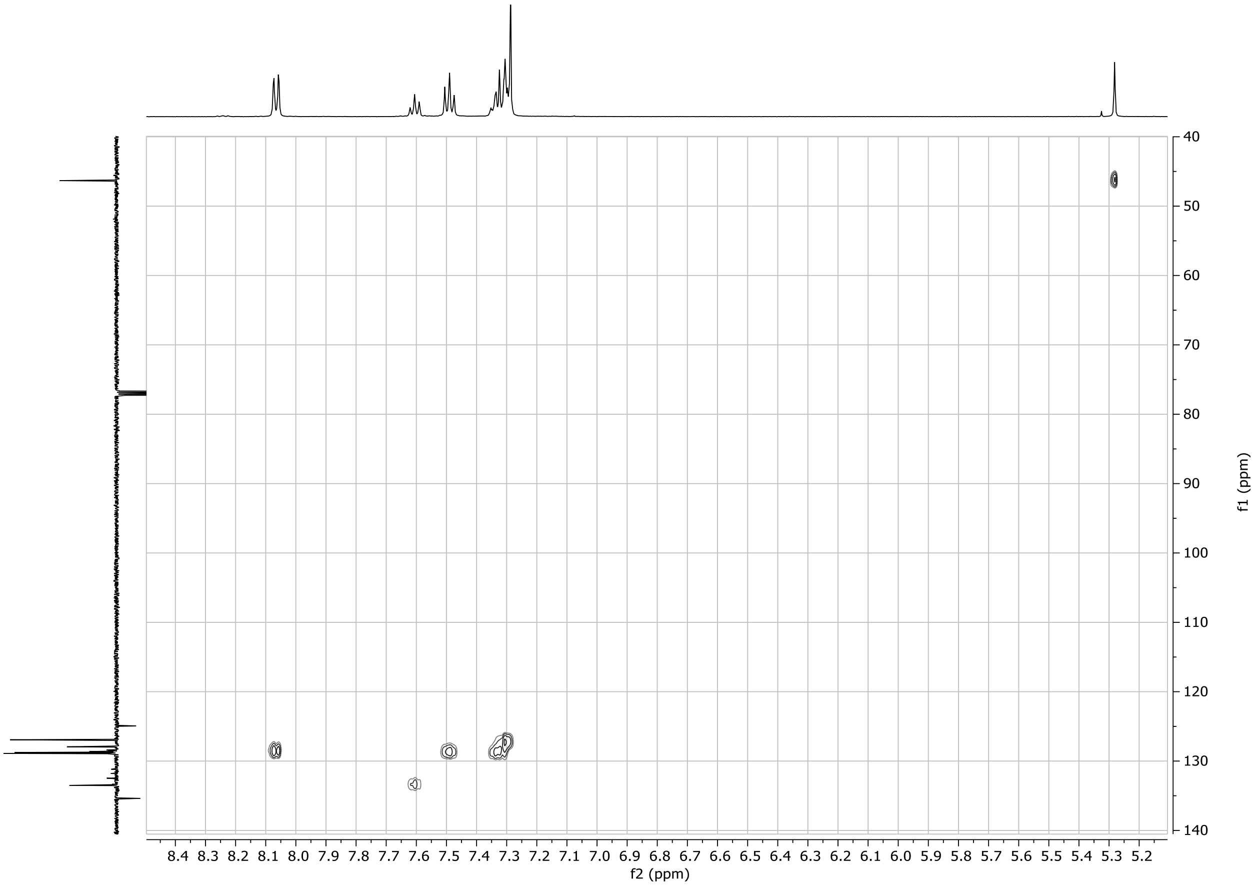

^{1}H-^{13}C HSQC correlation ($CDCl_3$) of **2a**

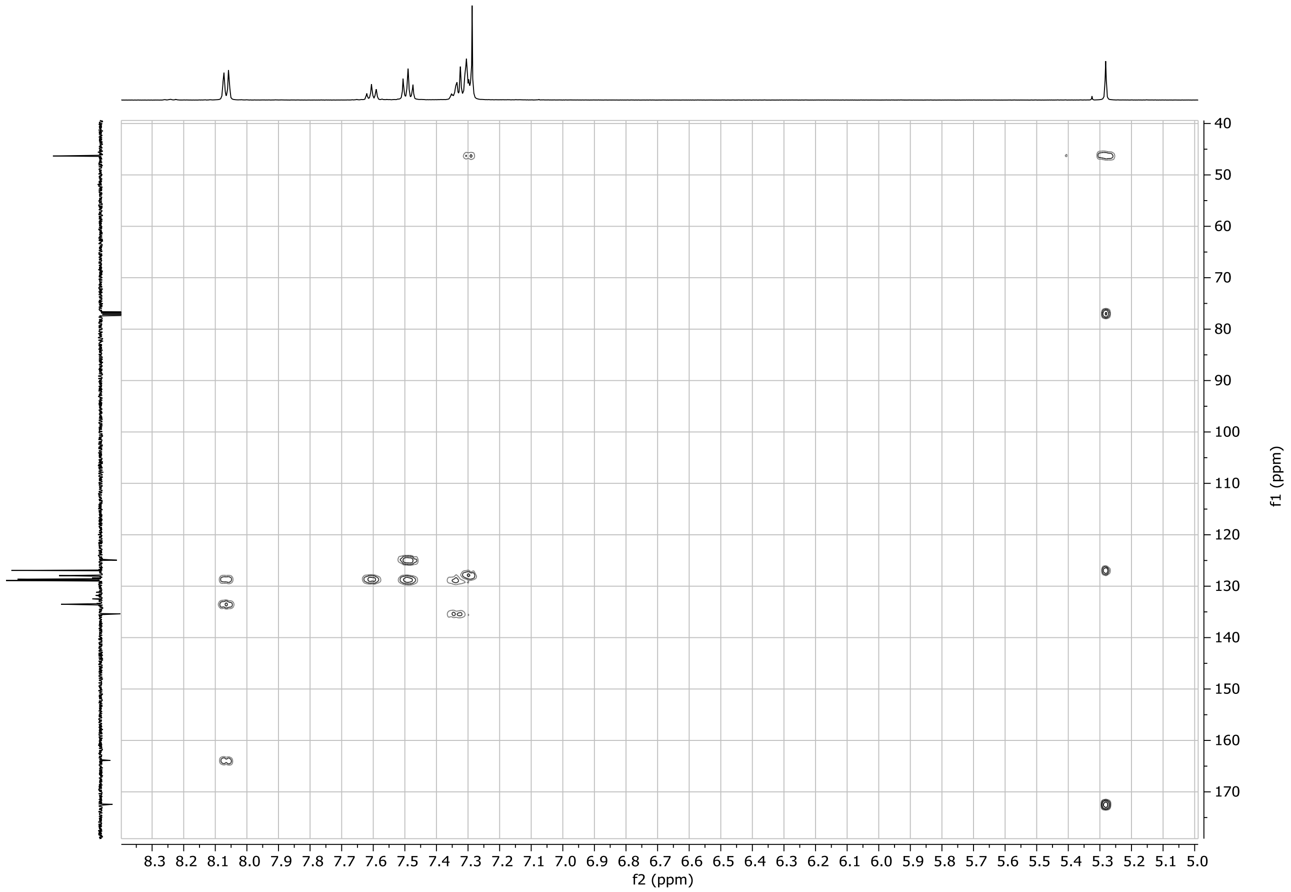

^{1}H-^{13}C HMBC correlation ($CDCl_3$) of **2a**

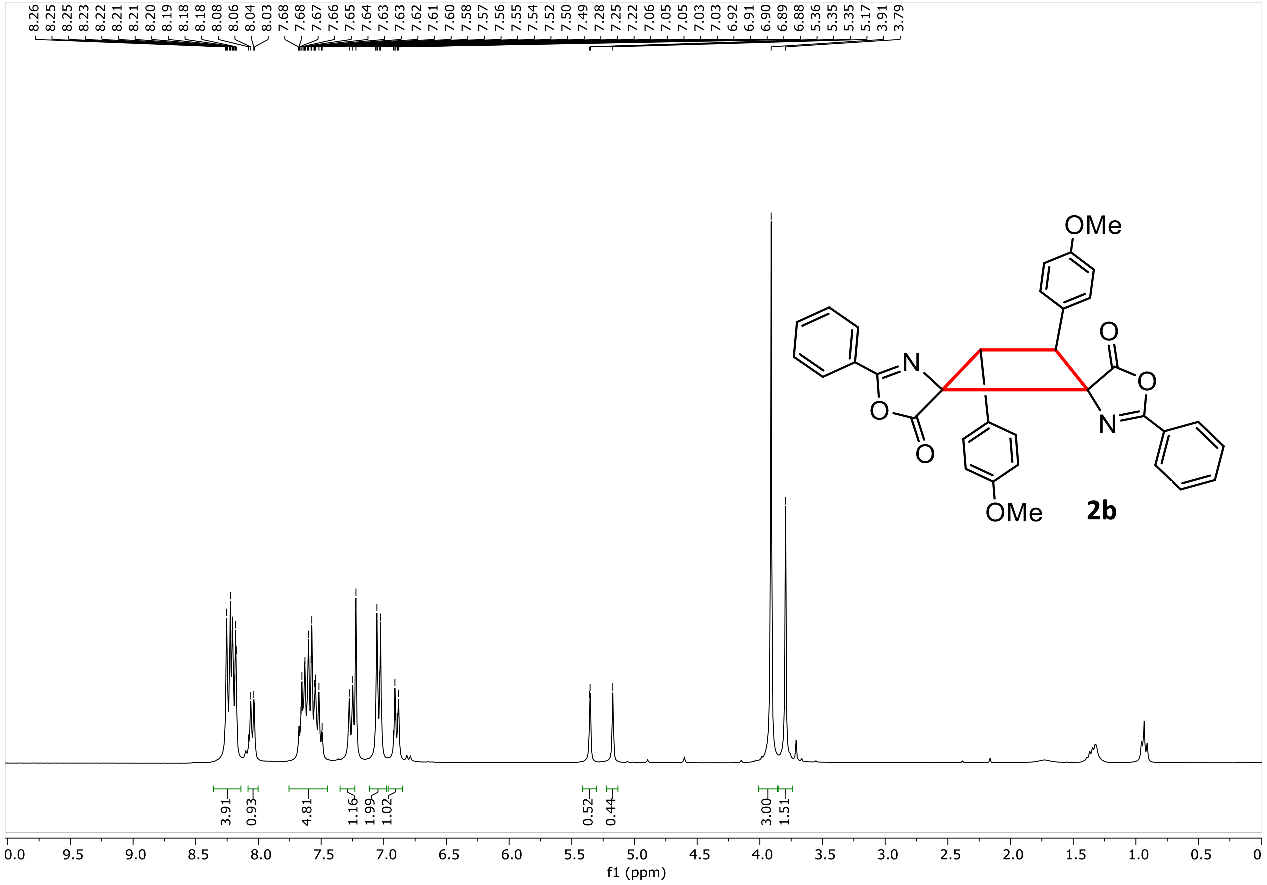

^{1}H NMR (CD_2Cl_2, 300.13 MHz) of **2b**

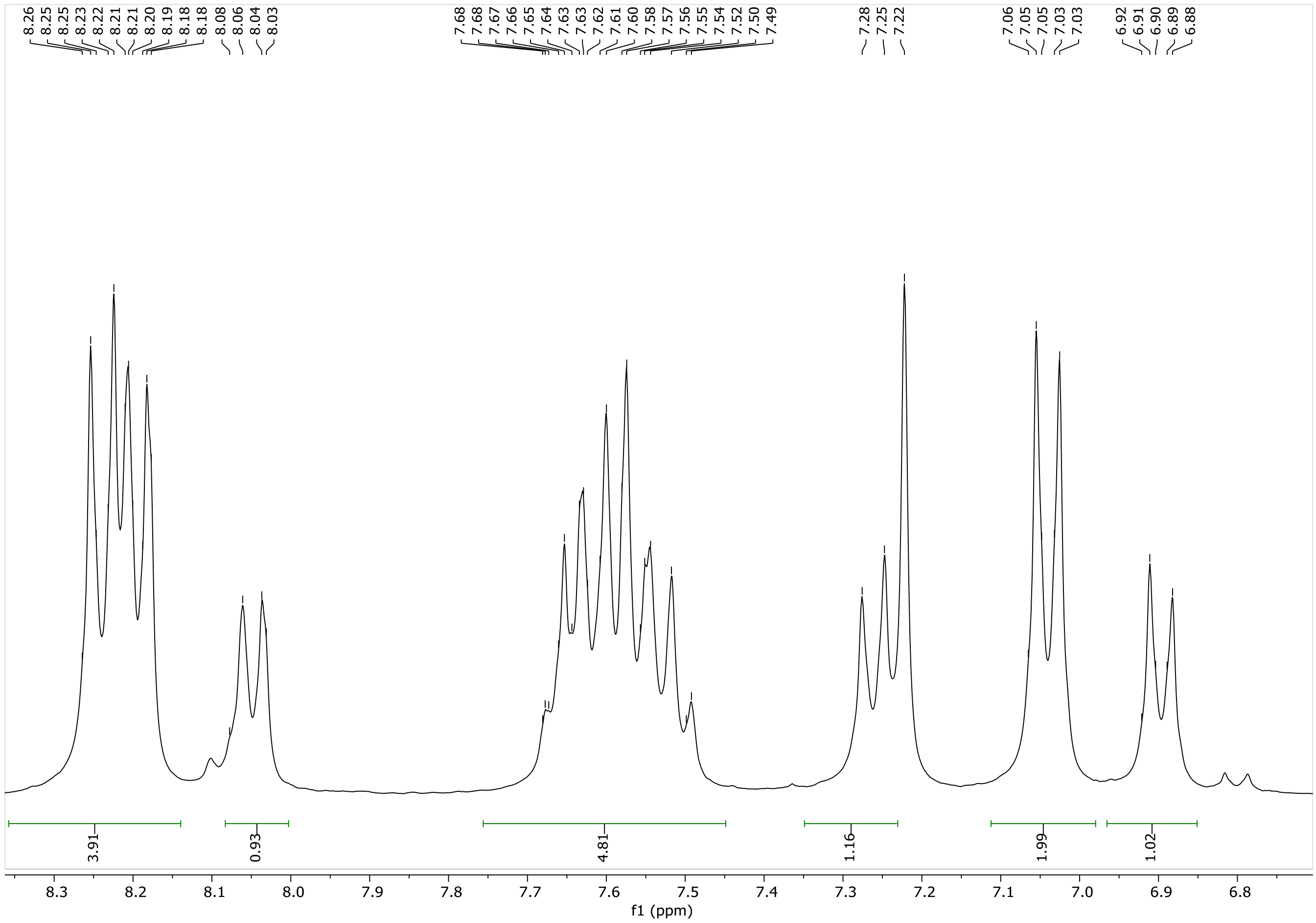

^{1}H NMR of **2b** (zoom aromatic region)

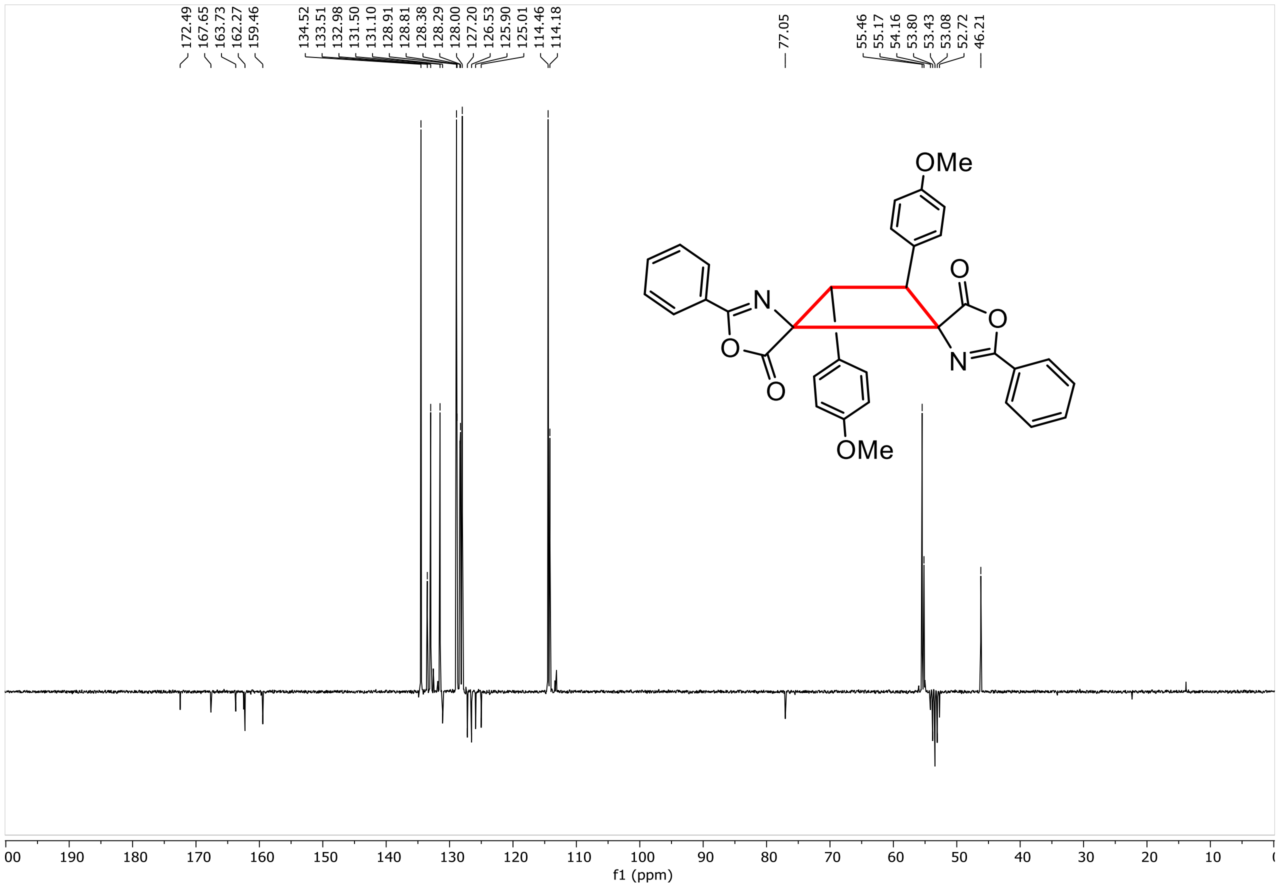

$^{13}C\{^{1}H\}$ (APT) NMR (CD_2Cl_2, 75.5 MHz) of **2b**

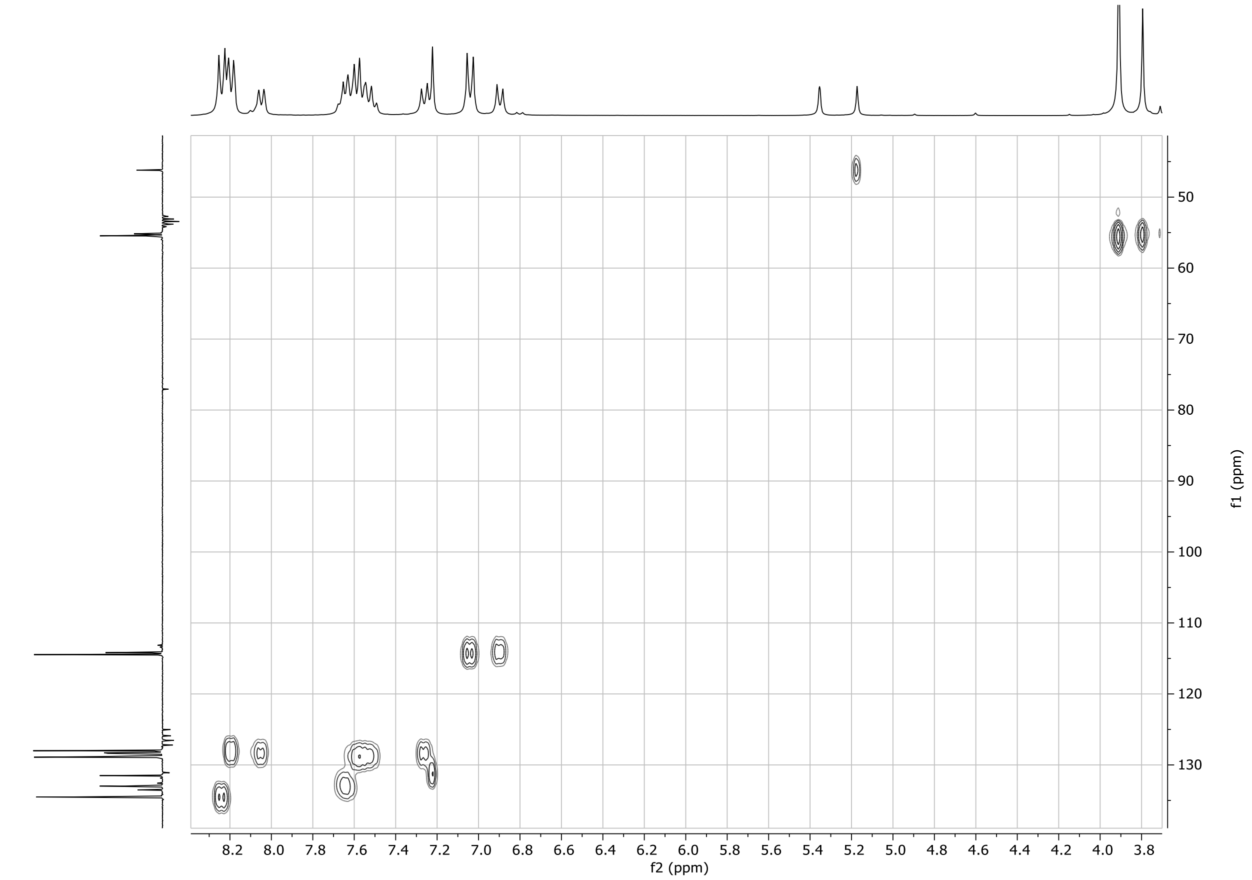

^1H-^{13}C HSQC correlation (CD_2Cl_2) of **2b**

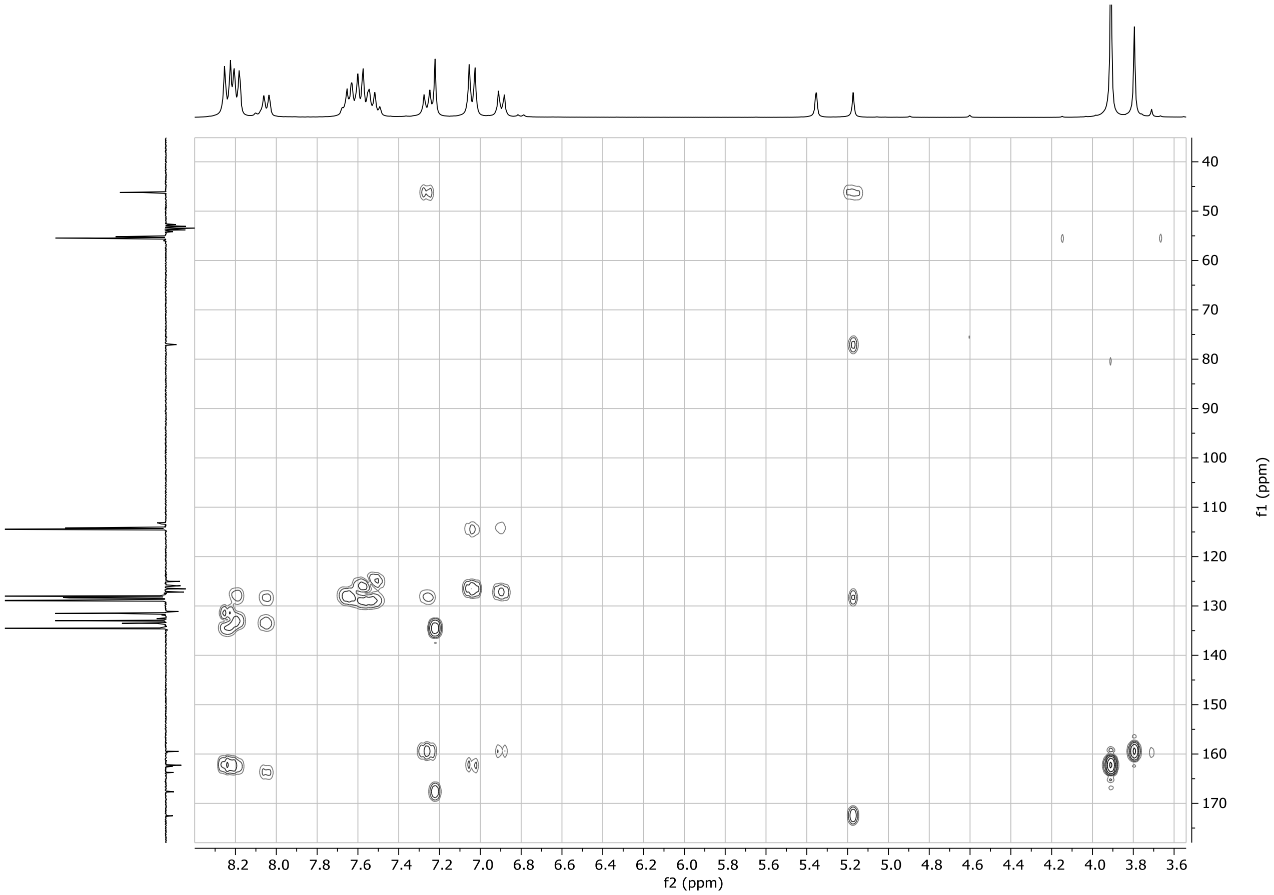

^1H-^{13}C HMBC correlation (CD_2Cl_2) of **2b**

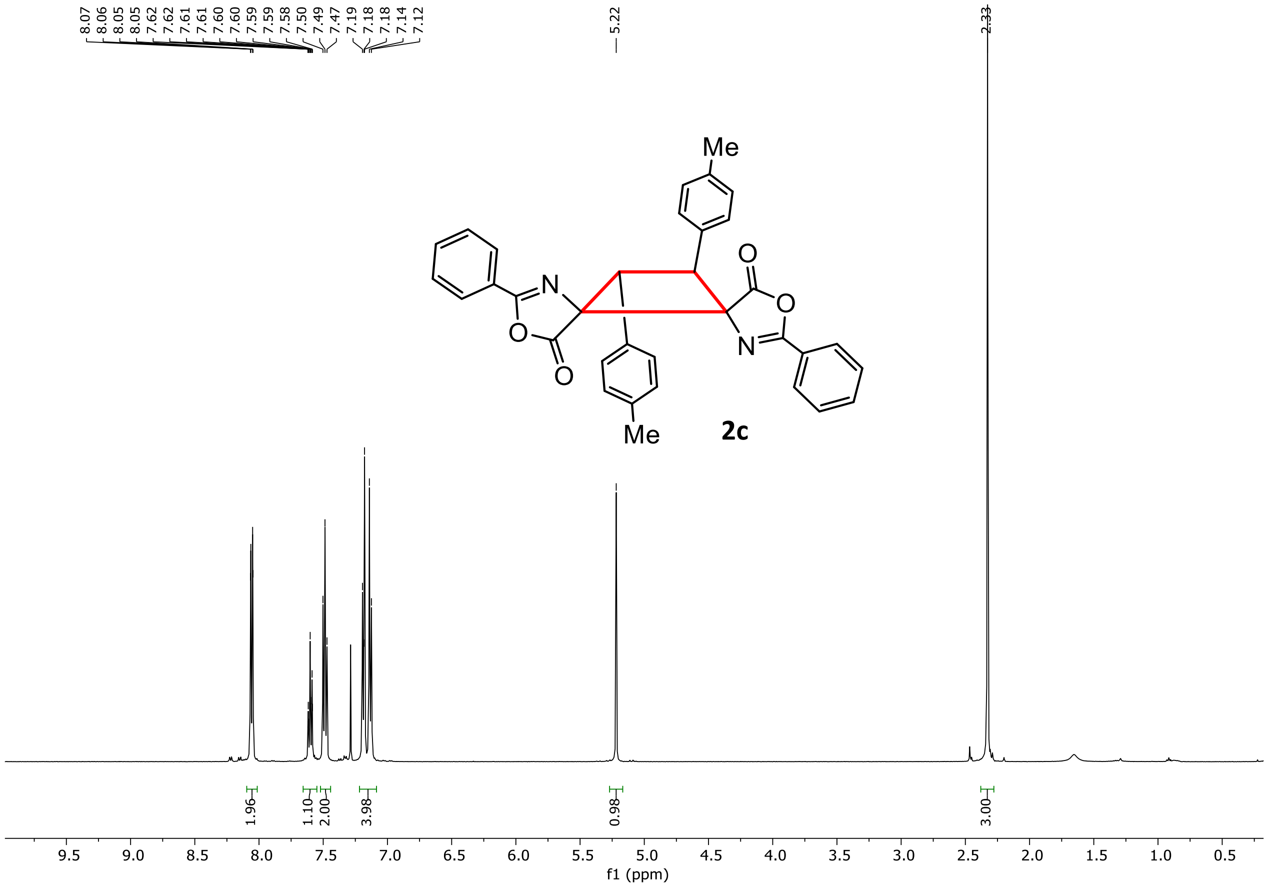

^{1}H NMR (CDCl$_3$, 300.13 MHz) of **2c**

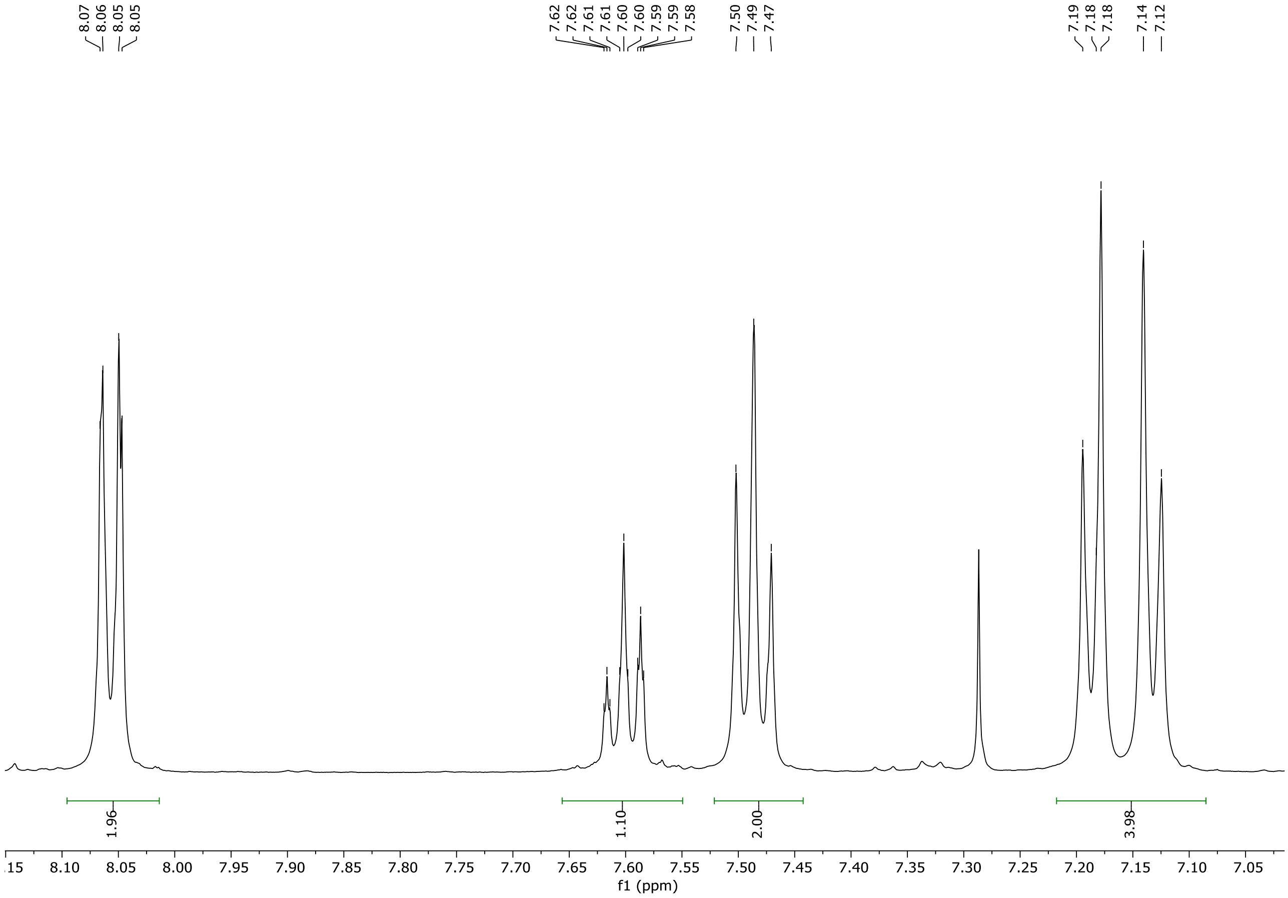

^{1}H NMR of **2c** (zoom aromatic region)

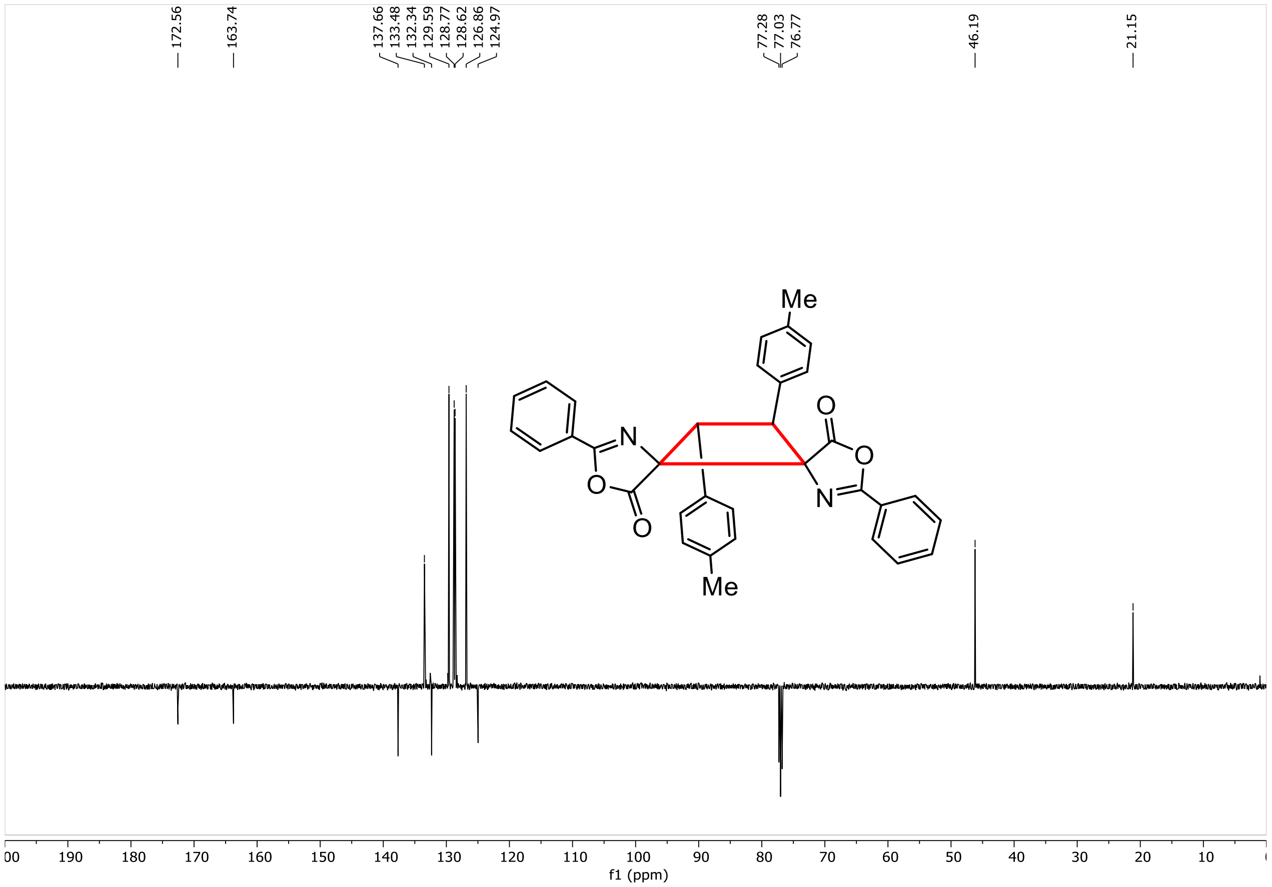

$^{13}C\{^{1}H\}$ (APT) NMR ($CDCl_3$, 75.5 MHz) of **2c**

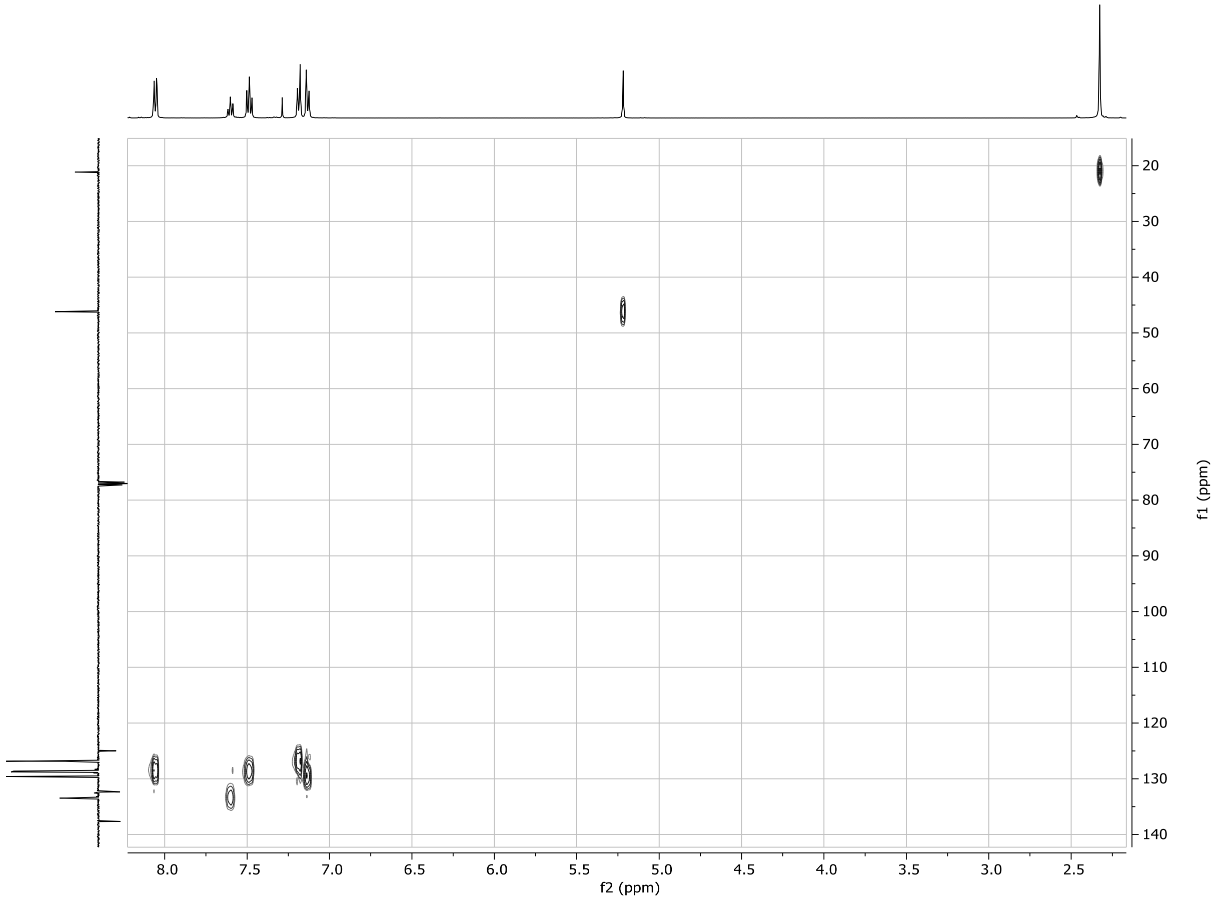

^{1}H-^{13}C HSQC correlation ($CDCl_3$) of **2c**

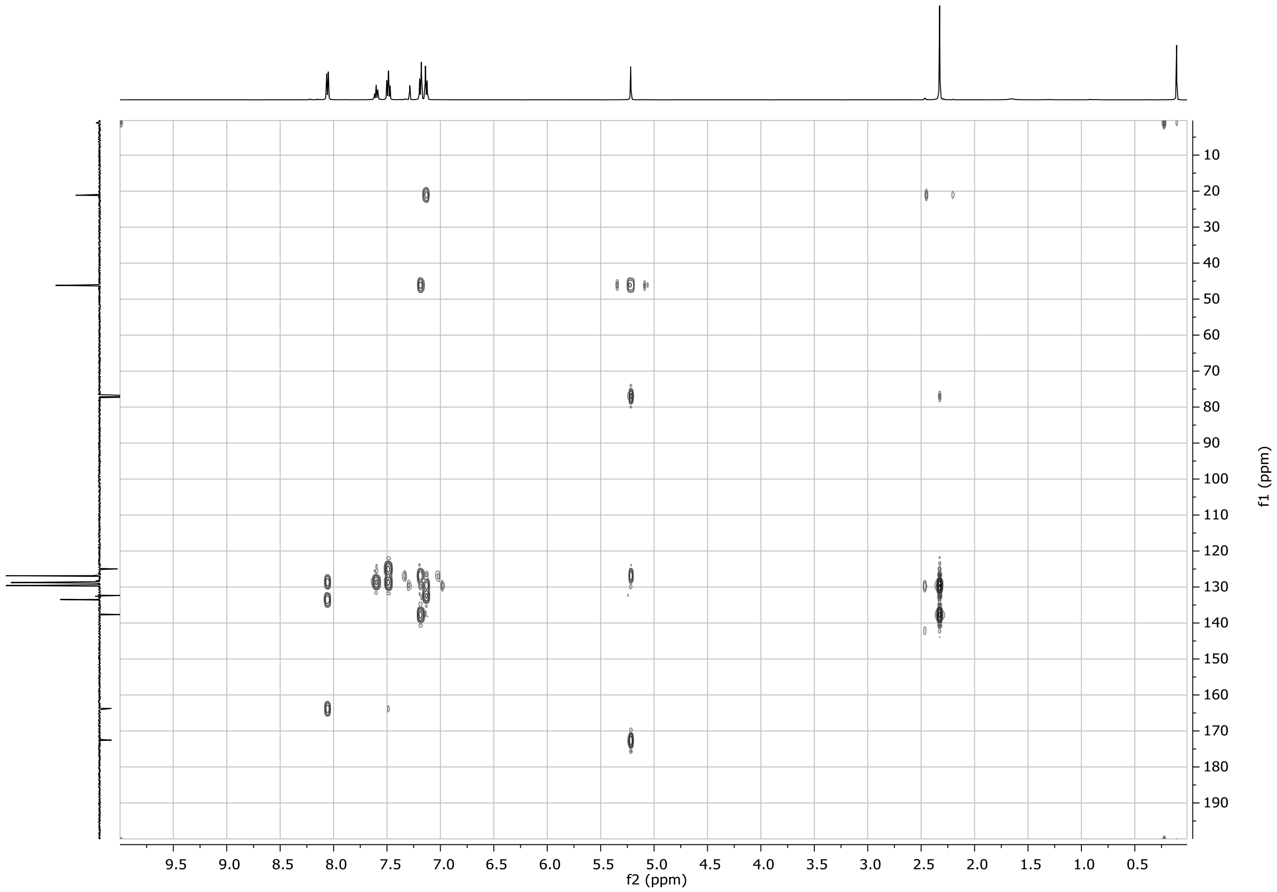

^{1}H-^{13}C HMBC correlation ($CDCl_3$) of **2c**

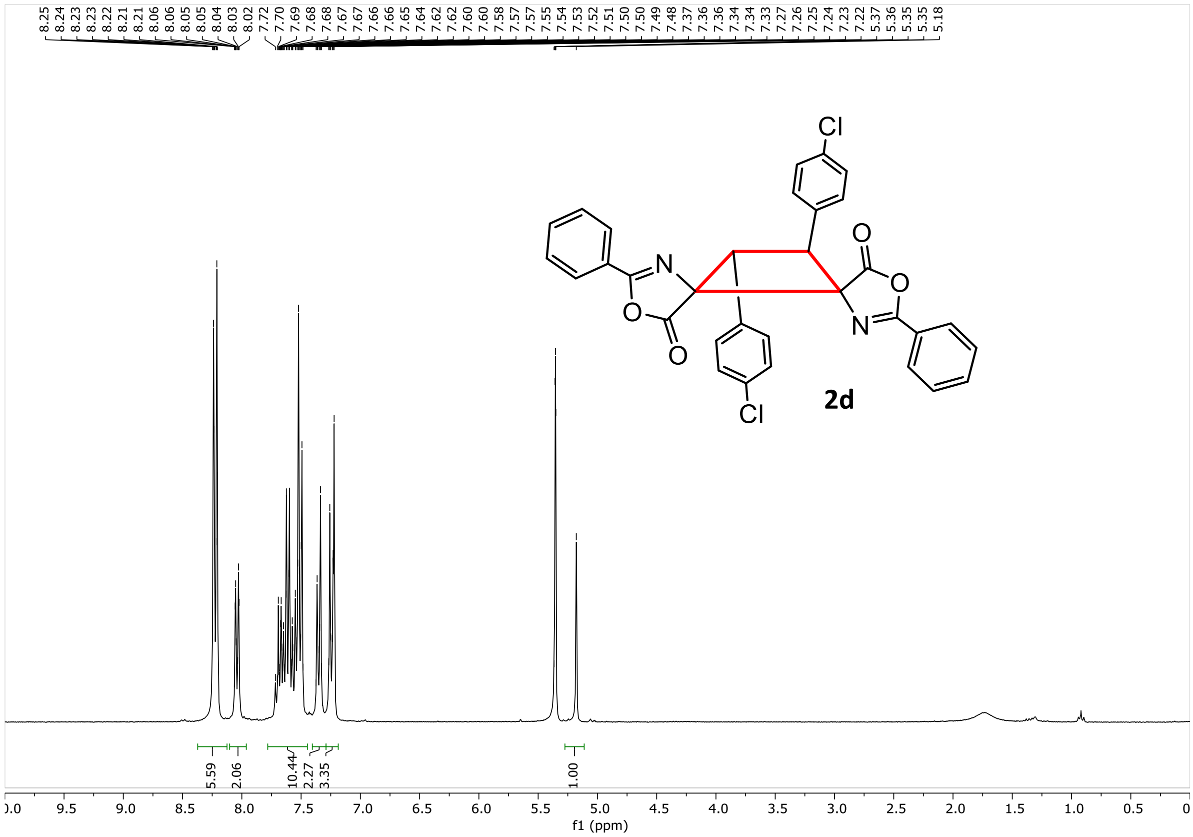

2d

^{1}H NMR (CD_2Cl_2, 300.13 MHz) of **2d**

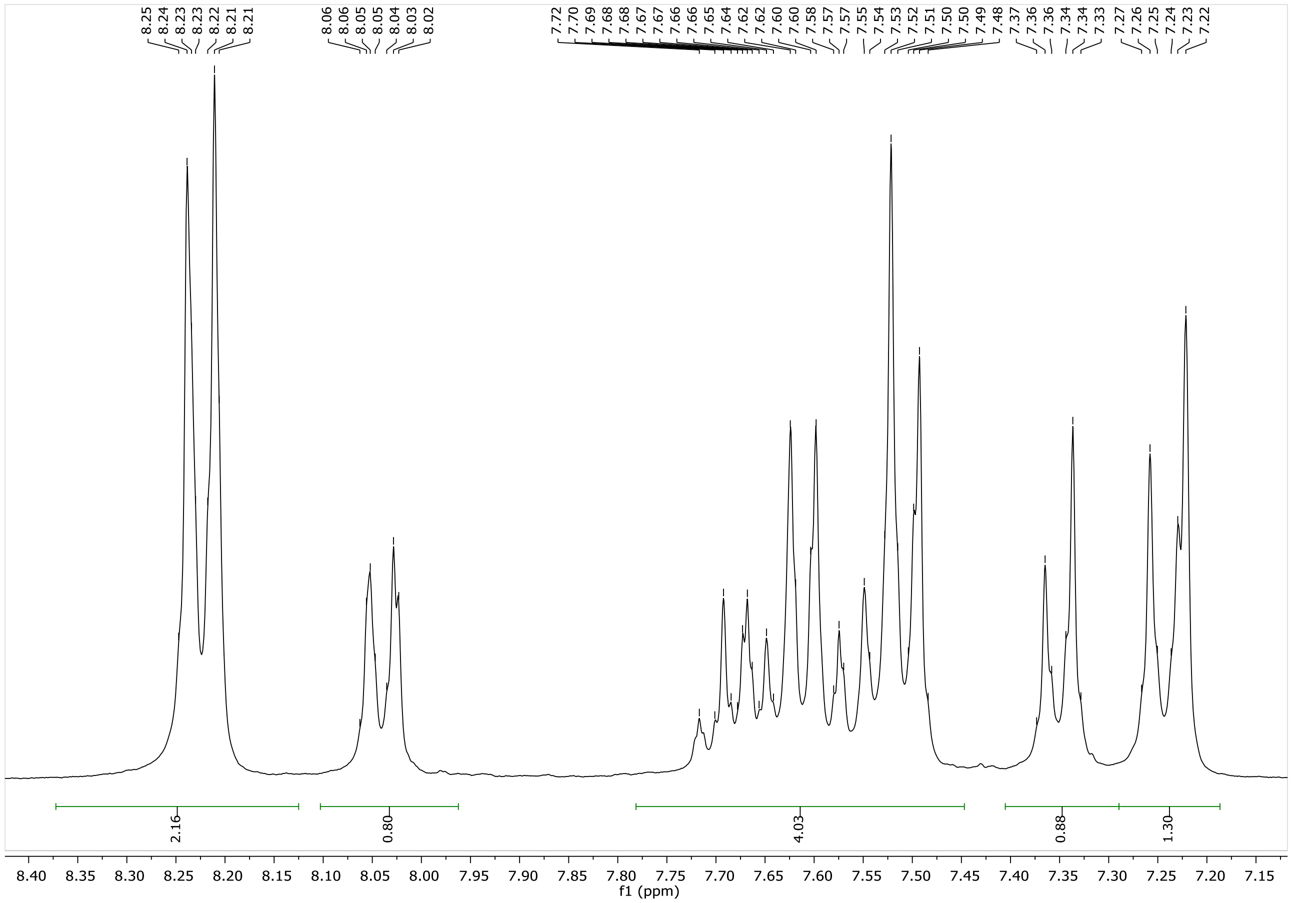

^{1}H NMR of **2d** (zoom aromatic region)

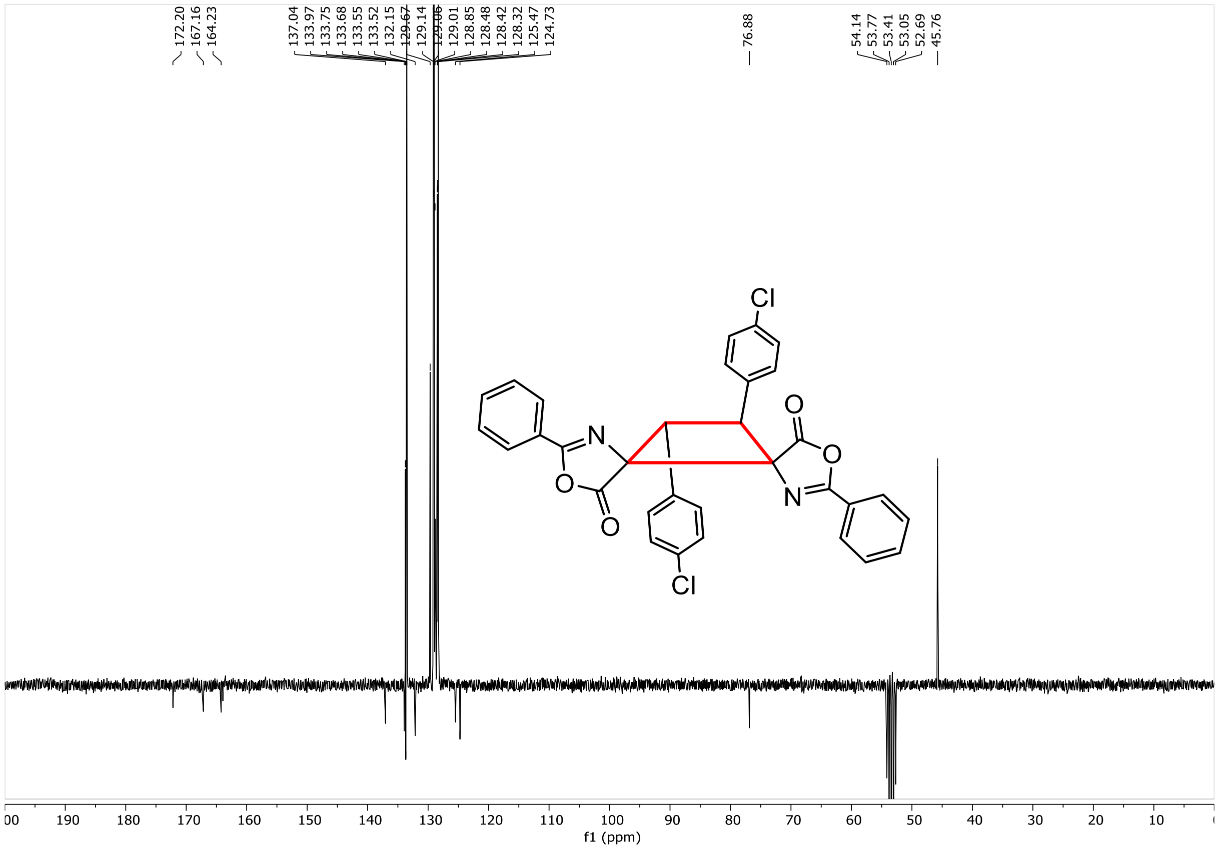

$^{13}C\{^{1}H\}$ (APT) NMR (CD_2Cl_2, 75.5 MHz) of **2d**

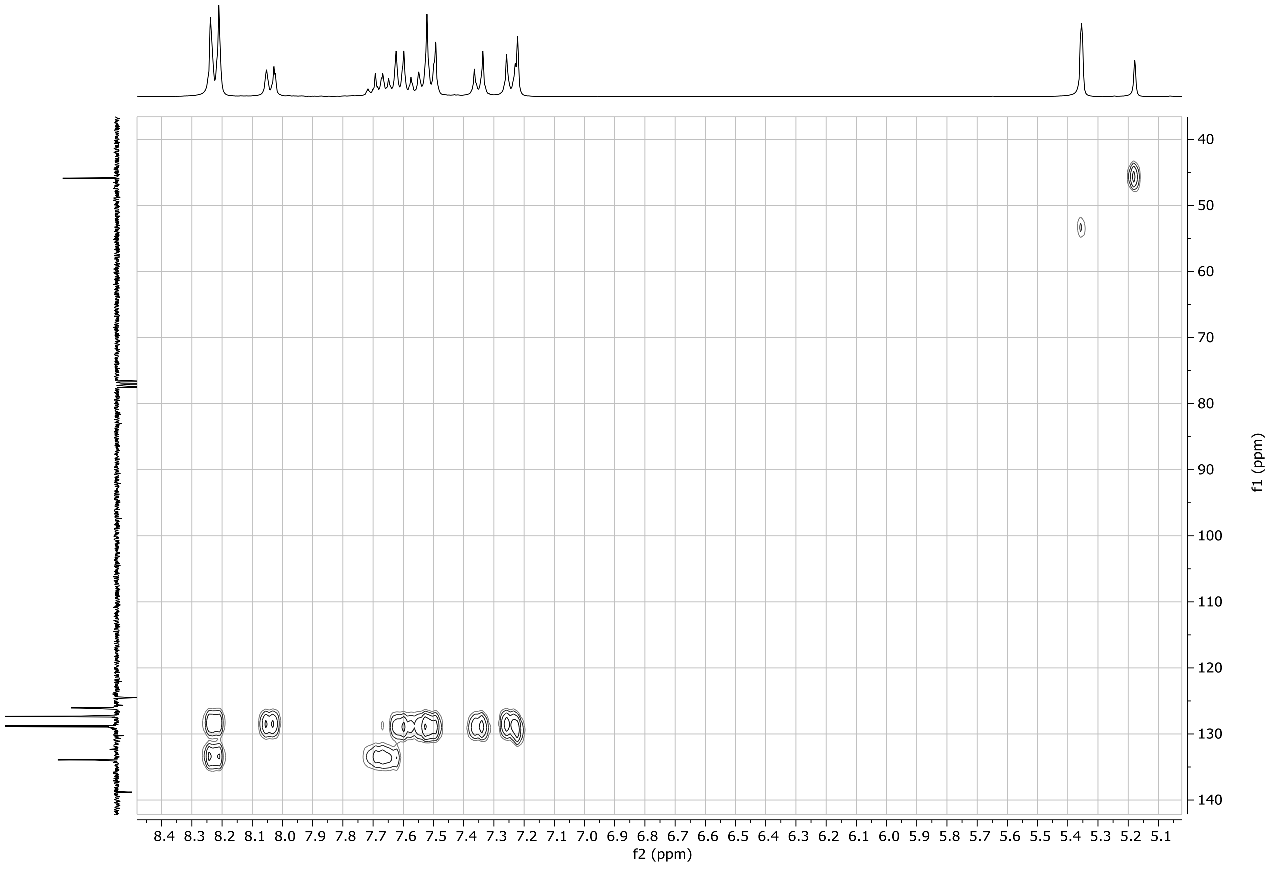

^{1}H-^{13}C HSQC correlation (CD_2Cl_2) of **2d**

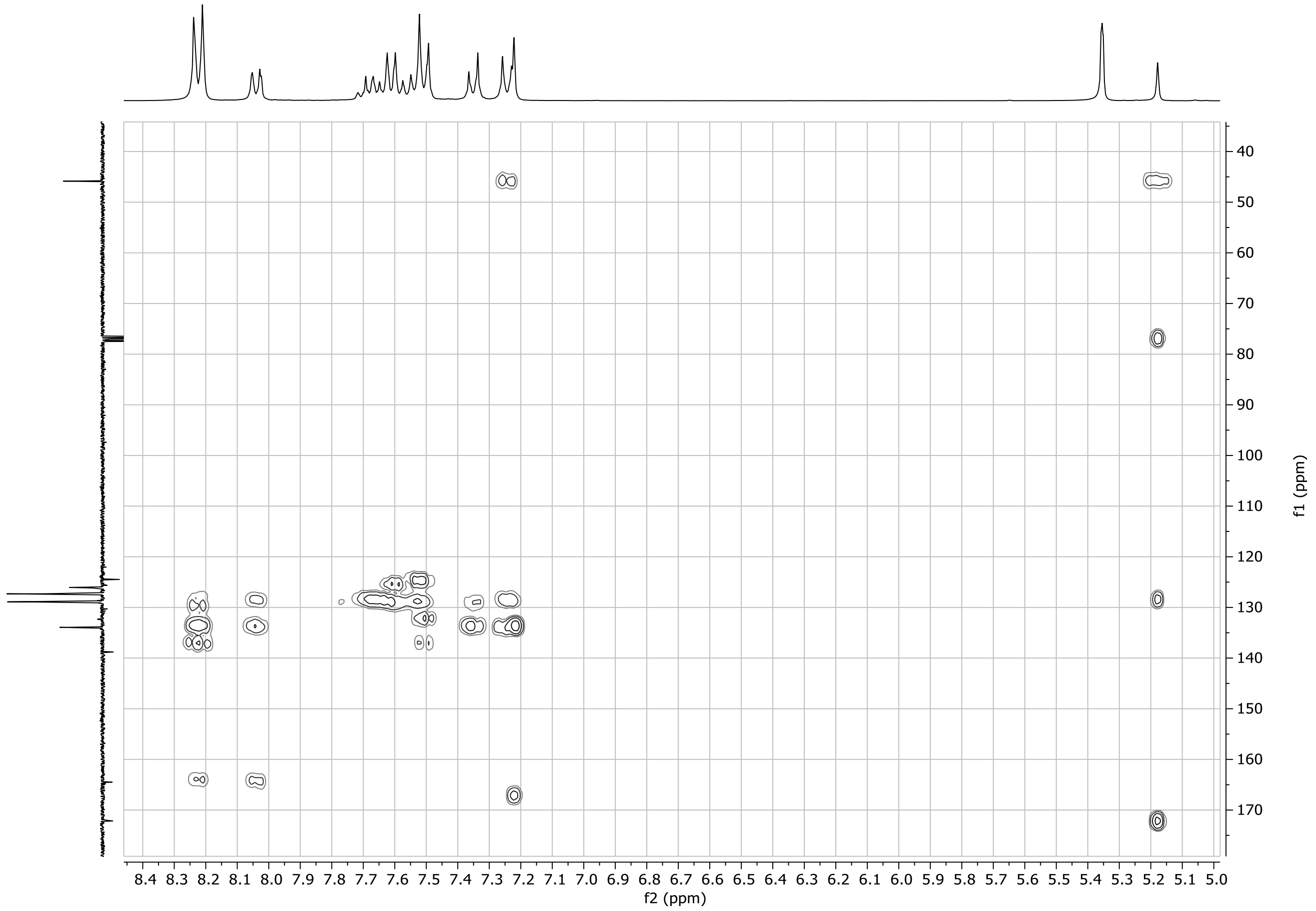

^{1}H-^{13}C HMBC correlation (CD_2Cl_2) of **2d**

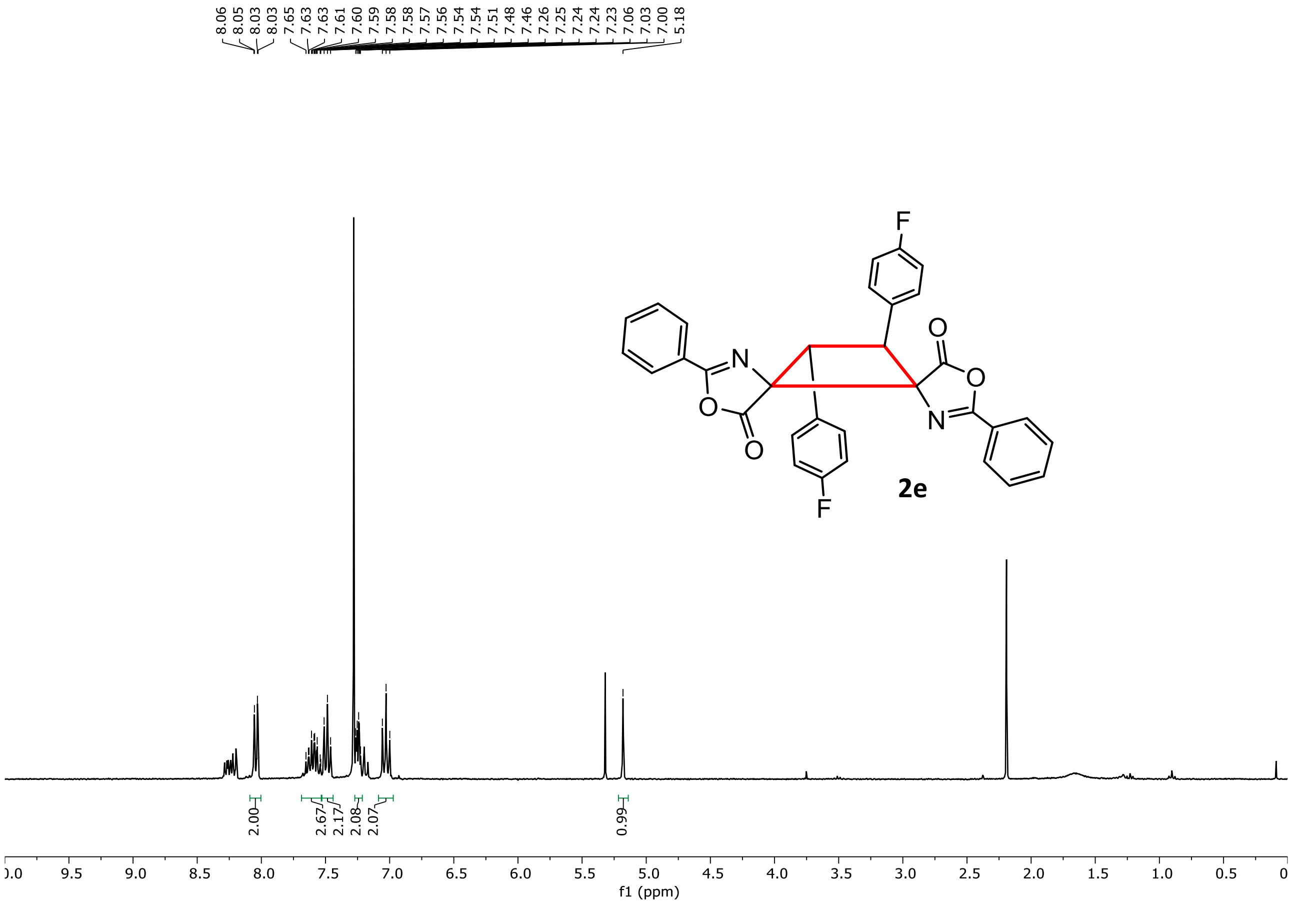

^{1}H NMR ($CDCl_3$, 300.13 MHz) of **2e**

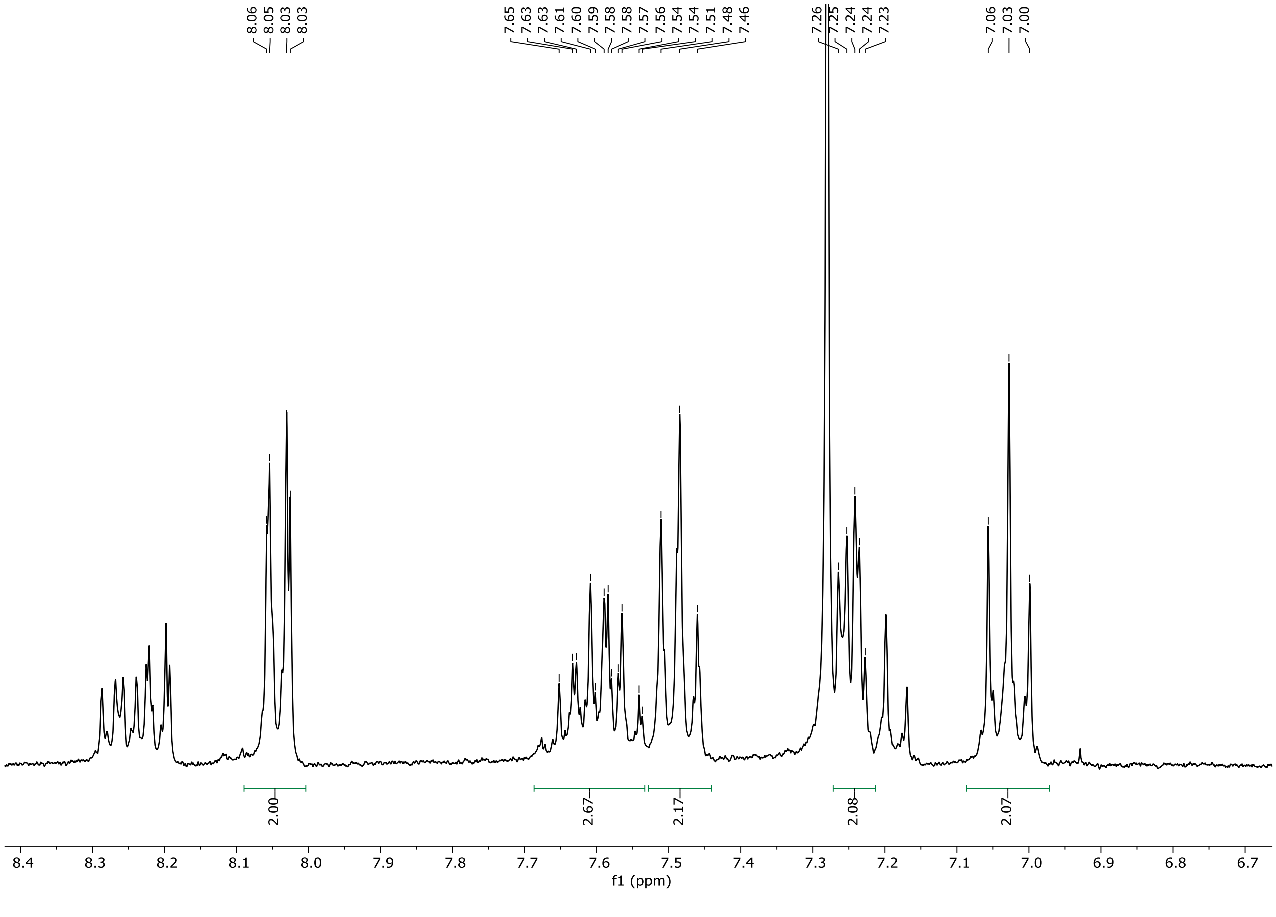

^{1}H NMR of **2e** (zoom aromatic region)

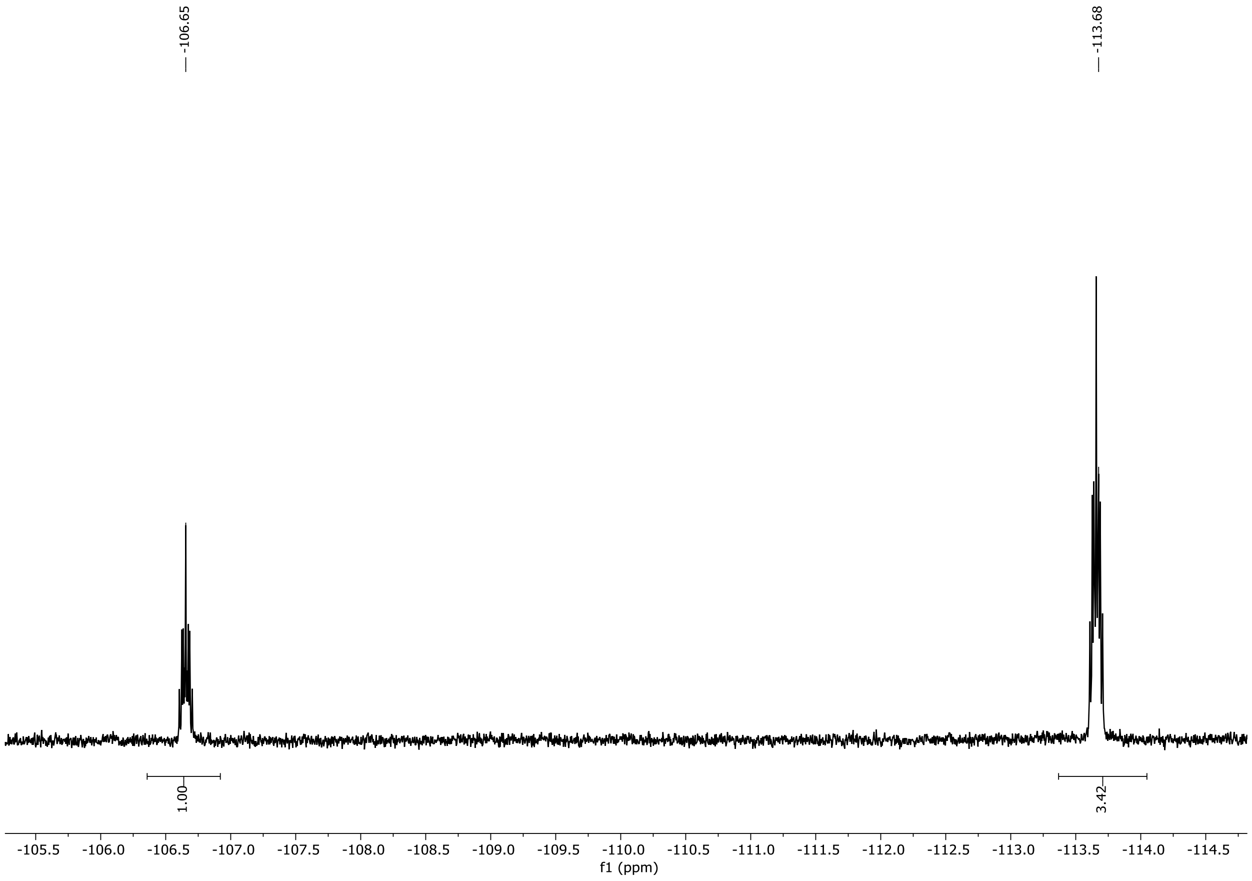

^{19}F NMR ($CDCl_3$, 282.40 MHz) of **2e**

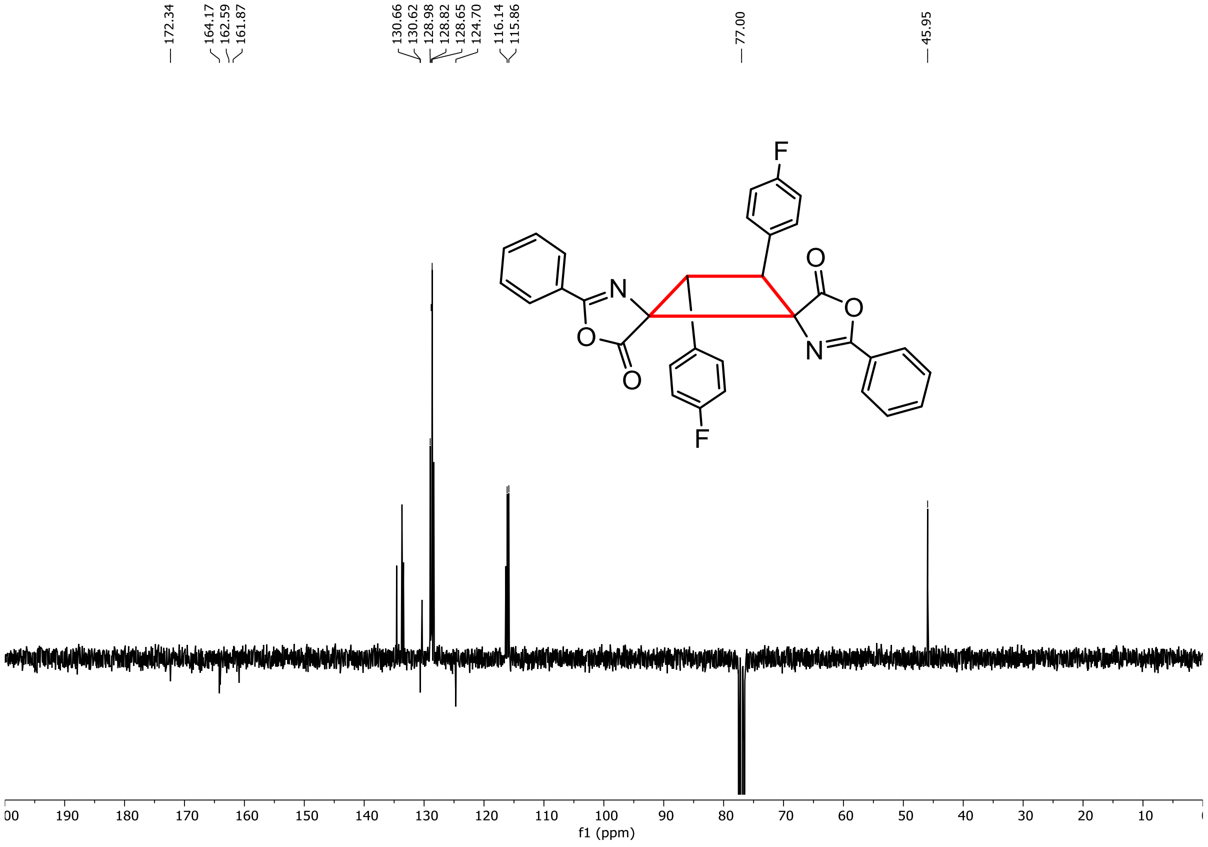

^{13}C{^{1}H} (APT) NMR ($CDCl_3$, 75.5 MHz) of **2e**

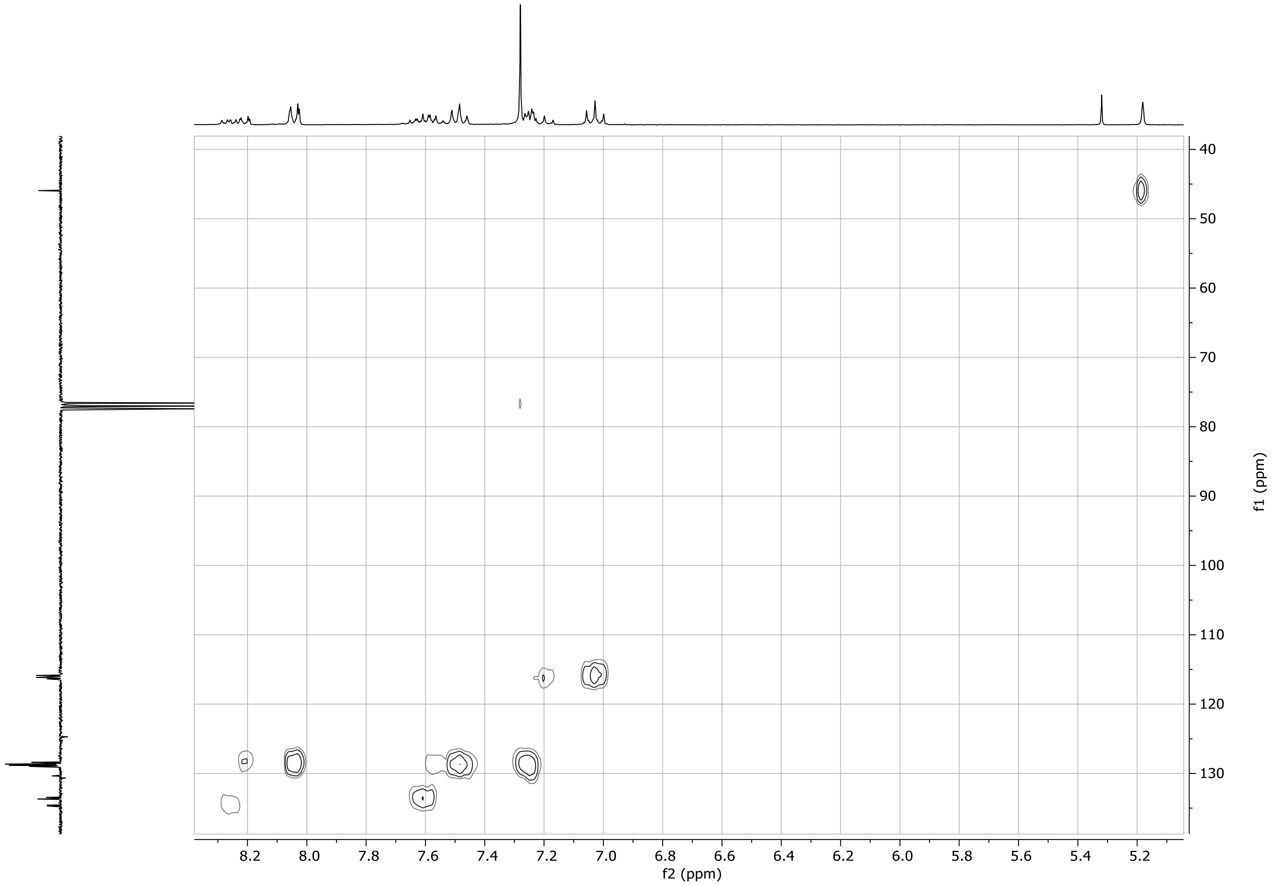

^{1}H-^{13}C HSQC correlation ($CDCl_3$) of **2e**

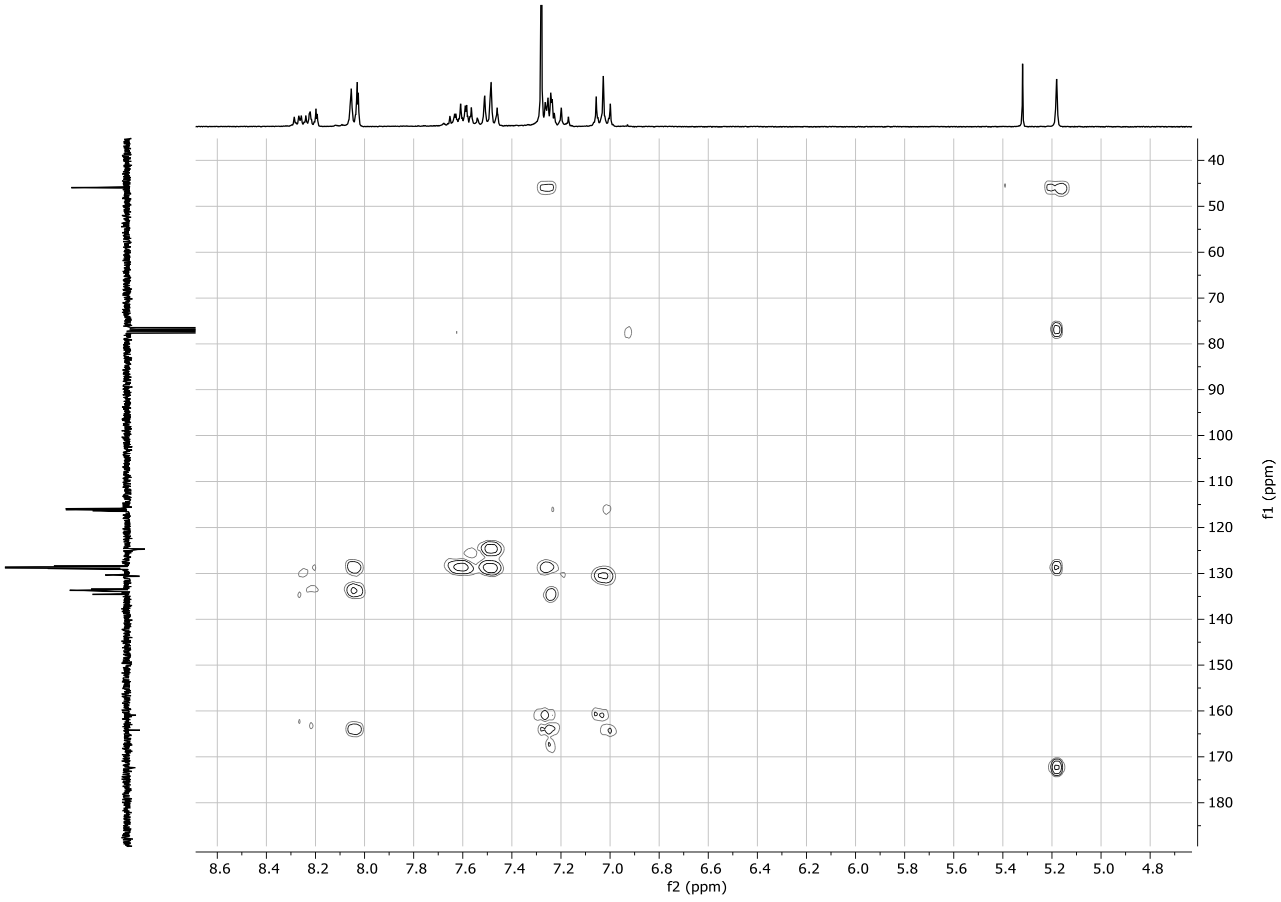

^{1}H-^{13}C HMBC correlation ($CDCl_3$) of **2e**

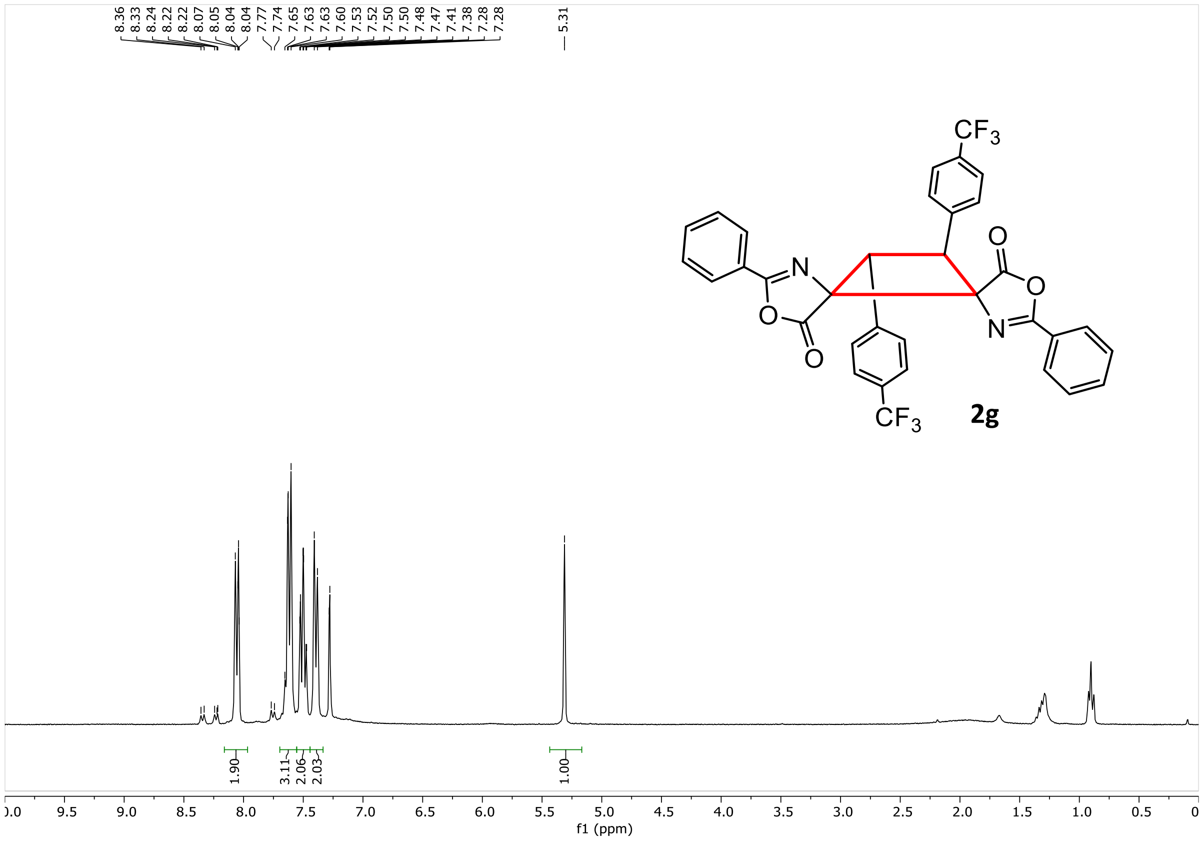

^{1}H NMR ($CDCl_3$, 300.13 MHz) of **2g**

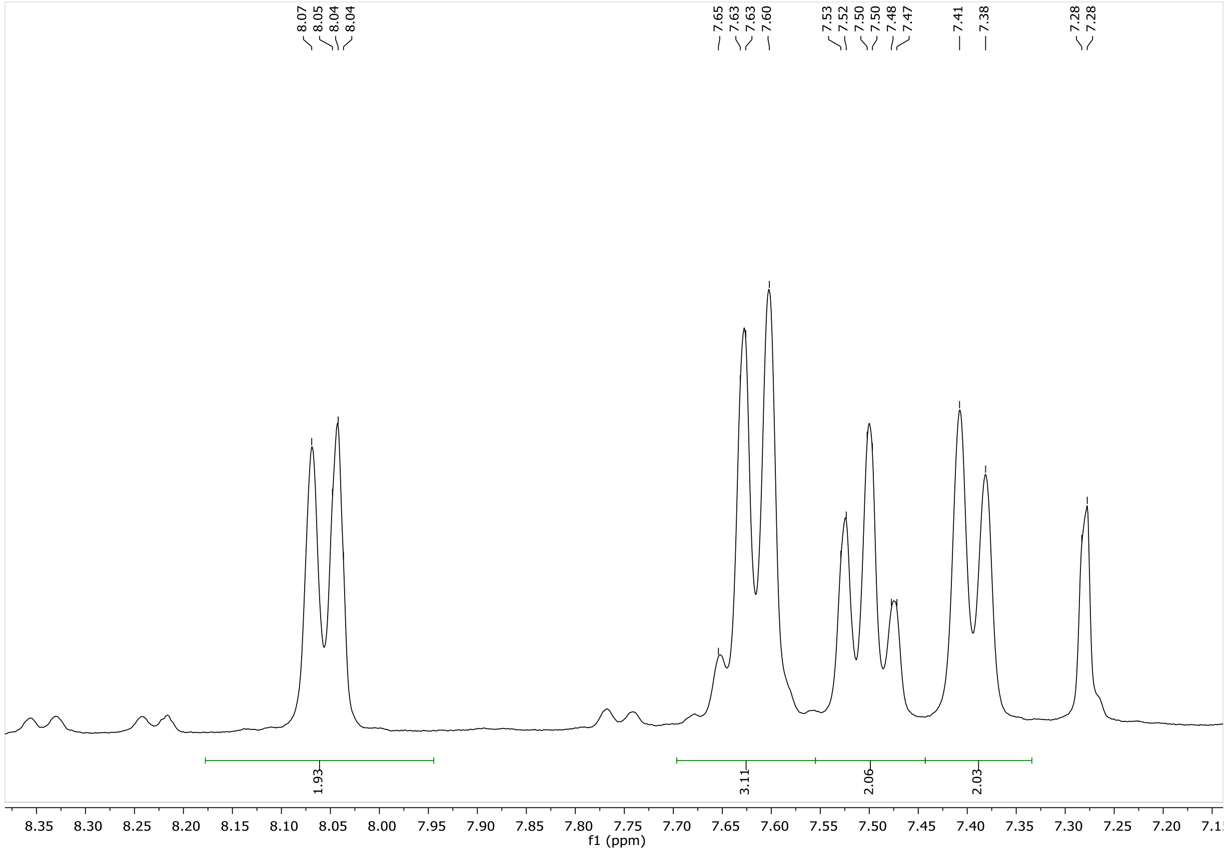

^{1}H NMR of **2g** (zoom aromatic region)

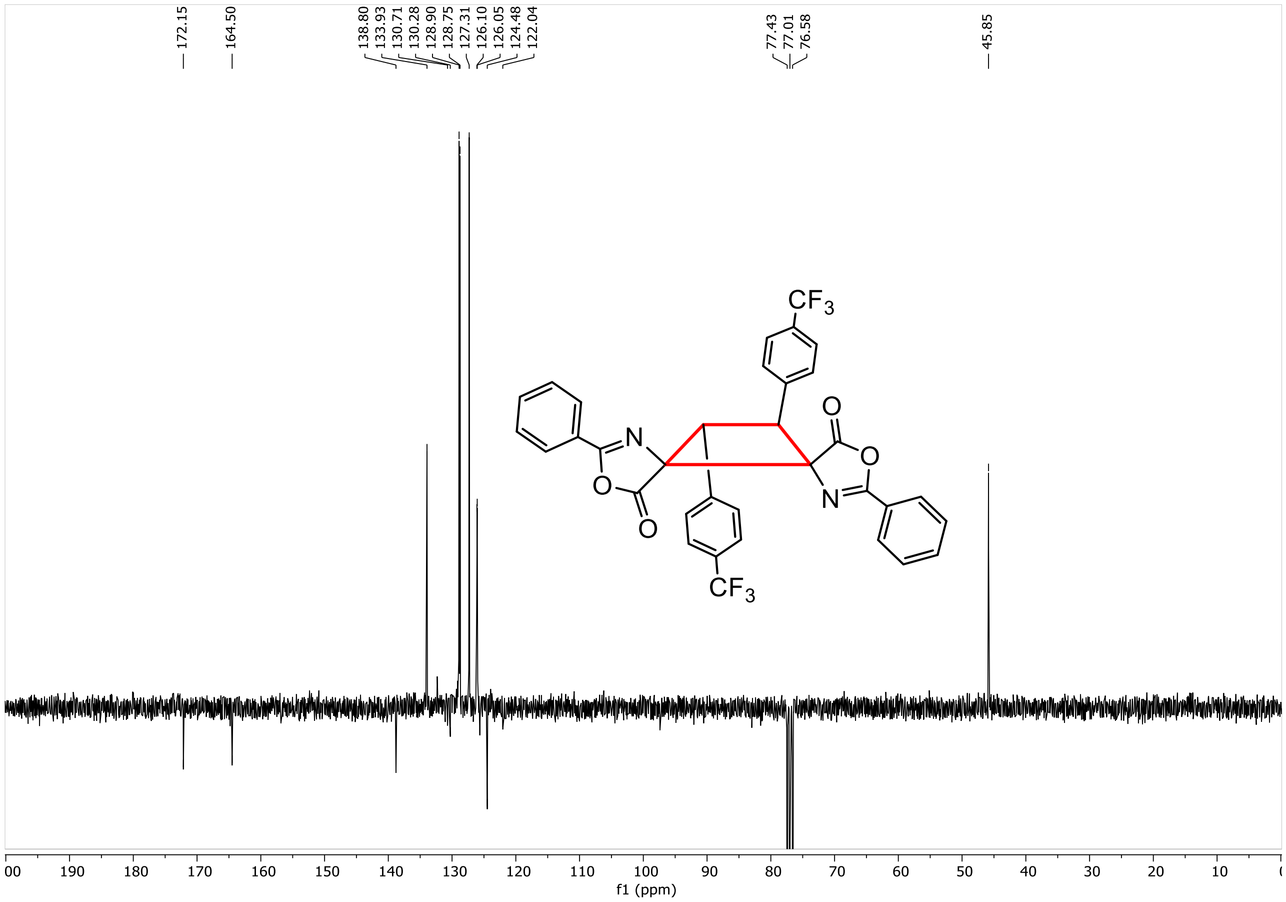

$^{13}C\{^1H\}$ (APT) NMR ($CDCl_3$, 75.5 MHz) of **2g**

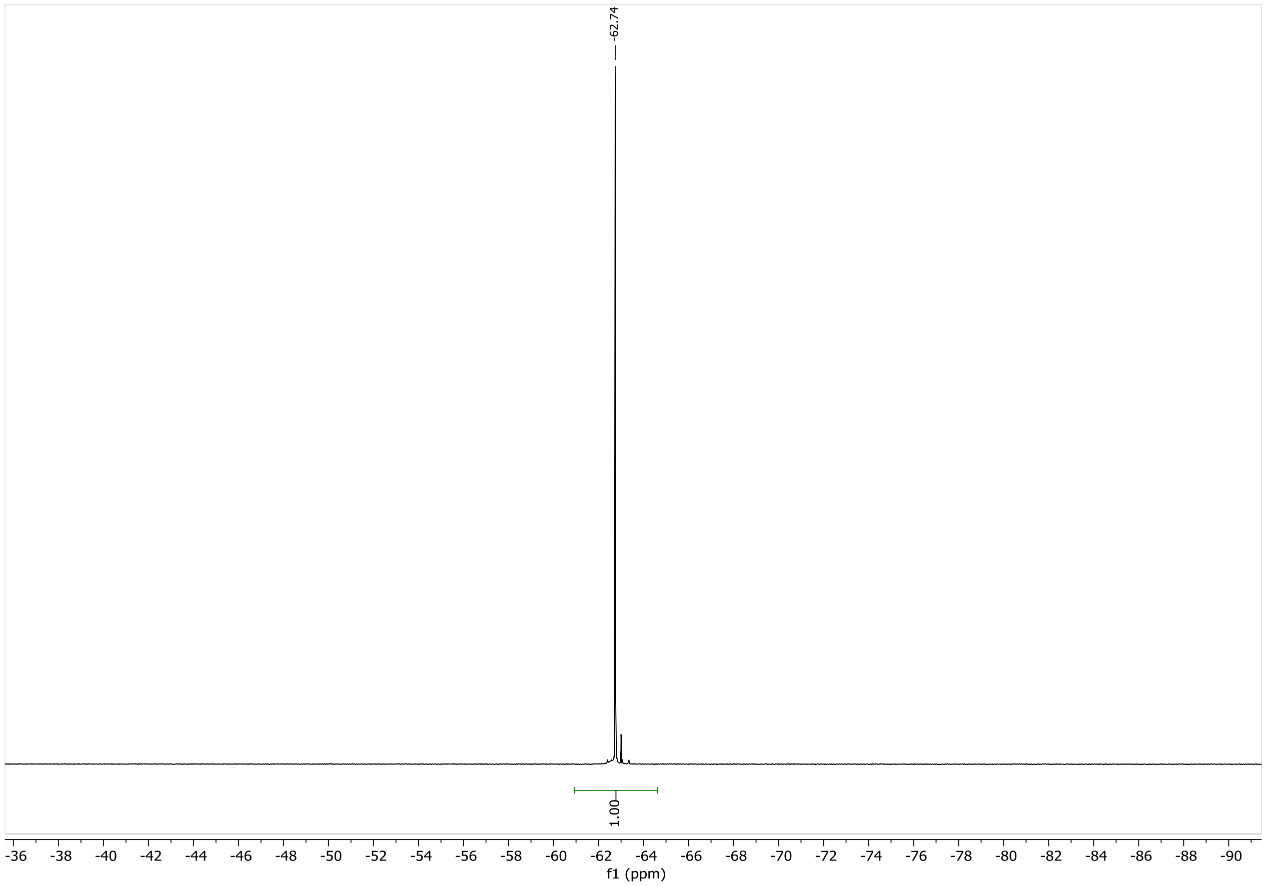

^{19}F NMR ($CDCl_3$, 282.40 MHz) of **2g**

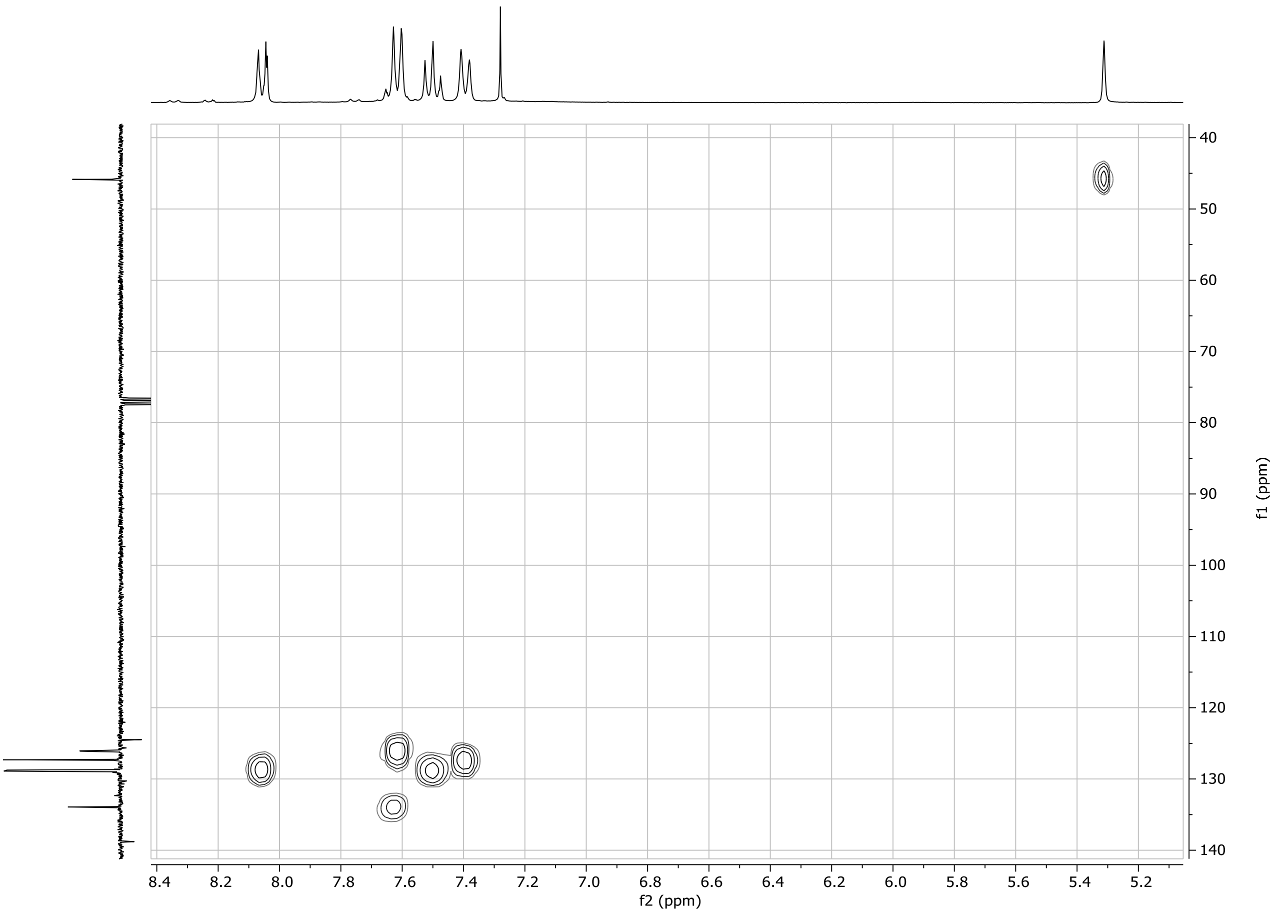

^{1}H-^{13}C HSQC correlation ($CDCl_3$) of **2g**

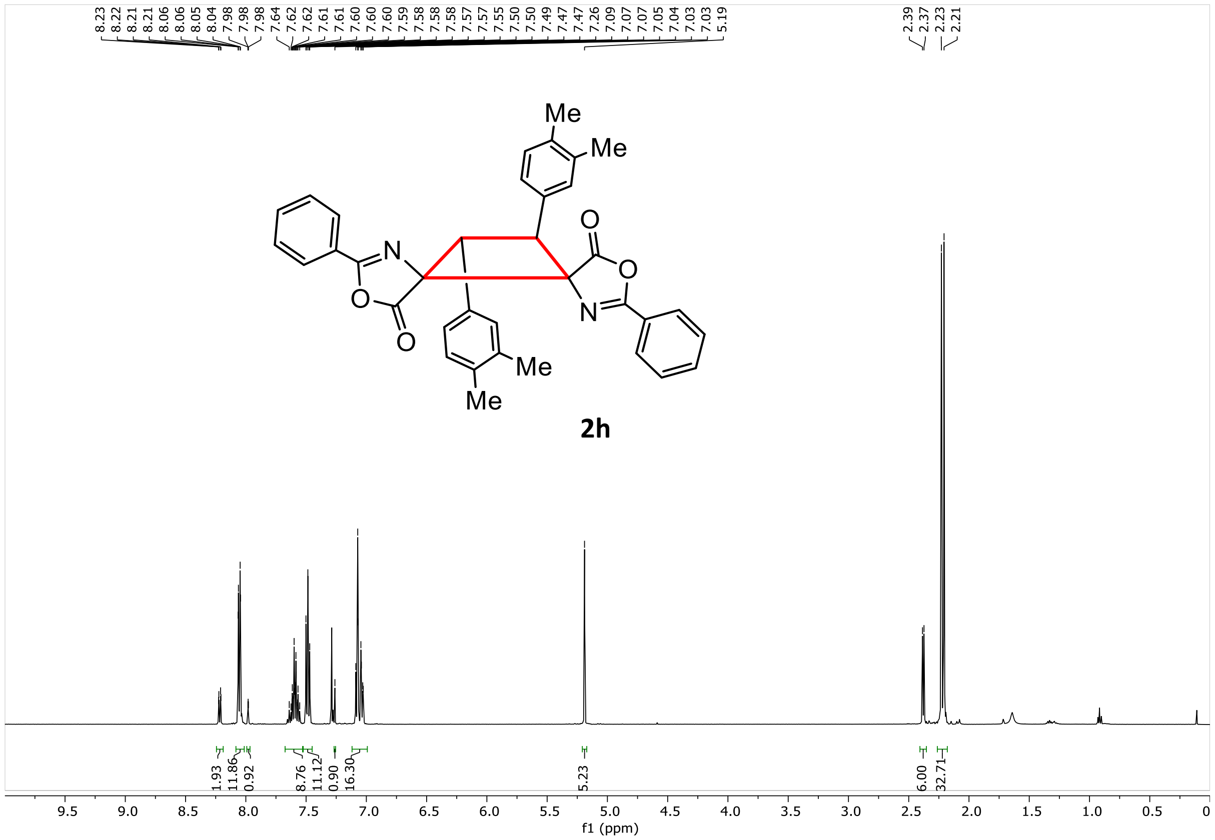

^{1}H NMR ($CDCl_3$, 300.13 MHz) of **2h**

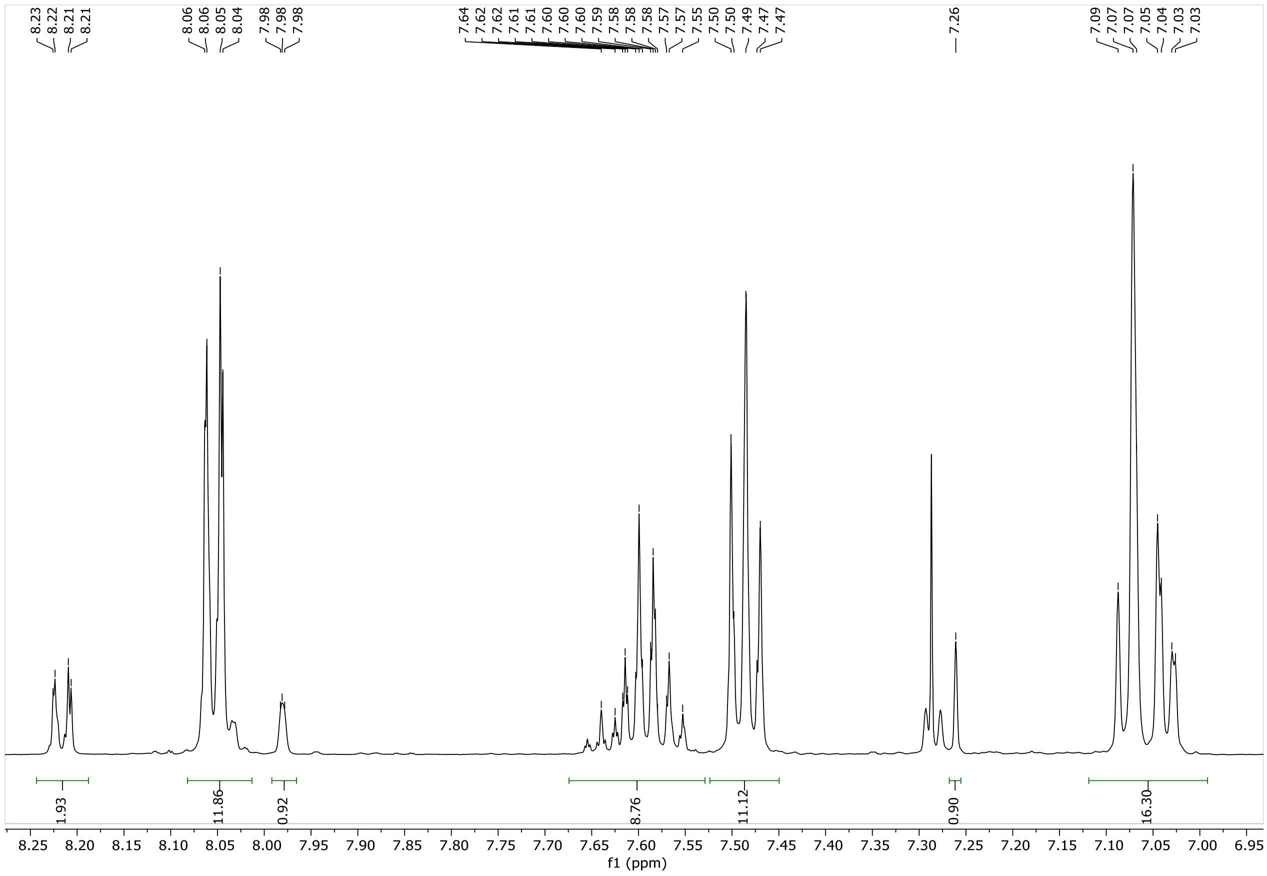

^{1}H NMR of **2h** (zoom aromatic region)

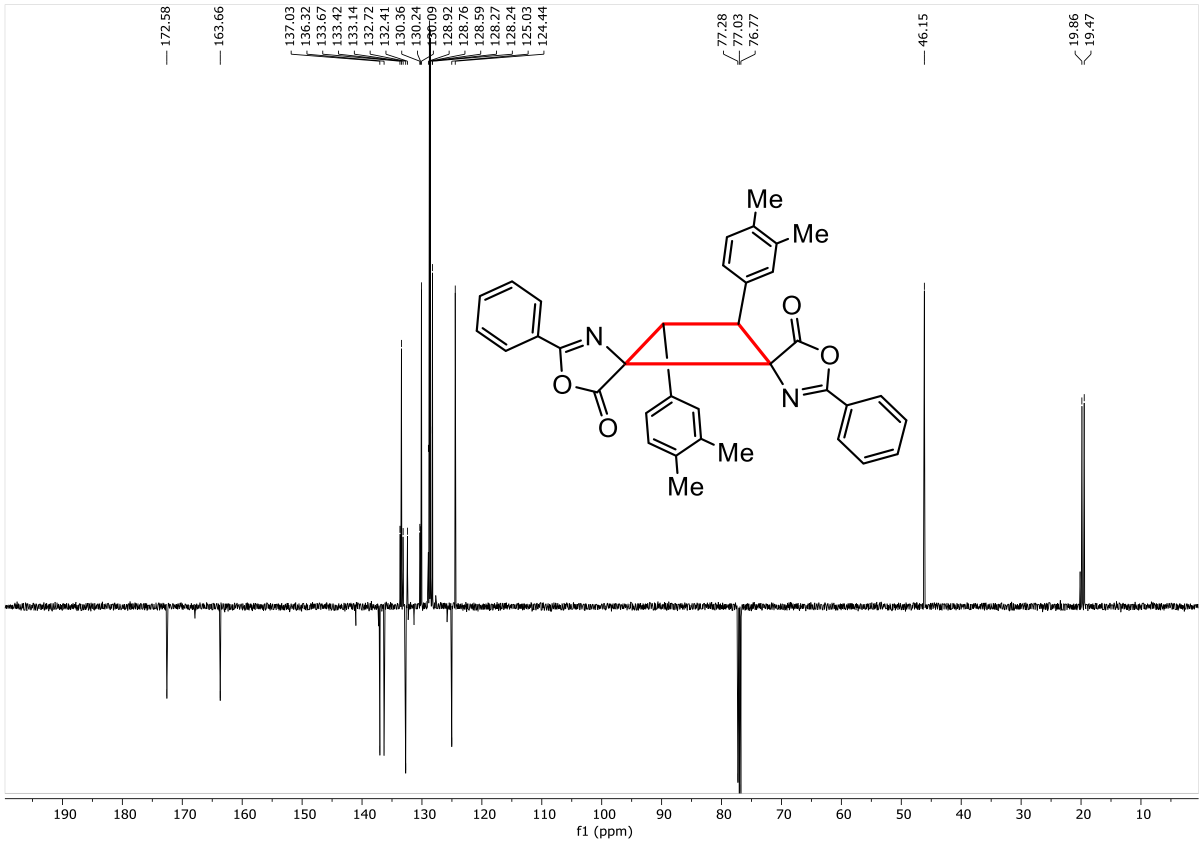

^{13}C{^{1}H} (APT) NMR ($CDCl_3$, 75.5 MHz) of **2h**

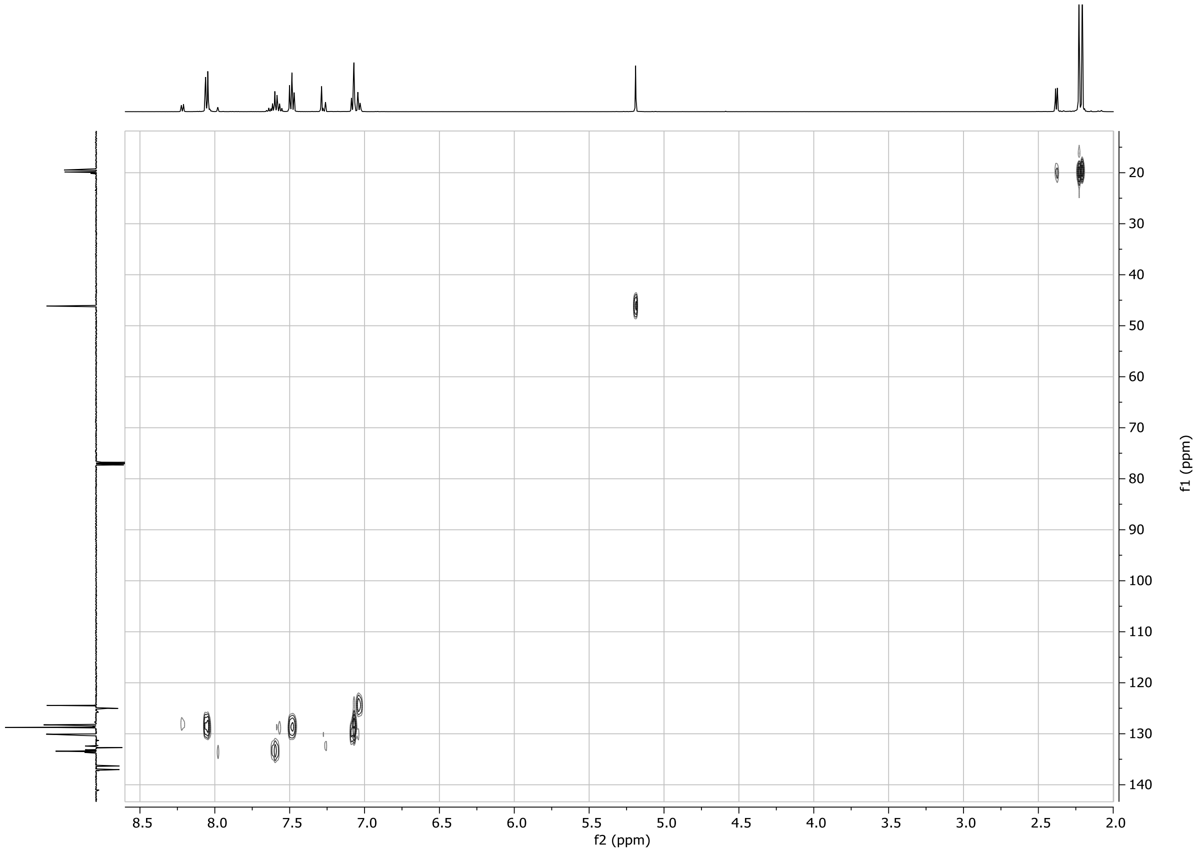

^{1}H-^{13}C HSQC correlation ($CDCl_3$) of **2h**

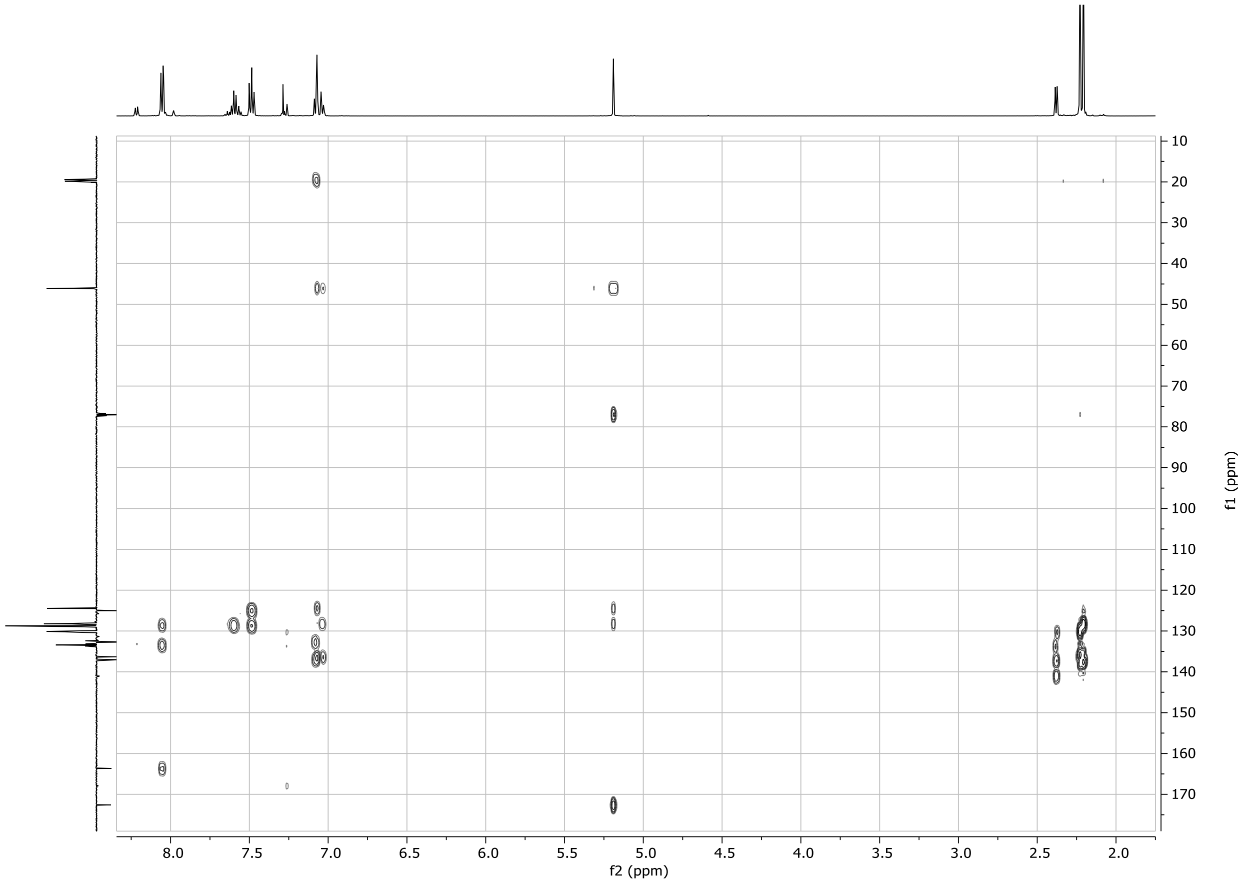

^{1}H-^{13}C HMBC correlation ($CDCl_3$) of **2h**

3.- NMR spectra of the 1,2-diaminotruxinic acid derivatives 3a-3j (except 3g, see main text)

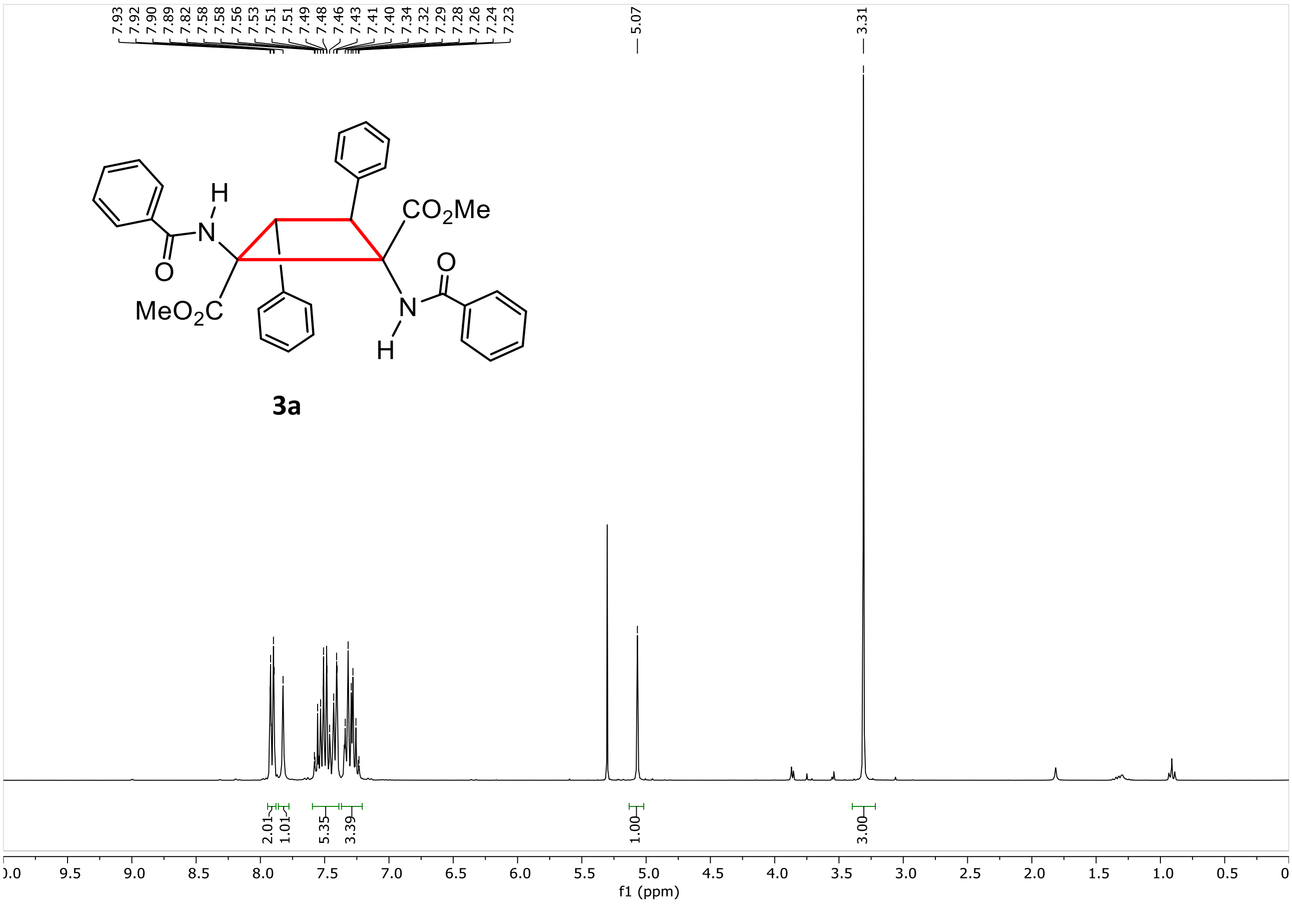

^{1}H NMR ($CDCl_3$, 300.13 MHz) of **3a**

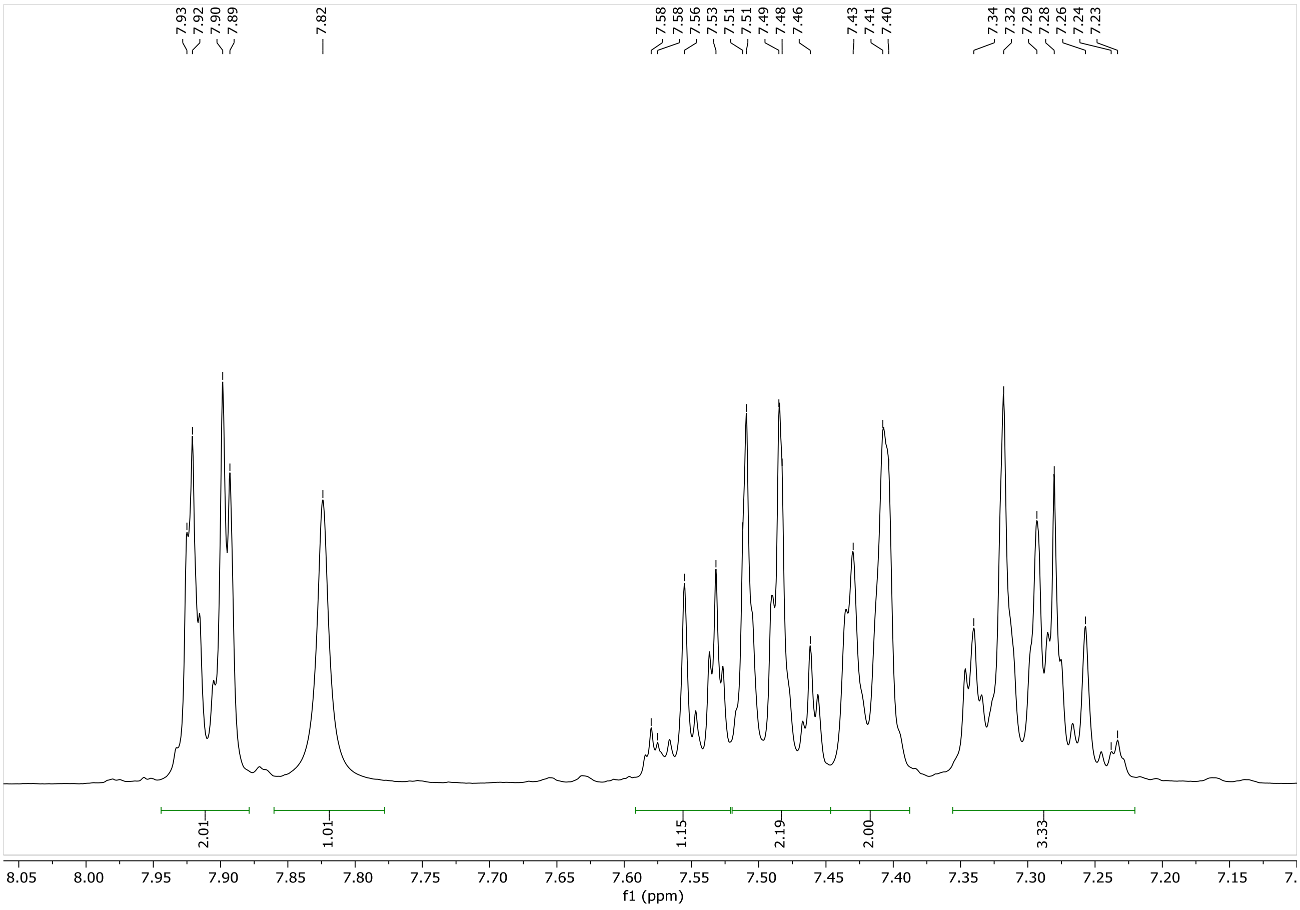

^{1}H NMR of **3a** (zoom aromatic region)

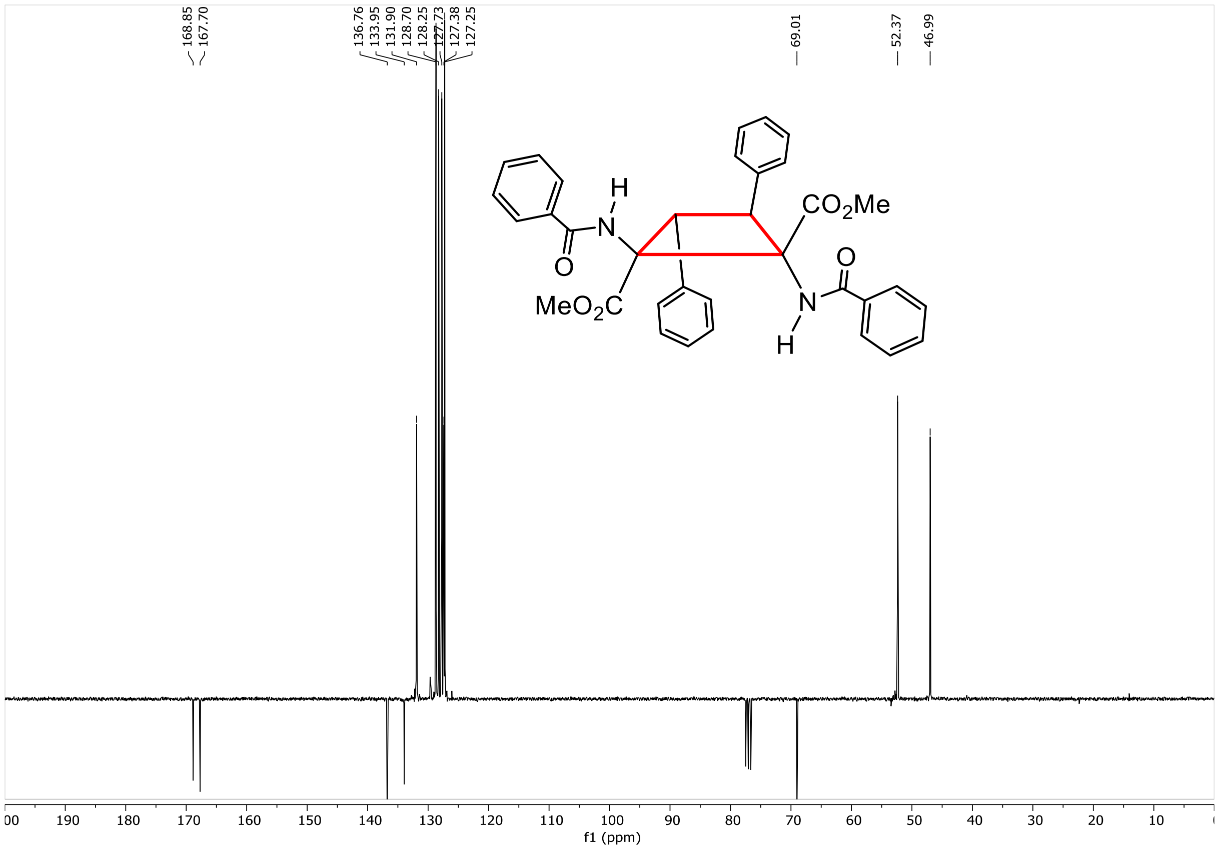

$^{13}C\{^{1}H\}$ (APT) NMR ($CDCl_3$, 75.5 MHz) of **3a**

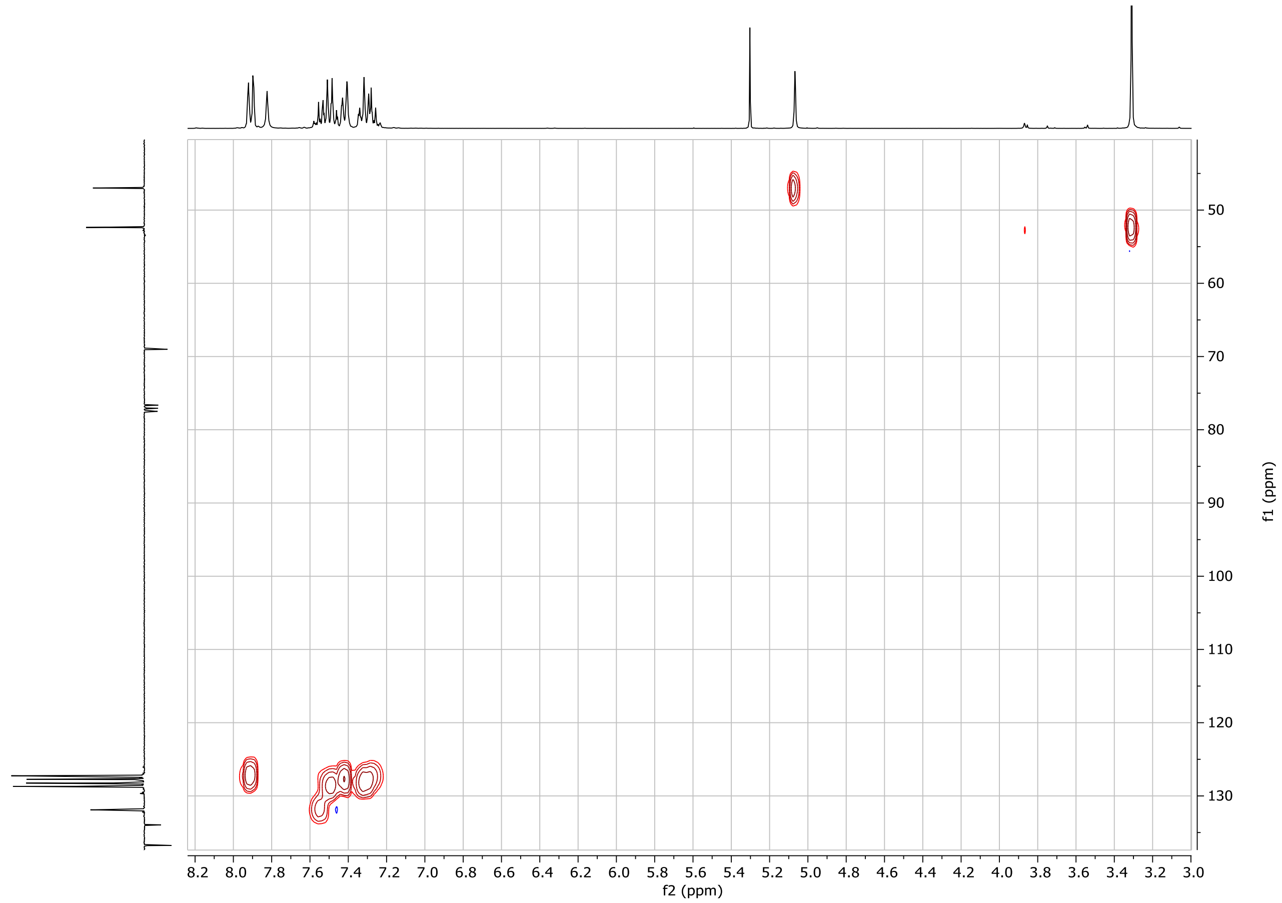

^{1}H-^{13}C HSQC correlation ($CDCl_3$) of **3a**

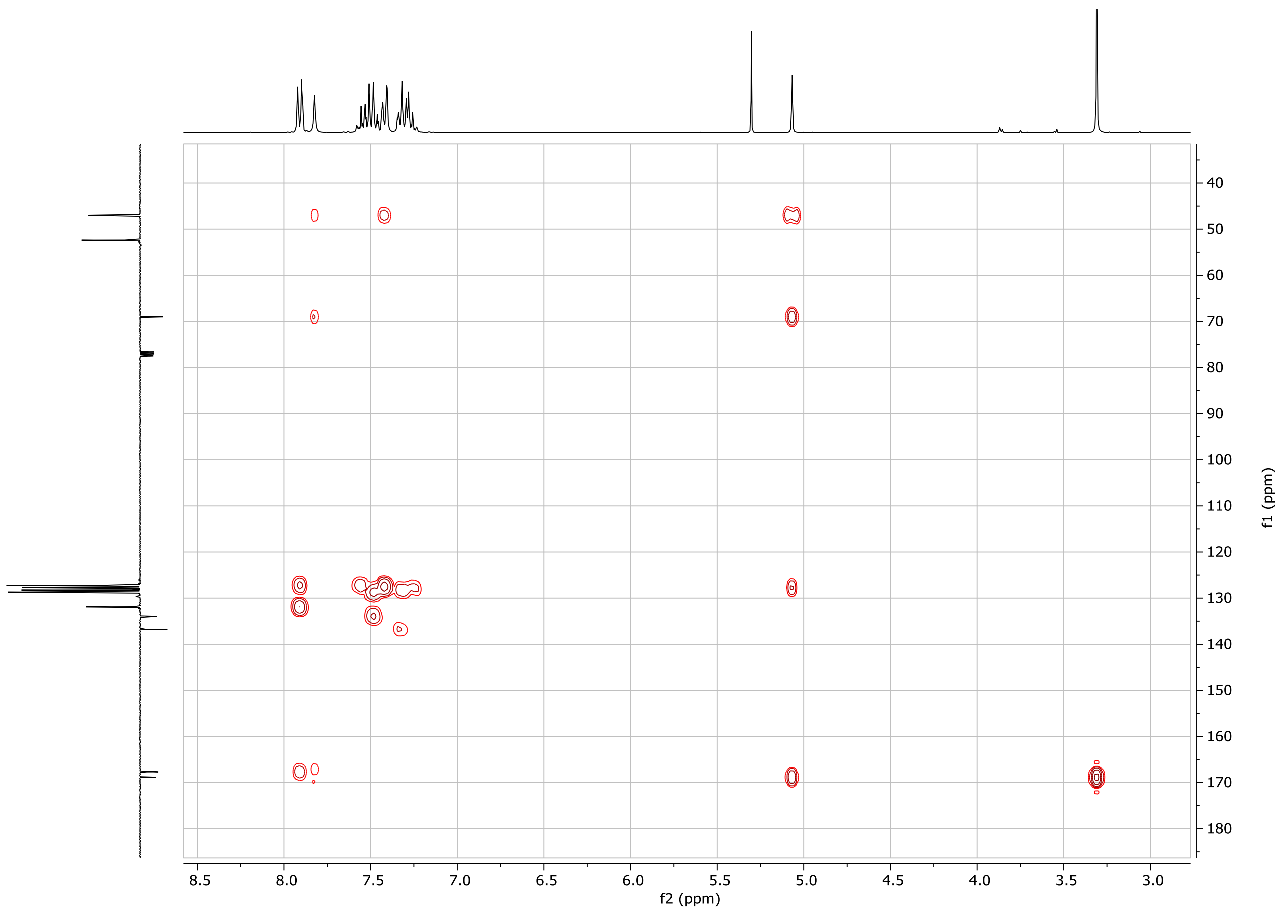

^{1}H-^{13}C HMBC correlation ($CDCl_3$) of **3a**

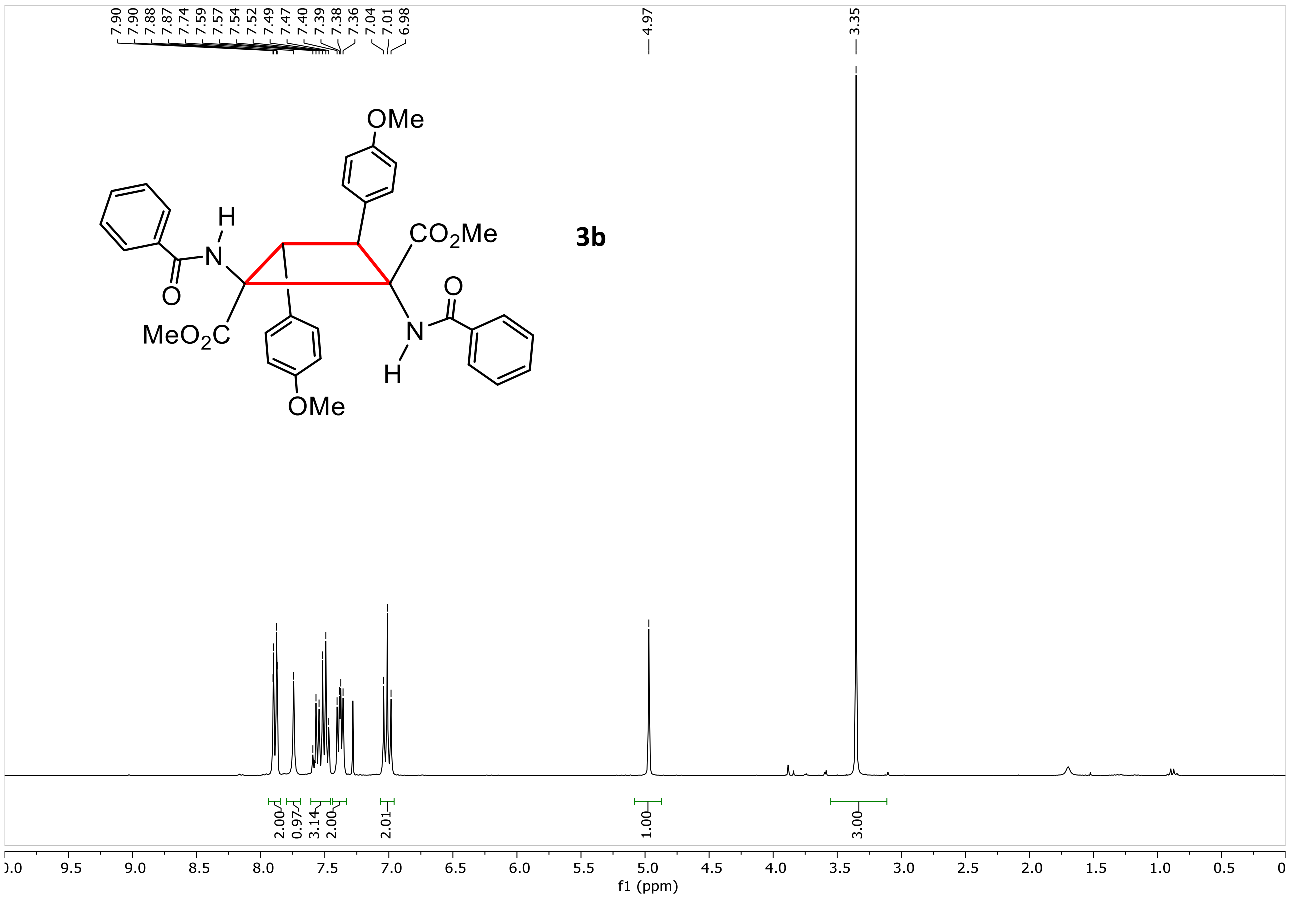

^{1}H NMR ($CDCl_3$, 300.13 MHz) of **3b**

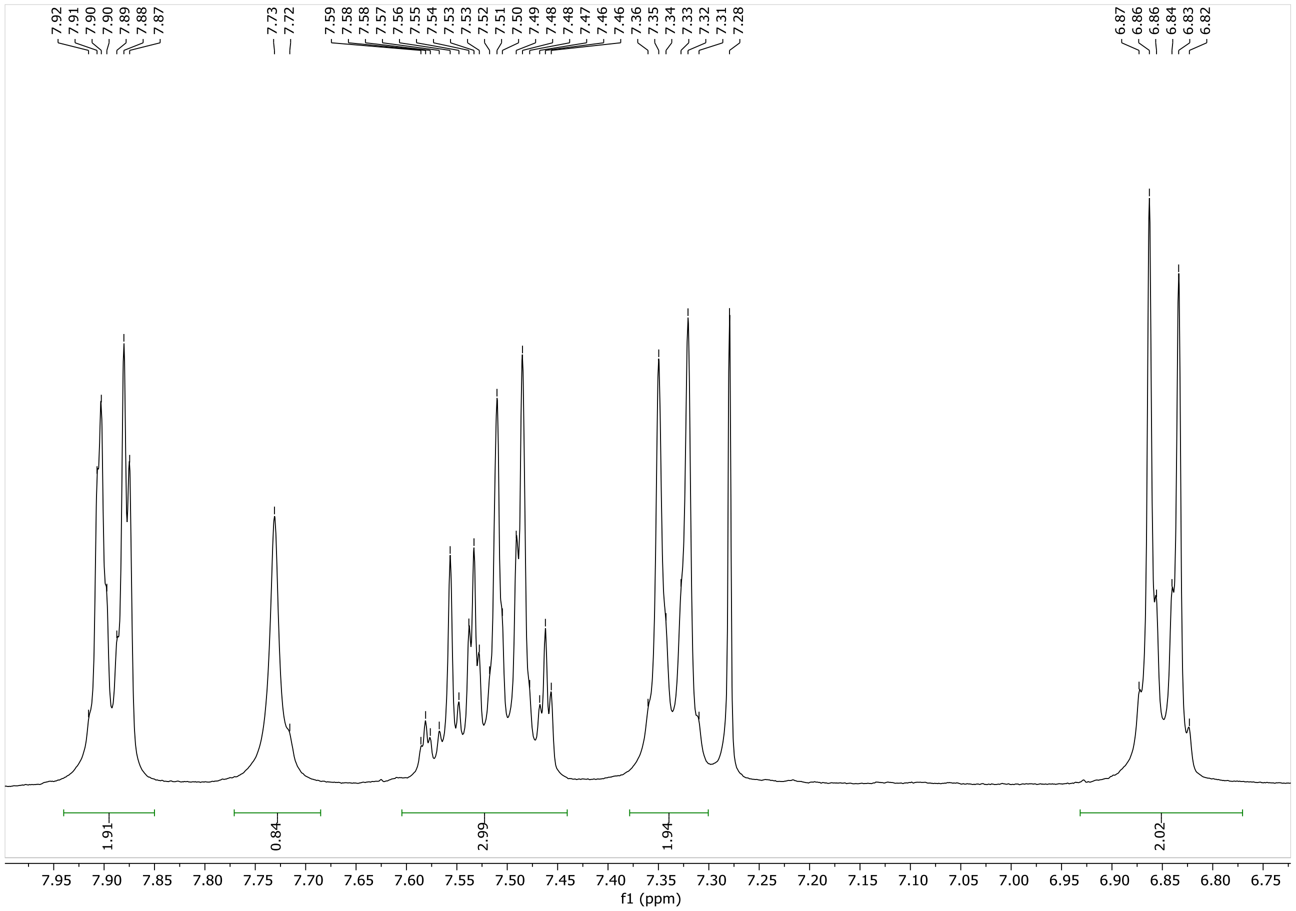

^{1}H NMR of **3b** (zoom aromatic region)

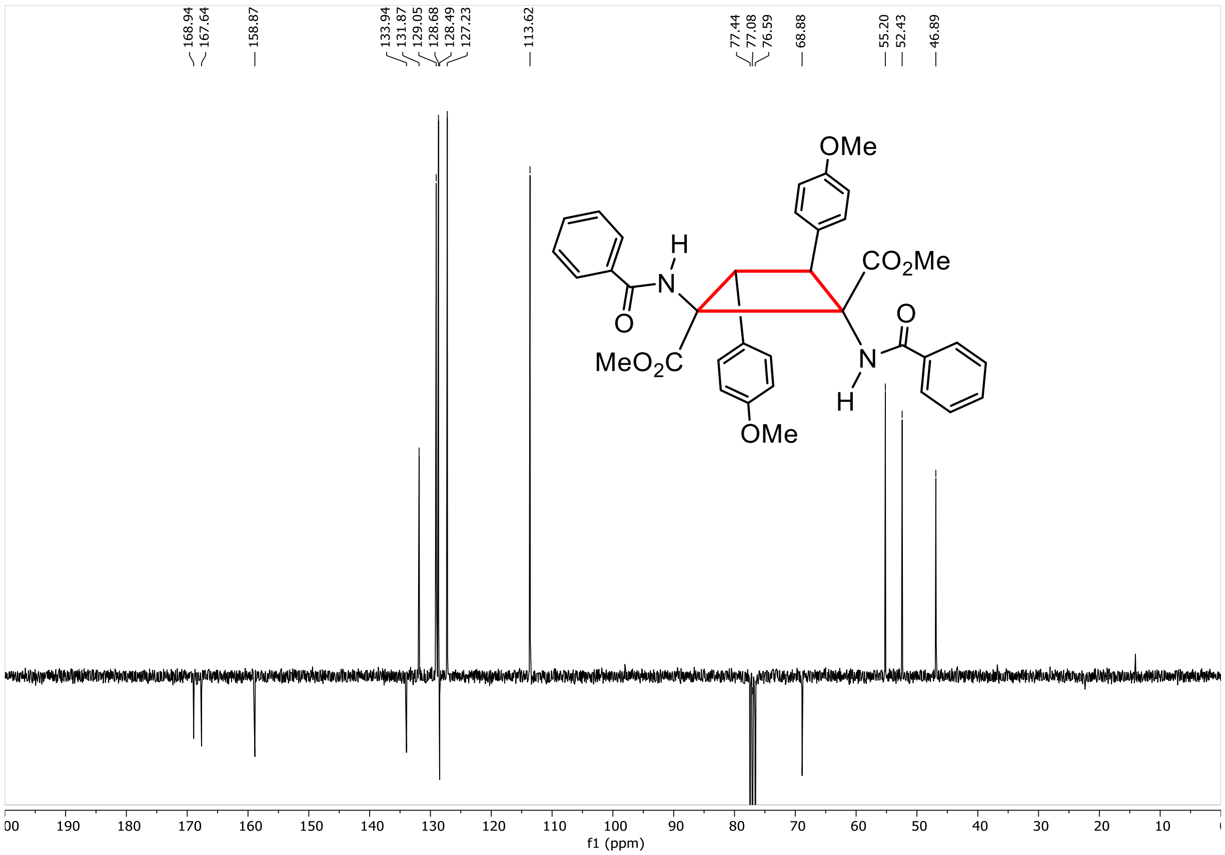

$^{13}C\{^{1}H\}$ (APT) NMR ($CDCl_3$, 75.5 MHz) of **3b**

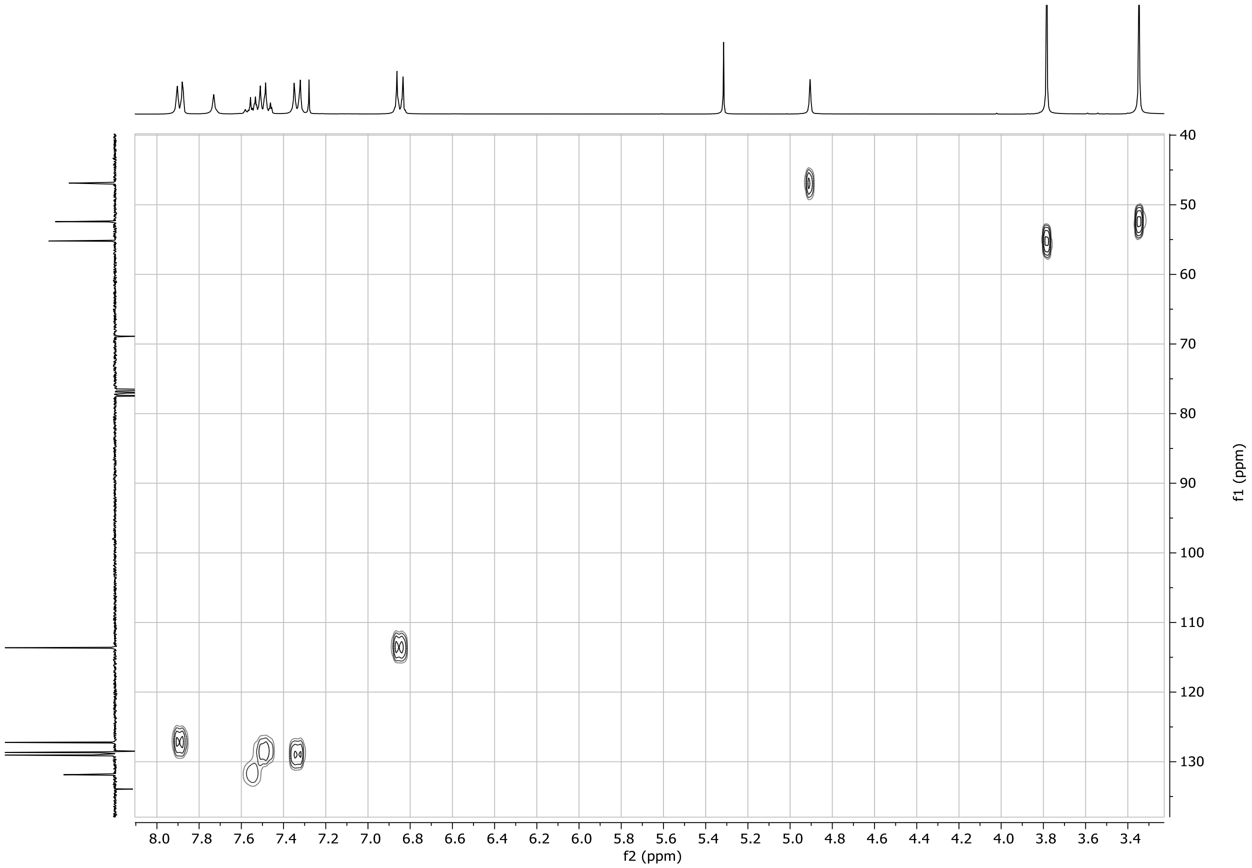

^{1}H-^{13}C HSQC correlation ($CDCl_3$) of **3b**

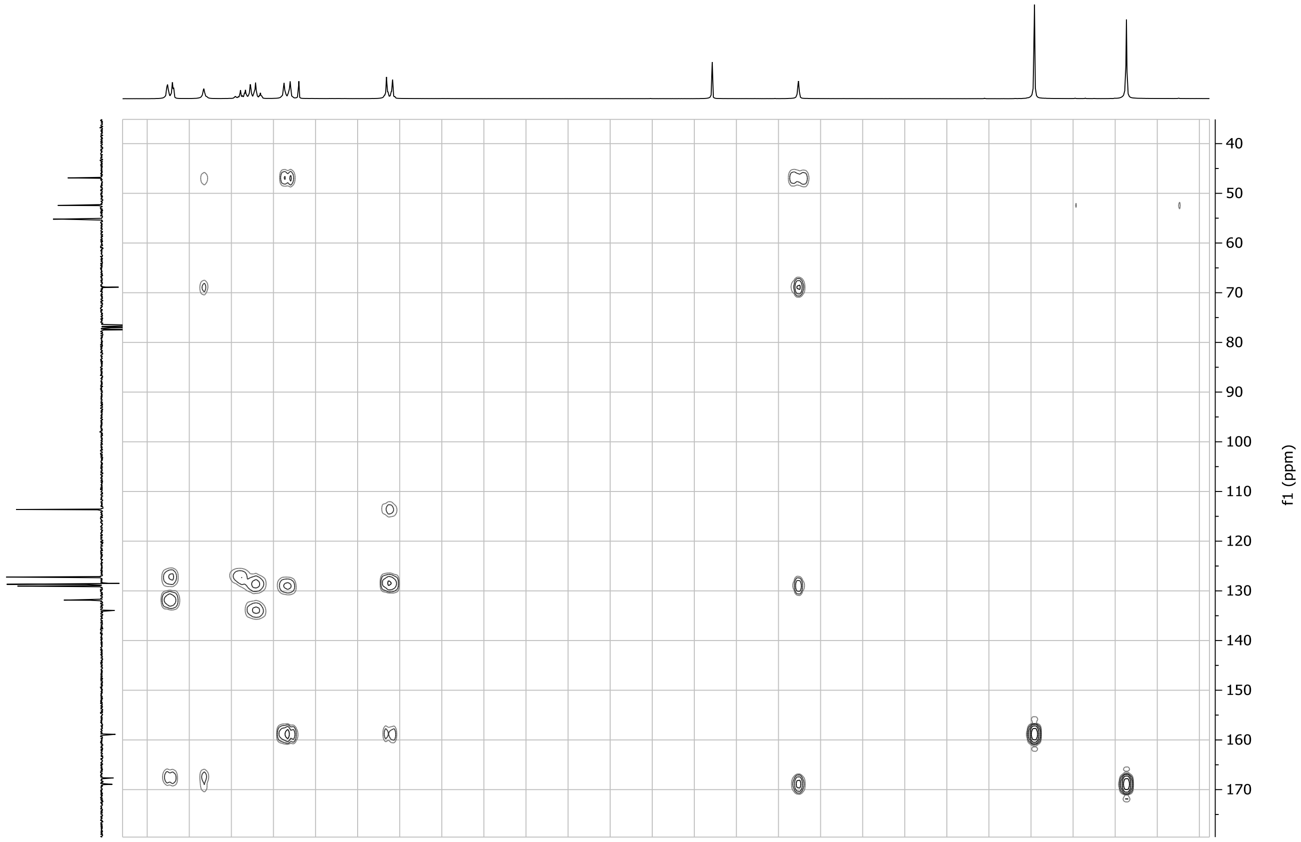

^{1}H-^{13}C HMBC correlation ($CDCl_3$) of **3b**

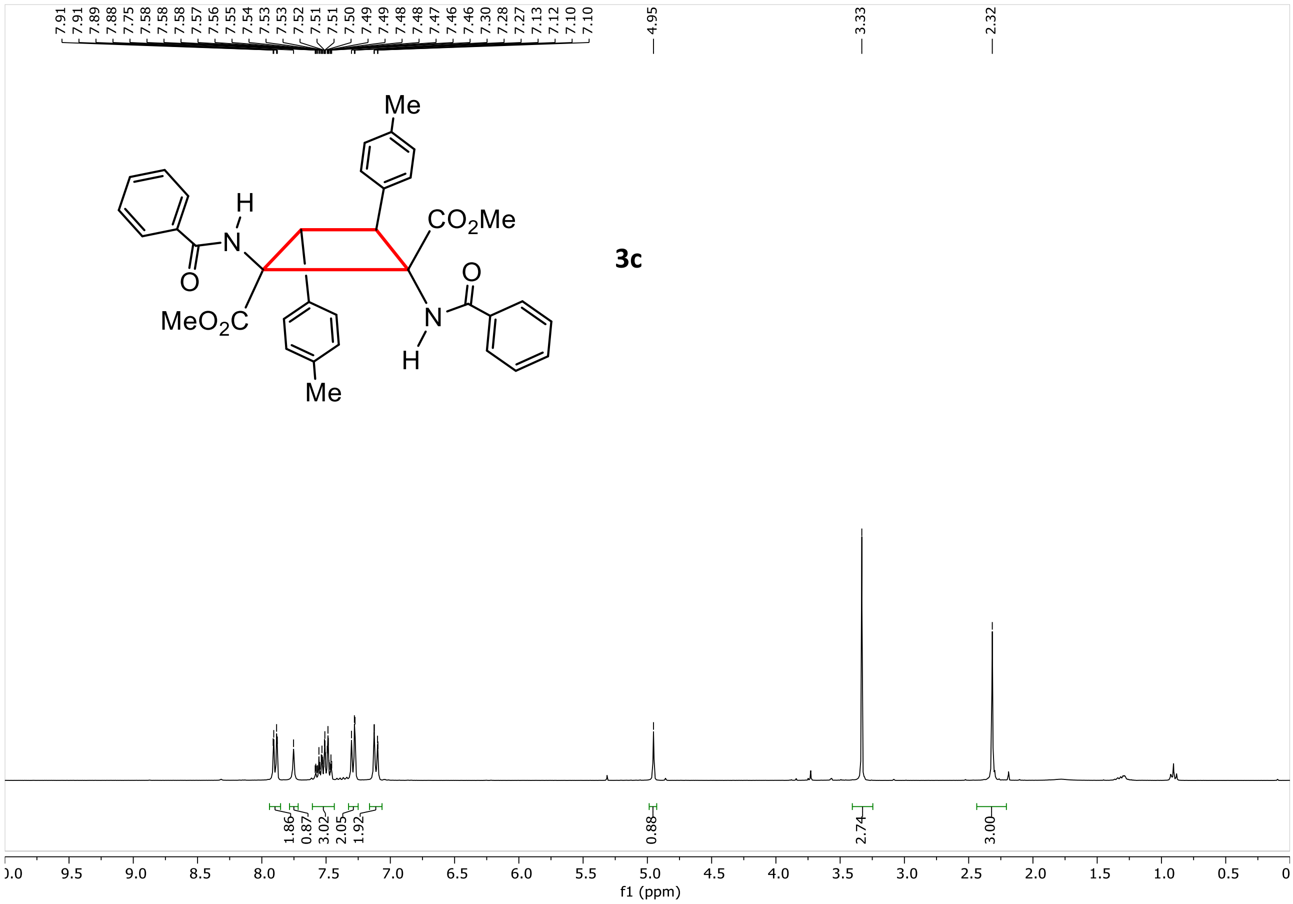

^1H NMR ($CDCl_3$, 300.13 MHz) of **3c**

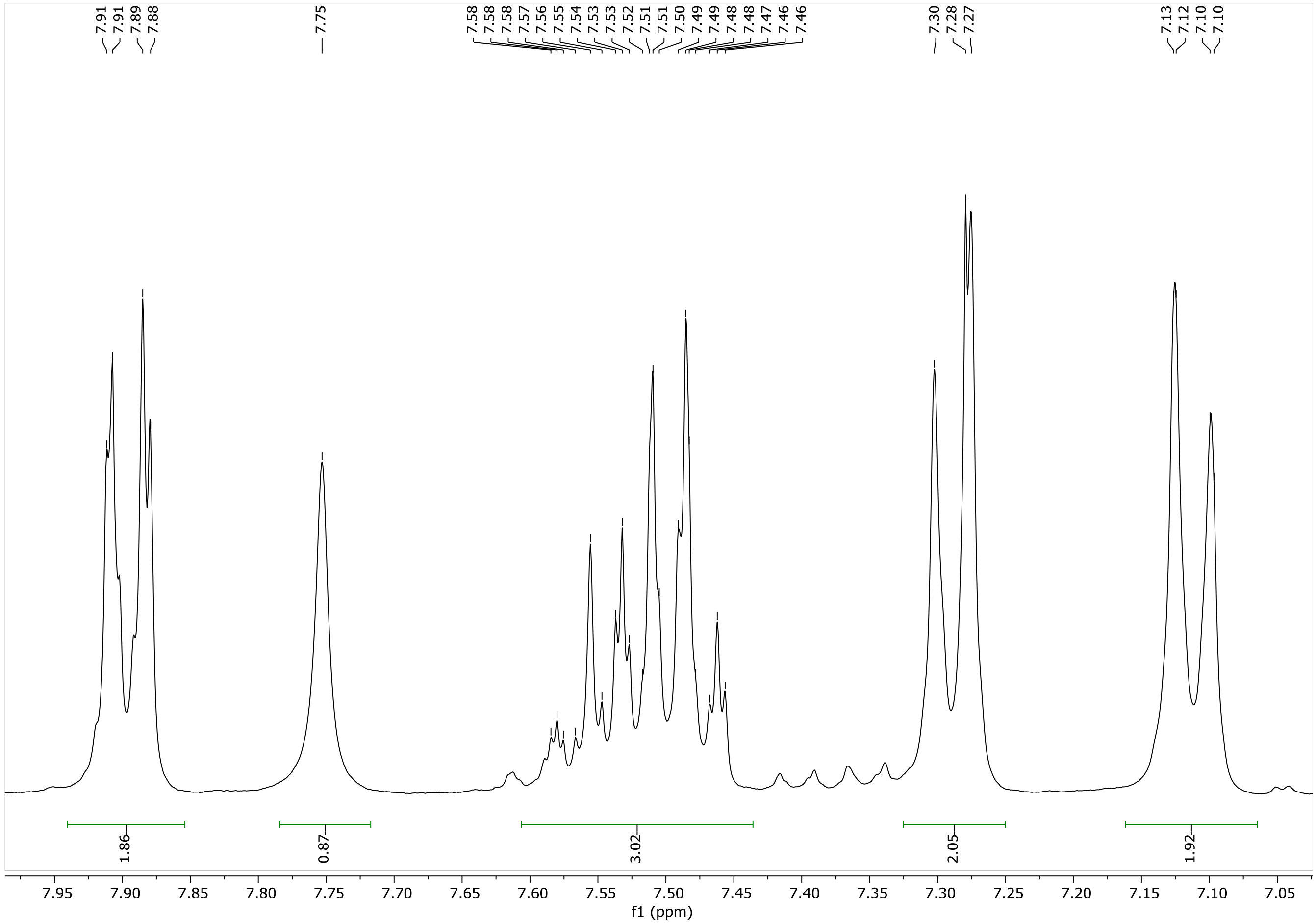

^1H NMR of **3c** (zoom aromatic region)

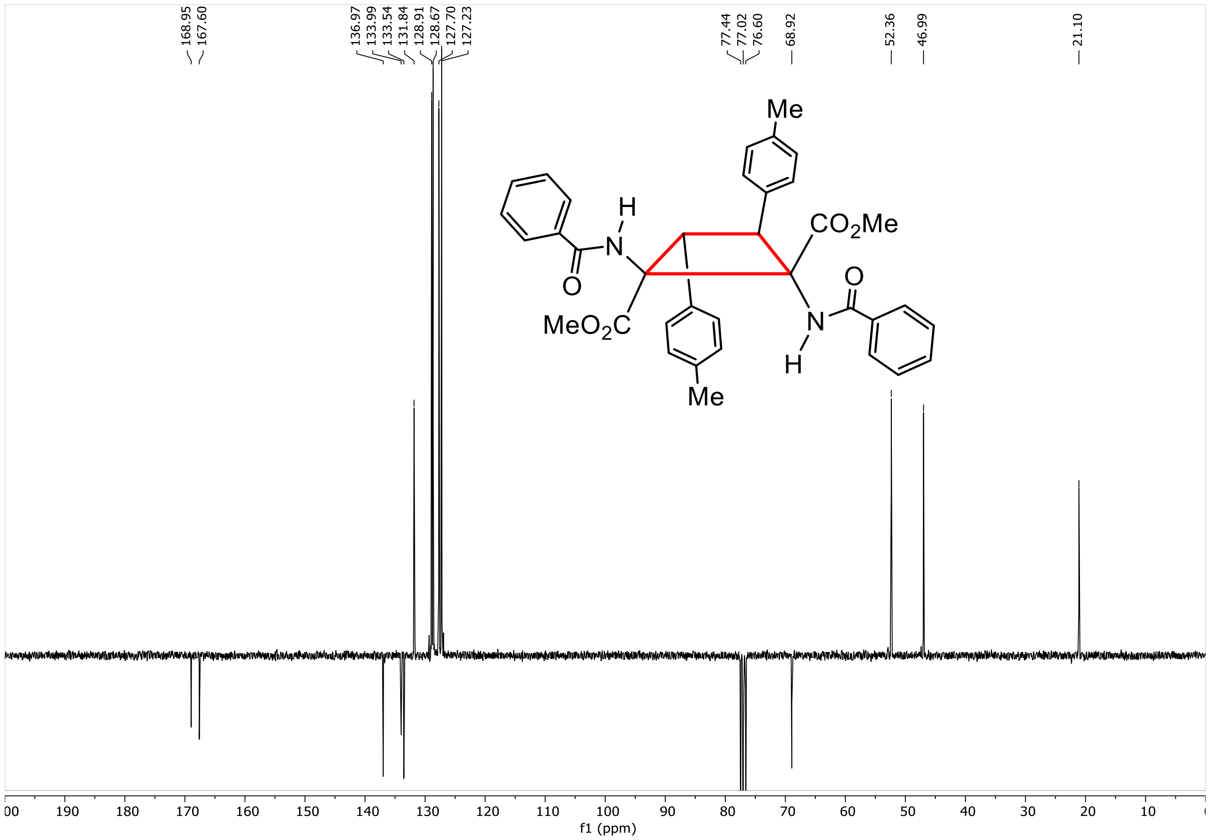

$^{13}C\{^{1}H\}$ (APT) NMR ($CDCl_3$, 75.5 MHz) of **3c**

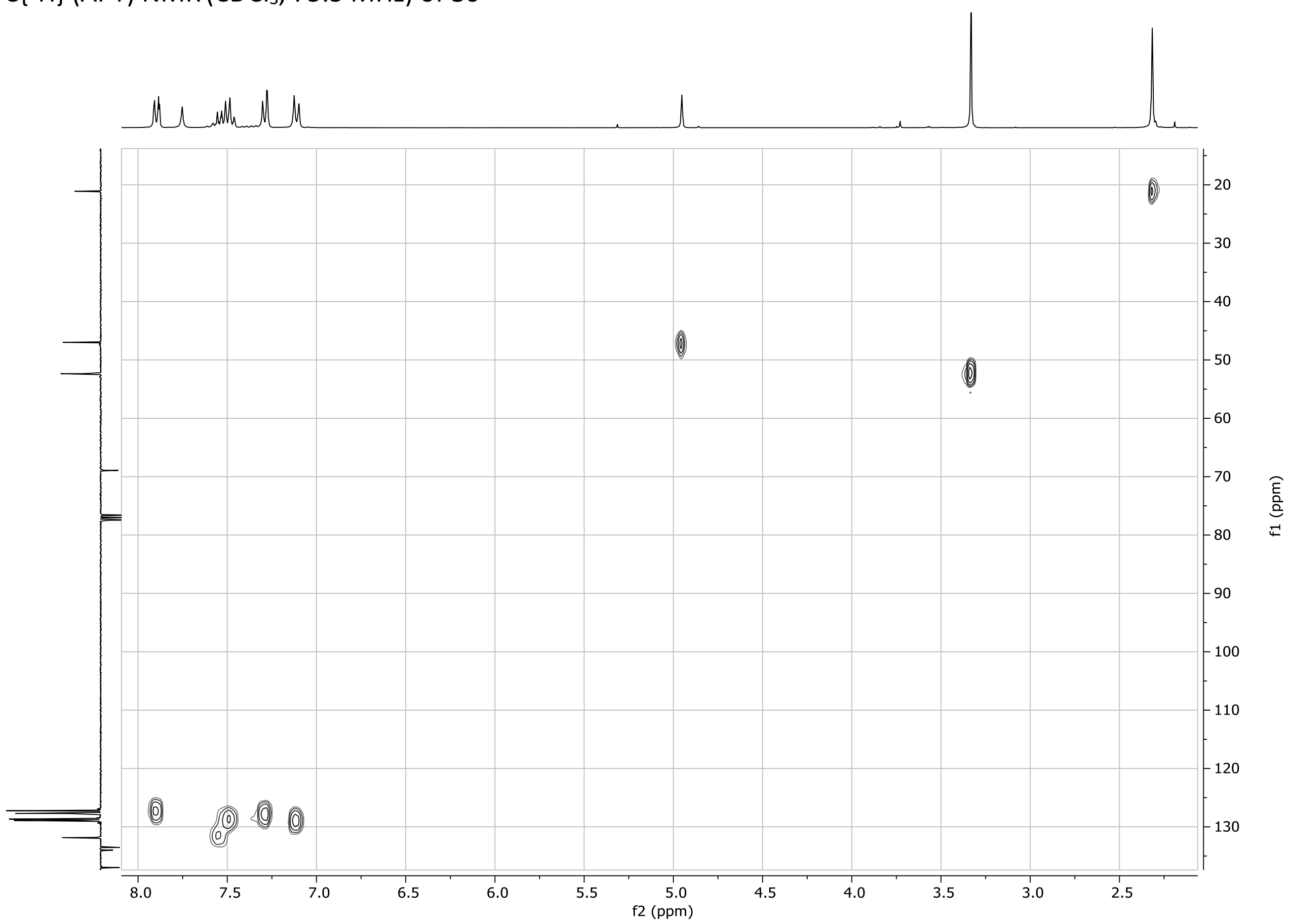

^{1}H-^{13}C HSQC correlation ($CDCl_3$) of **3c**

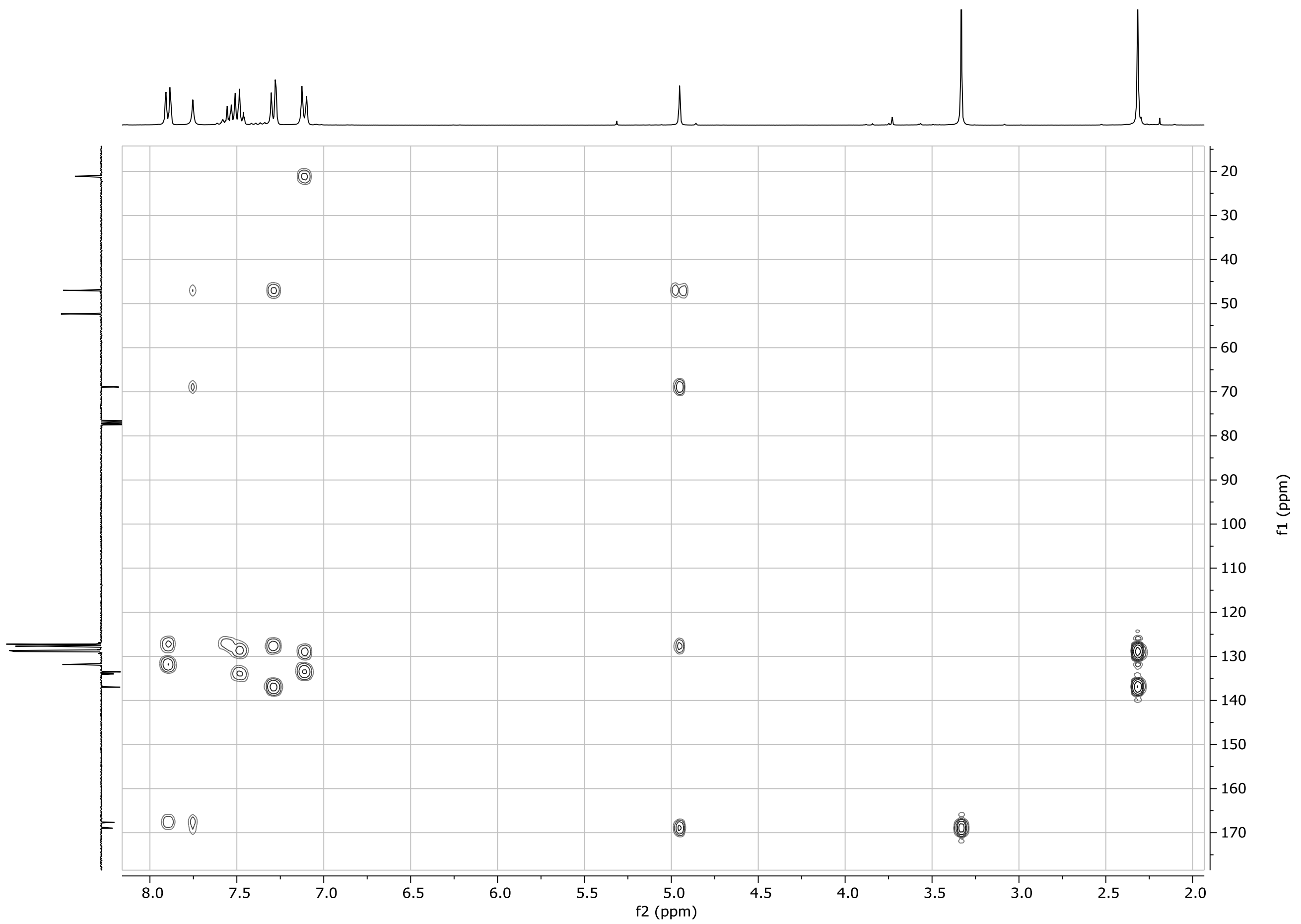

^{1}H-^{13}C HMBC correlation ($CDCl_3$) of **3c**

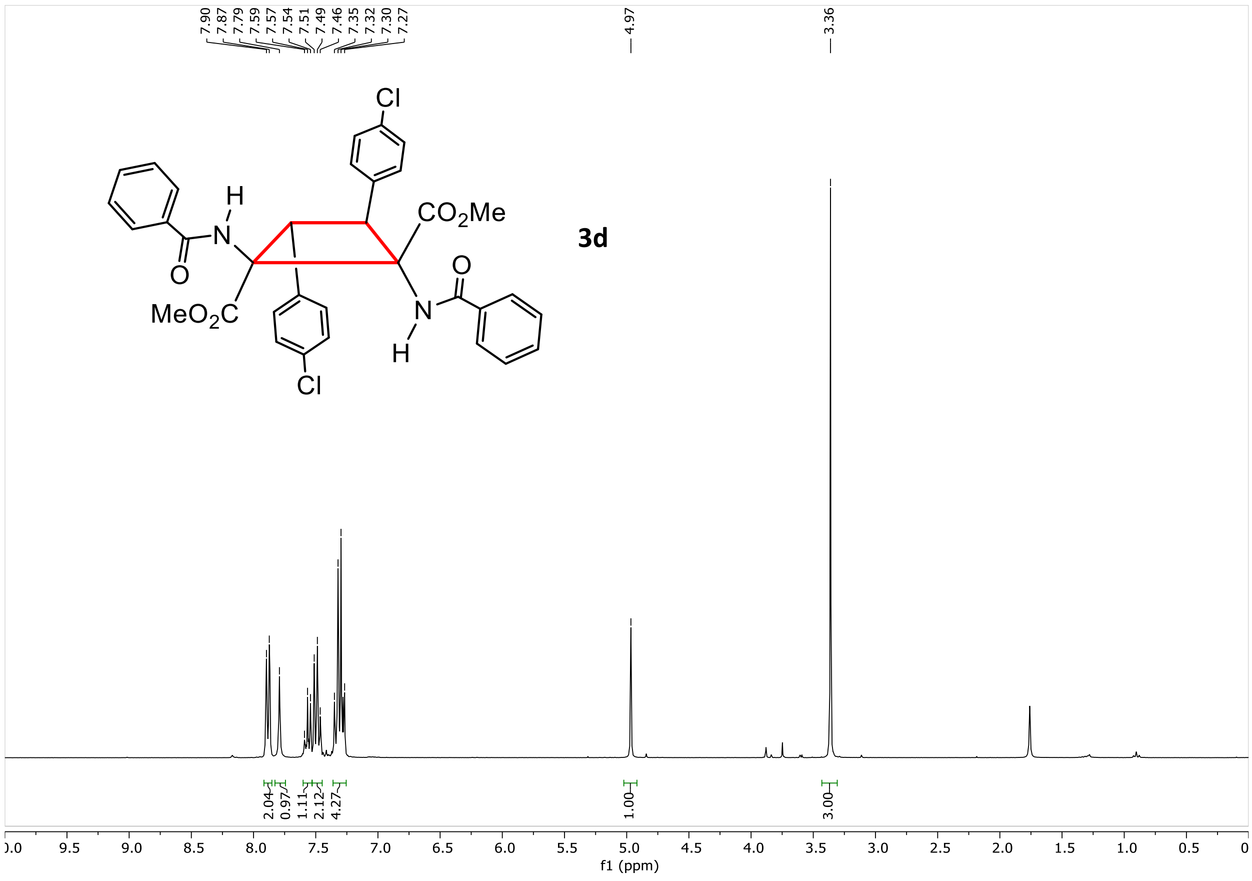

^{1}H NMR ($CDCl_3$, 300.13 MHz) of **3d**

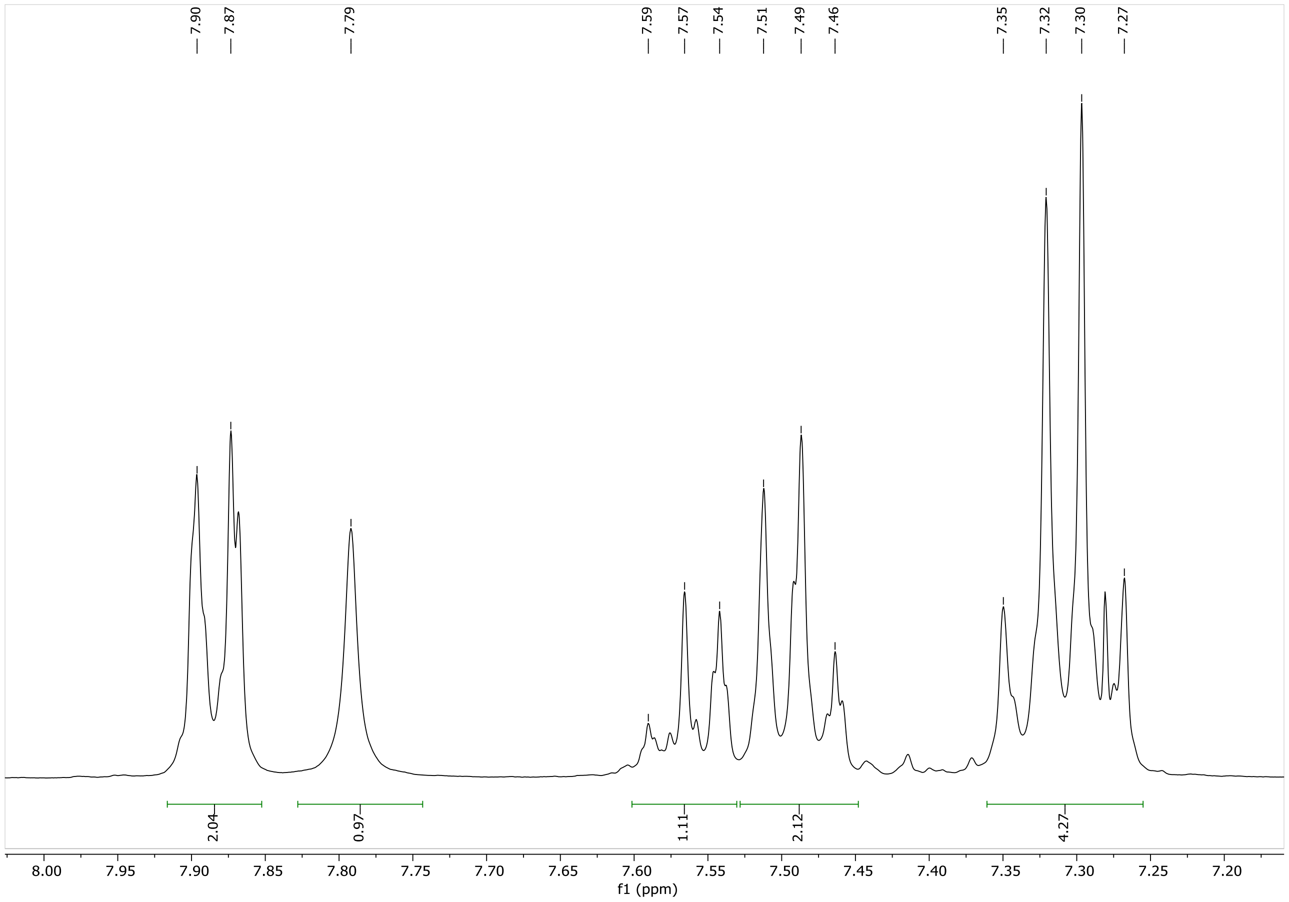

^{1}H NMR of **3d** (zoom aromatic region)

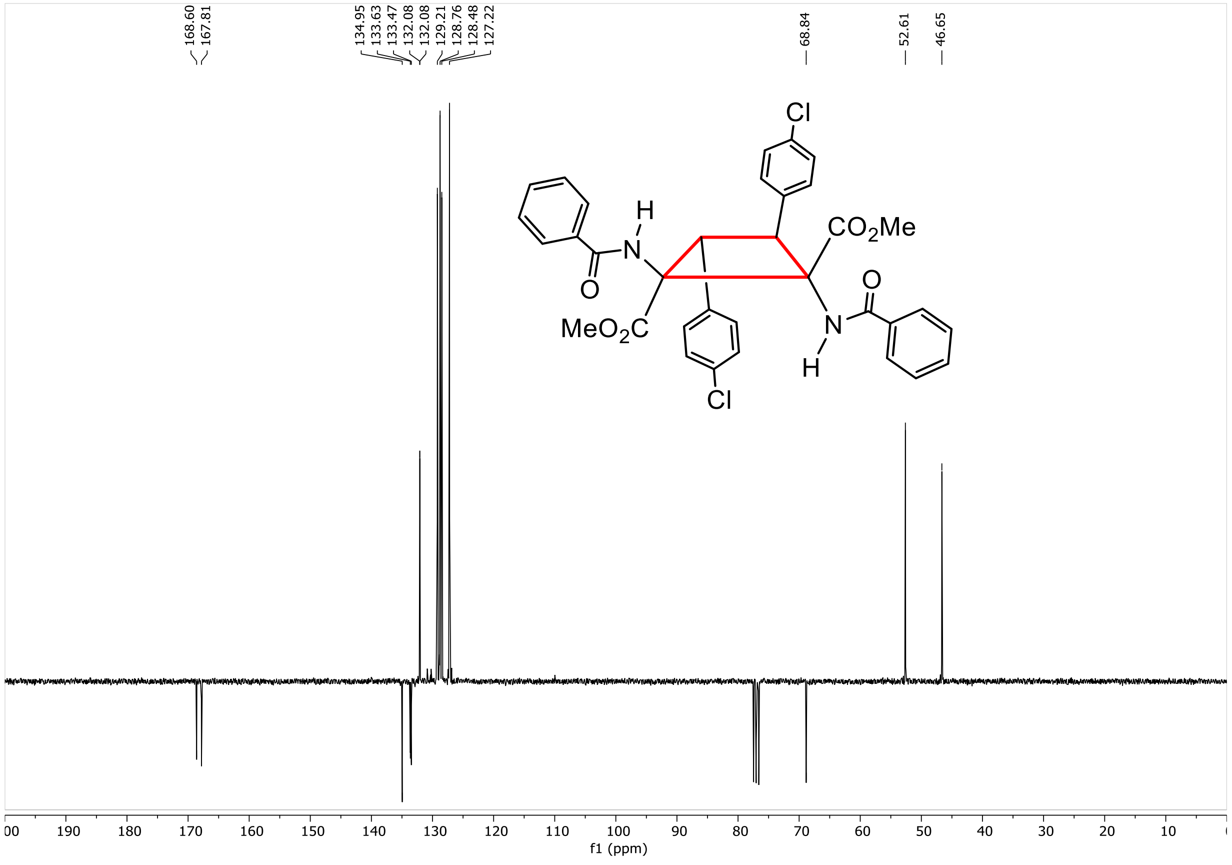

^{13}C{^{1}H} (APT) NMR ($CDCl_3$, 75.5 MHz) of **3d**

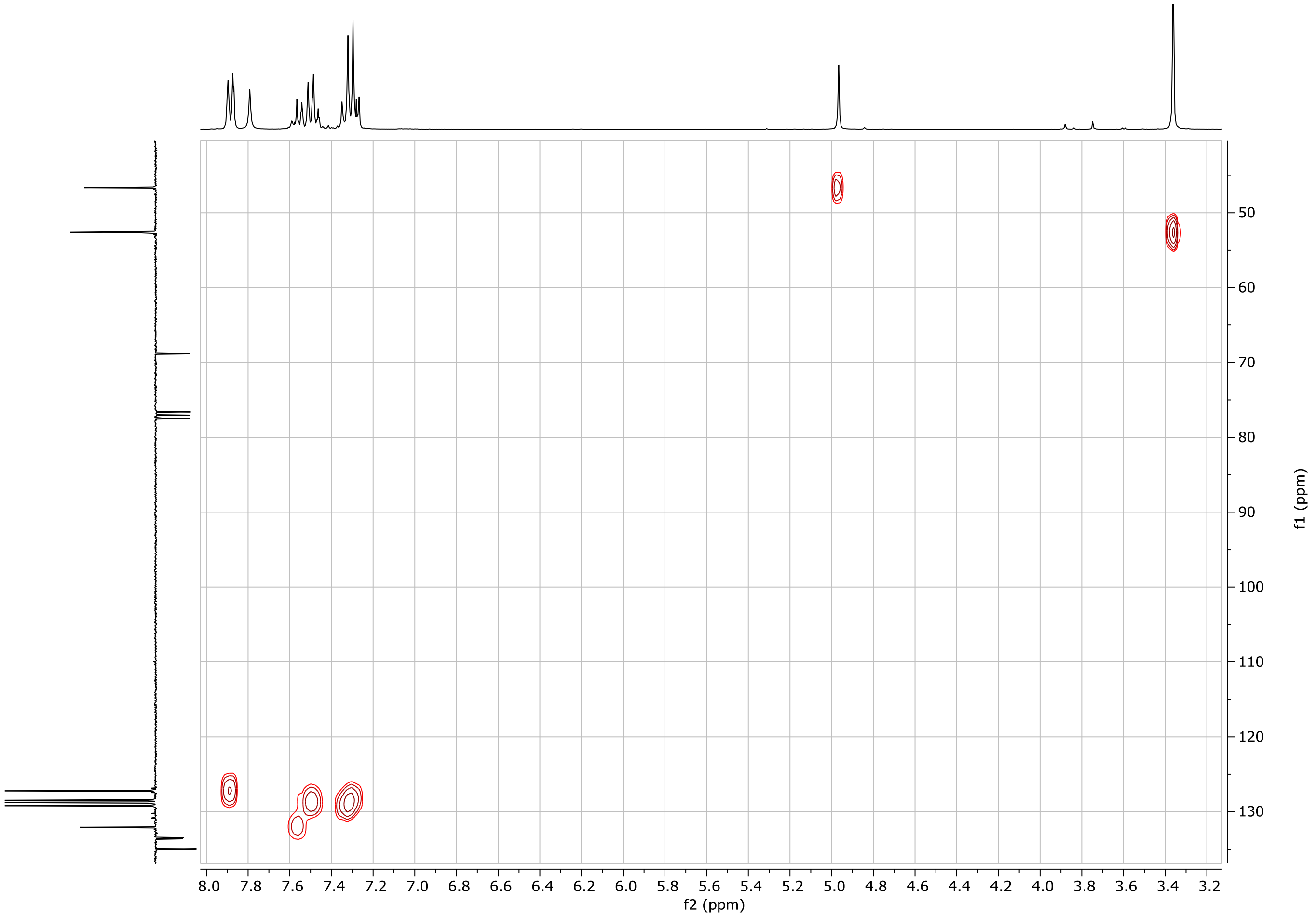

^{1}H-^{13}C HSQC correlation ($CDCl_3$) of **3d**

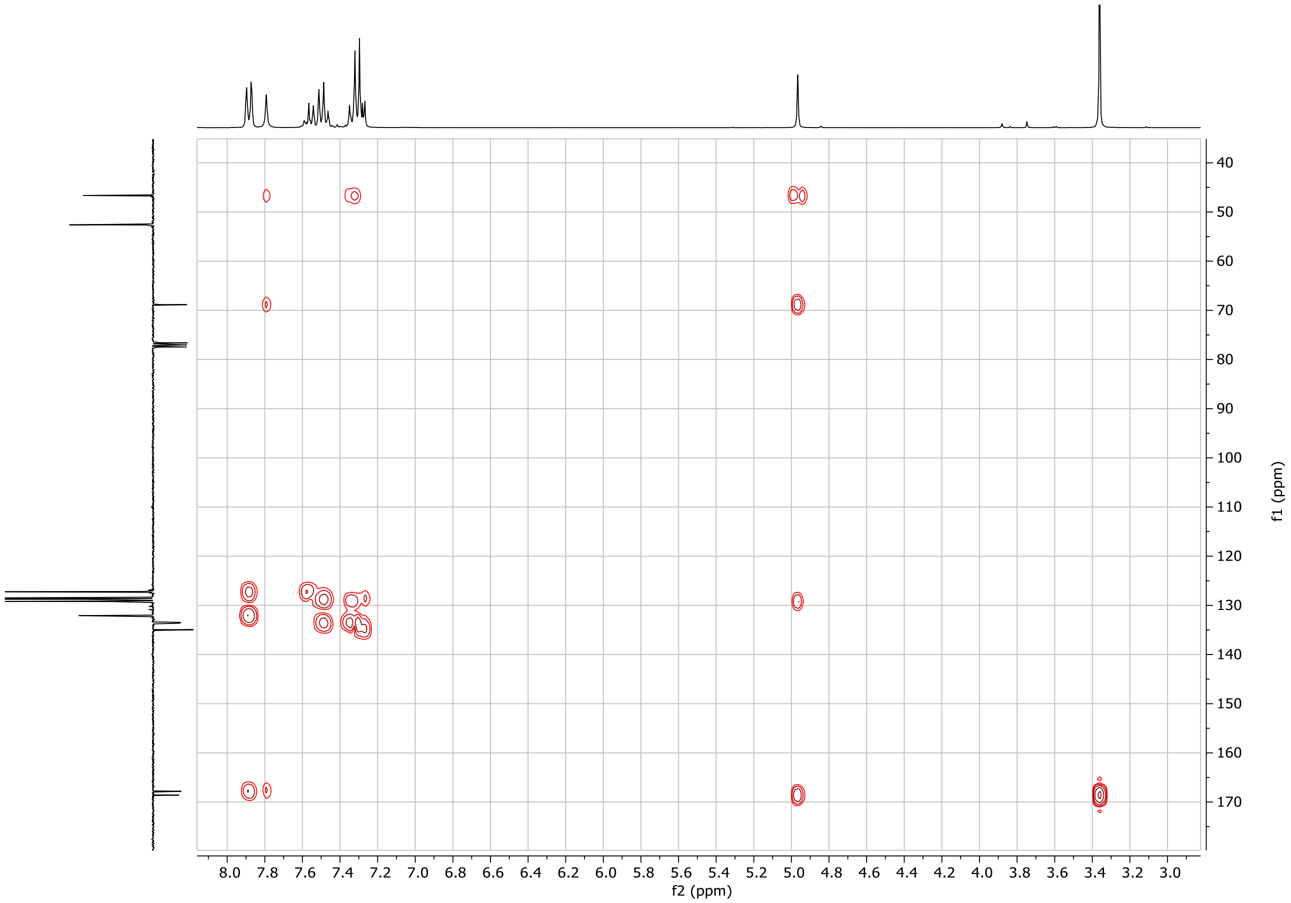

^{1}H-^{13}C HMBC correlation ($CDCl_3$) of **3d**

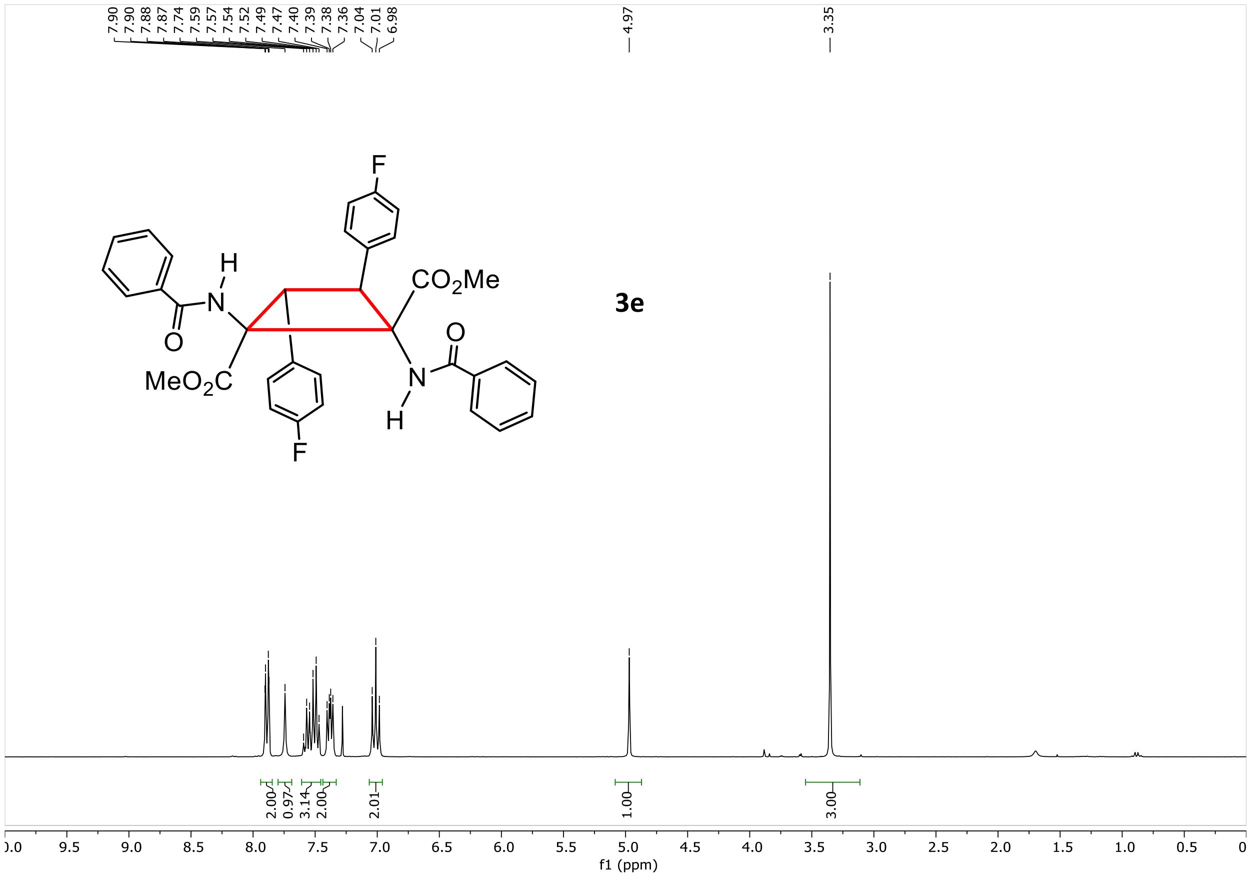

^{1}H NMR ($CDCl_3$, 300.13 MHz) of **3e**

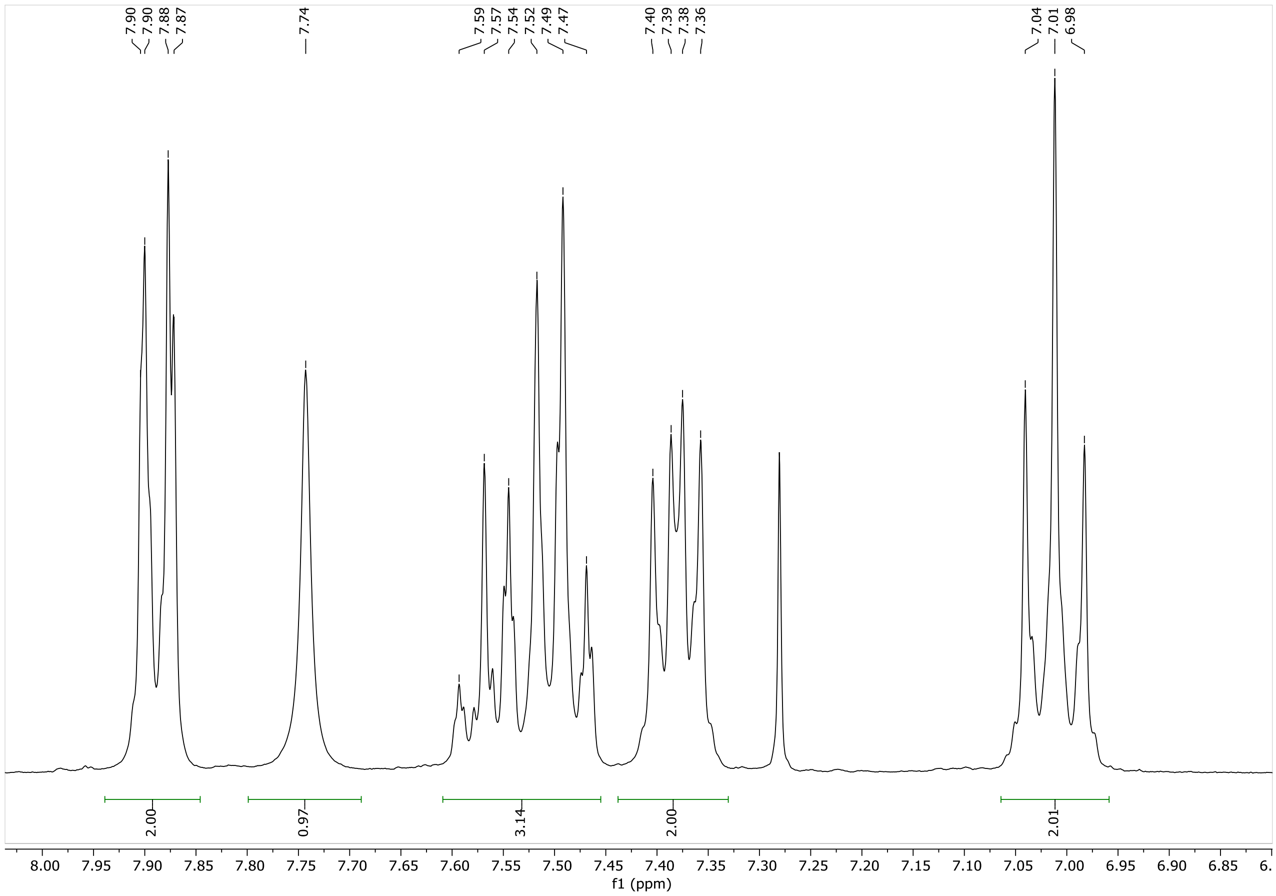

^{1}H NMR of **3e** (zoom aromatic region)

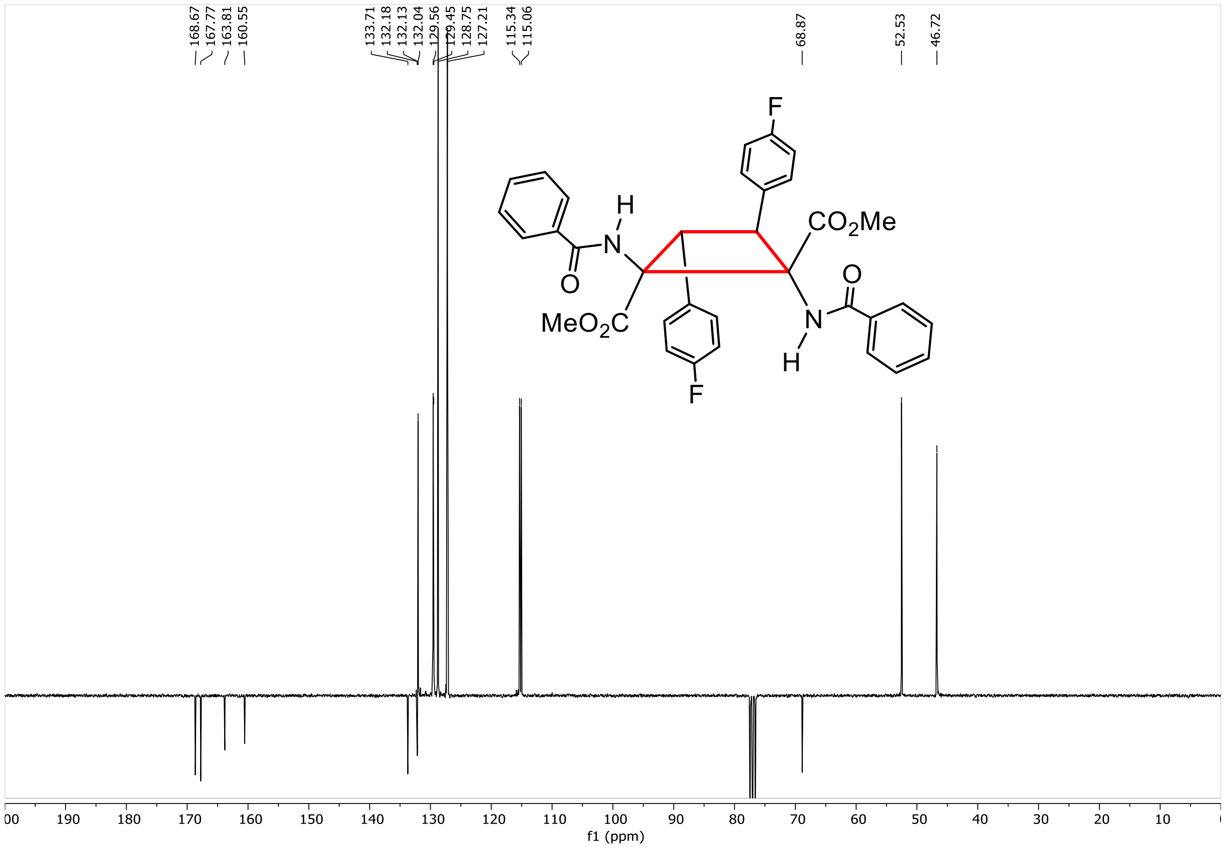

$^{13}C\{^1H\}$ (APT) NMR ($CDCl_3$, 75.5 MHz) of **3e**

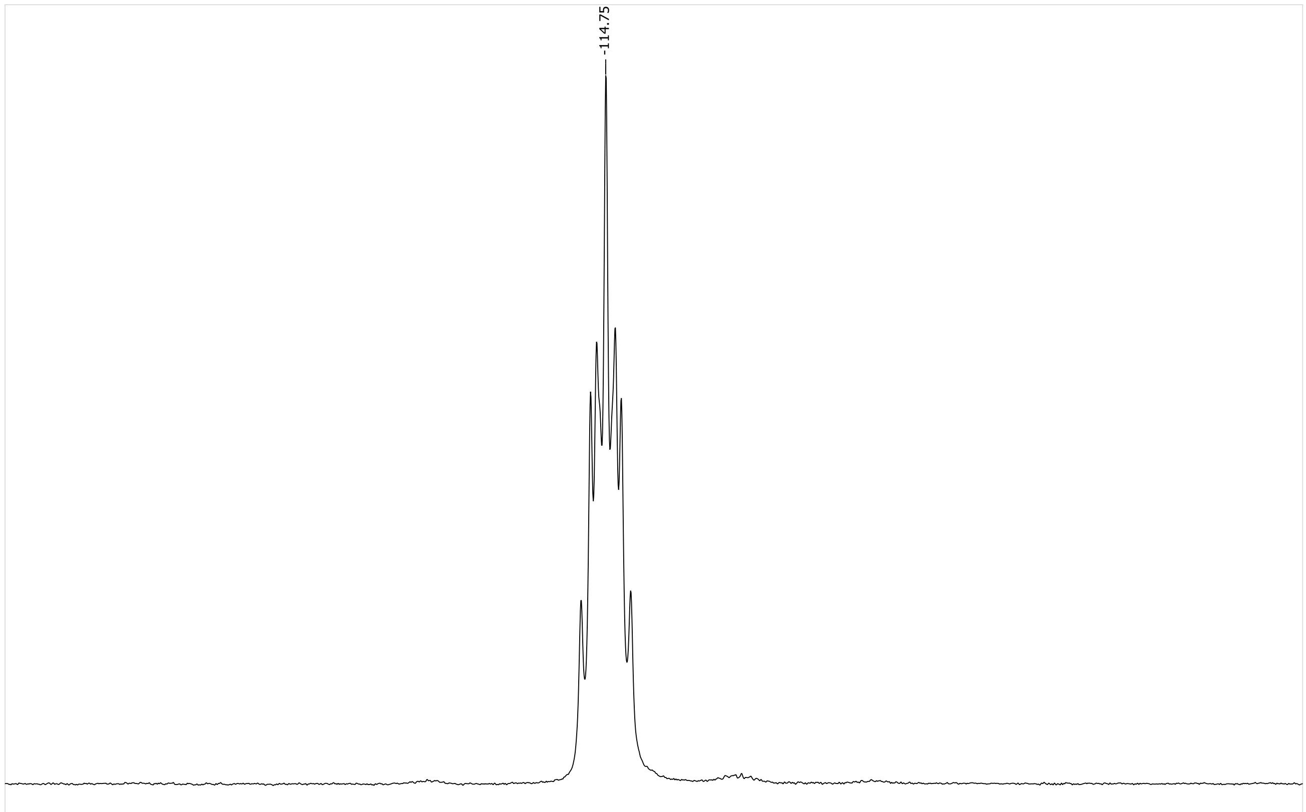

^{19}F NMR ($CDCl_3$, 282.6 MHz) of **3e**

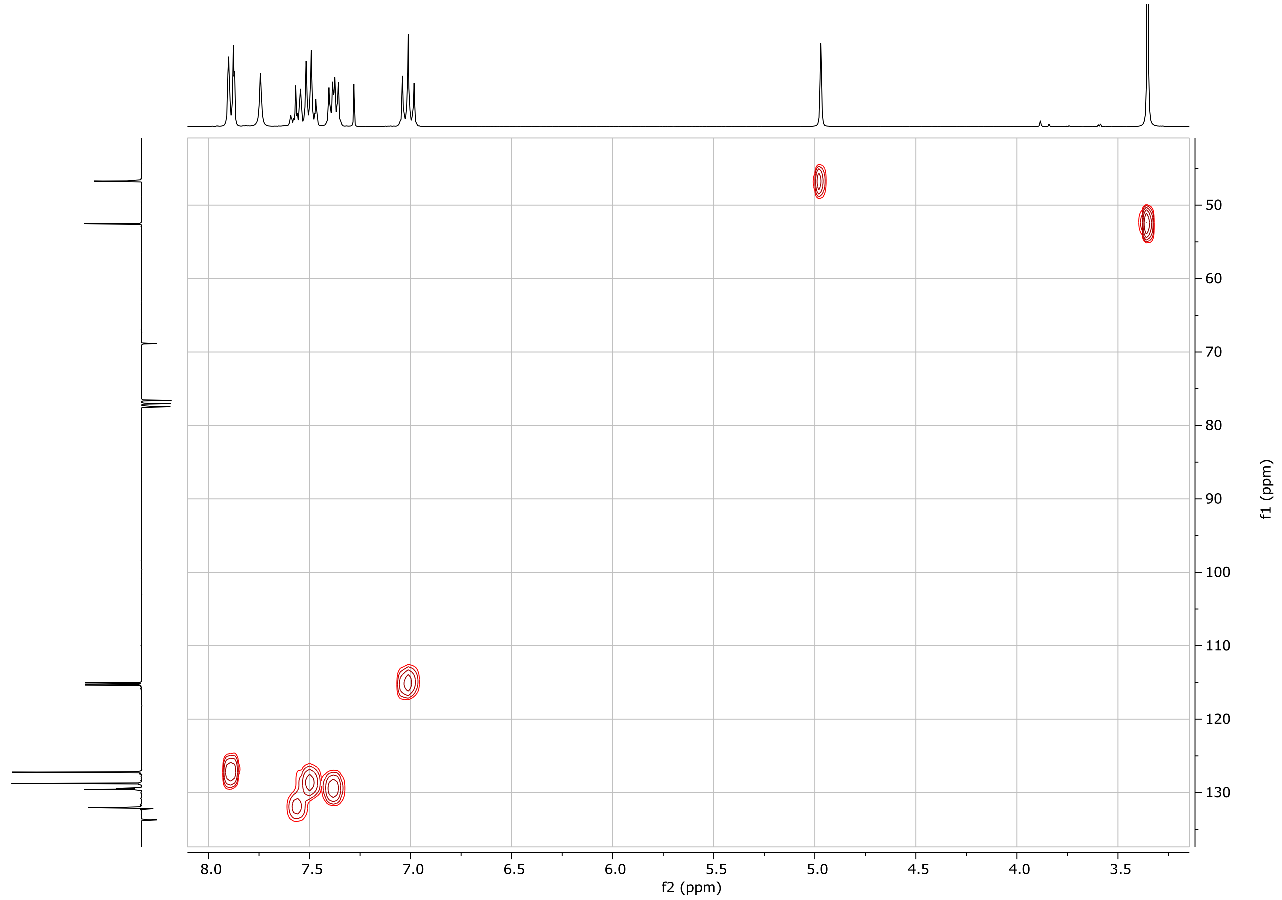

^{1}H-^{13}C HSQC correlation ($CDCl_3$) of **3e**

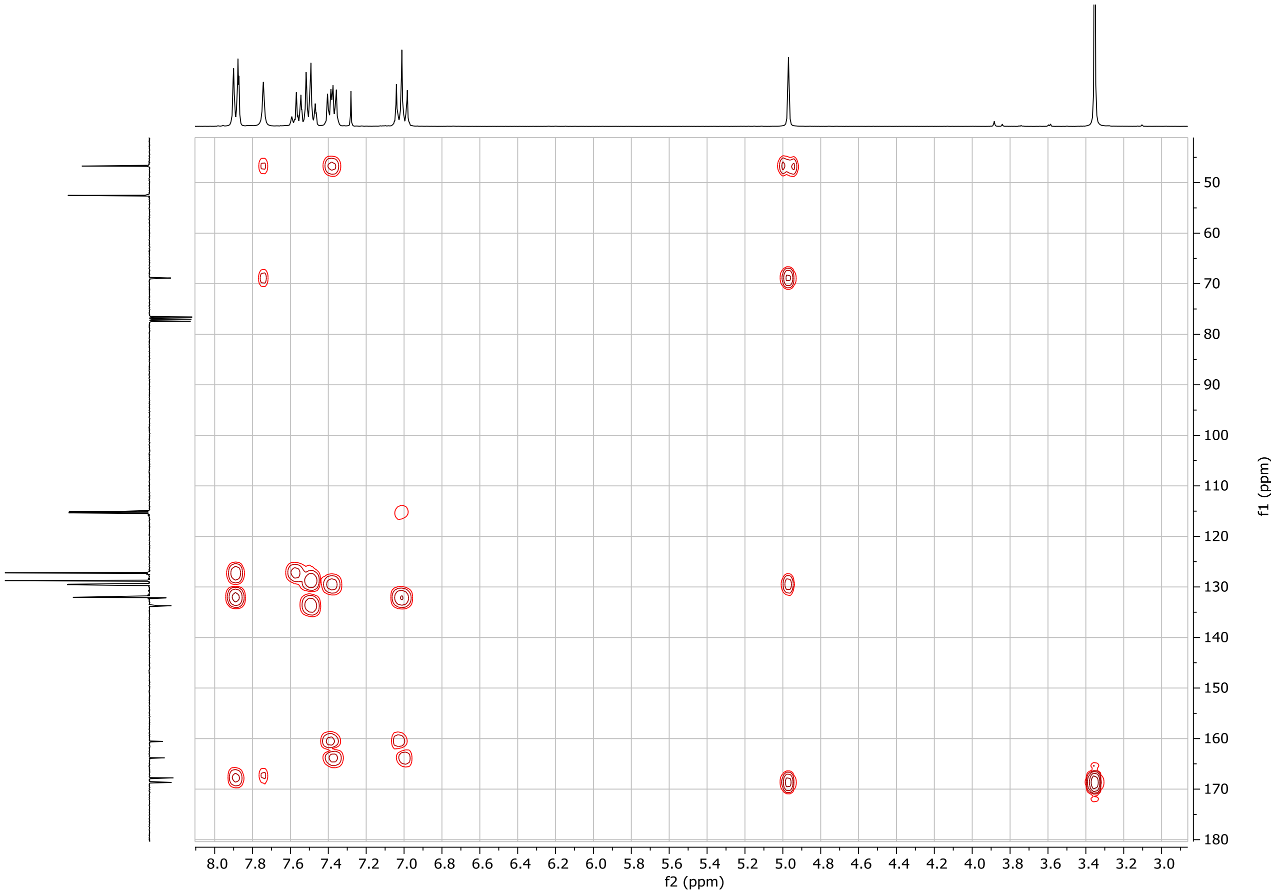

^{1}H-^{13}C HMBC correlation ($CDCl_3$) of **3e**

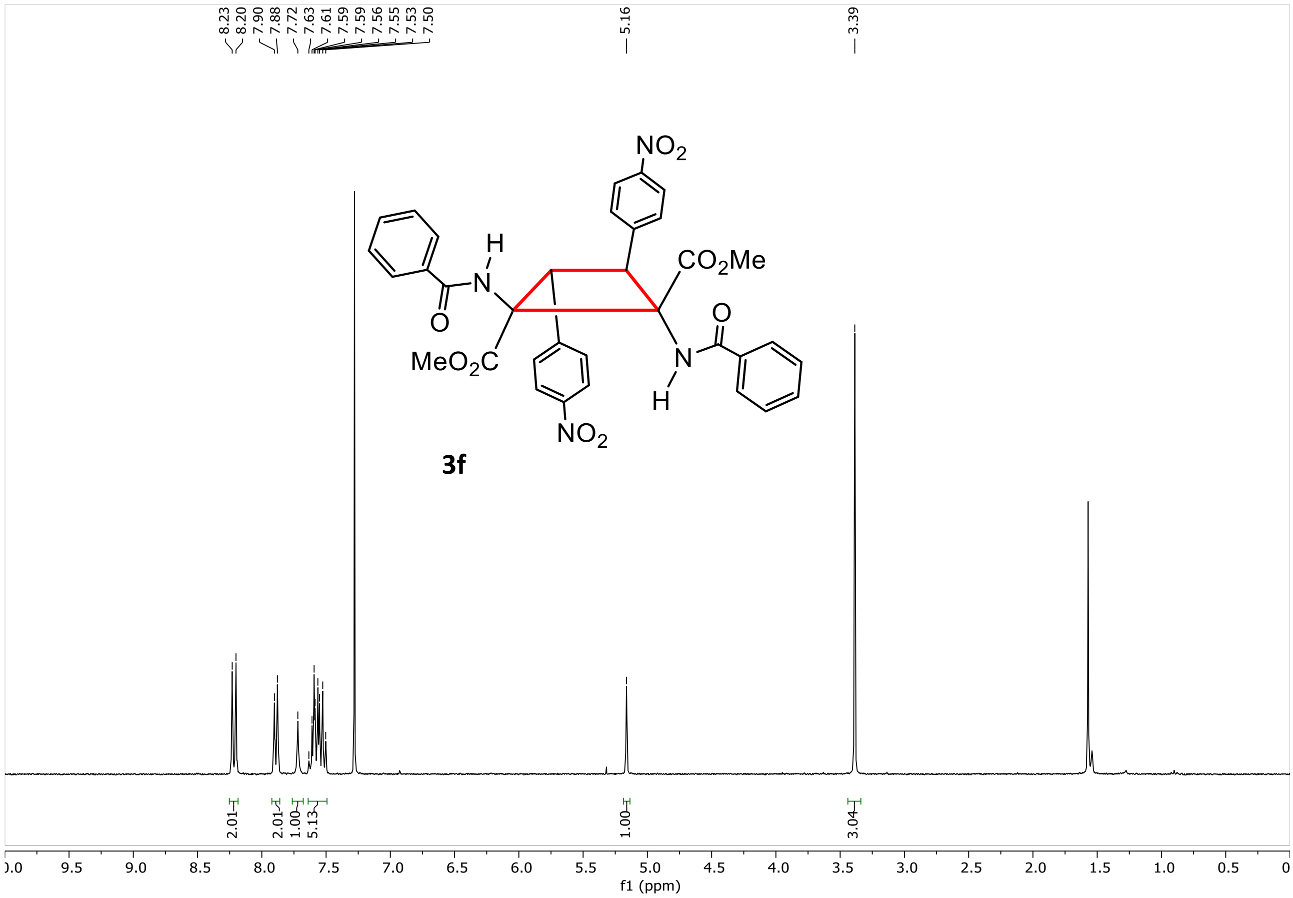

^{1}H NMR ($CDCl_3$, 300.13 MHz) of **3f**

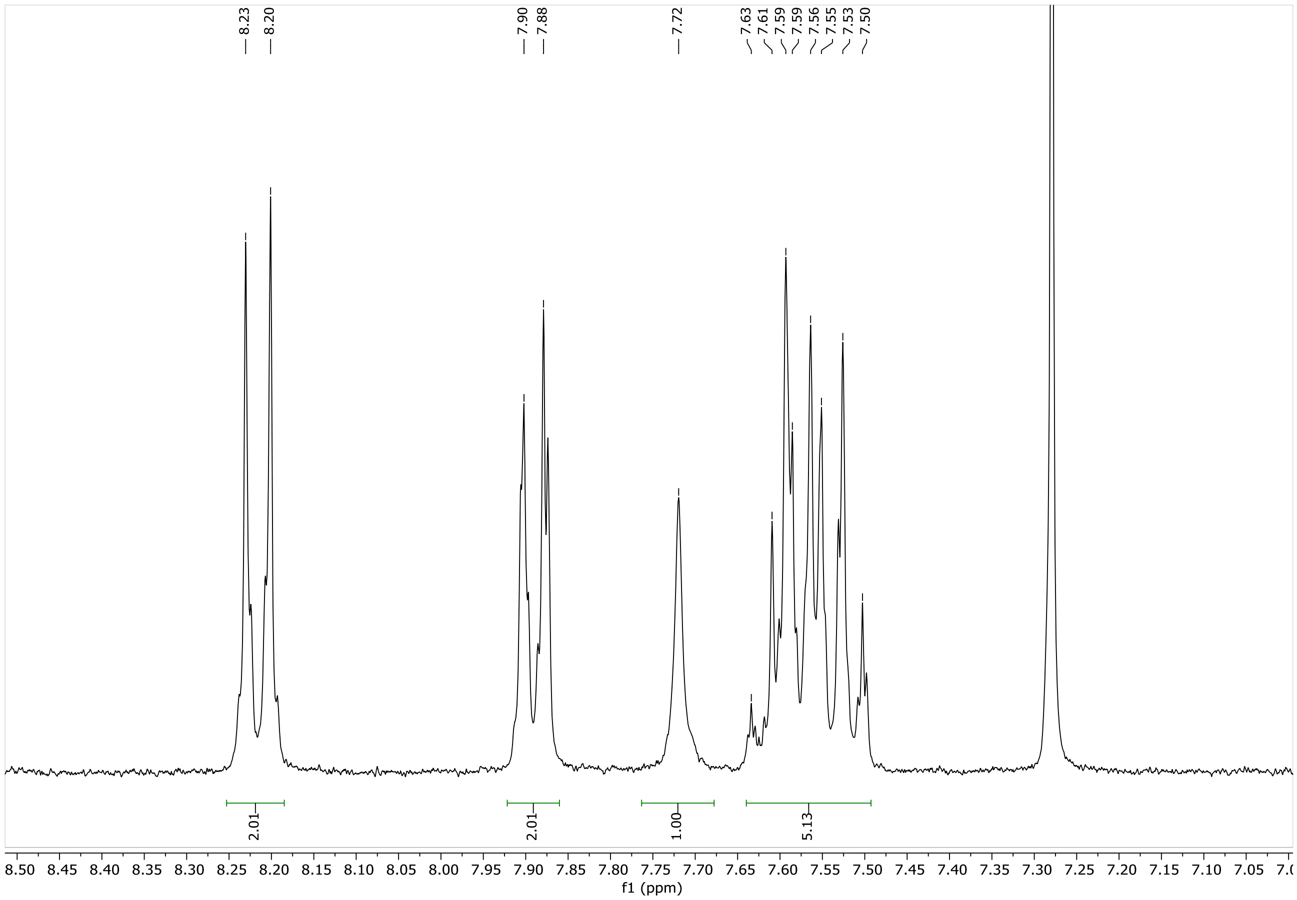

^{1}H NMR of **3f** (zoom aromatic region)

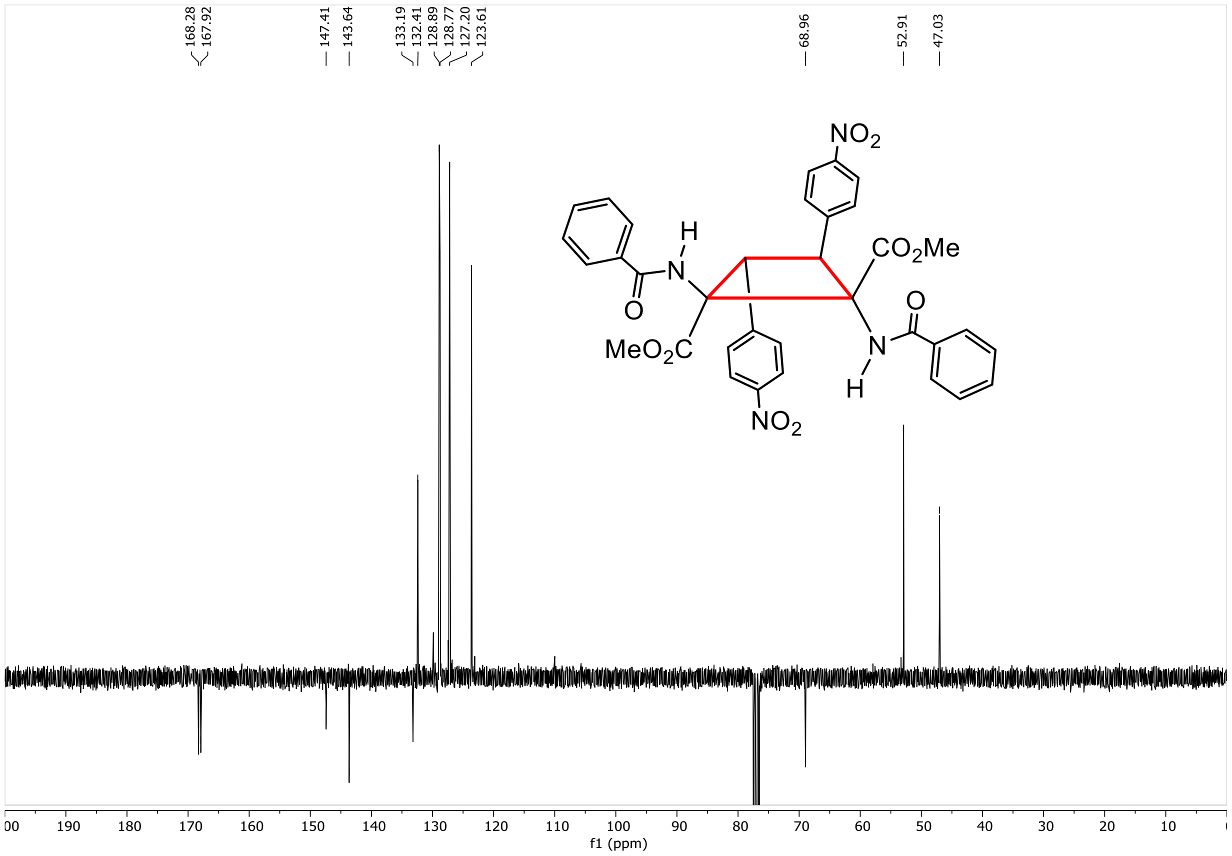

$^{13}C\{^{1}H\}$ (APT) NMR ($CDCl_3$, 75.5 MHz) of **3f**

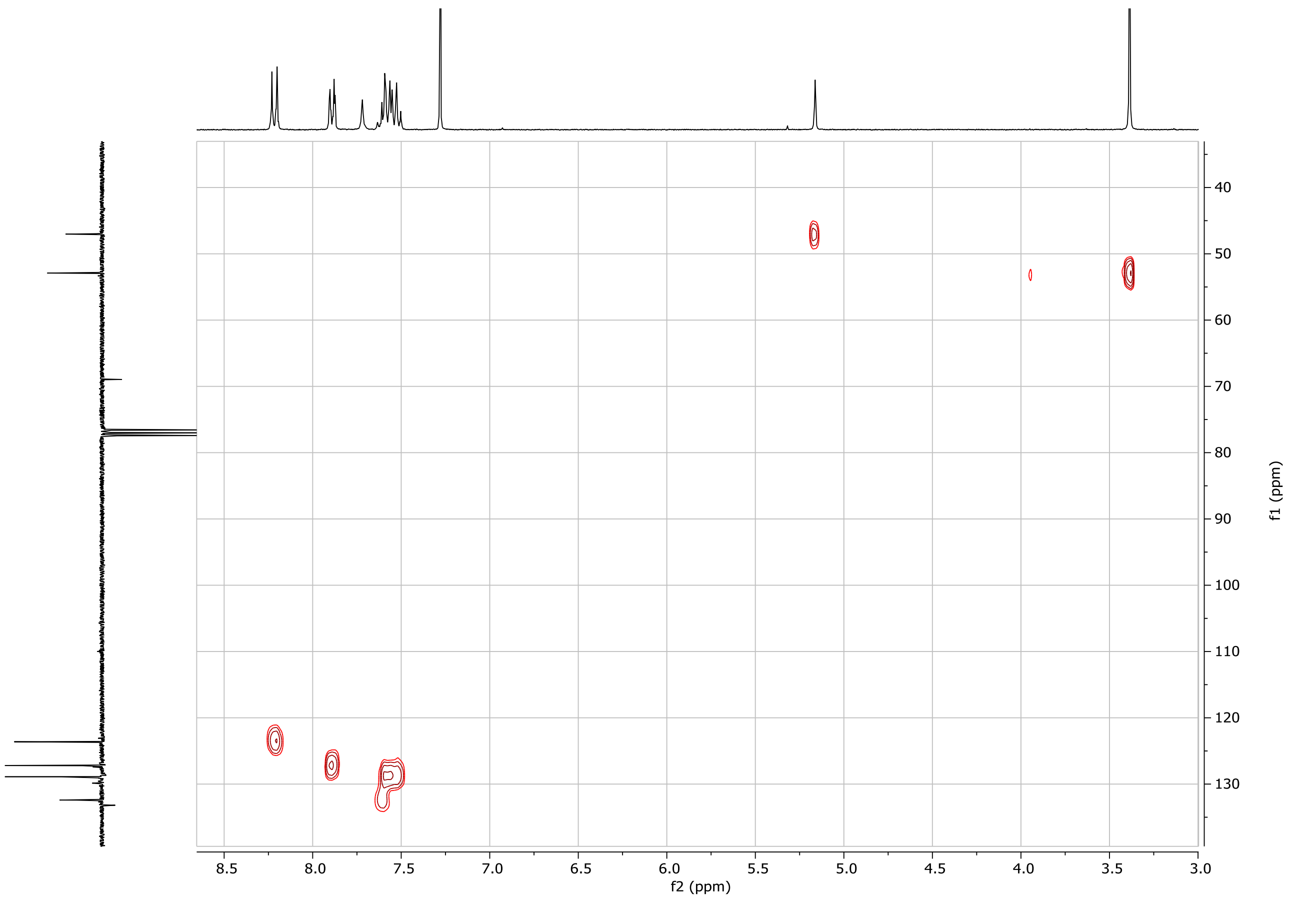

^{1}H-^{13}C HSQC correlation ($CDCl_3$) of **3f**

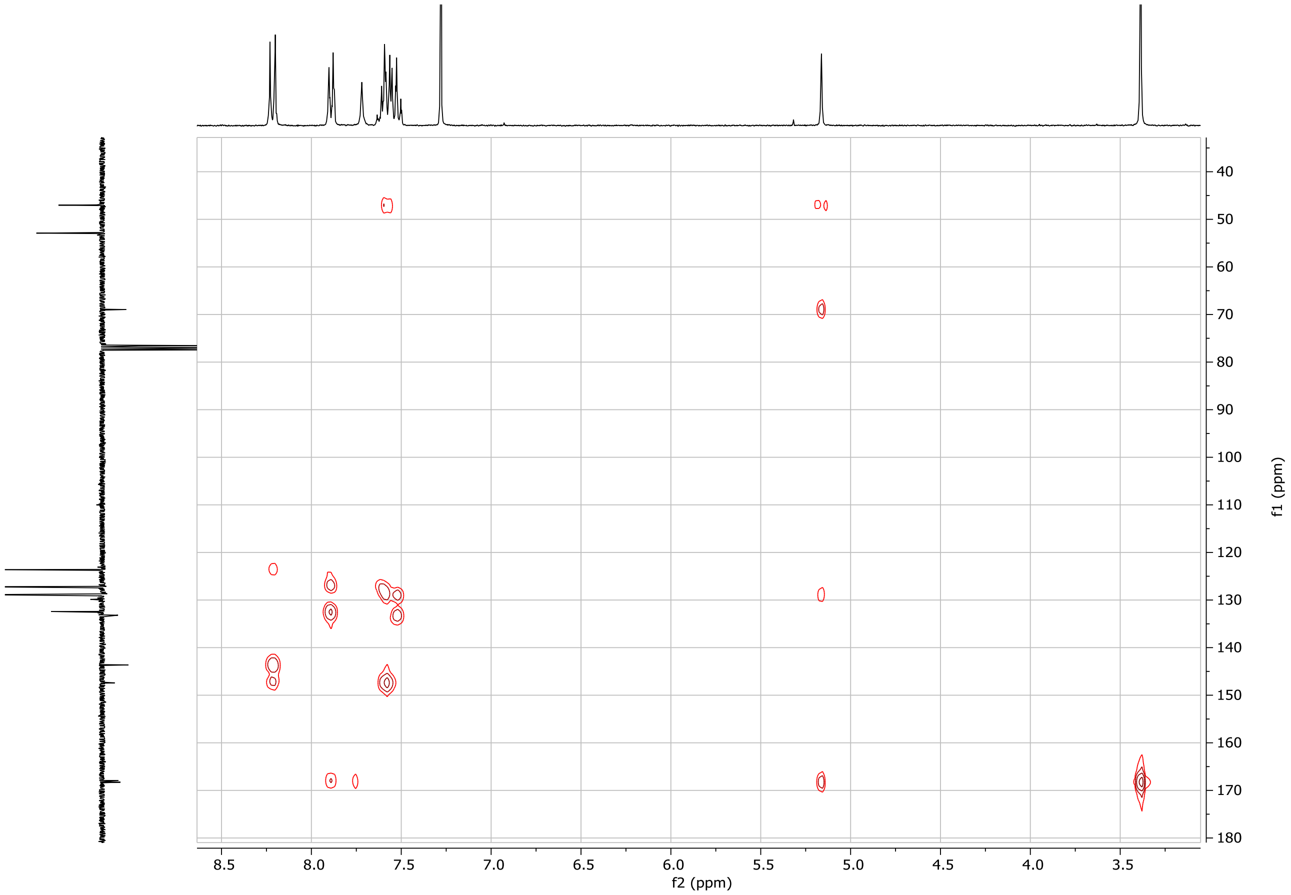

^{1}H-^{13}C HMBC correlation (CDCl$_3$) of **3f**

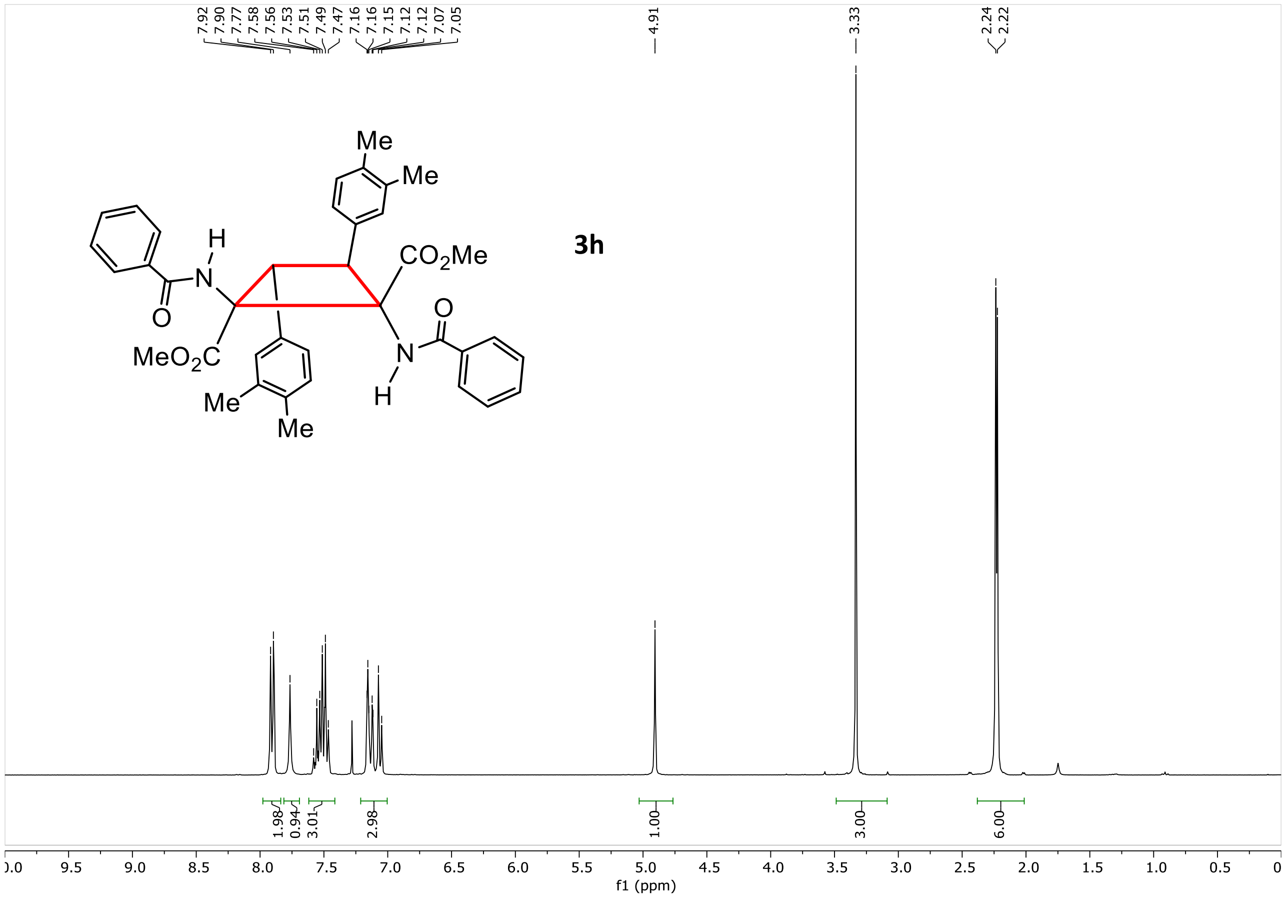

^{1}H NMR (CDCl$_3$, 300.13 MHz) of **3h**

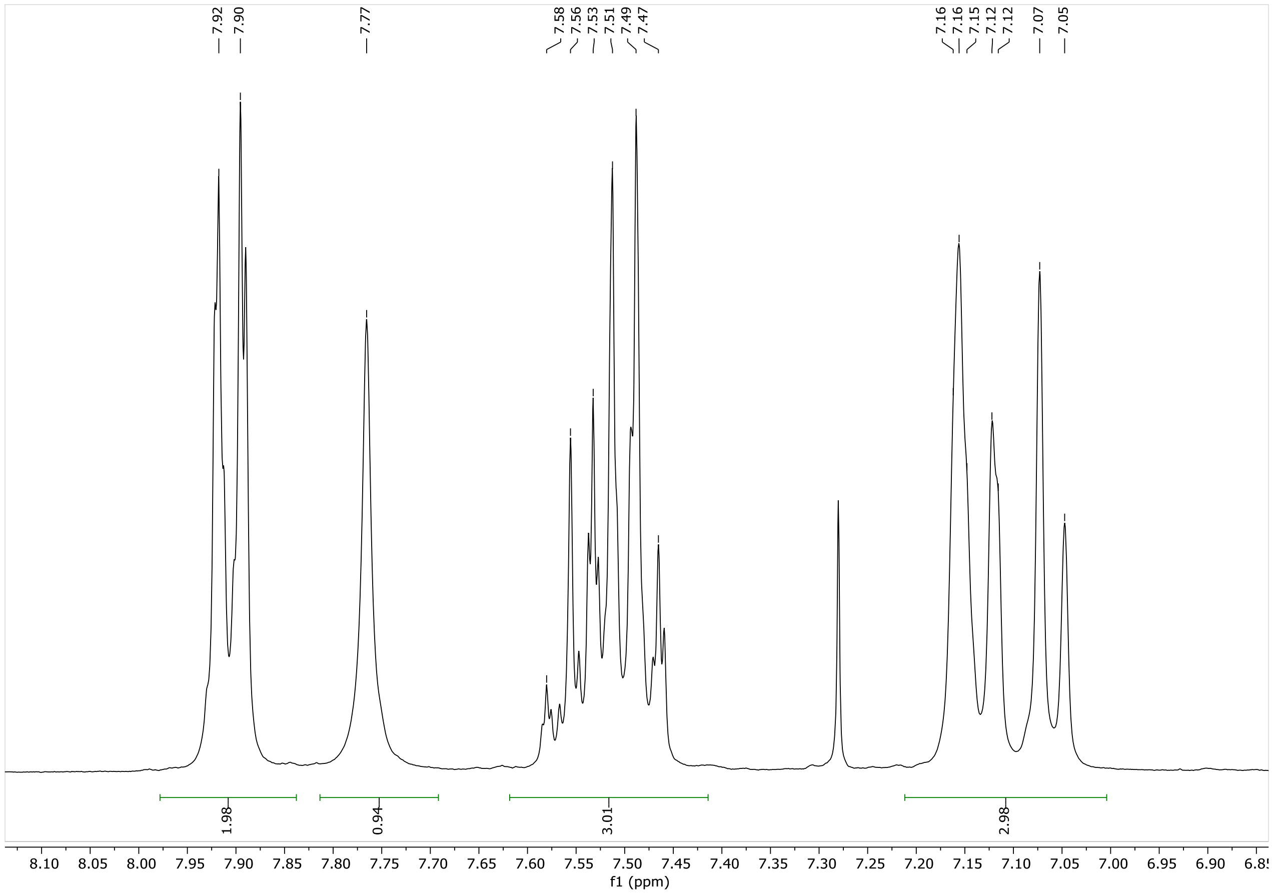

^{1}H NMR of **3h** (zoom aromatic region)

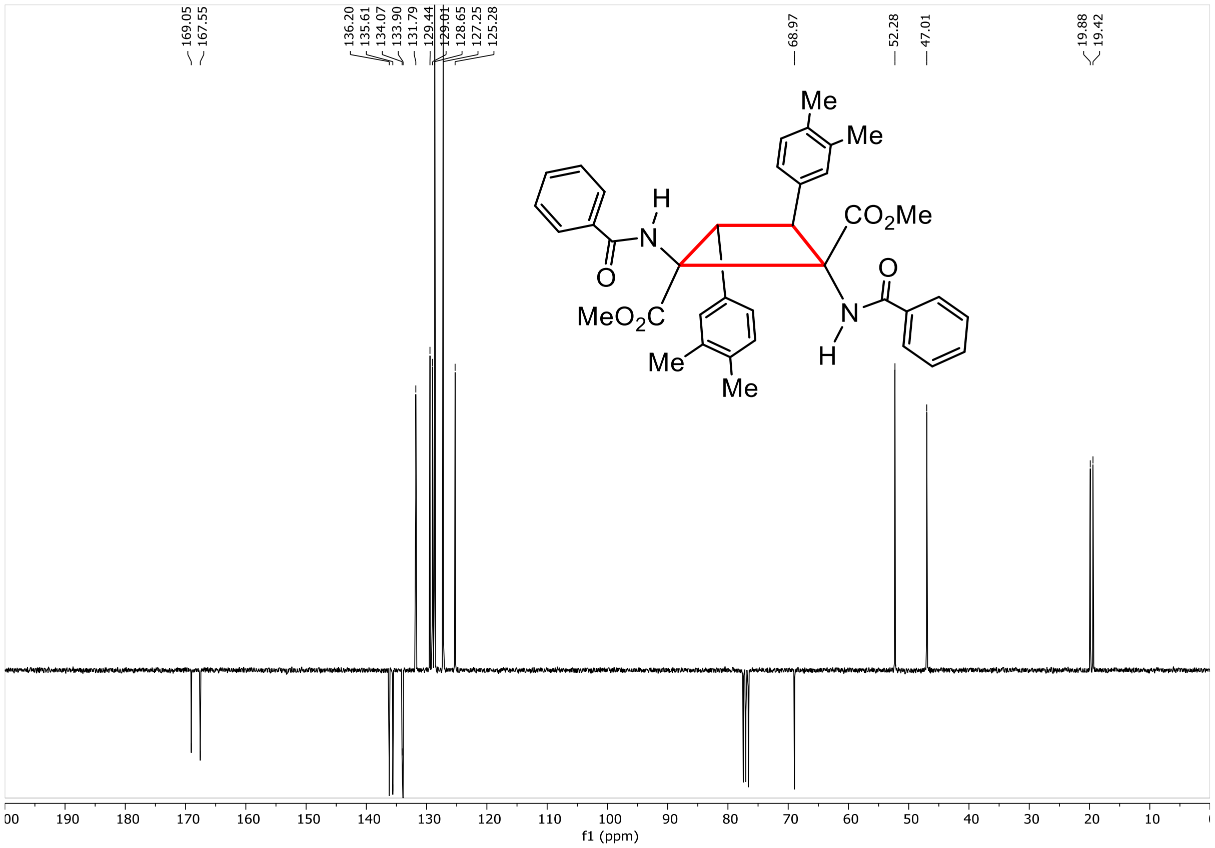

$^{13}C\{^1H\}$ (APT) NMR ($CDCl_3$, 75.5 MHz) of **3h**

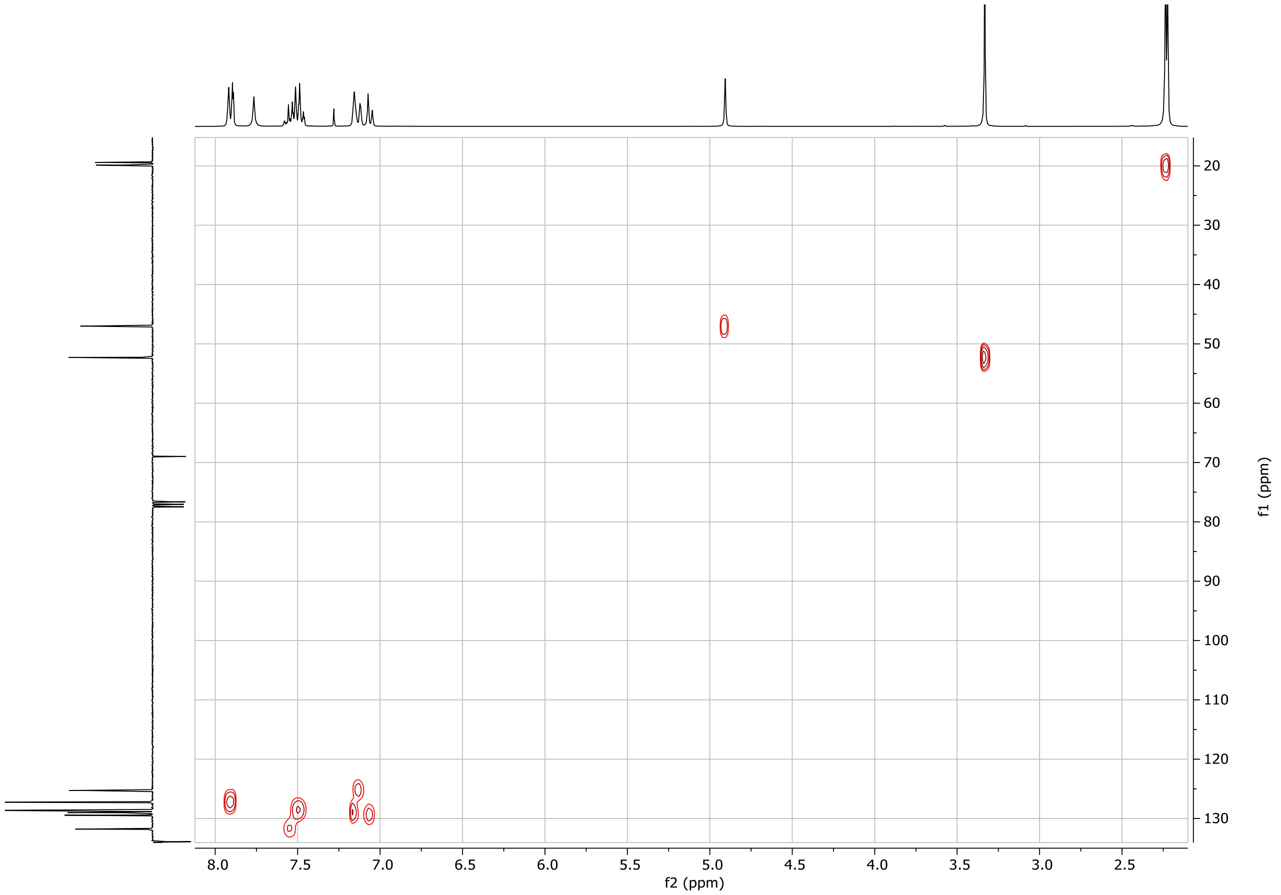

^{1}H-^{13}C HSQC correlation ($CDCl_3$) of **3h**

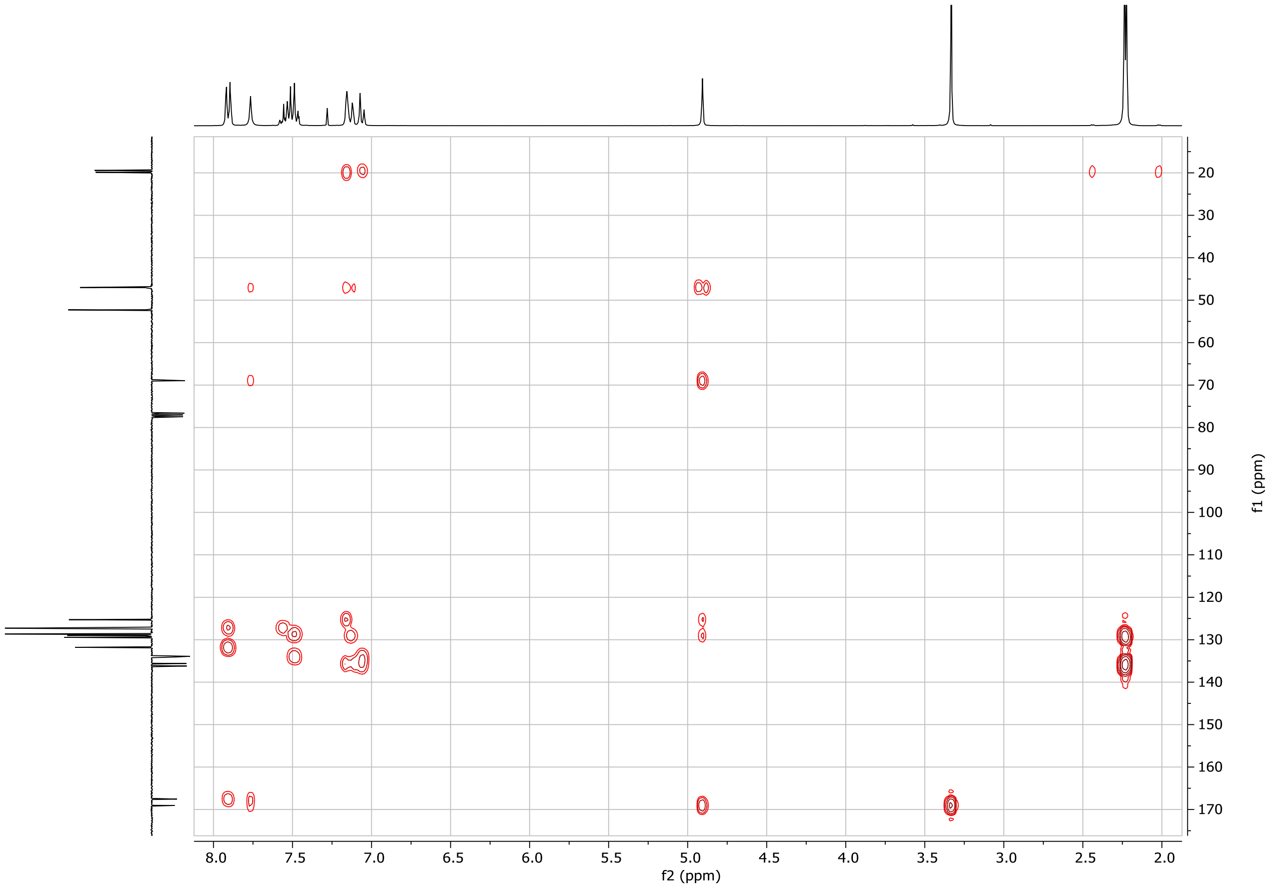

^{1}H-^{13}C HMBC correlation ($CDCl_3$) of **3h**

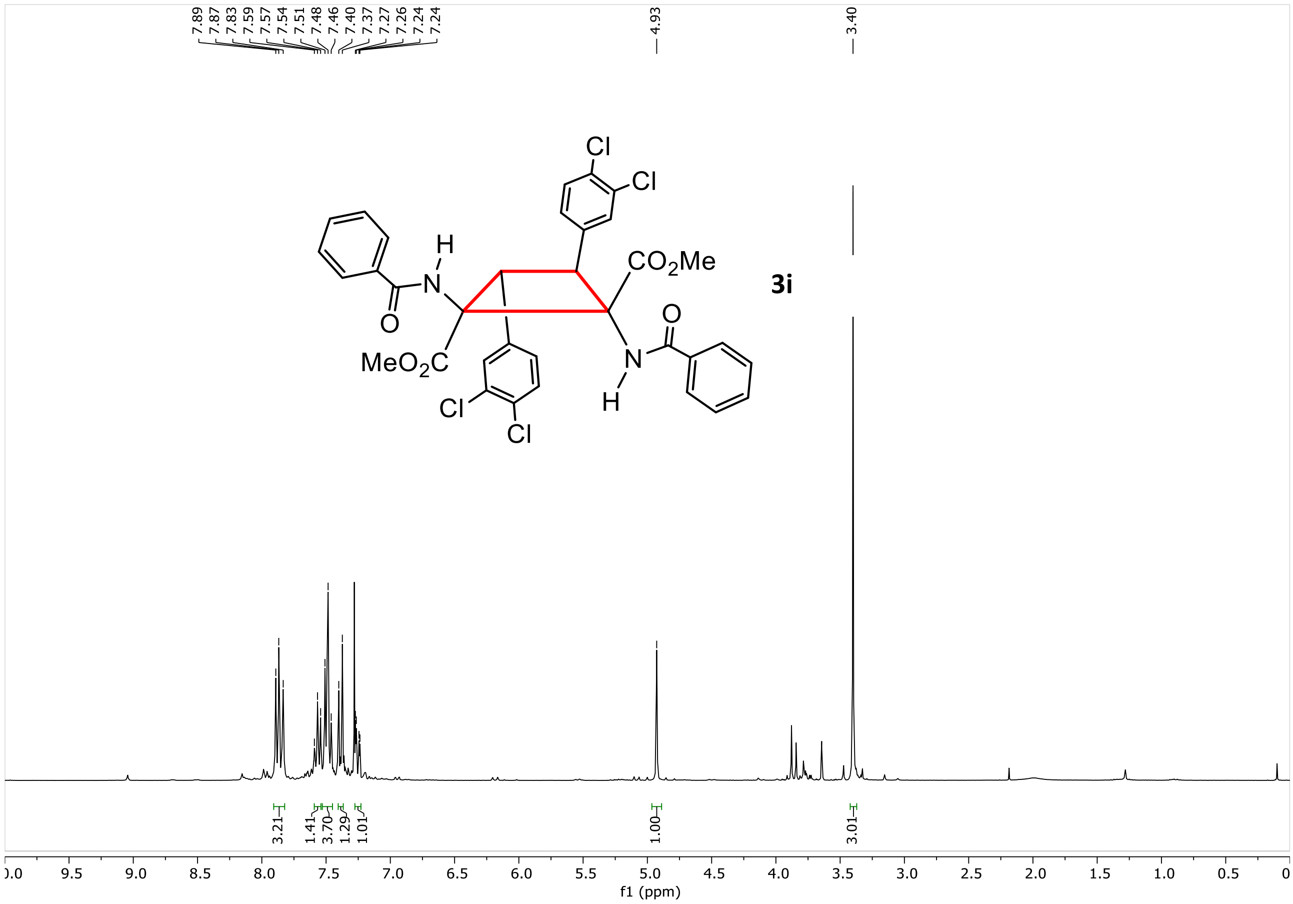

^{1}H NMR ($CDCl_3$, 300.13 MHz) of **3i**

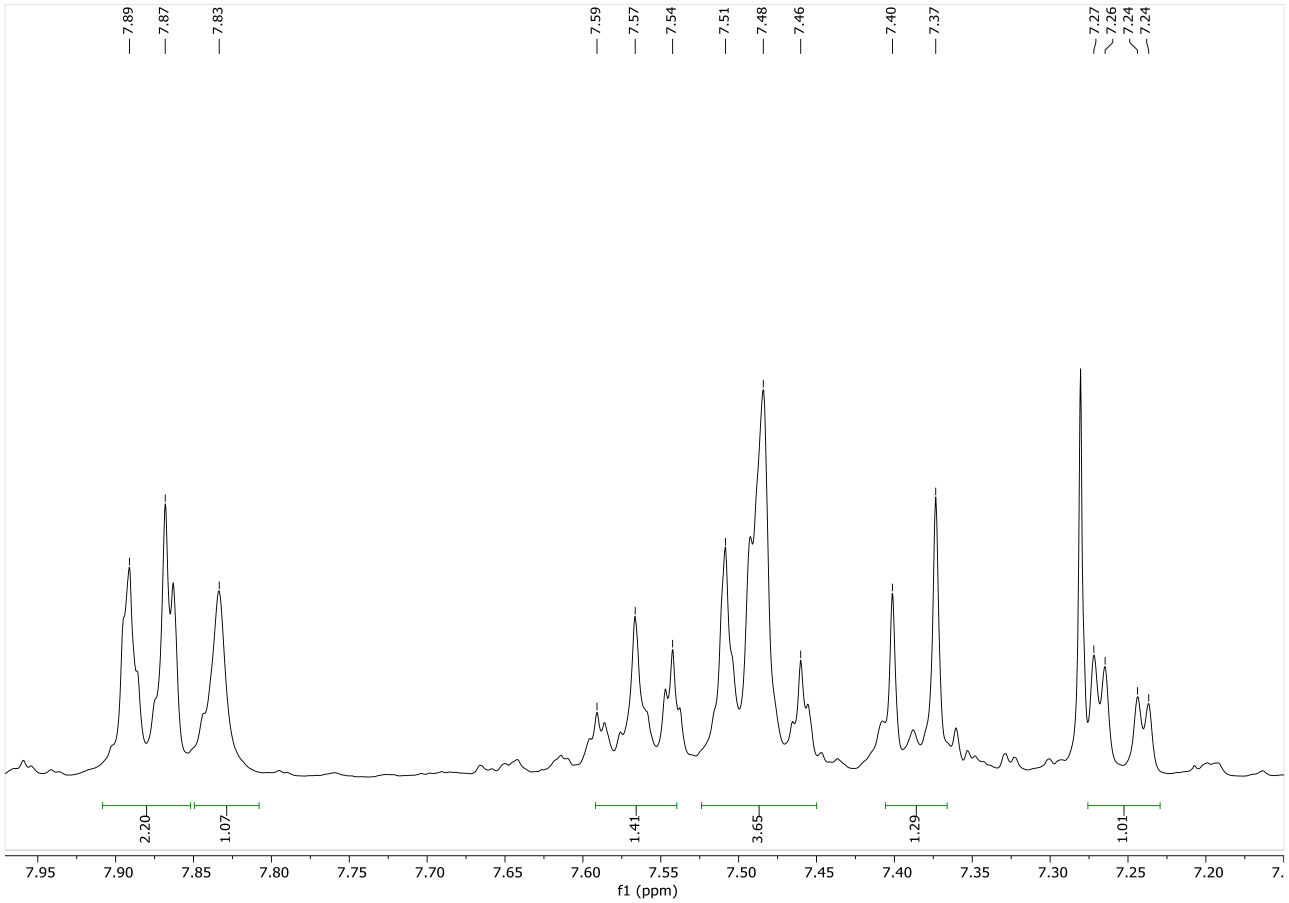

^{1}H NMR of **3i** (zoom aromatic region)

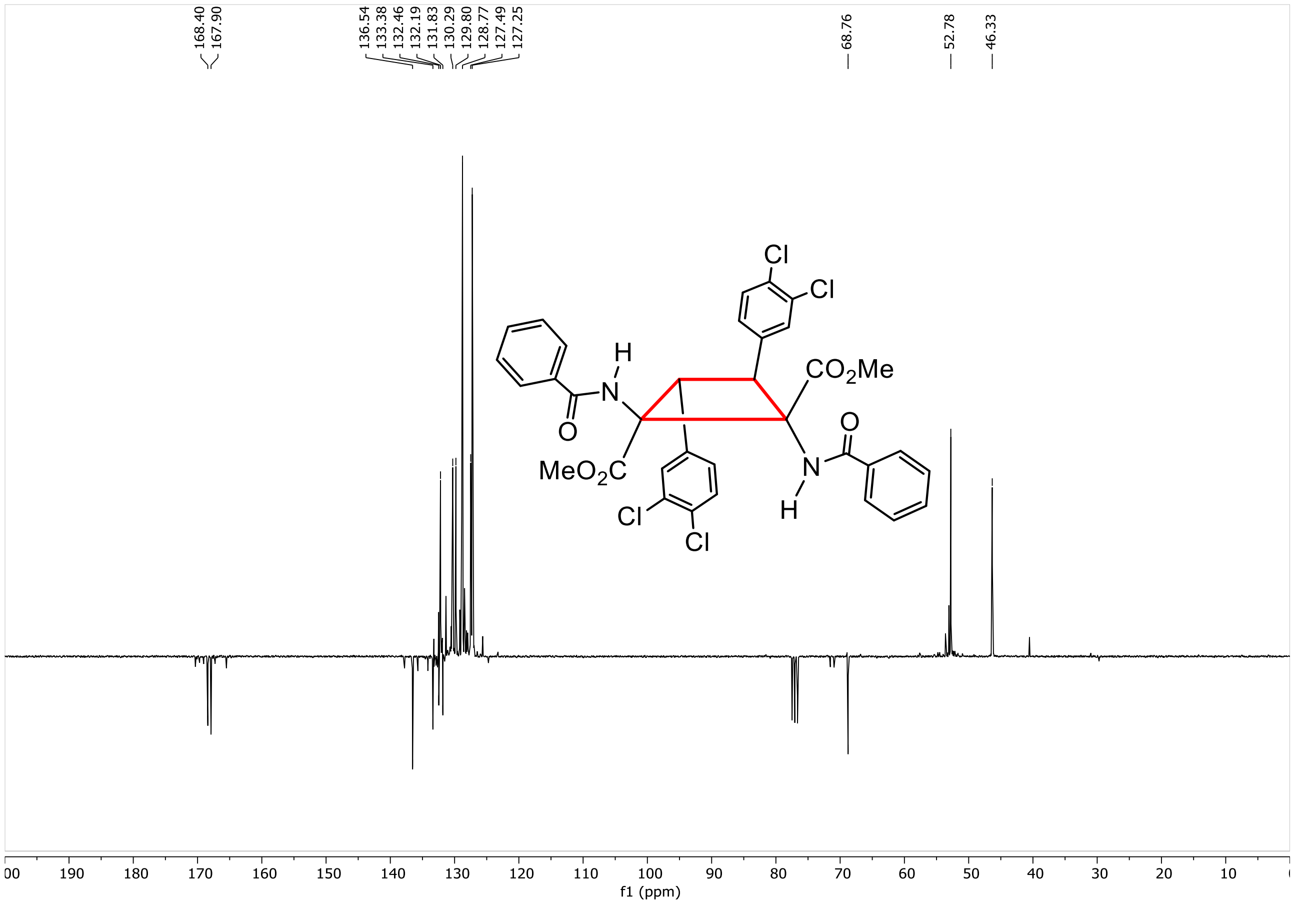

$^{13}C\{^{1}H\}$ (APT) NMR ($CDCl_3$, 75.5 MHz) of **3i**

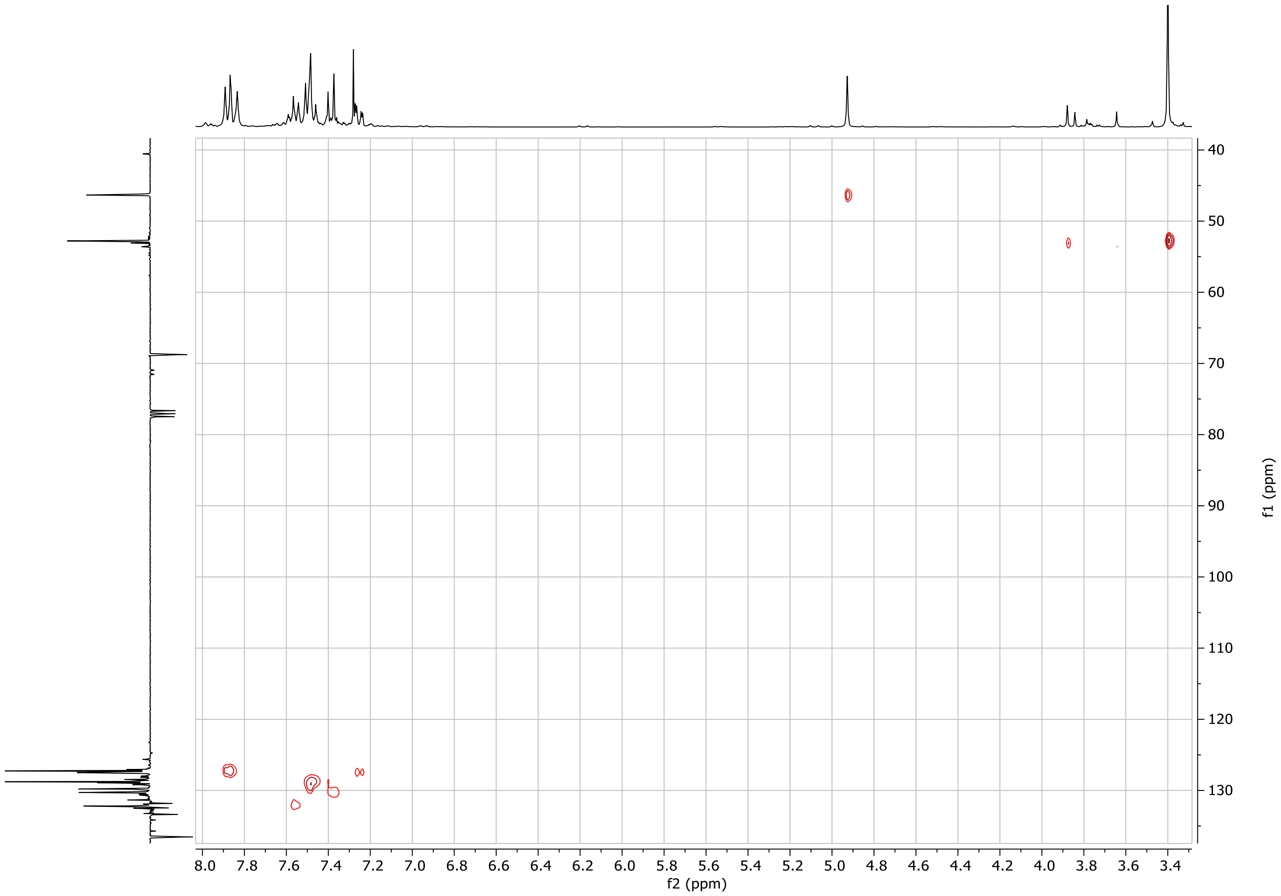

^{1}H-^{13}C HSQC correlation ($CDCl_3$) of **3i**

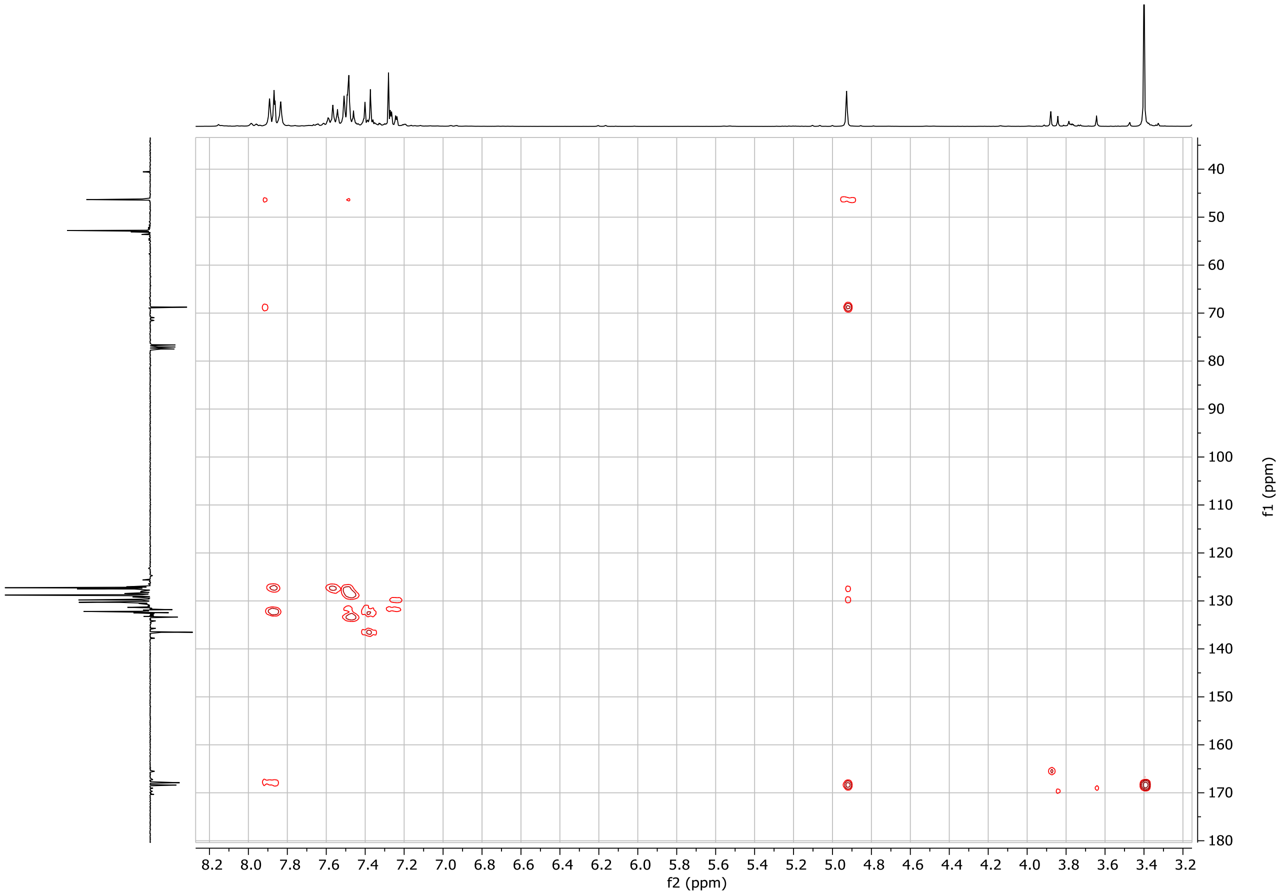

^{1}H-^{13}C HMBC correlation ($CDCl_3$) of **3i**

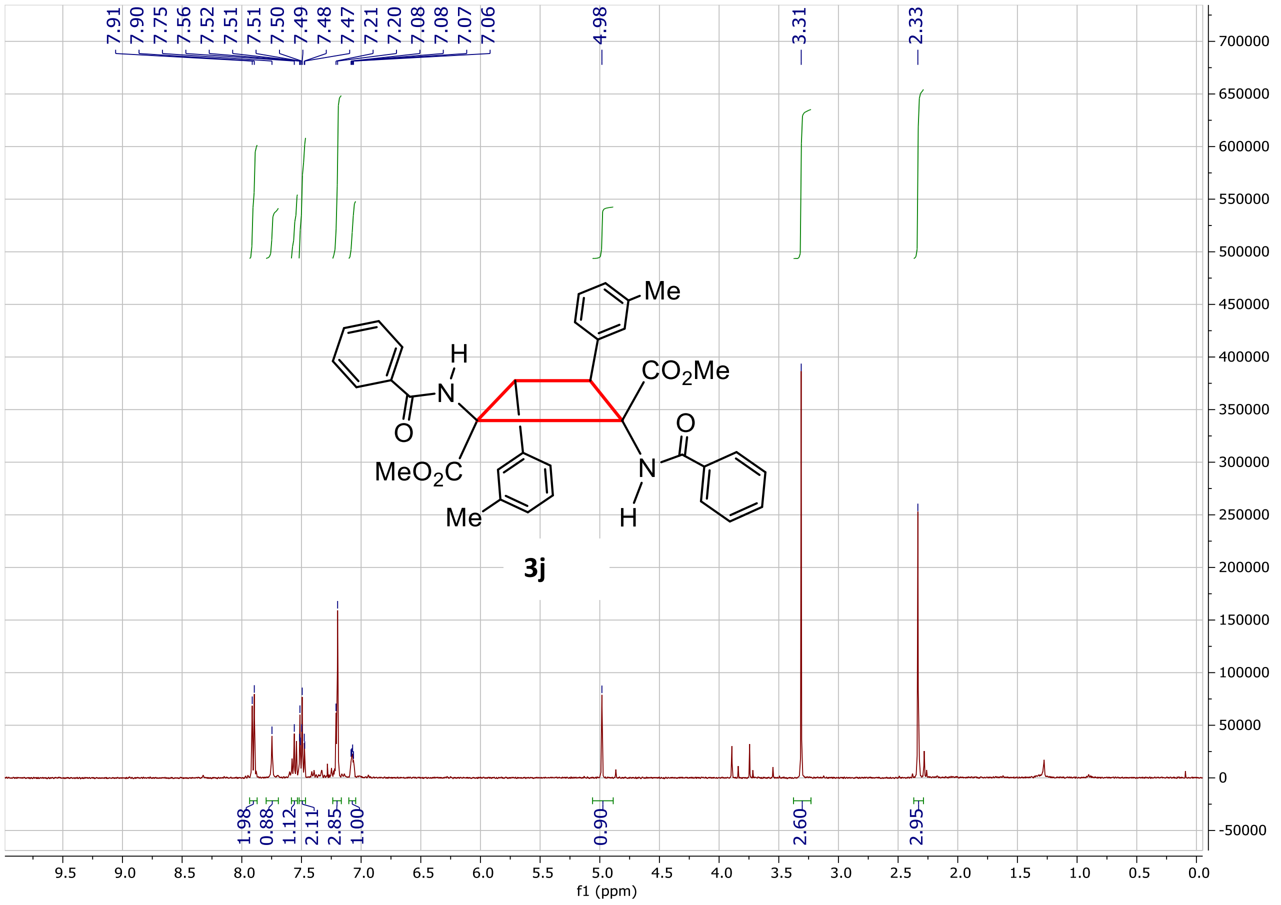

^{1}H NMR ($CDCl_3$, 300.13 MHz) of **3j**

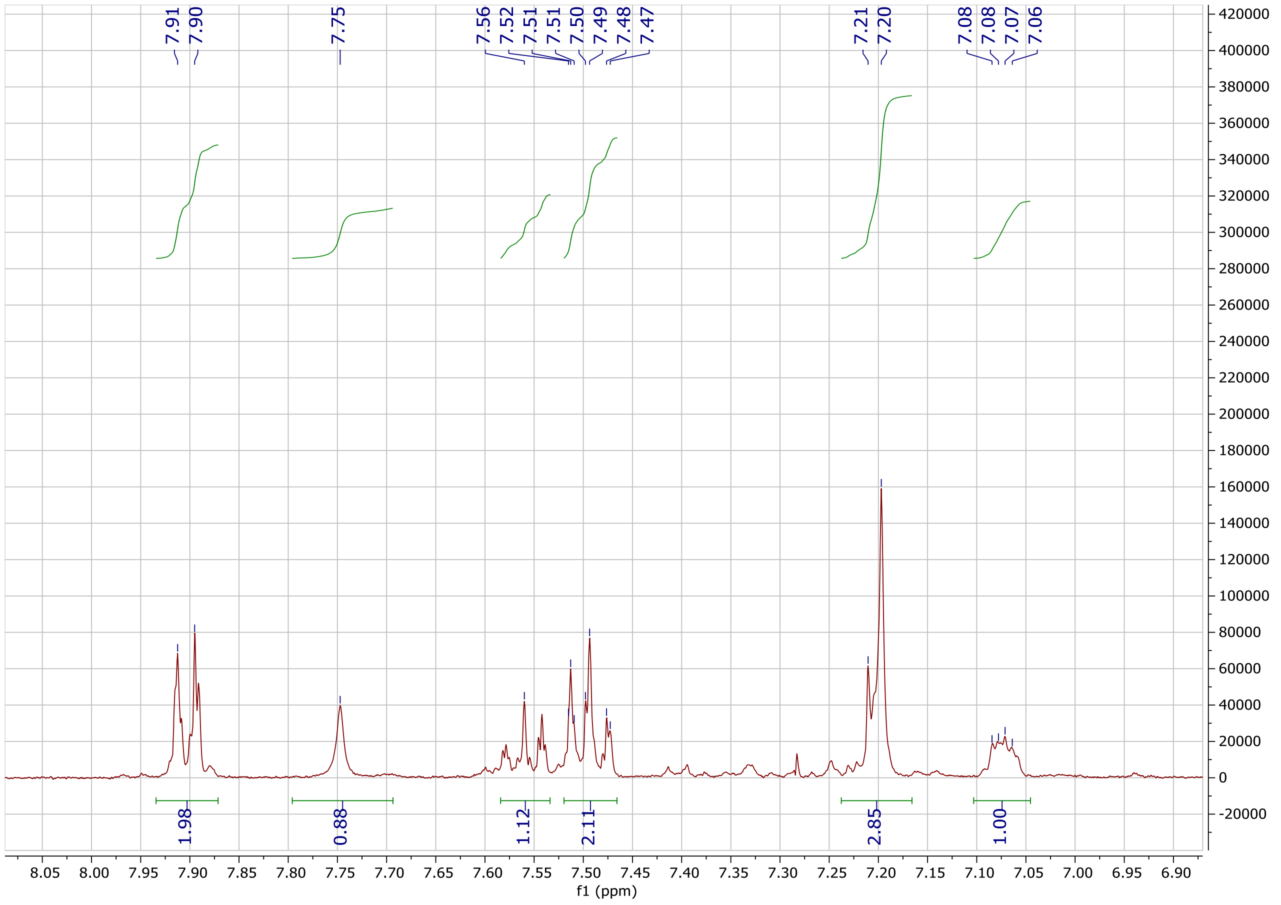

^{1}H NMR of **3j** (zoom aromatic region)

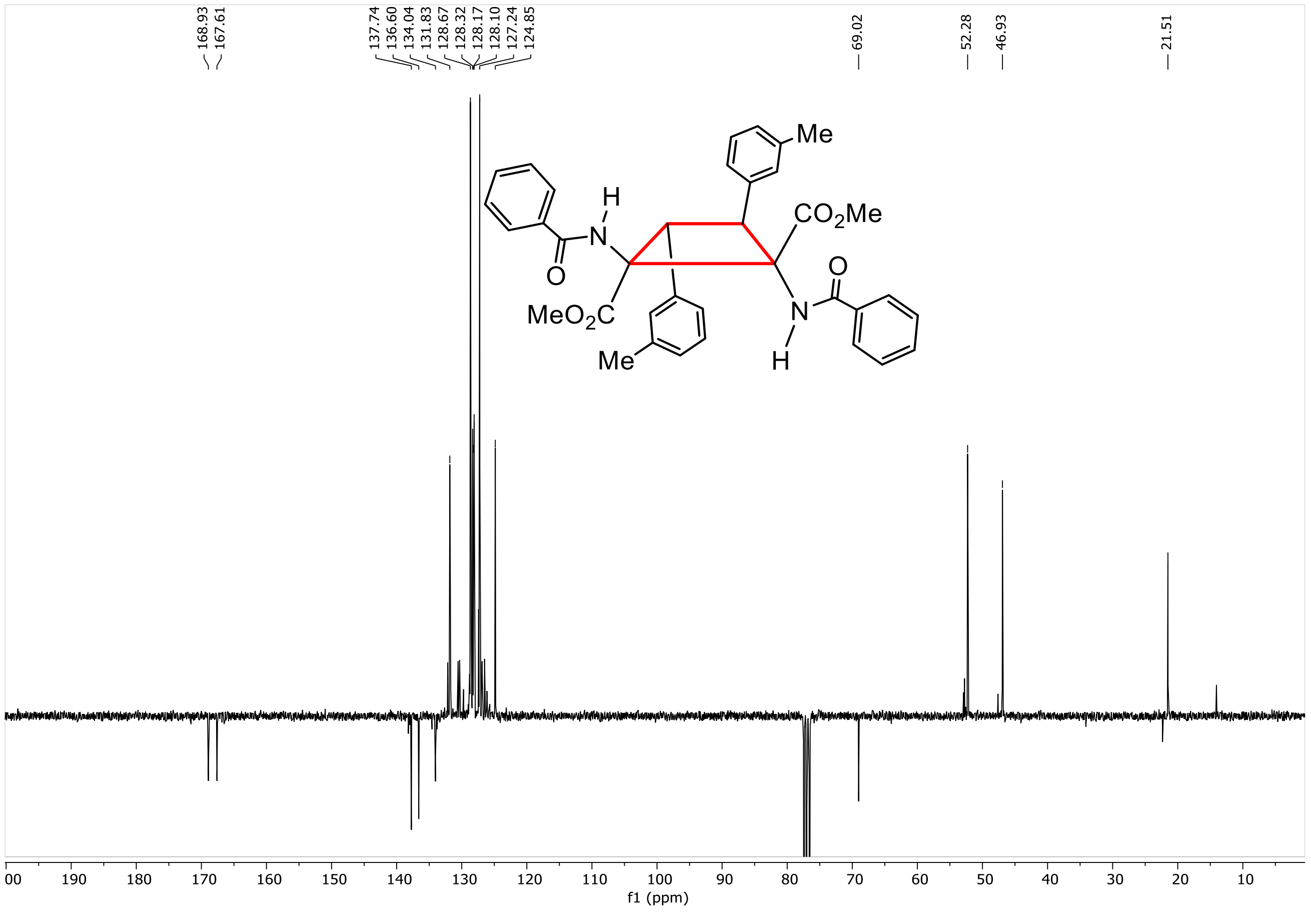

^{13}C{^{1}H} (APT) NMR ($CDCl_3$, 75.5 MHz) of **3j**

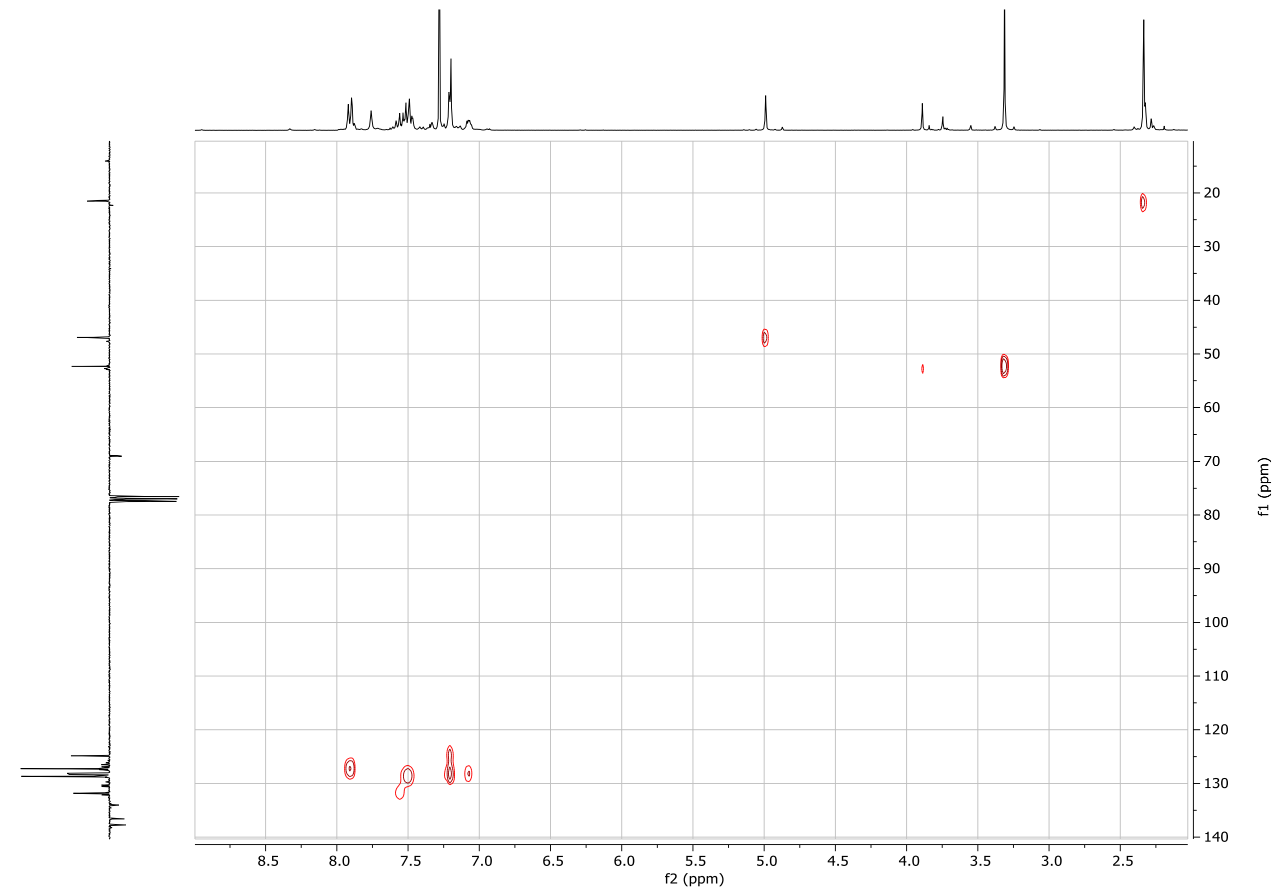

^{1}H-^{13}C HSQC correlation ($CDCl_3$) of **3j**

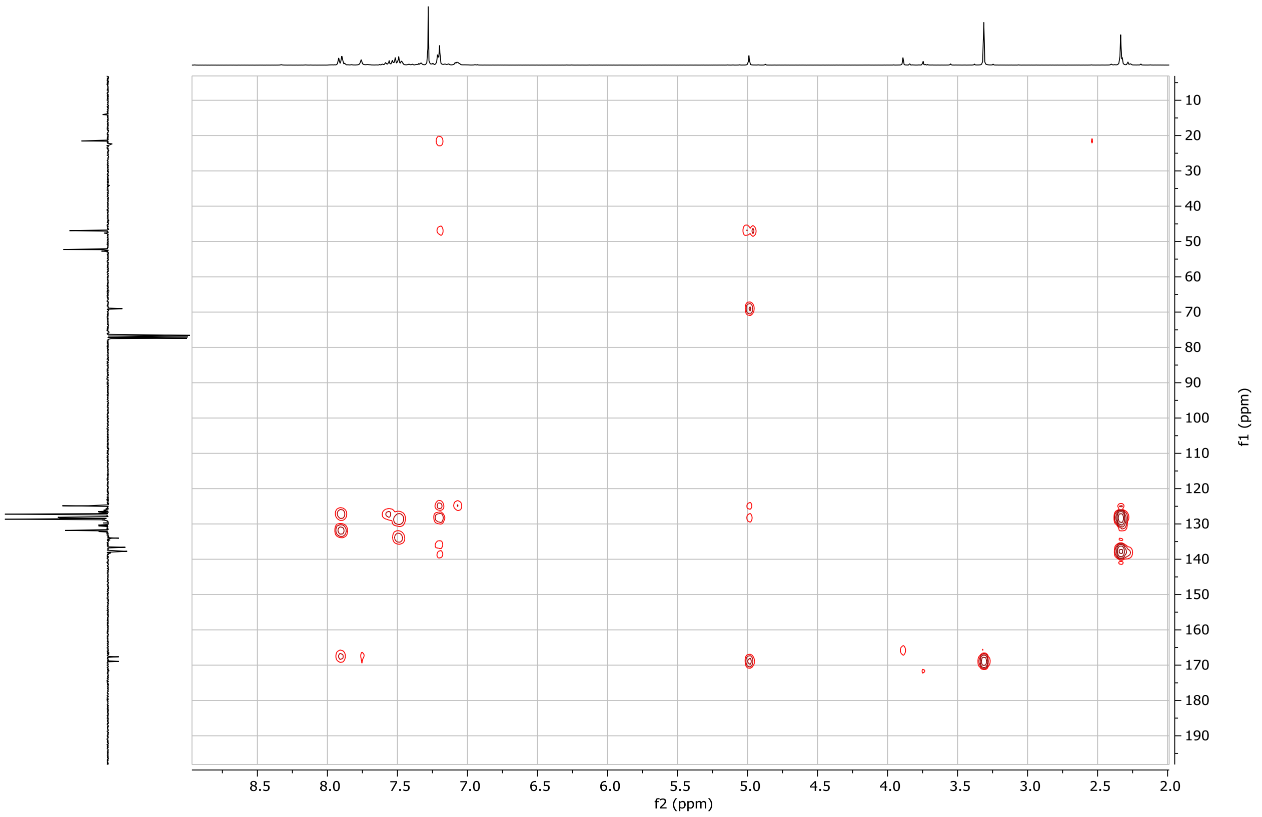

^{1}H-^{13}C HMBC correlation ($CDCl_3$) of **3j**

Miscellaneous information

All potential isomers for [2+2] cycloaddition and corresponding NMR symmetry

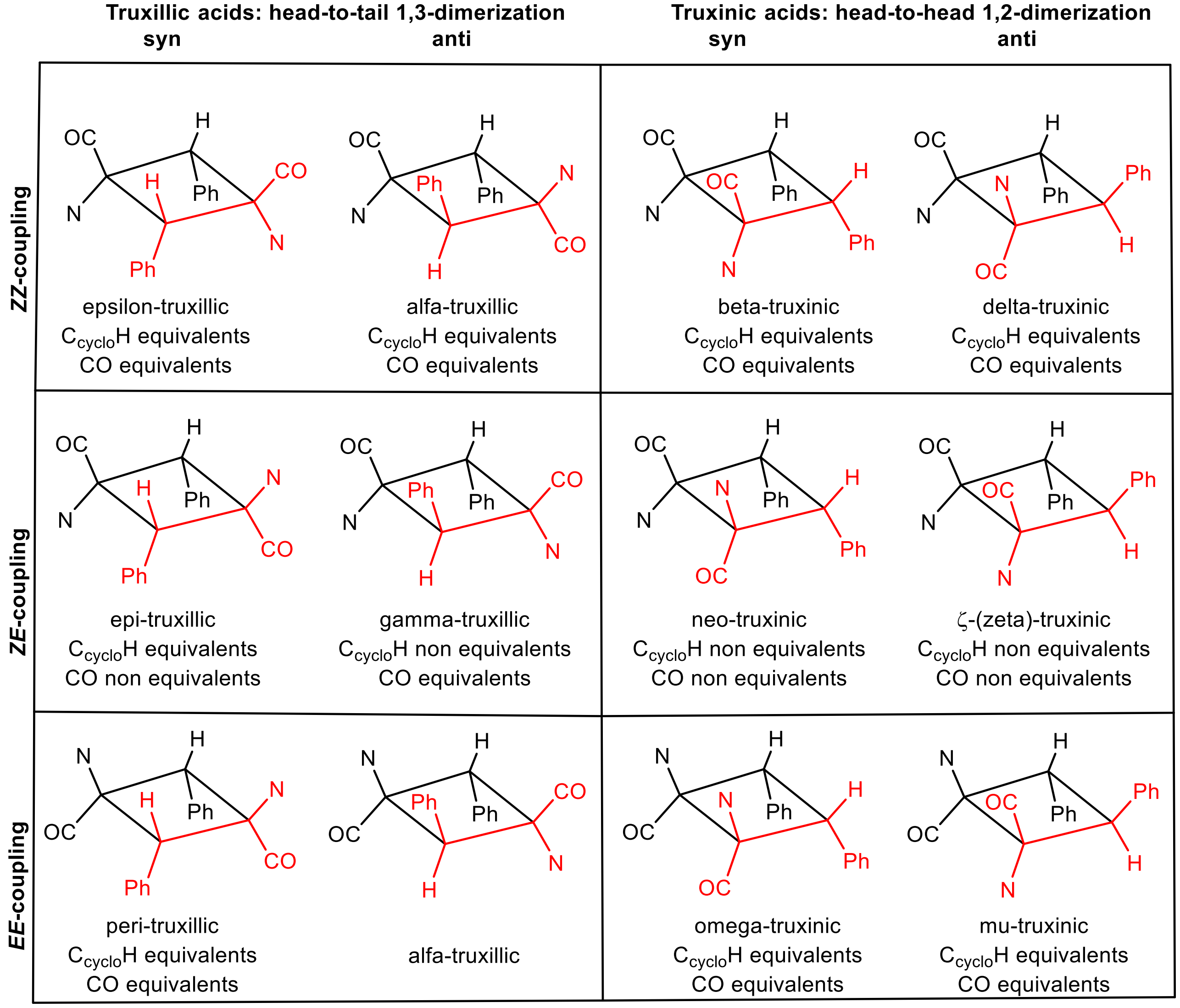

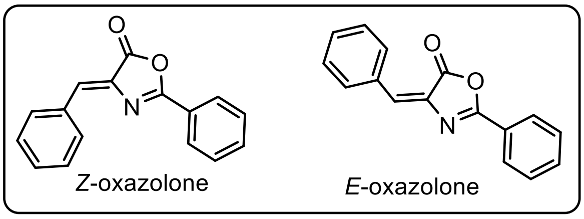

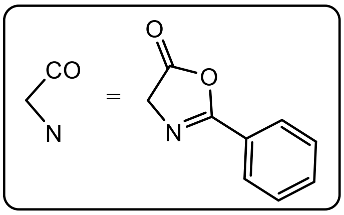

DP4 characterization of mu isomer: comparison of experimental (NMR) and DFT values. For NMR spectra, see compound 2a.

In cases where one single set of experimental data can be due to only one of several different isomers, statistical tools based on the comparison of experimental data (the experimental set of chemical shifts) and the calculated chemical shifts determined by DFT methods, are available.[1-10] One of the most popular tools is the DP4 method, developed by J. M. Goodman in 2010.[1-3] This method evaluates the distribution of the deviations between the set of experimental chemical shifts and each set of DFT-calculated chemical shifts for each isomer, in such a way that the isomer where these deviations fits better with the Student's t distribution will have a higher probability. The method applies with outstanding precision for a variety of situations, from the determination of molecular structures of natural products (under continuous revision),[11-13] to the structural elucidation of compounds with pharmacological activity.[2,14,15]

Our structural determination fits exactly in the premises of DP4 (only one experimental set of data and several possible isomers). We have selected the simplest representative compound among all obtained (**2a**) because it can be obtained in pure form and DFT calculations will be easier than in other isomers. In addition, we have selected the ^{13}C chemical shifts for comparison, because of the larger chemical shift dispersion of ^{13}C NMR data compared with ^{1}H NMR. Therefore, we have calculated the structures (DFT-B3LYP) of the five isomers whose symmetry match with the experimental NMR data of **2a**. A very simple applet freely available allows the application of the DP4 method.[16] The results of the application of DP4 (see below) gives a probability of 86.2% to the mu isomer, and the next isomer with some probability (omega) has only a 13.2%. Therefore, NMR and DFT methods suggest that the isomer obtained by photocycloaddition of (*Z*)-oxazolones is the mu-isomer of the 1,2-diaminotruxinic acid, as a result of the *anti*-head-to-head dimerization of two (*E*)-oxazolones. However, the best value of probability for the mu-isomer (86.2%) is far away from the values requested to consider a given structure unambiguously determined, which are typically higher than 98%.[1] Therefore, DP4 suggests the structure, but it cannot be considered as the definitive proof.

(1) Smith, S. G.; Goodman, J. M. Assigning Stereochemistry to Single Diastereoisomers by GIAO NMR Calculation: The DP4 Probability. *J. Am. Chem. Soc.* **2010**, *132*, 12946.

(2) Ermanis, K.; Parkes, K. E. B.; Agback, T.; Goodman, J. M. Expanding DP4: Application to drug compounds and automation. *Org. Biomol. Chem.* **2016**, *14*, 3943.

(3) Ermanis, K.; Parkes, K. E. B.; Agback, T.; Goodman, J. M. Doubling the Power of DP4 for Computational Structure Elucidation. *Org. Biomol. Chem*. **2017**, *15*, 8998.

(4) Grimblat, N.; Zanardi, M. M.; Sarotti, A. M. Beyond DP4: an Improved Probability for the Stereochemical Assignment of Isomeric Compounds using Quantum Chemical Calculations of NMR Shifts. *J. Org. Chem.* **2015**, *80*, 12526.

(5) Navarro-Vázquez, A. State of the art and perspectives in the application of quantum chemical prediction of ^{1}H and ^{13}C chemical shifts and scalar couplings for structural elucidation of organic compounds *Magn. Res. Chem.* **2017**, *55*, 29.

(6) Aliev, A. E.; Mia, Z. A.; Khaneja, H. S.; King, F. D.. Structures in Solutions from Joint Experimental-Computational Analysis: Applications to Cyclic Molecules and Studies of Noncovalent Interactions. *J. Phys. Chem. A* **2012**, *116*, 1093.

(7) Lodewyk, M. W.; Siebert, M. R.; Tantillo, D. J. Computational Prediction of ^{1}H and ^{13}C Chemical Shifts: A Useful Tool for Natural Product, Mechanistic, and Synthetic Organic Chemistry. *Chem. Rev.* **2012**, *112*, 1839.

(8) Xin, D.; Sader, C. A.; Chaudhary, O.; Jones, P.-J.; Wagner, K.; Tautermann, C. S.; Yang, Z.; Busacca, C. A.; Saraceno, R. A.; Fandrick, K. R.; Gonnella, N. C.; Horspool, K.; Hansen, G.; Senanayake, C. H.. Development of a ^{13}C NMR Chemical Shift Prediction Procedure Using B3LYP/cc-pVDZ and Empirically Derived Systematic Error Correction Terms: A Computational Small Molecule Structure Elucidation Method. *J. Org. Chem.* **2017**, *82*, 5135.

(9) Xin, D.; Jones, P.-J.; Gonnella, N. C.. DiCE: Diastereomeric in Silico Chiral Elucidation, Expanded DP4 Probability Theory Method for Diastereomer and Structural Assignment. *J. Org. Chem.* **2018**, *83*, 5035.

(10) Zanardi, M. M.; Biglione, F. A.; Sortino, M. A.; Sarotti, A. M. General Quantum-Based NMR Method for the Assignment of Absolute Configuration by Single or Double Derivatization: Scope and Limitations. *J. Org. Chem.* **2018**, *83*, 11839.
(11) Cooper, J. K.; Li, K.; Aubé, J.; Coppage, D. A.; Konopelski, J. P. Application of the DP4 Probability Method to Flexible Cyclic Peptides with Multiple Independent Stereocenters: The True Structure of Cyclocinamide A. *Org. Lett.* **2018**, *20*, 4314.
(12) Calabro, K.; Chalén, B. E.; Genta-Jouve, G.; Jaramillo, K. B.; Domínguez, C.; de la Cruz, M.; Cautain, B.; Reyes, F.; Thomas, O. P.; Rodríguez, J. Callyspongidic Acids: Amphiphilic Diacids from the Tropical Eastern Pacific Sponge Callyspongia cf. californica. *J. Nat. Prod.* **2018**, *81*, 2301.
(13) Jamison, M. T.; Dalisay, D. S.; Molinski, T. F. Peroxide Natural Products from Plakortis zyggompha and the Sponge Association Plakortis halichondrioides–Xestospongia deweerdtae: Antifungal Activity against Cryptococcus gattii. *J. Nat. Prod.* **2016**, *79*, 555.
(14) MacGregor, C. I.; Han, B. Y.; Goodman, J. M.; Paterson, I. Toward the stereochemical assignment and synthesis of hemicalide: DP4f GIAO-NMR analysis and synthesis of a reassigned C16-C28 subunit. *Chem. Commun.* **2016**, *52*, 4632.
(15) Haensele, E.; Saleh, N.; Read, C. M.; Banting, L.; Whitley, D. C.; Clark, T. Can Simulations and Modeling Decipher NMR Data for Conformational Equilibria? Arginine–Vasopressin. *J. Chem. Inf. Model.* **2016**, *56*, 1798.
(16) http://www-jmg.ch.cam.ac.uk/tools/nmr/DP4/

Screen reflecting the input of the applet.

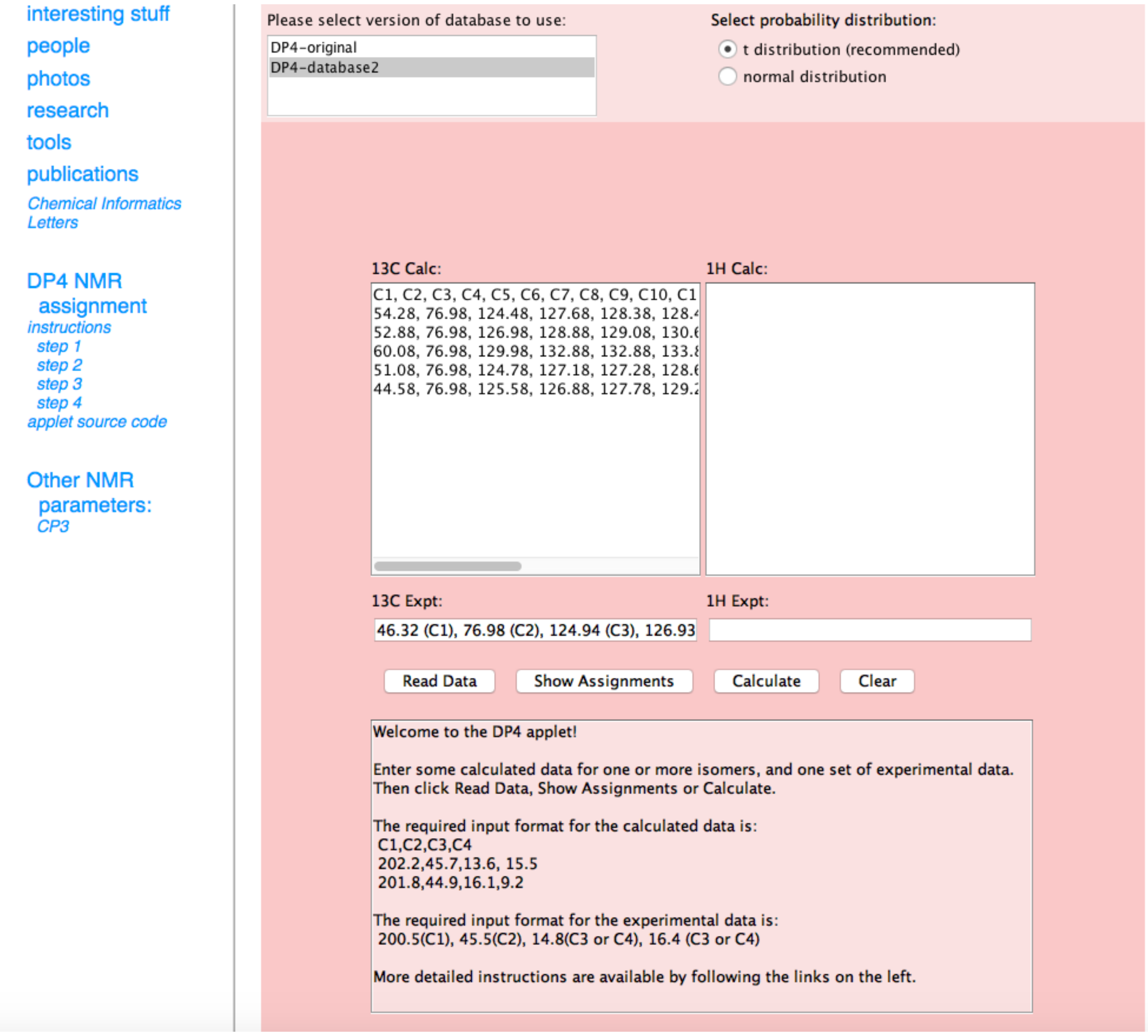

Output of the applet after DP4. Isomer 1: peri-truxillic; Isomer 2: beta-truxinic; Isomer 3: delta-truxinic; Isomer 4: omega-truxinic; Isomer 5: mu-truxinic. For geometries, see preceding figure. The output of the program attributes a probability confidence of 86.2% to the mu isomer.

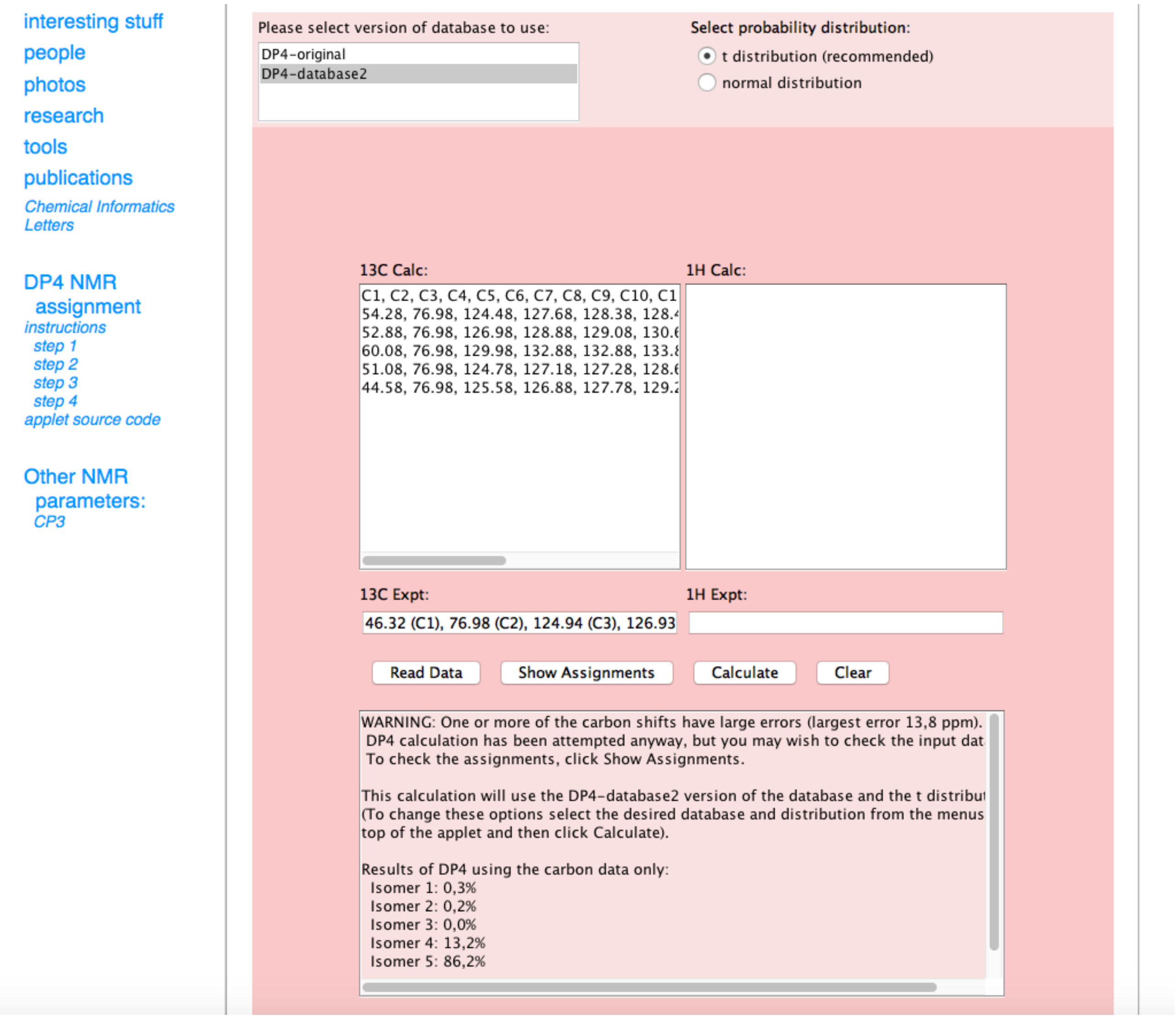

Conversions (%) of oxazolones 1 to give cyclobutene-bis(oxazolones) 2 from two consecutive reactions in continuous flow conditions

After the first reaction of 30 min, the photobleached catalyst was eliminated and fresh catalyst was added to carry out the second cycle (30 min.) In all cases, the second cycle increases considerable the reaction conversion.

oxazolone	[**1**] (mM)[a]	Flow (O_2, 30 min)	Flow (O_2, 30 min)
**1a**	120	60	75
**1b**	120	45	75
**1c**	150	43	65
**1d**	120	53	85
**1e**	120	50	80
**1f**	25	< 5	70
**1g**	120	44	78
**1i**	120	58	59
**1j**	150	56	79

[a]Concentration is 120 mM by default, except for **1c** and **1k** (150 mM) and poorly soluble **1f** (25 mM);

Absorption spectra in CH_2Cl_2 solution of *Z*-oxazolones 1 and some examples of *E*-oxazolones

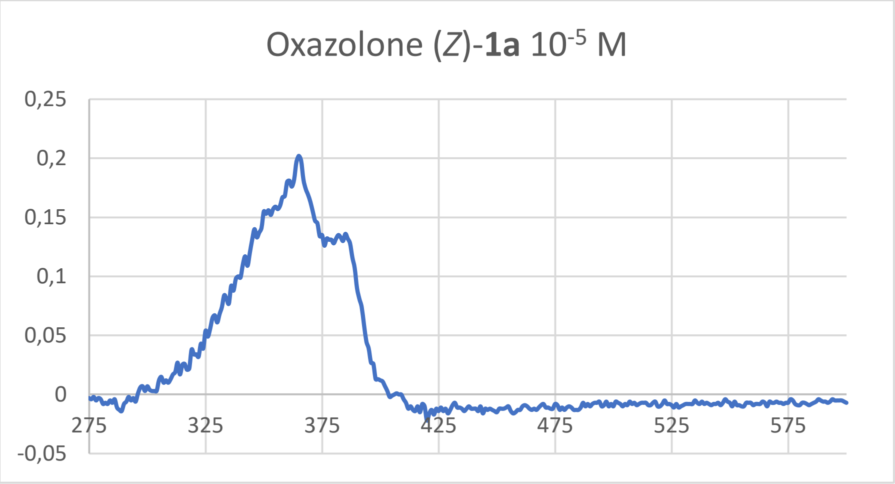

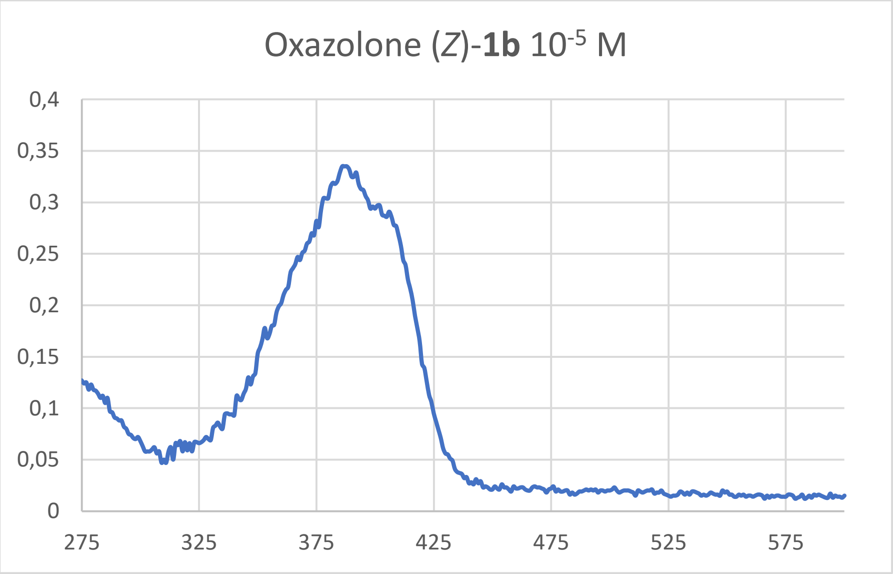

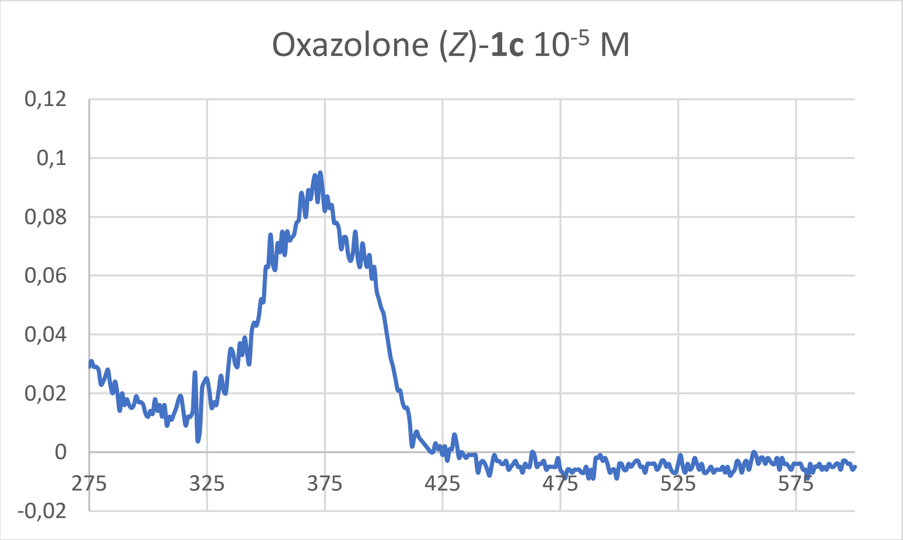

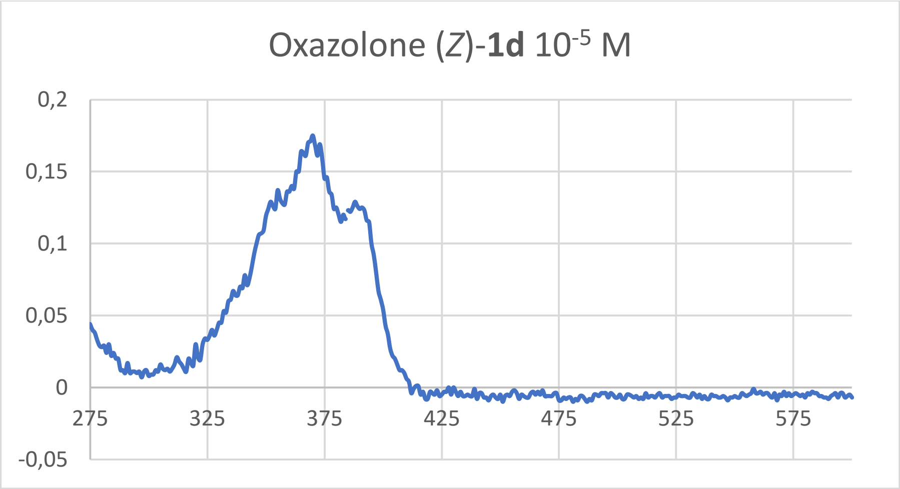
Oxazolone (Z)-1d 10-5 M
0,2
0,15
0,1
0,05
0
-0,05
275
325
375
425
475
525
575

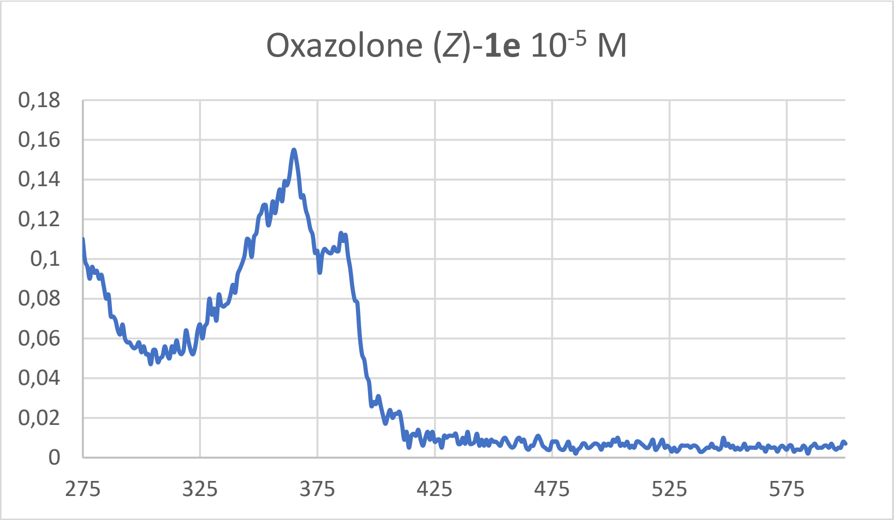
Oxazolone (Z)-1e 10-5 M
0,18
0,16
0,14
0,12
0,1
0,08
0,06
0,04
0,02
0
275
325
375
425
475
525
575

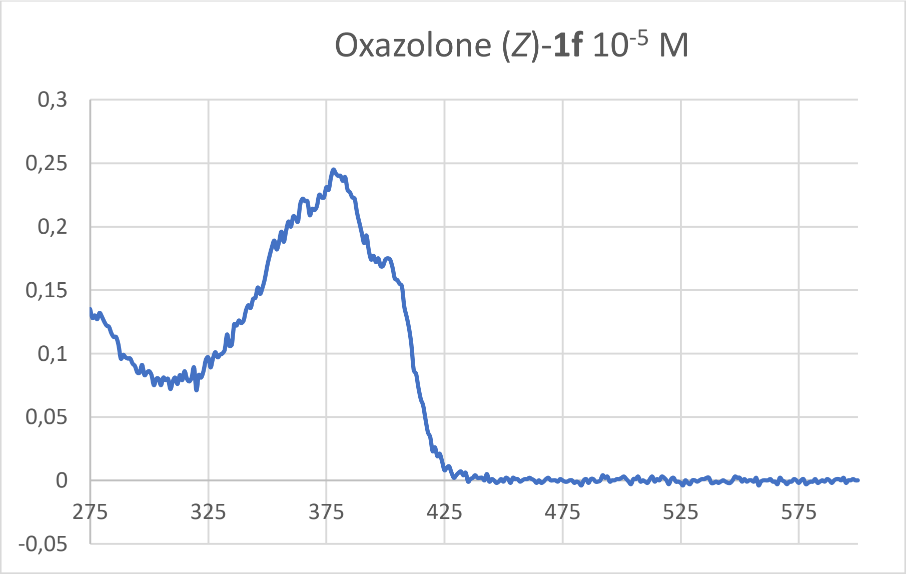
Oxazolone (Z)-1f 10-5 M
0,3
0,25
0,2
0,15
0,1
0,05
0
-0,05
275
325
375
425
475
525
575

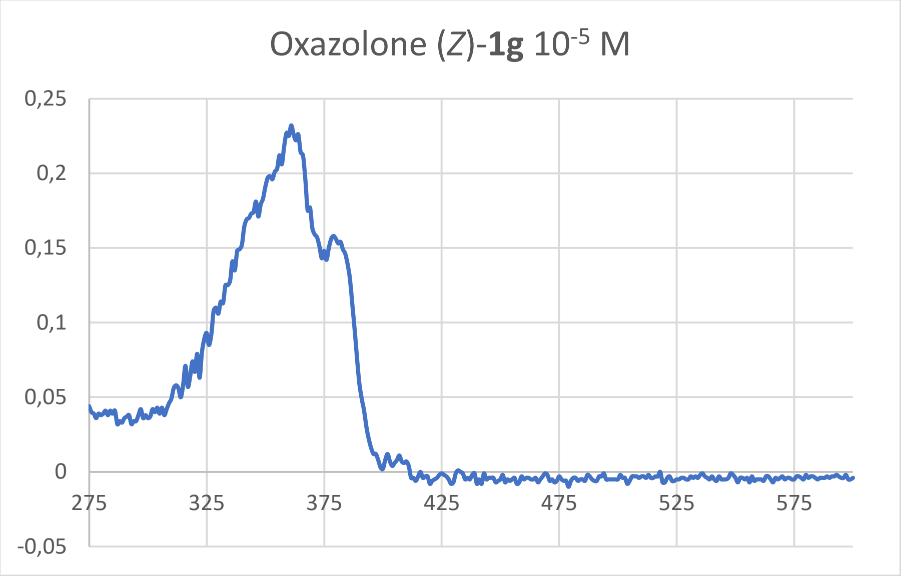
Oxazolone (Z)-1g 10^{-5} M
0,25
0,2
0,15
0,1
0,05
0
-0,05
275
325
375
425
475
525
575

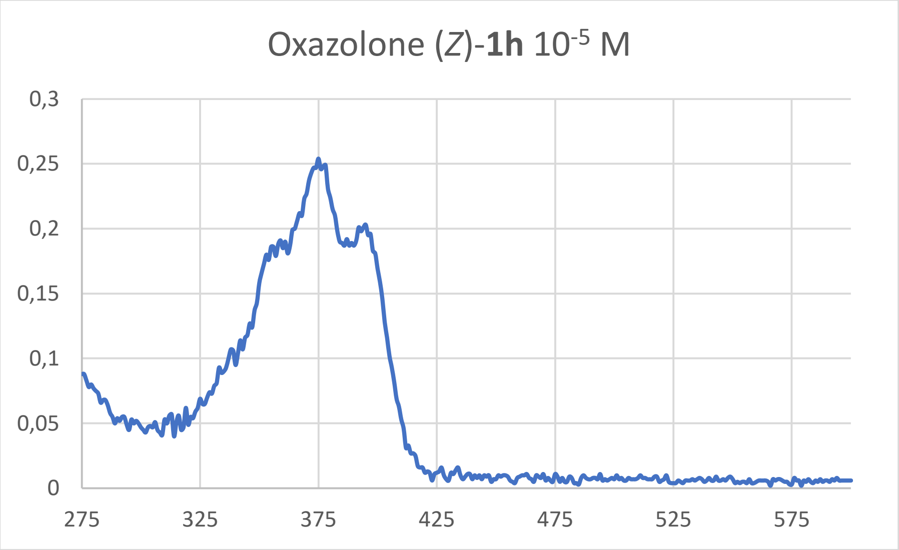
Oxazolone (Z)-1h 10^{-5} M
0,3
0,25
0,2
0,15
0,1
0,05
0
275
325
375
425
475
525
575

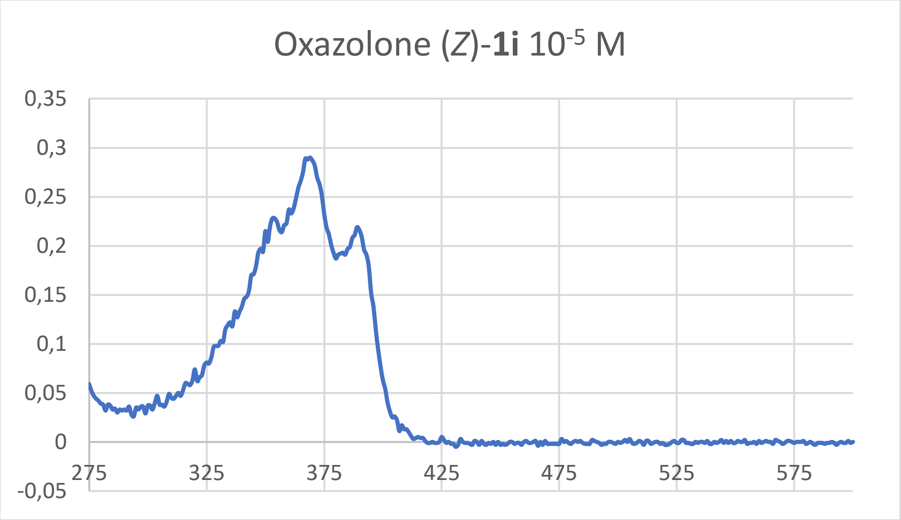
Oxazolone (Z)-1i 10^{-5} M
0,35
0,3
0,25
0,2
0,15
0,1
0,05
0
-0,05
275
325
375
425
475
525
575

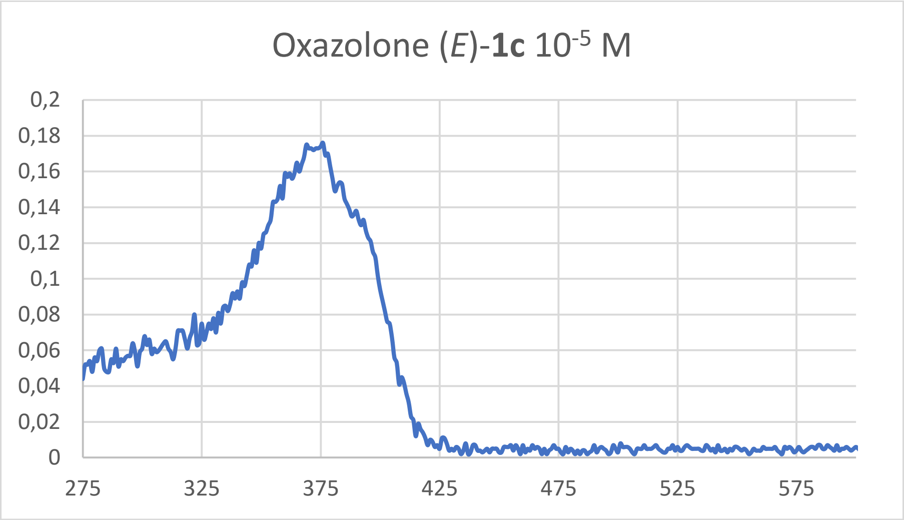
Oxazolone (E)-1c 10-5 M
0,2
0,18
0,16
0,14
0,12
0,1
0,08
0,06
0,04
0,02
0
275
325
375
425
475
525
575

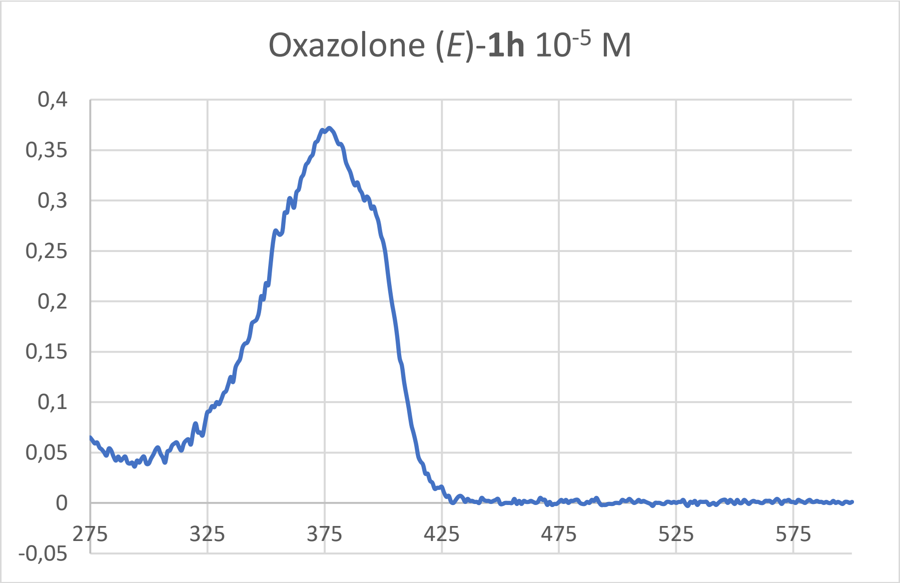
Oxazolone (E)-1h 10-5 M
0,4
0,35
0,3
0,25
0,2
0,15
0,1
0,05
0
-0,05
275
325
375
425
475
525
575

Cyclic Voltammetry of oxazolone (Z)-1c

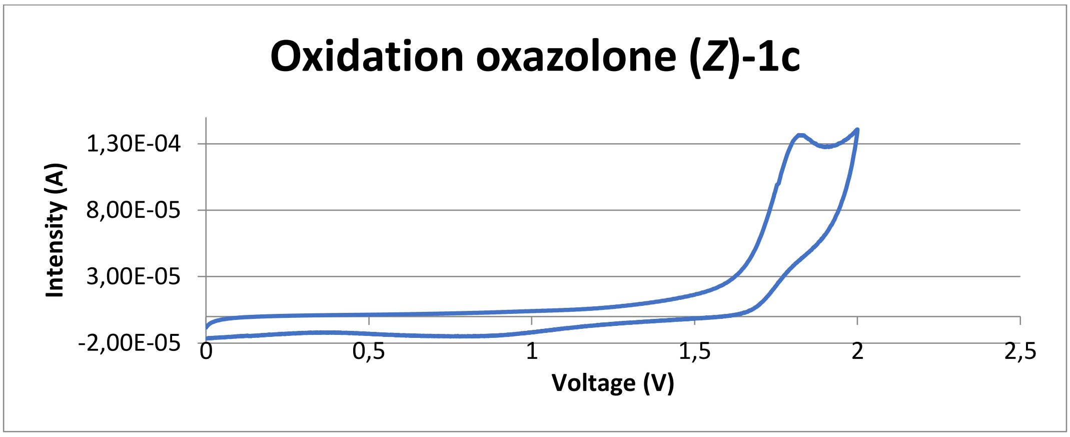

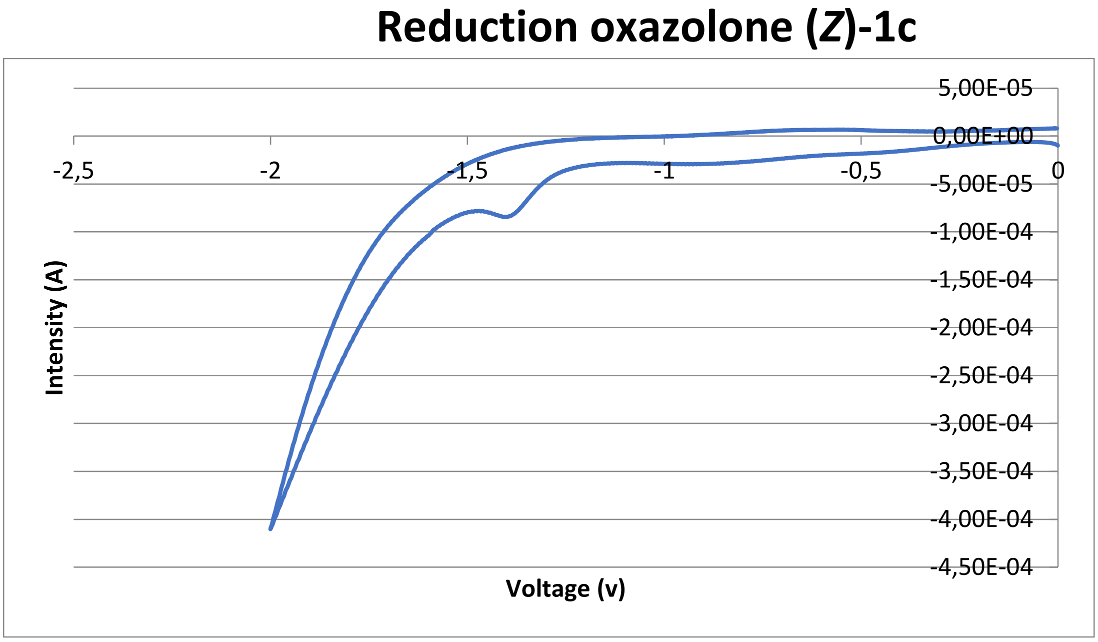

Transient absorption spectra of a deaerated CH_2Cl_2 solution of $Ru(bpy)_3^{2+}$ recorded at different times after the laser pulse: 10 ns (black); 42 ns (red); 124 ns (blue); 224 ns (pink); 550 ns (green); 924 ns (dark blue) and 1376 ns (violet) (λ_{exc} = 532 nm).

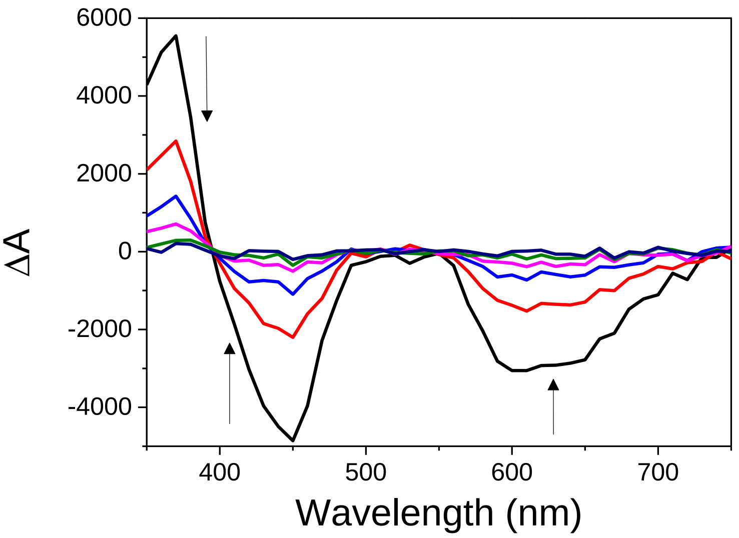

NMR spectra of mixtures of oxazolones (*Z*)-1c or (*E*)-1c with the Ru-photocatalyst after 10 min irradiation with LED light (465 nm)

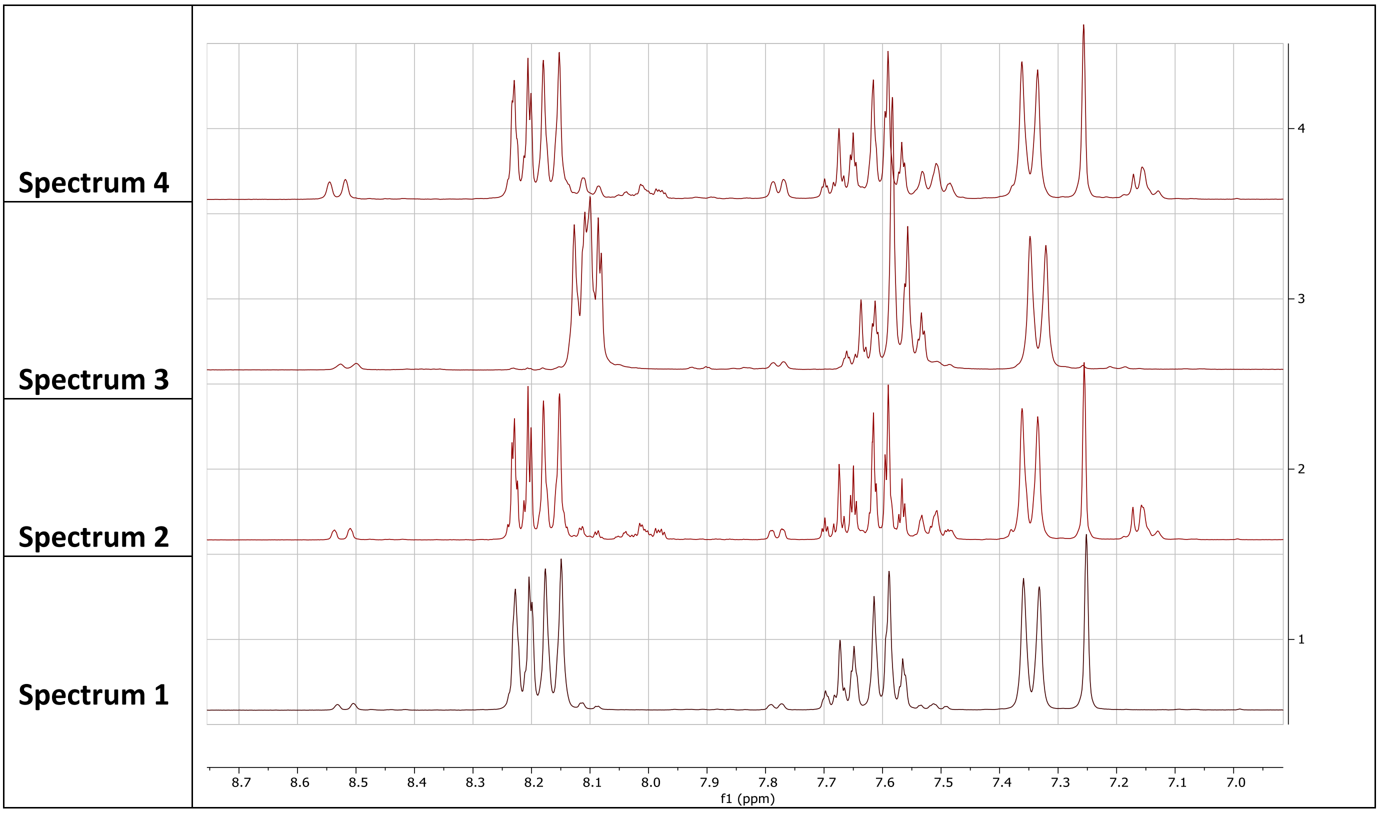

Spectrum 1 (lowest): ^{1}H NMR of oxazolone (*Z*)-**1c** in presence of $[Ru(bpy)_3](BF_4)_2$, initial mixture, no irradiation. **Spectrum 2 (mid-low)**: ^{1}H NMR of the mixture (1) after 10 minures irradiation with blue light (465 nm). **Spectrum 3 (mid-high):** ^{1}H NMR of oxazolone (*E*)-**1c** in presence of $[Ru(bpy)_3](BF_4)_2$, initial mixture, no irradiation. **Spectrum 4 (highest):** ^{1}H NMR of the mixture (3) after 10 minutes irradiation with blue light (465 nm).

Figure S3. Optimized geometries (B3LYP-D3/cc-pVTZ) of selected intermediates as shown in Figure 11. Geometries obtained with other functionals are very similar

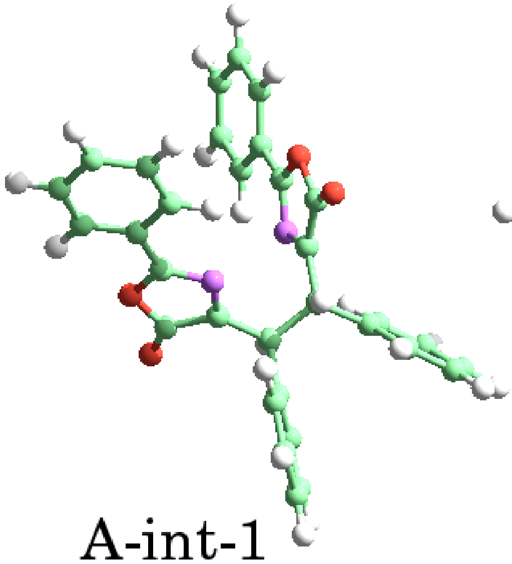

A-int-1

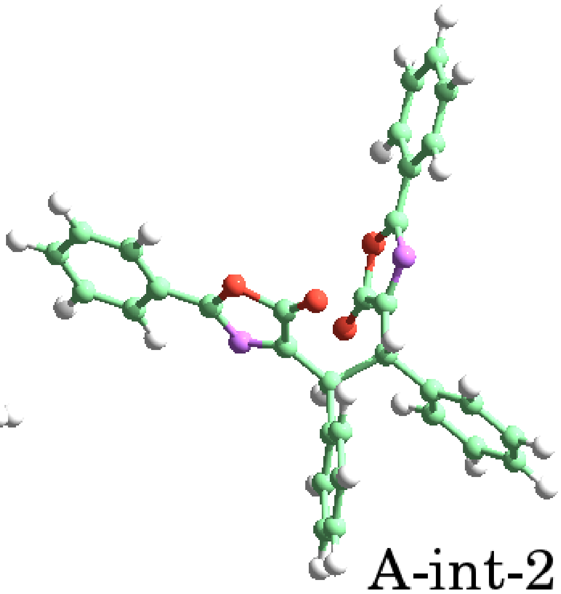

A-int-2

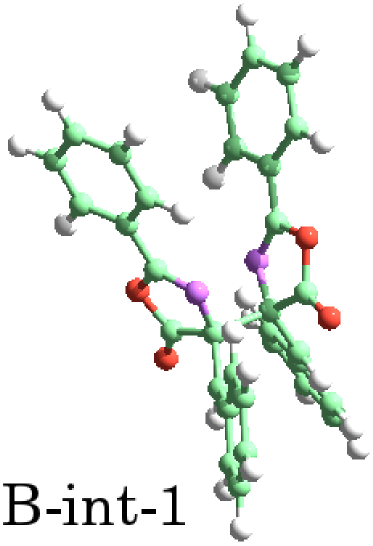

B-int-1

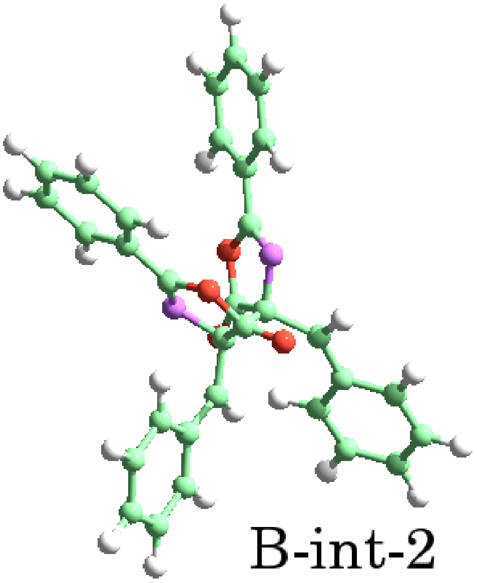

B-int-2

Figure S4. Optimized geometries of B-int-1 using cc-pVTZ basis set and different functionals. For the other intermediates, geometries obtained with different functionals are also very similar.

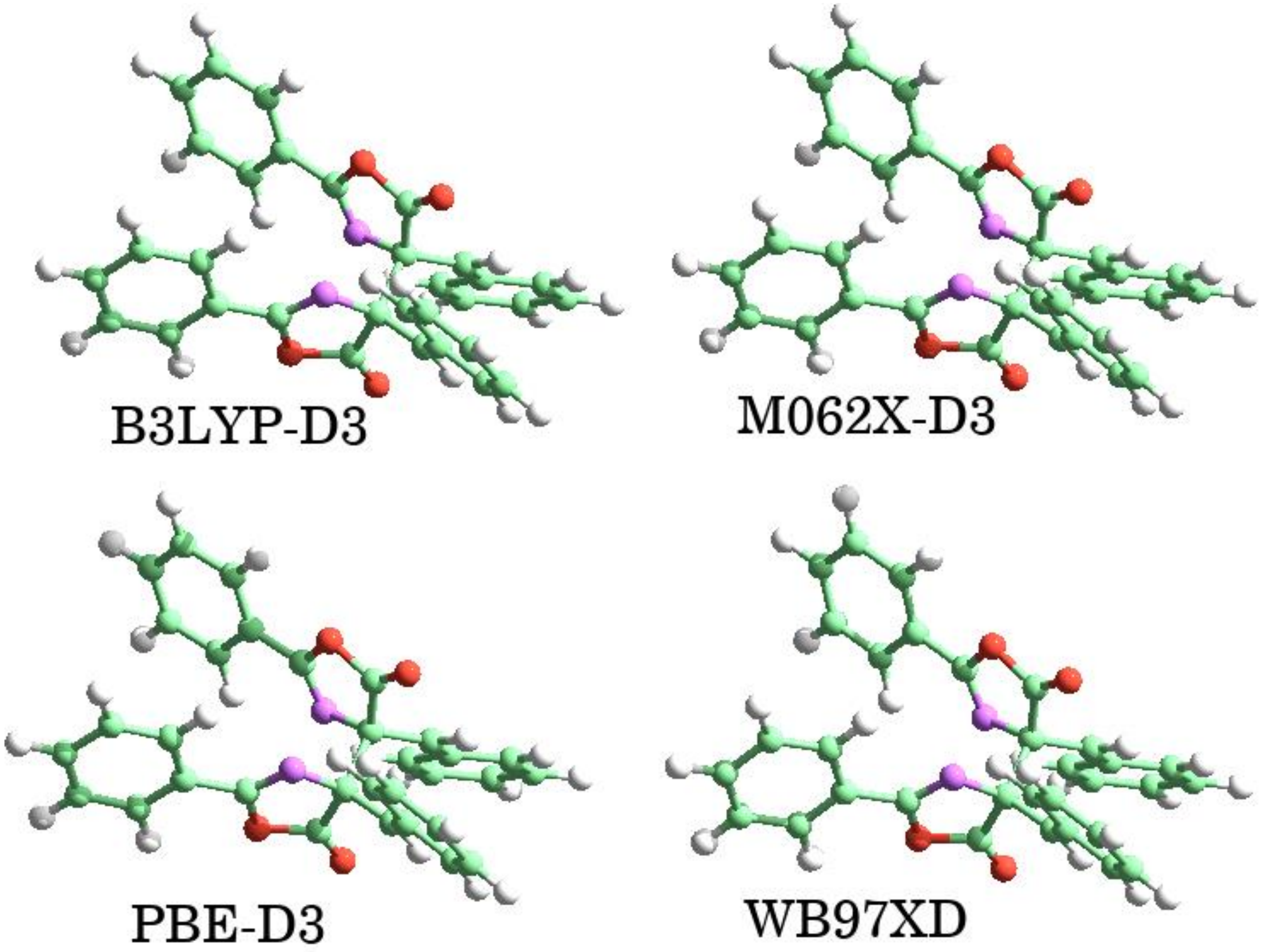

Table S2. Absolute (Ha) and relative (kJ/mol) DFT energies of minimum energy conformations of intermediates using four different functionals including dispersion: B3LYP-D3, M062X-D3, PBE-D3 and wB97XD, as well as cc-pVTZ as basis set.

Method/Molecule	Abs. energy (Ha)	Rel. energy (kJ/mol)
b3lyp-d3_ccpvtz/A-int-1	-1643.673487	0.0
b3lyp-d3_ccpvtz/A-int-2	-1643.670443	8.0
b3lyp-d3_ccpvtz/B-int-1	-1643.620540	139.0
b3lyp-d3_ccpvtz/B-int-2	-1643.614788	154.1
m062x-d3_ccpvtz/A-int-1	-1642.981621	0.0
m062x-d3_ccpvtz/A-int-2	-1642.978250	8.9
m062x-d3_ccpvtz/B-int-1	-1642.928844	138.6
m062x-d3_ccpvtz/B-int-2	-1642.924521	149.9
pbe1pbe-d3_ccpvtz/A-int-1	-1641.747690	0.0
pbe1pbe-d3_ccpvtz/A-int-2	-1641.744086	9.5
pbe1pbe-d3_ccpvtz/B-int-1	-1641.693357	142.7
pbe1pbe-d3_ccpvtz/B-int-2	-1641.687567	157.9
wb97xd_ccpvtz/A-int-1	-1643.041316	0.0
wb97xd_ccpvtz/A-int-2	-1643.037671	9.6
wb97xd_ccpvtz/B-int-1	-1642.986198	144.7
wb97xd_ccpvtz/B-int-2	-1642.981183	157.9

With a different basis set (TZVP) the results are very similar:

b3lyp-d3_tzvp/A-int-2	-1643.635510	0.0
b3lyp-d3_tzvp/B-int-1	-1643.586982	127.4
b3lyp-d3_tzvp/B-int-2	-1643.581119	142.8
pbe1pbe-d3_tzvp/A-int-2	-1641.710242	0.0
pbe1pbe-d3_tzvp/B-int-1	-1641.661355	128.4
pbe1pbe-d3_tzvp/B-int-2	-1641.655515	143.7

Table S3. Absolute (Ha) and relative (kJ/mol) DFT energies of minimum energy conformations of intermediates, using four different functionals including dispersion: B3LYP-D3, M062X-D3 and PBE-D3, and cc-pVTZ basis sets. The calculations include the presence of dichloromethane as solvent (using the SCRF/PCM option in Gaussian09).

**Method/Molecule**	**Abs. energy (Ha)**	**Rel. energy (kJ/mol)**
b3lyp-d3_ccpvtz/A-int-1	-1643.687489	0.0
b3lyp-d3_ccpvtz/A-int-2	-1643.684299	8.4
b3lyp-d3_ccpvtz/B-int-1	-1643.633378	142.1
b3lyp-d3_ccpvtz/B-int-2	-1643.627527	157.4
m062x-d3_ccpvtz/A-int-1	-1642.996749	0.0
m062x-d3_ccpvtz/A-int-2	-1642.993427	8.7
m062x-d3_ccpvtz/B-int-1	-1642.943045	141.0
m062x-d3_ccpvtz/B-int-2	-1642.938508	152.9
pbe1pbe-d3_ccpvtz/A-int-1	-1641.761977	0.0
pbe1pbe-d3_ccpvtz/A-int-2	-1641.758296	9.7
pbe1pbe-d3_ccpvtz/B-int-1	-1641.706911	144.6
pbe1pbe-d3_ccpvtz/B-int-2	-1641.700944	160.2

Table S4. Cartesian coordinates of calculated structures of intermediates shown in Figure 12.

***anti*-1,2-coupling: mu-(μ)-isomer**

Standard orientation:

Center Number	Atomic Number	Atomic Type	Coordinates (Angstroms) X	Y	Z
1	6	0	-5.552073	-2.810477	-0.018280
2	6	0	-5.309096	-2.048908	1.121187
3	6	0	-4.097781	-1.380170	1.264929
4	6	0	-3.114455	-1.461147	0.275823
5	6	0	-3.367714	-2.219512	-0.870283
6	6	0	-4.578962	-2.892515	-1.010468
7	1	0	-6.062228	-1.972748	1.899367
8	1	0	-3.915221	-0.775358	2.149518
9	1	0	-2.620490	-2.284355	-1.654550
10	1	0	-4.761296	-3.482850	-1.903400
11	6	0	-1.849325	-0.648815	0.455248
12	1	0	-1.807007	-0.300733	1.493695
13	6	0	-0.529588	1.322091	-0.205752
14	6	0	-0.010527	1.802053	1.076059
15	7	0	0.490170	1.177629	-1.066430
16	6	0	1.583785	1.527865	-0.408715
17	8	0	-0.498599	2.033479	2.153396
18	8	0	1.365733	1.941498	0.853094
19	6	0	2.941651	1.422536	-0.894501
20	6	0	3.166418	0.887709	-2.171737
21	6	0	4.020799	1.783439	-0.076002
22	6	0	4.467668	0.733129	-2.628193
23	1	0	2.314758	0.593858	-2.776965
24	6	0	5.317545	1.624546	-0.545040
25	1	0	3.831284	2.173052	0.918729
26	6	0	5.542779	1.103493	-1.819086
27	1	0	4.646576	0.321312	-3.616180
28	1	0	6.155874	1.898604	0.087421
29	1	0	6.559081	0.980345	-2.180844
30	6	0	-5.551982	2.810582	0.018223
31	6	0	-5.309010	2.048982	-1.121222
32	6	0	-4.097714	1.380202	-1.264930
33	6	0	-3.114402	1.461166	-0.275809
34	6	0	-3.367656	2.219564	0.870276
35	6	0	-4.578884	2.892609	1.010427
36	1	0	-6.062132	1.972830	-1.899413
37	1	0	-3.915160	0.775364	-2.149502
38	1	0	-2.620444	2.284400	1.654555
39	1	0	-4.761215	3.482969	1.903343
40	6	0	-1.849295	0.648788	-0.455202
41	1	0	-1.806978	0.300696	-1.493646
42	6	0	-0.529645	-1.322168	0.205798
43	6	0	-0.010606	-1.802143	-1.076017
44	7	0	0.490115	-1.177761	1.066482
45	6	0	1.583730	-1.527926	0.408730
46	8	0	-0.498677	-2.033438	-2.153383
47	8	0	1.365673	-1.941475	-0.853107
48	6	0	2.941599	-1.422572	0.894500
49	6	0	3.166364	-0.887832	2.171772
50	6	0	4.020751	-1.783367	0.075956
51	6	0	4.467615	-0.733233	2.628221
52	1	0	2.314700	-0.594062	2.777035
53	6	0	5.317497	-1.624457	0.544987
54	1	0	3.831236	-2.172914	-0.918801
55	6	0	5.542728	-1.103492	1.819070
56	1	0	4.646520	-0.321484	3.616236
57	1	0	6.155827	-1.898435	-0.087506
58	1	0	6.559031	-0.980331	2.180822
59	1	0	-6.495457	3.335305	0.133438
60	1	0	-6.495563	-3.335166	-0.133520

syn-1,2-coupling: omega-(ω)-isomer

Standard orientation:

Center Number	Atomic Number	Atomic Type	Coordinates (Angstroms) X	Y	Z
1	6	0	3.795078	-2.397036	-2.956497
2	6	0	3.897530	-2.985131	-1.698271
3	6	0	2.987834	-2.651396	-0.701589
4	6	0	1.967396	-1.725042	-0.940769
5	6	0	1.869047	-1.143801	-2.206326
6	6	0	2.780420	-1.478363	-3.205100
7	1	0	4.685423	-3.702616	-1.490812
8	1	0	3.078573	-3.099834	0.284978
9	1	0	1.096248	-0.412951	-2.417212
10	1	0	2.695872	-1.009674	-4.180443
11	6	0	1.035952	-1.382558	0.211081
12	1	0	1.114119	-2.204265	0.931293
13	6	0	1.087099	1.192232	0.405994
14	6	0	1.648302	1.874885	-0.756638
15	7	0	0.016982	1.872159	0.847215
16	6	0	-0.119278	2.908428	0.036927
17	8	0	2.584119	1.684939	-1.492384
18	8	0	0.809695	2.986120	-0.926225
19	6	0	-1.147118	3.924087	0.113517
20	6	0	-2.096689	3.857558	1.142949
21	6	0	-1.197150	4.961542	-0.826767
22	6	0	-3.086464	4.825332	1.227678
23	1	0	-2.037710	3.044506	1.858864
24	6	0	-2.192784	5.924948	-0.732559
25	1	0	-0.458169	4.998276	-1.620096
26	6	0	-3.136310	5.859465	0.291885
27	1	0	-3.822226	4.776827	2.023914
28	1	0	-2.234680	6.729158	-1.459896
29	1	0	-3.912453	6.615256	0.361470
30	6	0	5.458461	-0.610310	2.579750
31	6	0	4.350006	-0.875951	3.378385
32	6	0	3.068409	-0.687196	2.869248
33	6	0	2.874625	-0.235423	1.561181
34	6	0	3.992846	0.022047	0.761868
35	6	0	5.274268	-0.161443	1.274479
36	1	0	4.480095	-1.225110	4.398200
37	1	0	2.203835	-0.891567	3.496927
38	1	0	3.861727	0.356617	-0.262261
39	1	0	6.133668	0.043864	0.643639
40	6	0	1.449250	-0.109097	1.052160
41	1	0	0.791112	-0.141318	1.927642
42	6	0	-0.418575	-1.349401	-0.147790
43	6	0	-1.137654	-0.426613	-1.022538
44	7	0	-1.286652	-2.224328	0.380856
45	6	0	-2.473946	-1.911436	-0.112539
46	8	0	-0.824264	0.521719	-1.703113
47	8	0	-2.466477	-0.859938	-0.950446
48	6	0	-3.724195	-2.582479	0.165289
49	6	0	-3.729718	-3.684117	1.033525
50	6	0	-4.916876	-2.140858	-0.424119
51	6	0	-4.923231	-4.335266	1.306835
52	1	0	-2.793808	-4.008153	1.476441
53	6	0	-6.105458	-2.800756	-0.143027
54	1	0	-4.897992	-1.287828	-1.094196
55	6	0	-6.111035	-3.895812	0.720407
56	1	0	-4.931279	-5.188229	1.977530
57	1	0	-7.030534	-2.461982	-0.597811
58	1	0	-7.042914	-4.408676	0.936860
59	1	0	6.460668	-0.752518	2.972466
60	1	0	4.503296	-2.653559	-3.738437

anti-1,3-coupling: alpha-(α)-isomer

Standard orientation:

Center Number	Atomic Number	Atomic Type	Coordinates (Angstroms) X	Y	Z
1	6	0	-2.758751	-3.733913	0.909093
2	6	0	-3.034569	-2.768584	1.874359
3	6	0	-2.255032	-1.619612	1.949541
4	6	0	-1.189178	-1.418430	1.067001
5	6	0	-0.921363	-2.388929	0.095981
6	6	0	-1.703500	-3.539501	0.023298
7	1	0	-3.857979	-2.907798	2.567938
8	1	0	-2.478996	-0.858173	2.691987
9	1	0	-0.099076	-2.246505	-0.596188
10	1	0	-1.484202	-4.286216	-0.733617
11	6	0	-0.414307	-0.117497	1.155135
12	1	0	-0.632553	0.362618	2.115187
13	6	0	-1.016190	0.965326	0.111491
14	6	0	-1.048464	0.334402	-1.290031
15	7	0	-2.429442	1.098877	0.455028
16	6	0	-3.094511	0.598555	-0.504277
17	8	0	-0.180469	0.009119	-2.041026
18	8	0	-2.376739	0.129991	-1.582886
19	6	0	-4.551136	0.454758	-0.585961
20	6	0	-5.328585	0.868227	0.499907
21	6	0	-5.153084	-0.093972	-1.719613
22	6	0	-6.709031	0.736011	0.445603
23	1	0	-4.833526	1.287229	1.370057
24	6	0	-6.537528	-0.223570	-1.765915
25	1	0	-4.537066	-0.416004	-2.552154
26	6	0	-7.314295	0.190657	-0.687171
27	1	0	-7.316290	1.056572	1.285967
28	1	0	-7.009516	-0.649418	-2.645383
29	1	0	-8.394333	0.087776	-0.726770
30	6	0	3.525445	3.711385	-0.671148
31	6	0	2.605618	4.504282	0.023438
32	6	0	1.332430	4.032878	0.275440
33	6	0	0.922214	2.742493	-0.155750
34	6	0	1.860422	1.968780	-0.886081
35	6	0	3.140666	2.450409	-1.123462
36	1	0	2.894489	5.489505	0.375991
37	1	0	0.628277	4.642885	0.834479
38	1	0	1.575641	0.999207	-1.274028
39	1	0	3.844283	1.832452	-1.674170
40	6	0	-0.376005	2.310833	0.222025
41	1	0	-0.983849	3.012065	0.784622
42	6	0	1.060341	-0.239470	1.081223
43	6	0	1.936834	0.593346	1.892066
44	7	0	1.793300	-1.054275	0.306030
45	6	0	3.053350	-0.756625	0.572959
46	8	0	1.726247	1.430136	2.731076
47	8	0	3.225302	0.200672	1.504217
48	6	0	4.220485	-1.336614	-0.056631
49	6	0	4.051412	-2.333858	-1.027995
50	6	0	5.506211	-0.898705	0.289351
51	6	0	5.164319	-2.888727	-1.642253
52	1	0	3.046411	-2.652408	-1.284283
53	6	0	6.612927	-1.461258	-0.332658
54	1	0	5.621329	-0.122976	1.039045
55	6	0	6.444925	-2.455067	-1.296183
56	1	0	5.036575	-3.660492	-2.394318
57	1	0	7.609638	-1.124610	-0.066563
58	1	0	7.313075	-2.891988	-1.779708
59	1	0	4.529010	4.078690	-0.860252
60	1	0	-3.365501	-4.632054	0.847023

syn-1,3-coupling: peri-isomer

Standard orientation:

Center Number	Atomic Number	Atomic Type	X	Y	Z
1	6	0	-3.862694	-3.095709	-1.843421
2	6	0	-3.318120	-2.241078	-2.798285
3	6	0	-2.227615	-1.439941	-2.473083
4	6	0	-1.679924	-1.475076	-1.188813
5	6	0	-2.222027	-2.344653	-0.238381
6	6	0	-3.308070	-3.149379	-0.567263
7	1	0	-3.740545	-2.196179	-3.797279
8	1	0	-1.811693	-0.763762	-3.214942
9	1	0	-1.820737	-2.366224	0.770488
10	1	0	-3.730772	-3.809959	0.183160
11	6	0	-0.538389	-0.537763	-0.849449
12	1	0	-0.085356	-0.199259	-1.789136
13	6	0	-1.013468	0.788354	-0.096480
14	6	0	-1.563393	1.748689	-1.166663
15	7	0	0.192595	1.476283	0.377262
16	6	0	0.272806	2.575007	-0.254042
17	8	0	-2.477700	1.661173	-1.926745
18	8	0	-0.716533	2.836788	-1.176848
19	6	0	1.307635	3.604729	-0.110300
20	6	0	2.343095	3.396023	0.805614
21	6	0	1.261925	4.772937	-0.873444
22	6	0	3.331239	4.359090	0.955002
23	1	0	2.354147	2.479117	1.386003
24	6	0	2.256163	5.733466	-0.717271
25	1	0	0.452774	4.922182	-1.580129
26	6	0	3.288487	5.527862	0.194129
27	1	0	4.136579	4.200995	1.664939
28	1	0	2.223863	6.643066	-1.308092
29	1	0	4.062685	6.279499	0.313508
30	6	0	-1.917756	0.600809	1.085496
31	6	0	0.589350	-1.169313	-0.104119
32	6	0	0.635061	-1.571830	1.294334
33	7	0	1.790752	-1.358982	-0.667640
34	6	0	2.562901	-1.858305	0.282172
35	8	0	-0.166926	-1.586290	2.196013
36	8	0	1.954346	-2.008508	1.473693
37	6	0	3.951192	-2.242039	0.147751
38	6	0	4.584444	-2.089963	-1.094384
39	6	0	4.658324	-2.758730	1.241997
40	6	0	5.915456	-2.453390	-1.234887
41	1	0	4.016479	-1.688389	-1.927028
42	6	0	5.990472	-3.119321	1.089374
43	1	0	4.156339	-2.872059	2.197071
44	6	0	6.620008	-2.967846	-0.145488
45	1	0	6.407990	-2.337200	-2.194807
46	1	0	6.540323	-3.520194	1.934639
47	1	0	7.661517	-3.251580	-0.259870
48	1	0	-4.714976	-3.719868	-2.094051
49	6	0	-3.312624	0.338234	1.172760
50	6	0	-3.858482	0.235932	2.480327
51	6	0	-4.197657	0.149865	0.080142
52	6	0	-5.199280	-0.033473	2.684950
53	1	0	-3.195173	0.365613	3.331222
54	6	0	-5.538588	-0.119962	0.298981
55	1	0	-3.824776	0.201039	-0.934700
56	6	0	-6.052551	-0.212658	1.593613
57	1	0	-5.585055	-0.108141	3.697097
58	1	0	-6.191924	-0.268688	-0.555508
59	1	0	-7.104980	-0.426392	1.751347
60	1	0	-1.383152	0.686545	2.024581
